# Proceedings of the 15th Annual Conference on the Science of Dissemination and Implementation in Health

**DOI:** 10.1186/s13012-023-01299-8

**Published:** 2023-10-05

## Introduction

### Gila Neta^1^, David A. Chambers^1^, Lisa Simpson^2^

#### ^1^National Cancer Institute, Rockville, MD, USA; ^2^AcademyHealth, Washington, DC, USA

As we reemerge from the acute phase of the COVID pandemic, top of mind is how best to rebuild our health systems to maximize effectiveness, efficiency, and equity, and what lessons we can learn moving forward. How can we be more proactive to better anticipate challenges and more rapidly overcome them? How can we be more nimble to adapt to rapidly changing circumstances and evidence? How can we be more responsive to the diverse needs of our communities to ensure equity? These significant challenges for the field framed the theme of the 15th Annual Conference on the Science of Dissemination and Implementation in Health: **(Re)Building Better Systems: Being Proactive, Nimble, and Responsive**.

Co-hosted by the National Institutes of Health and AcademyHealth in collaboration with our co-sponsors, the Agency for Healthcare Research and Quality (AHRQ), the Patient Centered Outcomes Research Institute (PCORI), the Robert Wood Johnson Foundation (RWJF), and the US Department of Veterans Affairs (VA), the conference brought together nearly 1400 researchers, practitioners, and other partners from around the world on December 11-14, 2022, in Washington, DC. Participants included 150 trainees, 8 patient scholarship recipients, and 31 participants from 13 low- and middle-income countries, including in sub-Saharan Africa, Latin America, South and Southeast Asia, Eastern Europe, and the Middle East. As we continue to enhance the diversity, equity, inclusion, and accessibility (DEIA) of the D&I Science community, we offered for the first time DEIA travel scholarships to 35 US-based participants, as well as travel scholarships to an additional 14 participants based in low- and middle-income countries.

The conference kicked off with an opening keynote from Dr. Kathleen Hall Jamieson on minimizing public susceptibility to misinformation and its impact on behavior and health. Drawing on Annenberg Public Policy Center research on health communication, Dr. Jamieson discussed ways to increase the likelihood that the public understands, accepts, and acts on the best available knowledge to support healthful decision-making and access to effective care. She outlined three key steps: 1) using consequential protective knowledge; 2) capsulizing language; and 3) reaching audiences in need. Consequential knowledge increases the likelihood of science-consistent behavior, can be identified, and minimizes susceptibility to misconception or deception. Dr. Hall Jamieson reviewed the complexity of terms related to infection in the COVID era and how our communication strategies fell short. For example, in a nationwide poll on the meaning of “m” in mRNA (when referring to the COVID19 vaccine) only 37 percent of respondents appropriately selected “messenger”.

In the plenary keynote on day two, Dr. Olugbenga Ogedegbe highlighted the need for “transcending limits and boundaries” of D&I science to improve health equity, exploring examples from research in low-income countries as well as low-resource settings in the United States. Two subsequent plenary panels focused on the importance of inclusion of diverse partner perspectives in D&I science and data systems to guide the next generation of studies. The former panel discussed front-line priorities and guidance, including the importance of building trust and engaging partners, to more effectively build better health and proactive, nimble, and responsive public health, healthcare and community systems. The latter panel explored issues around data systems and data sharing to enable understanding of ongoing implementation efforts and help focus our efforts to optimize access, quality, efficiency, and health outcomes at a population level. Both panels reflected on the many ways that an intentional focus on health equity and engagement is needed to improve care delivery and data systems.

To mark the 15^th^ anniversary of this event and the first in-person gathering of the community, the entire audience engaged in an interactive plenary to highlight the accomplishments of the field to date, prioritize challenges, and set goals for the next fifteen. Key themes that emerged included successes in raising awareness about the field, training our D&I science workforce, developing and iterating frameworks, advancing methods, and integrating an equity perspective.

The conference also included concurrent podium and poster sessions, workshops, discussion forums, and multiple networking events. For the first time, workshop proposals were solicited through the call for abstracts, yielding 24 submissions and resulting in five half-day pre-conference workshops. Additionally, the call for abstracts generated 887 submissions (an increase of 217 (32%) over last year), including individual paper presentations, individual posters, and panel presentations spread across nine thematic tracks: Behavioral Health, Clinical Care Settings (separated into two tracks: Patient-Level Interventions and System-Level Interventions), Global Dissemination and Implementation Science, Promoting Health Equity and Eliminating Disparities, Health Policy Dissemination and Implementation Science, Prevention and Public Health, and Models, Measures and Methods, and Building the Future of D & I Science: Training, Infrastructure, and Emerging Research Areas. As in prior years, the tracks with the greatest number of submissions were Clinical Care Settings: System-Level Interventions (23%) and Models, Measures, Methods (16%). Across the 8 years of the conference being organized by thematic tracks, we find that the field has increasingly focused on Promoting Health Equity and Eliminating Disparities and Building the Future, each of which had the largest increase in the proportion of submissions (125% and 111%, respectively). This supplement is organized by those tracks and includes 168 abstracts from the concurrent paper and panel sessions, which represents a variety of dissemination and implementation research funded by our conference sponsors as well as other agencies, organizations, and systems. The additional 586 abstracts from the poster sessions are not included here but can be viewed at https://academyhealth.confex.com/academyhealth/2022di/meetingapp.cgi/ModulePosterSessions/0.

The conference also featured a pre-recorded orientation with live Q&A, social musical gathering, yoga, fun run, meet the editors and experts sessions, ancillary meetings, and daily morning coffee chats with D&I experts facilitating open discussions about key priorities for the field. The networking sessions again were hugely popular and well attended, providing attendees with the opportunity to connect with the leaders in the field.

We look forward to welcoming attendees at the 16th Annual D&I Science conference this December 10-13 in Arlington, VA at the Crystal Gateway Marriott.

## Behavioral Health

### S1 Moderators of implementation strategy effectiveness to support cbt delivery at michigan high schools: results from the adaptive school-based implementation of cbt (asic) clustered-smart

#### Shawna Smith^1^, Daniel Almirall^2^, Seo Youn Choi^3^, Elizabeth Koschmann^4^, Amy Rusch^1^, Emily Bilek^4^, Annalise Lane^1^, James Abelson^4^, Daniel Eisenberg^5^, Joeseph Himle^3^, Kate Fitzgerald^6^, Celeste Liebrecht^3,4^, Carolyn Andrews^7^, Amy Kilbourne^4^

##### ^1^University of Michigan School of Public Health, Ann Arbor, MI, USA; ^2^University of Michigan Institute for Social Research, Ann Arbor, MI, USA; ^3^University of Michigan, Ann Arbor, MI, USA; ^4^University of Michigan Medical School, Ann Arbor, MI, USA; ^5^UCLA Fielding School of Public Health, Los Angeles, CA, USA; ^6^Columbia University Irving Medical Center/New York State Psychiatric Institute, Ann Arbor, MI, USA; ^7^University of Michigan, Ann Arbor, USA

###### **Correspondence:** Shawna Smith (shawnana@med.umich.edu)

*Implementation Science 2023,* **18(Suppl 3):**S1

**Background**

Youth mental health services are increasingly provided in schools. However, implementation of mental health evidence-based practices (EBPs) in schools remains deficient, in part due to heterogeneous barriers to school-based delivery of EBPs. While implementation strategies hold promise for addressing a variety of barriers, empirical knowledge as to which implementation strategies work best in which school settings remains lacking.

**Methods**

The Adaptive School-based Implementation of CBT (ASIC) study recruited 94 Michigan high schools to compare the effects of different sequences of implementation strategies (including some that adapt based on ongoing need) on school professional (SP) delivery of cognitive behavioral therapy (CBT) sessions over the 55-week study. After the run-in phase, all schools were randomized to receive skills-based Coaching or not. After 8 weeks, schools in both arms that were deemed “slower-responders” were re-randomized to either add Facilitation to their current support or not. Longitudinal multilevel models examined whether the effects of Coaching or (among slower-responders) Facilitation were moderated by school-level variables, including SPs’ formal CBT training, pre-randomization CBT delivery, baseline perceptions of CBT, school administrator support, and/or barriers to CBT delivery.

**Findings**

Overall, SPs in Coaching arm (vs. no Coaching) reported 0.8 fewer CBT sessions per week; among slower-responders, those who received Facilitation (vs. no Facilitation) reported 1.1 more CBT sessions per week. Coaching improved delivery more in schools where fewer SPs had formal CBT training (b_C*week*training_=-.92 [CI=-1.57,-.28]), and SPs did not deliver CBT pre-randomization (b_C*week*repCBT_=-.94; CI=-1.37,-.50). Facilitation (among slower-responders) improved delivery more in schools where SPs reported on average 2+ barriers prior to second randomization (b_F*week*barriers_=1.10; CI=-.89, 3.10), positive perceptions of CBT (b_F*week*perceptions_=.11; CI=-.25,.46), and lower school administrator support (b_F*week*support_=-.36; CI=-.69,-.03).

**Implications for D&I Research**

Successfully scaling up implementation support for EBPs in schools requires understanding how contextual factors differentially affect implementation strategy effectiveness. Moderator analyses can inform how to target and tailor implementation strategy provision by understanding the conditions under which strategies are most effective. Here, Coaching worked best when SP prior experience with CBT was minimal, while Facilitation was most effective when clear organizational barriers were apparent. The findings can help guide deploying implementation strategies to best fit the local needs of schools.

**Primary Funding Source**

The National Institute of Mental Health

### S2 Uncovering hypothesized mechanisms of action aimed to increase traumatic brain injury screening adoption in behavioral healthcare

#### Kathryn Hyzak, Uwe Wernekinck, Alicia Bunger

##### The Ohio State University, Columbus, OH, USA

###### **Correspondence:** Kathryn Hyzak (coxe.6@osu.edu)

*Implementation Science 2023,* **18(Suppl 3):**S2

**Background:** Implementation strategies that address barriers can accelerate the public health benefit of innovations in behavioral health settings, yet mechanisms of action that explain how strategies address these barriers remain unclear which limits our understanding of why implementation succeeds or fails. Guided by the Consolidated Framework for Implementation Research (CFIR) and Expert Recommendations for Implementing Change (ERIC), this study advances implementation strategy selection and identifies mechanisms of action for increasing traumatic brain injury (TBI) screening adoption in behavioral health contexts.

**Methods:** 20 licensed behavioral health providers in the U.S. (social workers, counselors, psychologists) participated in qualitative interviews about barriers to adopting TBI screening. Interview transcripts were managed in NVivo and co-coded using content analysis to identify barriers to TBI screening adoption and linked to CFIR domains and constructs. Next, using the CFIR/ERIC query tool, barriers were mapped to implementation strategies, and hypothesized mechanisms of action were identified based on census agreement between two coders.

**Findings:** In CFIR's ‘Inner-Setting’ domain, barriers included poor leadership engagement, low priority, and lack of organizational incentives/rewards, which mapped to conducting local consensus discussions, informing local opinion leaders, and funding/contracting for TBI screening. These strategies target knowledge-acquisition, awareness-building, norms/social pressures, and motivations (mechanisms). In CFIR’s ‘Outer-Setting’ domain, lack of external policies/incentives mapped to involving executive boards, obtaining formal commitments, and mandating changes. These strategies target norms/social pressures (mechanism). In CFIR’s ‘Characteristics-of-Individuals,’ barriers included lack of knowledge and awareness about TBI and low self-efficacy to conduct TBI screening, which mapped to conducting educational meetings, distributing educational materials, shadowing other experts, and conducting ongoing training. These strategies target awareness-building, knowledge-acquisition, and skill-development (mechanisms). In CFIR’s ‘Process’ domain, lack of stakeholder engagement mapped to identifying/preparing champions. This strategy targets norms/social pressures (mechanism).

**Implications for D&I Research:** This study advances implementation science by describing a systematic, data-driven approach to implementation strategy selection tailored to a TBI screening innovation implemented in behavioral healthcare contexts. This represents a fundamental step in the field where understanding of contextual determinants to TBI screening adoption can lead to more precise strategy selection, mechanism specification, and potential impact that can be tested in subsequent studies.

**Primary Funding Source**

National Institutes of Health

### S3 Pay-for-performance is a financing strategy that improved implementation effectiveness: results from a 25-site cluster-randomized type 3 hybrid trial

#### Bryan Garner^1^, Stephen Tueller^2^, Michael Bradshaw^2^, Kathryn Speck^3^, Denna Vandersloot^4^, Derek Satre^5^, James Ford^6^, Mat Roosa^7^, Mark Zehner^8^, Jackie Mungo^2^, Sarah McDaniel^2^, Richa Ruwal^2^, Carla Rash^9^

##### ^1^The Ohio State University, Columbus, OH, USA; ^2^RTI International, Research Triangle Park, NC, USA; ^3^University of Nebraska, Lincoln, Lincoln, NE, USA; ^4^University of Washington, Portland, OR, USA; ^5^University of California San Francisco, San Francisco, CA, USA; ^6^University of Wisconsin - Madison, Madison, WI, USA; ^7^Independen Consultant, Syracuse, NY, USA; ^8^University of Wisconsin, Center for Tobacco Research and Intervention, Madison, WI, USA; ^9^Univeristy of Connecticut, Farmington, CT, USA

###### **Correspondence:** Bryan Garner (bryan.garner@osumc.edu)

*Implementation Science 2023,* **18(Suppl 3):**S3

**Background:** Of high relevance to dissemination and implementation science is rigorous experimental research testing the effectiveness of strategies to improve the implementation of evidence-based practices (EBPs) in real-world practice settings. As part of a dual-randomized type 2 hybrid trial, which included 39 HIV service organizations (HSOs) across the United States, 78 HSO staff, and 824 client participants with HIV and a substance use disorder (SUD), a motivational interviewing-based brief intervention (MIBI) was found to be effective. However, the MIBI was only effective when implemented within the organization-level implementation condition that provided HSOs with (1) the training, feedback, and consultation (TFC) control strategy (i.e., online/workshop training, fidelity feedback, consultation meetings with MIBI expert) and (2) the Implementation and Sustainment Facilitation (ISF) experimental Strategy (i.e., monthly 30-60 minute team-focused facilitation meetings via Zoom), relative to the TFC strategy. Building on this research and prior research supporting the effectiveness and cost-effectiveness of a financing strategy called pay-for-performance (P4P; providing conditional economic incentives for meeting/exceeding pre-defined level of performance), this presentation highlights the main finding from a cluster-randomized type 3 hybrid trial testing P4P as a strategy for improving MIBI implementation beyond the TFC+ISF Strategy.

**Methods:** After obtaining institutional review board approval, 25 HSOs, as well as participating staff and clients, were cluster randomized to either the control strategy (TFC+ISF) or the experimental strategy (TFC+ISF+P4P). MIBI staff working at HSOs randomized to the experimental strategy had the opportunity to receive $10 USD per MIBI implemented, as well as $10 USD per MIBI implemented at or above the 80th percentile level of fidelity achieved as part of the dual-randomized type 2 hybrid trial. Guided by the Theory of Implementation Effectiveness, the primary implementation outcome measure was implementation effectiveness (i.e., the consistency and quality of MIBI implementation), a staff-level measure representing the standardized sum of the total number of MIBIs implemented and the total quality/fidelity scores.

**Findings:** The P4P strategy had a medium-sized impact (d = .47) that significantly (p = .001) improved the level of implementation effectiveness achieved by HSO’s trained MIBI staff.

**Implications for D&I Research:** P4P is a financing strategy that can improve implementation effectiveness.

**Primary Funding Source**

National Institutes of Health

### S4 Economic evaluation of two implementation strategies for training college mental health providers in interpersonal psychotherapy: a cluster randomized trial

#### Ramesh Raghavan^1^, Ellen Fitzsimmons-Craft^2^, G. Terence Wilson^3^, W. Stewart Agras^4^, Denise Wilfley^2^

##### ^1^New York University, New York, NY, USA; ^2^Washington University in St. Louis, St Louis, MO, USA; ^3^Rutgers University, New Brunswick, NJ, USA; ^4^Stanford University, Palo Alto, CA, USA

###### **Correspondence:** Ramesh Raghavan (raghavan@nyu.edu)

*Implementation Science 2023,* **18(Suppl 3):**S4

**Background:** Depression and eating disorders are among the most common mental disorders observed among among college students; interpersonal psychotherapy (IPT) is an evidence-based treatment for these conditions. Identifying cost-effective approaches to implementing IPT in college counseling centers is necessary.

**Methods:** We conducted a cluster randomized trial of two implementation strategies to train therapists in IPT at 24 college counseling centers. These centers were randomly assigned to either: (1) a strategy in which therapists were coached to train other staff to implement IPT (“train-the-trainer;” TTT, or experimental condition), or (2) a strategy comprising a workshop, therapy manual, and expert follow-up consultation (“expert consultation”). We used an activity-based costing survey and interviews to capture implementation costs at each site. IPT fidelity (adherence and competence) was assessed by auditing audio recordings of selected therapy sessions, and analyzed using linear mixed effects models.

**Findings:** Out of a total of 184 therapists trained in this study, 95 were in the TTT condition. Each counseling center spent a mean of $8,194 in training and supervision costs to train their one trainer (range: $7,042-$10,078). These trainers then trained between 2 and 9 therapists at their centers in IPT. Across all 12 TTT sites, overall mean costs to produce one therapist in this study was $3,407 (median= $3,077). Mean costs to produce one trained therapist using the expert consultation strategy was $2,055 (median costs = $1,932). Therapists in both training groups showed improvements over time in both adherence and competence, with effect sizes in the large range. There was no statistically significant difference in the magnitude of improvement between the experimental and control conditions in the adherence outcome. However, on the competence outcome, therapists in the TTT condition had 0.073 higher scores on competence compared to controls (95% CI, 0.008-0.138; *p* = .03). Each 1 unit improvement in therapist competence scores requires an investment of $19,033 using a TTT strategy and $19,386 using an expert consultation strategy.

**Implications for D&I Research:** The TTT implementation strategy is a fiscally sound approach for IPT training. Despite higher short run costs, it results in more competent therapists and permits training of new therapists within the site over time.

**Primary Funding Source**

National Institutes of Health

### S5 Effects of the leadership and organizational change for implementation (LOCI) strategy on the implementation of digital measurement-based care in mental health clinics serving youth: A mechanistic analysis within a cluster RCT

#### Nate Williams^1^, Steven Marcus^2^, Mark Ehrhart^3^, Nallely Ramirez^1^, Kristine Carandang^4^, Marisa Sklar^4^, Lauren Brookman-Frazee^5^, Susan Esp^1^, Alexandra Wilson^1^, Gregory Aarons^5^

##### ^1^Boise State University, Boise, ID, USA; ^2^University of Pennsylvania, Philadelphia, PA, USA; ^3^University of Central Florida, Orlando, FL, USA; ^4^University of California, San Diego, La Jolla, CA, USA; ^5^UC San Diego ACTRI Dissemination and Implementation Science Center, La Jolla, CA, USA

###### **Correspondence:** Nate Williams (natewilliams@boisestate.edu)

*Implementation Science 2023,* **18(Suppl 3):**S5

**Background**

The aims of this cluster randomized, hybrid Type III effectiveness-implementation trial were to: (1) test the main effects of the Leadership and Organizational Change for Implementation (LOCI) strategy on implementation climate, clinician motivation, and clinician implementation of digital measurement-based care (MBC), and (2) test a theorized contextual-motivational mechanism underlying LOCI.

**Methods**

A total of 252 clinicians working in 21 outpatient mental health clinics in three states received training and technical assistance to implement a well-established digital MBC system. Clinics were randomly assigned to the 12-month LOCI strategy (n=11) or active control (leadership webinars) (n=10). Clinicians completed surveys assessing clinic implementation climate, intentions, and use of the digital MBC system during the 12-month LOCI intervention period and at 6-month follow-up (18-months post-baseline). Mixed effects regression models accounting for nesting of observations within clinicians and clinicians within clinics tested whether LOCI led to greater improvement in study variables over time. Multilevel mediation analysis tested whether LOCI’s effects on digital MBC implementation at 6-month follow-up were transmitted through a hypothesized contextual-motivational mechanism in which improved clinic implementation climate was theorized to increase clinician motivation which in turn was expected to increase implementation behavior.

**Findings**

In main effects analyses, LOCI significantly improved clinic climate for digital MBC implementation by 4-months post-baseline and this effect was sustained through 6-month follow-up (18-months post-baseline). Clinician intentions to use the digital MBC system were significantly improved at 8- and 12-months post-baseline. Overall, clinician use of the digital MBC system was low throughout the study, likely influenced by the exigencies of the COVID-19 pandemic; however, LOCI significantly improved clinicians’ self-reported use of digital MBC by 8-months post-baseline and this effect was sustained through follow-up (18-months after baseline). Mediation analysis indicated LOCI’s effect on clinicians’ implementation behavior at 18-months was transmitted through improvement in climate at 4-months and improvement in clinician intentions at 12-months.

**Implications for D&I Research**

Organizational leadership- and climate-focused strategies such as LOCI improve the implementation of digital health technologies by creating motivating clinic contexts that are robust even as healthcare systems face significant external shocks (i.e., the COVID pandemic).

**Primary Funding Source**

National Institutes of Health

### S6 Implementing chronic care treatment for tobacco cessation into community mental health care

#### Sandra Japuntich^1^, Sarah Helseth^2^, Melissa Adkins-Hempel^3^, Nathalia Gutierrez Sacasa^4^, Rebekah Pratt^5^, Steven Fu^6^, Jennifer Tidey^7^, A. Eden Evins^8^, Shira Dunsiger^7^, Sara Becker^7^

##### ^1^Hennepin Healthcare, Minneapolis, MN, USA; ^2^Brown University, Providence, RI, USA; ^3^Hennepin Healthcare Research Institute, Minneapolis, MN, USA; ^4^Hennepin Healthcare Research, Minneapolis, MN, USA; ^5^University of Minnesota, Minneapolis, MN, USA; ^6^VA Minneapolis Healthcare Center Minneapolis, MN, USA; ^7^Brown School of Public Health, Providence, RI, USA; ^8^Harvard University, Boston, MA, USA

###### **Correspondence:** Sandra Japuntich (sandra.japuntich@hcmed.org)

*Implementation Science 2023,* **18(Suppl 3):**S6

**Background:** Tobacco dependence is a chronic disease. Individuals with serious mental illness (SMI) have high tobacco use prevalence and tobacco-related mortality. Certified community behavioral health clinics (CBHC) provide care for people with SMI, yet delivery of tobacco cessation interventions is limited.

**Methods:** We will present implementation outcomes from a type 2 hybrid implementation-effectiveness trial testing a chronic care tobacco cessation intervention delivered in a CBHC. The intervention was the US Public Health Service guideline 5As (Ask about tobacco use, Advise to quit, Assess interest, Assist with quitting, Arrange for follow-up). The implementation strategy was the New England ATTC’s multi-level “Science to Service Laboratory” model including a 2-hour virtual/synchronous training followed by 9 months of CBHC-wide performance feedback (i.e., chart documented tobacco treatment) and monthly coaching calls. Feasibility was assessed via medical record review and provider survey (from training attendees) at post-training, 6 and 12 months (data available 10/22).

**Findings:** 76 CBHC clinicians attended didactic training. Coaching calls were provided monthly at team meetings; average attendance was 9.2 providers/call (22 calls). Post-training, clinician knowledge of and confidence in providing tobacco cessation treatment increased (ps <.05). At 6 months, clinicians reported increases in Advise and Assist behaviors (ps<.05), but not Assess or Arrange (Ask not collected). Medical record data evidenced high levels of Ask and Assess but low levels of Assist amongst providers (regardless of training attendance) with no changes over the reporting period. The organization implemented a tobacco use clinical reminder. Clinicians surveyed reported improvements in availability of tobacco cessation materials and referral processes 6 months post-trainings.

**Implications for D&I Research:** A multi-level implementation strategy consisting of didactic training, performance feedback and coaching was feasible and demonstrated preliminary effectiveness in improving tobacco cessation services in CBHCs. Clinicians who attended the training demonstrated increases in knowledge, confidence, and 5As behaviors over the implementation period. In addition, providers reported improved systems to address tobacco use including medical record prompts, resources, and referral options. Regardless of training attendance, all CBHC clinicians demonstrated high levels of Asking and Assessing tobacco use, but lagged in assisting with tobacco cessation. This study provides preliminary evidence that clinicians at CHBCs can deliver effective tobacco treatment.

**Primary Funding Source**

National Institutes of Health

### S7 Client experiences of an innovative mobile crisis model: qualitative evaluation of San Francisco’s street crisis response team

#### Rachel Odes^1^, Megan McDaniel^2^, Deepa Manjanatha^2^, Paige Lerman^3^, Siva Sundaram^3^, Janet Myers^3^, Matthew L. Goldman^2,3^

##### ^1^National Clinician Scholars Program, San Francisco, CA, USA; ^2^San Francisco Department of Public Health, San Francisco, CA, USA; ^3^University of California San Francisco, San Francisco, CA, USA

###### **Correspondence:** Rachel Odes (Rachel.Odes@ucsf.edu)

*Implementation Science 2023,* **18(Suppl 3):**S7

**Background:** The Substance Abuse and Mental Health Services Administration (SAMHSA) recommends including mobile crisis teams in the continuum of care to facilitate access to behavioral health services while reducing reliance on law enforcement to intervene crises that happen in public spaces. In 2020 San Francisco implemented the Street Crisis Response Team (SCRT), comprised of on-call clinicians, paramedics and peers who can provide specialized de-escalation, referral, and support services. The current study, guided by the Consolidated Framework for Implementation Research (CFIR), describes clients’ experiences of SCRT encounters.

**Methods:** This qualitative investigation is one arm of a mixed-methods evaluation of SCRT. We interviewed 20 adults who recently received SCRT services. Interviews were conducted during September 2021 – March 2022. Participant ages ranged from 27 to 64 years (mean=45). The sample was 75% male and was racially diverse: Black (45%), white (30%), Latino/a (20%), and Asian (5%). Semi-structured interviews lasted approximately one hour; questions were informed by CFIR domains. We used qualitative methods to code transcripts and thematic analysis to interpret findings.

**Findings:** Clients described their needs/goals, including access to medical care, case management, and housing services, and pointed out that SCRT was one channel for attaining placements or referrals. Relative advantage was a key theme for SCRT clients, who frequently pointed out that SCRTs’ “gentle” approach made encounters preferable to those involving emergency medical providers or law enforcement. Within the inner setting, SCRT team members’ personal characteristics, including the availability of a peer counselor with experience of the mental health system, helped clients feel supported. SCRT encounters incorporating basic support like food and water provided an immediate, sensitive response to crisis, although underlying housing instability often remained.

**Implications for D&I Research:** This study identified SCRT’s benefits for clients, but also described ways service needs of people experiencing homelessness are often more than what a mobile crisis program can provide. These findings are highly relevant to inform mobile crisis team implementation in communities seeking to improve access to behavioral health services. Stakeholders should look carefully at how program success is defined and incorporate clients’ goals. Consideration of CFIR domains can facilitate identification of which specific program elements improve outcomes.

**Primary Funding Source**

The Robert Wood Johnson Foundation

### S8 Qualitative study of telehealth delivery of “suicide-specific” group treatment “project life force”

#### Marianne Goodman^1^, Sapana Patel^2^, Barbara Stanley^3^, Angie Waliski^4^

##### ^1^Veterans Health Administration, BRONX VA, NY, USA; ^2^Columbia University Department of Psychiatry, New York, NY, USA; ^3^The New York State Psychiatric Institute and Columbia University, New York, NY, USA; ^4^Veterans Health Administration, Little Rock VA, USA

###### **Correspondence:** Marianne Goodman (mggoodman2001@yahoo.com)

*Implementation Science 2023,* **18(Suppl 3):**S8

**Background:** Minimal evidence exists for “suicide-specific” group treatment for high-risk patients offered over telehealth. This qualitative study assessed the acceptability, feasibility and impact of a group suicide safety planning intervention (SPI) offered over telehealth.

**Methods:** High-risk suicidal Veterans (n=17) participating in “Project Life Force telehealth” (PLF-T); a manualized, 10-session SPI group, completed semi-structured qualitative interviews including measures of acceptability, appropriateness, and feasibility. We also interviewed PLF-T group facilitators and coordinator to identify adaptations to deliver PLF-T and learn about barriers and facilitators to implementation. A summary template and matrix analysis approach were used to analyze qualitative data.

**Findings:** Group participants were mostly male (88%), age 50 (SD=15.6), ethnically diverse, and either divorced or separated (54%). Suicide symptoms upon study entry included past month ideation with methods (100%) and past year aborted, interrupted or actual suicide attempt (59%). Interviews revealed overall positive endorsement of PLF-telehealth with enhanced suicidal disclosure, improved ability to manage urges and mitigate loneliness. On scales from 1-20, PLF-T was rated as highly acceptable (M=17.50; SD=2.92), appropriate (M=17.25; SD=3.59), and feasible (M=18; SD=2.45). Adaptations to deliver PLF-T included using a communications coordinator to conduct assertive outreach to facilitate engagement, instituting a telehealth orientation session and restructuring sessions to review suicide severity and screen sharing safety plans to maximize learning. PLF-T enhanced convenience and access without compromising safety. Concerns included privacy, and technological limitations including connectivity. In conclusion, our findings suggest that suicide-specific safety planning group treatment is acceptable and feasible to deliver via telehealth.

**Implications for D&I Research:** Information about barriers and facilitators implementing PLF-T with this small sample of high-risk Veterans provides an opportunity to develop implementation strategies to support future implementation and scale up of suicide safety planning group telehealth treatment. Strategies may include creating an easy-to-access repository for all intervention materials, evaluating criteria for enrollment into PLF-T based on access to technology, comfort in group therapy and with using technology and participating in groups online. Other strategies may include sharing best practices for telehealth and establishing a consortium of support for research and clinical programs navigating regulations and policies for telehealth treatment.

**Primary Funding Source**

Department of Veterans Affairs

### S9 Peer recovery specialist-delivered, behavioral activation intervention to improve retention in methadone treatment: results from an open-label, type 1 hybrid effectiveness-implementation pilot trial

#### Jessica Magidson^1^, Valerie Bradley^1^, Mary Kleinman^1^, Morgan Anvari^2^, Abigail Hines^3^, Annabelle Belcher^4^, Aaron Greenblatt^4^, Tolulope Abidogun^3^, Dwayne Dean^3^, Cj Seitz-Brown^3^, Michael Wagner^2^, Melanie Bennett^4^, Julia Felton^5^

##### ^1^University of Maryland College Park, College Park, MD, USA; ^2^University of Maryland, College Park, USA; ^3^University of Maryland, College Park, MD, USA; ^4^University of Maryland, Baltimore, USA; ^5^Henry Ford Health System, Detroit, MI, USA

###### **Correspondence:** Jessica Magidson (jmagidso@umd.edu)

*Implementation Science 2023,* **18(Suppl 3):**S9

**Background:** Despite the efficacy of medication treatment for opioid use disorder (OUD), retention is an urgent priority, particularly among low-income, minoritized populations. Peer recovery specialists may be well-positioned to engage vulnerable patients in care, particularly when trained in an evidence-based intervention to promote retention. This Type 1 hybrid effectiveness-implementation pilot trial aimed to demonstrate the proof-of-concept of a peer recovery specialist-delivered behavioral activation approach (*Peer Activate*) to improve methadone retention.

**Methods:** Implementation outcomes were feasibility, acceptability, and fidelity, guided by Proctor’s model for defining implementation outcomes. Feasibility and acceptability were defined by the percentage of participants who initiated the intervention (≥75%) and completed ≥75% of core sessions, respectively. We also used a validated quantitative measure of feasibility and acceptability—the Applied Mental Health Research (AMHR) assessment (0-3 scale; Haroz et al., 2019). Fidelity was assessed via independent rating of a randomly selected 20% of sessions. The primary effectiveness outcome was methadone retention at three-months post-intervention vs. a comparison cohort initiating methadone during the same time period. Secondary effectiveness outcomes included substance use frequency and related problems.

**Findings:** Benchmarks for feasibility and acceptability were surpassed: 86.5% (32/37) initiated the intervention, and 81.3% of participants who initiated attended ≥75% of core sessions. The AMHR quantitative assessment indicated high levels of feasibility (*M*=2.04, *SD*=0.43) and acceptability (*M*=2.92, *SD*=0.19). The mean independent rater fidelity score was 87.9%, indicating high peer fidelity. For effectiveness outcomes, 88.6% of participants in *Peer Activate* were retained in methadone treatment at three-months post-intervention—28.9% more than individuals initiating methadone in the same time period [*x*^2^(1)=10.10, *p*=0.001]. There was a significant reduction in substance use frequency [*t*(25)=1.82, *p*=.041], and substance use-related problems [*t*(21)=1.84, *p*=0.040] among participants who completed the core *Peer Activate* sessions (*n*=26).

**Implications for D&I Research:** Given the rapid scale-up of peer recovery specialist programs nationwide, these results, although preliminary, have important implications for how D&I research can support the implementation of evidence-based peer programs to support individuals with OUD. The next steps are to conduct a larger Type 1 hybrid effectiveness-implementation randomized trial with longer-term follow-ups to further establish the implementation and effectiveness of the *Peer Activate* approach.

**Primary Funding Source**

National Institutes of Health

### S10 Implementing electronic measurement-based care in outpatient substance use disorder treatment settings

#### Megan O'Grady^1^, Patricia Lincourt^2^, Shazia Hussain^2^, Vanessa Bobadilla^3^, Jacqualin Ross^2^, Sueun Hong^3^, Charles Neighbors^4^

##### ^1^University of Connecticut School of Medicine, Farmington, CT, USA; ^2^NYS OASAS, Albany, USA; ^3^NYU Langone, New York, USA; ^4^New York University, New York, NY, USA

###### **Correspondence:** Megan O'Grady (ogrady@uchc.edu)

*Implementation Science 2023,* **18(Suppl 3):**S10

**Background:** Measurement-based care (MBC), routinely measuring and reviewing treatment progress with a standardized tool, can help inform clinical decision making. MBC has transformed mental health care, however the development and implementation of MBC for substance use disorder (SUD) treatment settings has been limited. While MBC measures for SUD treatment have recently been developed, putting them into user-friendly electronic formats and understanding their implementation is an existing knowledge gap. Study goals are to 1) describe user-centered development of an electronic version of the Treatment Progress Assessment-8 (eTPA8) for SUD care and 2) evaluate early implementation of the eTPA8.

**Methods:** Iterative user-centered development of the eTPA8 used cognitive interviewing, usability testing, and focus groups with SUD treatment clients and staff. The eTPA8 has been implemented in outpatient SUD clinics using an external practice facilitation approach guided by the Integrated Promoting Action on Research Implementation in Health Services (i-PARIHS) framework as part of a larger stepped-wedge trial. Semi-structured interviews (n = 35) with SUD program staff in 14 outpatient treatment clinics evaluated early eTPA8 implementation. Interviews were analyzed using conventional content analysis. The four core i-PARIHS constructs (Facilitation, Innovation, Context, and Recipients) are used to organize results.

**Findings:** Overall, SUD program staff find the eTPA8 user-friendly. There is variation among staff in the perceived utility of the eTPA8, especially given competing demands and time constraints. Staff find the eTPA8 a clinically relevant tool and use it to support clinical interactions, but vary in embracing new technology and the overall MBC concept. The inner (e.g., leadership support and priorities) and outer contexts (e.g., COVID-19 and staff shortages) influence implementation and require flexibility by practice facilitators and implementation teams. External practice facilitators and clinic implementation champions are key implementation supports, particularly in addressing barriers.

**Implications for D&I Research:** The eTPA8 is a promising MBC tool. It was viewed positively by SUD treatment programs, but requires intensive implementation supports (e.g., champions, facilitators) that are dynamic, proactive, and responsive. Findings have implications for guiding further development and refinement of responsive, theory-driven implementation strategies to support MBC in behavioral health settings. Future research should also investigate how MCB is sustained after initial implementation.

**Primary Funding Source**

National Institutes of Health

### S11 The mental health and addiction treatment tracking repository: How big data on substance use disorder treatment facilities can inform implementation research, practice, and policy

#### Jonathan Cantor^1^, Alex Dopp^1^, Aaron Kofner^2^, Maria DeYoreo^1^, Mark Godley^3^, Bing Han^4^, Sarah Hunter^5^, Beau Kilmer^5^, Rosanna Smart^1^

##### ^1^RAND Corporation, Santa Monica, CA, USA; ^2^RAND Corporation, Arlington, VA, USA; ^3^Chestnut Health, Normal, USA; ^4^Kaiser Permanente Southern California, Pasadena, USA; ^5^RAND Corporation, Santa Monica, USA

###### **Correspondence:** Jonathan Cantor (jcantor@rand.org)

*Implementation Science 2023,* **18(Suppl 3):**S11

**Background:** The United States has faced a worsening drug overdose crisis. There is limited data on the supply of substance use disorder treatment (SUDT) facilities, especially regarding key details like use of evidence-based practices (EBP) (e.g., provision of medication for opioid use disorder [MOUD]) and financing mechanisms (e.g., Medicaid expansion). Well-organized data on licensed SUDT facilities are critical to informing policy and practice improvement efforts.

**Methods:** We digitized records from the U.S. National Directory of Drug and Alcohol Abuse Treatment Facilities between 1975 and 2022 to create the Mental health and Addiction Treatment Tracking Repository (MATTR). MATTR also includes daily downloads of the Behavioral Health Treatment Locator since 2019. We geocoded each dataset and linked the data across years. Each facility is assigned a unique identifier to track the opening, closing, and changes in populations served, accepted forms of payment, and treatment services over time. To illustrate how MATTR can inform implementation, we quantified changes in the offering of MOUD and acceptance of Medicaid between 2012 and 2021. We also quantified the number of new or closing facilities that reported offering MOUD or accepting Medicaid in each year.

**Findings:** Between 2012 and 2021, the MATTR contained 27,699 unique SUDT facilities, with 14,581 openings and 14,073 closings. Rates of Medicaid acceptance increased from 2012 to 2021 (54.5% to 71.8%). There was a substantial increase in the rate of offering MOUD going from 22.1% to 51.2% between 2012 and 2021. In 2021, 54% of facilities that opened offered MOUD, and separately 66% accepted Medicaid as a form of payment. In 2020, 46% of the facilities that closed offered MOUD in the previous year, and 62.3% accepted Medicaid as a form of payment.

**Implications for D&I Research:** The MATTR data offer, almost in real-time, surveillance information on SUDT facilities. These data can be used to target the implementation of EBP at specific facilities and guide efforts to understand the facilitators and barriers to offering these services. This unique combination of big data and implementation science can support implementation researchers, practitioners, and policymakers in determining where, when, and how to target supports for implementation and sustainment of SUDT EBP.

**Primary Funding Source**

National Institutes of Health

### S12 Implementing interventions to connect parents involved in the child welfare system to substance use disorder treatment: geographic variations in implementation outcomes

#### Mary Finley^1^, Alicia Bunger^1^, Elinam Dellor^1^, Bridget Freisthler^1^, Fawn Gadel^2^, Jennifer Millisor^2^, Jen McClellan^2^, Kathryn Lancaster^1^, Marla Himmeger^2^

##### ^1^The Ohio State University, Columbus, OH, USA; ^2^Public Children Services Association of Ohio, Columbus, OH, USA

###### **Correspondence:** Mary Finley (finley.232@buckeyemail.osu.edu)

*Implementation Science 2023,* **18(Suppl 3):**S12

**Background:** Rural and Appalachian communities with limited behavioral health resources and significant geographic barriers to care may struggle to implement and deliver interventions that improve behavioral health service access and outcomes. However, implementation outside of urban contexts has received limited empirical attention. This study compares fidelity and timeliness across urban, suburban, rural, and Appalachian public children services agencies (PCSAs) that are implementing Ohio START (Sobriety, Treatment, and Reducing Trauma), an intervention for families affected by caregiver substance use disorder (SUD) and child maltreatment.

**Methods:** Preliminary data were drawn from administrative records of 556 caregivers with an open child welfare case and enrolled in Ohio START from forty PCSAs between March 2019 and January 2022. Fidelity was measured as the number of essential START components that caregivers received (SUD screening, family peer mentor visits, shared decision-making meetings, SUD treatment visits). Timeliness was measured as the number of days between the case opening date and each component. ANOVA and Kruskal-Wallis tests were used to compare fidelity and timeliness for caregivers from urban, suburban, rural, and Appalachian PCSAs.

**Findings:** Fidelity to START was strong; on average, caregivers received 3.6 components and 66% received all 4. There were no geographic differences in fidelity to screening, family peer mentor visits, or shared decision-making meetings. However, fewer parents received treatment in Appalachian PCSAs (65.91%) compared to parents in rural (84.62%, Tukey HSD, p=.002) or suburban PCSAs (84.68%, Tukey HSD, p=.002). In terms of timeliness, caregivers from Appalachian PCSAs waited longer (22.6 days) to be screened than those in rural PCSAs (7.4 days) (H(3)=23.31, p=<.001). Caregivers from Appalachian PCSAs also waited longer to connect to substance use disorder treatment (on average 49 days) compared to those in rural (48.6 days) (H(3)=18.305, p= <.001, and suburban (46 days) PCSAs [X2 (3)=18.33,p=<.001] although this difference might be due to a few extreme cases in Appalachian PCSAs.

**Implications for D&I Research:** Available resources and community context can shape implementation of interventions that promote behavioral health service access. Implementation strategies that support behavioral health service expansion and availability might be needed to optimize the public health benefit for Appalachian and other resource constrained communities.

Acknowledgements: RWJF, NIDA, PCSAO

**Primary Funding Source**

The Robert Wood Johnson Foundation

### S13 Advancing pharmacological treatments for opioid use disorder: outcomes of an implementation trial in eight veterans health administration facilities

#### Hildi Hagedorn^1,2^, Allison Gustavson^1^, Princess Ackland^1^, Ann Bangerter^1^, Mark Bounthavong^3^, Barbara Clothier^1^, Alex H.S. Harris^4^, Marie Kenny^1^, Siamak Noorbaloochi^1^, Hope Salameh^1^, Adam Gordon^5^

##### ^1^Center for Care Delivery & Outcomes Research, Minneapolis, MN, USA; ^2^University of Minnesota Medical School, Minneapolis, MN, USA; ^3^Health Economics Resource Center, Menlo Park, CA, USA; ^4^Center for Innovation to Implementation, Menlo Park, CA, USA; ^5^VA Salt Lake City Health Care System, Salt Lake City, UT, USA

###### **Correspondence:** Hildi Hagedorn (hildi.hagedorn@va.gov)

*Implementation Science 2023,* **18(Suppl 3):**S13

**Background:** Within the Veterans Health Administration (VHA), efforts over the past decade to improve access to medication treatments for opioid use disorder (MOUD) have resulted in a substantial increase in MOUD provision. However, facility-level provision of MOUD continues to be highly variable, requiring development and testing of implementation strategies that target low-performing facilities. This study aimed to determine the effectiveness of external facilitation in increasing the provision of MOUD among low-performing VHA facilities.

**Methods:** VHA facilities in the lowest quartile of MOUD provision (35 facilities) were identified. Eight of these facilities were randomly assigned to participate in the intervention with the remaining 27 facilities serving as matched controls. Intervention facilities participated in a 12-month external facilitation intervention which included assessment of local barriers/facilitators, formation of a local implementation team, a site visit for action planning and training/education, monthly coaching calls, quarterly cross-facility community of practice calls, and on-demand consultation. Outcome measures included pre- to post-change in: 1) facility-level ratio of patients with opioid use disorder (OUD) receiving MOUD, and 2) the number of patients with OUD prescribed buprenorphine/naloxone, both compared to matched control facilities.

**Findings:** Intervention facilities significantly increased the facility-level ratio of patients with OUD receiving MOUD from an average of 18% at baseline to 30% one-year later, with an absolute difference of 12% (95% Confidence Interval [CI]: 6.6%, 17.0%). The difference-in-differences between intervention and control facilities was 3.0% (95% CI: -0.2%. 6.7%). Intervention facilities significantly increased the number of patients prescribed buprenorphine/naloxone from pre- to post-implementation, with a mean increase of 41.8 new patients per facility (95% CI: 18.3, 61.0). The difference-in-differences between intervention and control facilities was 13.2 (95% CI: -4.8, 31.0). Sensitivity analysis excluding one outlier site demonstrated significantly greater changes in both outcome variables for the remaining seven intervention sites compared to their matched controls.

**Implications for D&I Research:** Healthcare systems interested in increasing adoption of MOUD, or other evidence-based practices, may consider a tiered approach where less resource intensive interventions (e.g., education, training) are initially employed, followed by more resource intensive, context-specific interventions for facilities that do not respond to initial efforts.

**Primary Funding Source**

Department of Veterans Affairs

### S14 Reach and equity of buprenorphine receipt following the stepped care for opioid use disorder train-the-trainer (scoutt) initiative in va primary care clinics

#### Eric Hawkins^1,2^, Carol Malte^2^, Brittany Blanchard^4^, Emily Williams^1,3,5^, Hildi Hagedorn^6^, Adam Gordon^7^, Andrew Saxon^2^

##### ^1^Center of Innovation for Veteran-Centered and Value-Driven Care, Seattle, WA, USA; ^2^VA Center of Excellence in Substance Addiction Treatment and Education, Seattle, WA, USA; ^3^VA Puget Sound Health Care System, Seattle,WA, USA; ^4^University of Washington, Seattle, USA; ^5^VA Puget Sound Health Care System, Health Services Research & Development, Center of Innovation for Veteran-Centered & Value-Driven Care, Portland, OR; ^6^Center for Care Delivery & Outcomes Research, Minneapolis, MN, USA; ^7^VA Salt Lake City Health Care System, Salt Lake City, UT, USA

###### **Correspondence:** Eric Hawkins (eric.hawkins@va.gov)

*Implementation Science 2023,* **18(Suppl 3):**S14

**Background**

Buprenorphine, a first-line medication treatment for OUD, reduces opioid overdose and mortality, and can be offered in primary care. However, it is underutilized and inequitably accessed across subgroups of patients with OUD. In 2018 the Department of Veterans Affairs (VA) implemented the Stepped Care for Opioid Use Disorder Train-the-Trainer (SCOUTT) initiative to improve access to buprenorphine nationally in general healthcare clinics such as primary care, using a multifaceted implementation strategy. Healthcare inequalities are common and can persist or worsen, after implementation of innovative treatments. Therefore, we evaluated the reach and equity of buprenorphine receipt following the SCOUTT initiative.

**Methods**

This prospective evaluation followed a cohort of patients with OUD seen in 17 VA primary care clinics in the year following August 2018. Main measures included Reach, defined as the proportion of patients seen in primary care with an OUD who received buprenorphine, and equitable reach of SCOUTT across social identity (age, sex, marital status, race, ethnicity, housing instability) and clinical characteristics (mental health, substance use, medical diagnoses) extracted from the heath record. Multivariable logistic regression was used to estimate the likelihood of buprenorphine receipt as a function of different characteristics, adjusted for all other characteristics.

**Findings**

Overall, 2,495 patients with an OUD were seen in participating primary care clinics; 10.8% received buprenorphine in primary care clinics. Approximately, 73.0% of patients were >50 years old, 7.6% were women, and 32.1% were Black, Indigenous, and people of color (BIPOC). In adjusted analyses, patients who received buprenorphine in primary care, compared to those who did not, were more likely to have housing instability (AOR=1.52, 95%CI: 1.06-2.17), sedative use disorder diagnosis (AOR=2.09, 95%CI: 1.24-3.52), any mental health diagnosis (AOR=1.54, 95%CI: 1.10-2.17), and past-year SUD specialty attendance (AOR=2.49, 95%CI: 1.73-3.59). Patients receiving buprenorphine were less likely to be BIPOC (AOR=0.61, 95%CI: 0.44-0.86) or have documented alcohol use disorder (AOR=0.33, 95%CI: 0.23-0.48), relative to those not receiving buprenorphine.

**Implications for D&I Research**

The SCOUTT initiative was implemented equitably across most social identity and clinical subgroups. However, results highlight the importance of monitoring access discrepancies for racially minoritized Veterans and those with alcohol use disorder during implementation efforts.

**Primary Funding Source**

Department of Veterans Affairs

### S15 Four years of external facilitation in the veterans health administration’s stepped care for opioid use disorder train the trainer (SCOUTT) initiative: Stability, innovation, and expansion

#### Jacob Baylis^1^, Malorie Carter^2^, Spencer Calder^3^, Marie Kenney^4^, Eric Hawkins^5^, Hildi Hagedorn^6^, Adam Gordon^1^

##### ^1^VA Salt Lake City Health Care System, Salt Lake City, UT, USA; ^2^University of Utah, Salt Lake City, USA; ^3^Veterans Health Administration, Salt Lake City, USA; ^4^Veterans Health Administration, Minneapolis, USA; ^5^Center of Innovation for Veteran-Centered and Value-Driven Care, Seattle, WA, USA; ^6^Center for Care Delivery & Outcomes Research Minneapolis, MN, USA

###### **Correspondence:** Adam Gordon (adam.gordon@hsc.utah.edu)

*Implementation Science 2023,* **18(Suppl 3):**S15

**Background:** The Department of Veterans Affairs’ (VA) Stepped Care for Opioid Use Disorder Train the Trainer Initiative (SCOUTT) intended to facilitate medication treatment for opioid use disorder (MOUD) in primary care (PC), general mental health (MH), and pain (P) clinics. SCOUTT—a operational and research collaborative—began in 2018 and was led by a coordinating center and external facilitators assigned to each facilities’ clinical champions to provide guidance, education, and overcome impediments to implementation. We sought to examine the models of care (MOC) implemented at 37 SCOUTT facilities and barriers and facilitators in implementation.

**Methods:** SCOUTT engaged clinical champions in 18 VA facilities (Phase 1) and these teams, in 2020, helped facilitate spread of MOUD in an additional 19 facilities (Phase 2). In June 2022, we conducted a mixed-methods email survey to clinical champions of 37 facilities to inquire about the primary MOC being implemented within their clinics and the top three barriers and facilitators to implementation. Facility responses were tabulated, structured data were recorded, and unstructured data were assessed and analyzed for themes contrasting frequent barriers and facilitators identified by the Phase 1 and 2 facilities.

**Findings:** Overall, 28 facilities responded (Phase 1: n=15 [83.3%], Phase 2: n=13 [68.4%]) with a total of 63 PC-MH-P clinics at those sites (Phase 1: n=35, Phase 2: n=28). The most common clinic MOC were pharmacy-collaborative-care-model (n=13, 37.1%) in Phase 1 and physician-directed-model (n=16, 57.1%) in Phase 2. The top three barriers to care were provider/staffing (turnover, understaffing), waivered prescribers not prescribing, and lack of incentives to provide care and provider stigma (tie). Phase 1 and 2 sites responded similarly to barriers, but Phase 2 sites reported patient stigma as the primary barrier. Phase 1 top facilitators included provider collaboration (nurses and pharmacists managing patients), clinical/programmatic support (support from clinicians and specialists, staffing), and leadership support. Phase 2 facilitators were similar, however a prominent facilitator in Phase 2 was that clinic prescribers were more accepting of MOUD and training.

**Implications for D&I Research:** The expansion of the national SCOUTT Initiative has demonstrated diverse MOC adoption throughout PC-MH-P clinics. Similar barriers and facilitators to implementation were observed across SCOUTT phases.

**Primary Funding Source**

Department of Veterans Affairs

### S16 Enhancing the implementation of telemedicine for veterans with serious mental illness

#### Pushpa Raja^1^, Sonya Gabrielian^2^, Neal Doran^3^

##### ^1^Greater Los Angeles VA Healthcare System, Los Angeles, CA, USA; ^2^Veterans Health Administration, Los Angeles, CA, USA; ^3^San Diego VA Healthcare System, San Diego, CA, USA

###### **Correspondence:** Pushpa Raja (Pushpa.Raja@va.gov)

*Implementation Science 2023,* **18(Suppl 3):**S16

**Background**

Across health systems, many anticipate that pandemic-driven telehealth adoption will be sustained. However, little is known about the relationship between telemedicine implementation and the quality of care for individuals with serious mental illness (SMI, defined as psychotic and bipolar disorders). Within the Department of Veterans Affairs (VA), we studied the relationship between facility-level telemedicine adoption and performance metrics focused on SMI care. This work aimed to inform potential adaptations to telemedicine services for individuals with SMI to support effective care.

**Methods**

Using national VA administrative data across 138 facilities, from January 2021 – March 2022, we examined facility-level adoption of telemedicine for veterans with SMI and facility-level performance metrics specified for this population. We quantified the percentage of each facility’s SMI outpatient visits delivered via VA Video Connect (VVC). Performance measures were specific to individuals with SMI and addressed: 1. Access to primary care; 2. Continuity of mental health utilization for a subpopulation with high-risk events; 3. Access and continuity of psychotherapy/psychosocial services; and 4. Access and continuity within two intensive SMI specialty programs. We used longitudinal mixed effects regression models to evaluate associations between VVC use and facility performance across these measures.

**Findings**

A higher proportion of SMI care being delivered via VVC was associated with lower scores on measures of continuity of psychotherapy/psychosocial care, access to primary care, and both access and continuity of case management for SMI. There was a positive association between VVC use and proportion of veterans accessing VA’s intensive outpatient program for SMI (all *p*s < .05). VVC use was unrelated to mental health care continuity following a high-risk event, or to the receipt of at least 1 psychosocial intervention visit.

**Implications for D&I Research**

While telemedicine implementation enabled healthcare for many veterans with SMI, facility-level telemedicine adoption may negatively impact continuity of care both within programs specific to SMI and across general mental health settings. Facility-level telemedicine adoption may also negatively impact primary care access among individuals with SMI. Implementation supports (e.g., augmentation with in-person case management, integration of peer supports) may be needed to optimize engagement of this vulnerable population.

### S17 Project mimic (maximizing the implementation of motivational incentives in clinics): a type 3 hybrid trial in 28 opioid treatment programs

#### Sara Becker

##### Northwestern University, Chicago, IL, USA

###### **Correspondence:** Sara Becker (sara_becker@brown.edu)

*Implementation Science 2023,* **18(Suppl 3):**S17

**Background:** CM is one of the most effective adjunctive treatments to medication for opioid use disorders, but its implementation in opioid treatment programs (OTPs) remains low. Project MIMIC is a cluster-randomized, type 3 hybrid effectiveness-implementation trial comparing two strategies to implement CM in the OTP setting. We describe Project MIMIC’s design and share preliminary results from the first 18 OTPs, including 131 staff (counselors/leaders) and 378 patient participants.

**Methods:** Eighteen OTPs were cluster-randomized to receive either the Addiction Technology Transfer Center (ATTC) strategy (workshop + feedback + coaching) or the Enhanced ATTC (E-ATTC) strategy, which layered in two additional strategies: Pay-For-Performance and Implementation Sustainment Facilitation. Consistent with the exploration, preparation, implementation, and sustainment (EPIS) framework, OTPs engaged in 5 months of preparation, 10 months of implementation, and 6 months of sustainment monitoring.

**Findings:** During the preparation phase, 105 counselors (55 E-ATTC, 50 ATTC) enrolled in Project MIMIC, of which 99 (100% EATTC, 94% ATTC) completed the didactic CM workshop and 64 (67% EATTC, 54% ATTC) submitted a role play for performance feedback. During the implementation phase, rates of patient recruitment, providers adopting CM, and providers meeting the CM exposure benchmark all favored E-ATTC relative to ATTC (recruitment: 87% vs 77%; adoption: 60% vs. 44%; exposure: 38% vs. 16%, all *p*-values < .0001). Of the first 18 OTPs, four E-ATTC sites and three ATTC sites sustained CM with fidelity following removal of external support: another four OTPs (1 E-ATTC, 3 ATTC) sustained CM incentives with low fidelity to the model. Another 10 OTPs are currently in the active implementation phase and additional data on these programs will be reported.

**Implications for D&I Research:** Preliminary data indicate that CM training engagement, recruitment, adoption, and exposure rates were greater in the E-ATTC condition, relative to the ATTC condition. Next steps include examining effects on patient outcomes, and refining ongoing fidelity monitoring. These data have informed design decisions for the implementation strategies used in the planned, Medicaid-funded rollout of CM to 200 clinics across California.

**Primary Funding Source**

National Institutes of Health

### S18 Cost-effectiveness of in-person vs. Virtual contingency management implementation strategies

#### Bryan Garner

##### The Ohio State University, Columbus, OH, USA

###### **Correspondence:** Bryan Garner (bryan.garner@osumc.edu)

*Implementation Science 2023,* **18(Suppl 3):**S18

**Background:** Project MIMIC (Maximizing Implementation of Motivational Incentives in Clinics) is an ongoing cluster-randomized hybrid type 3 trial evaluating multi-component strategies to implement contingency management (CM), a behavioral evidence-based practice, across opioid treatment programs (OTPs). Due to the COVID-19 pandemic, we had to rapidly shift the delivery of the training workshop component of the strategy to fully virtual. As a result, counselors in the first cohort received in-person workshop training, whereas counselors from the second cohort received virtual workshop. This unanticipated shift presented a rare opportunity to compare the effectiveness and cost-effectiveness of the two modalities for equipping OTP staff to implement contingency management with fidelity.

**Methods**

Twenty-six counselors from eight OTPs received in-person didactic training, whereas 31 counselors from 10 OTPs received virtual didactic training. Common training elements were the facilitator, learning objectives, and educational strategies/activities. All clinicians submitted a post-training role-play, independently scored with a validated fidelity instrument (Continency Management Competence Scale) for which performances were compared against benchmarks representing initial readiness and advanced proficiency. Cohort-specific rates for benchmark attainment were calculated and per-clinician costs were estimated for the two modalities. Adjusted differences between cohorts were estimated using ordinary least squares, and an incremental cost effectiveness ratio was calculated to specify cost differences across cohorts.

**Findings:** Attainment rates of the readiness and proficiency benchmarks were higher in the virtual than in-person condition (readiness: 86% vs. 96%, proficiency: 36% vs. 41%) though these differences were not statistically significant. Aggregated costs showed a $423 difference in per-clinician cost favoring virtual workshop training. Due to its lower cost and comparable effectiveness, the virtual modality was the dominant strategy.

**Implications for D&I Research:** Our findings support the utility, effectiveness, and cost-effectiveness of virtual workshop training as a means of promoting CM delivery with fidelity. These results may inform the delivery of workshop training as part of a multi-component implementation strategy for CM and other EBPs post-pandemic.

**Primary Funding Source**

National Institutes of Health

### S19 Translating research to practice: advancing the uptake of culturally tailored contingency management in partnership with american indian communities

#### Kait Hirchak

##### Washington State University, Pullman, WA, USA

###### **Correspondence:** Kait Hirchak (katherine.hirchak@wsu.edu)

*Implementation Science 2023,* **18(Suppl 3):**S19

**Background:** We conducted two community engaged randomized controlled trials of a culturally adapted contingency management (CM) intervention for alcohol and illicit drug use among American Indian and Alaska Native (AI/AN) adults residing in rural and urban areas. Across 272 participants, CM was associated with reduced alcohol, stimulant, and cannabis use. Responding to interest from Tribal communities, our research team pivoted to translate lessons learned from these studies to develop a suite of implementation support tools for AI/AN communities.

**Methods:** Tenets of community-based participatory research were applied across partnerships. The Quality Implementation Framework was used to guide the development process. In partnership with the AI/AN communities, four members of the university team developed implementation tools.

**Findings:** We developed a modified CM manual for Indigenous communities to aid intervention delivery and implementation with 8 new Tribal partners across 3 states. Intervention adaptations included integrating cultural values and alignment of CM with Indigenous worldviews; an incentive tracker that assures Medicaid-compliance; and client narratives (i.e., case studies). Implementation support tools included infographics to illustrate CM fidelity, a modified practice facilitation guide (i.e., a clinic asset and readiness assessment), pre- and post- knowledge surveys, and resources for coaching consultations to support ongoing, high-fidelity implementation.

**Implications for D&I Research:** A suite of implementation support tools were identified to quickly meet the interest in CM by AI/AN communities. Results indicate that rapid movement from community-engaged Phase III clinical trials to adoption may be feasible and can strengthen the capacity among AI/AN communities in their efforts to provide culturally and clinically meaningful treatment to Tribal members.

**Primary Funding Source**

National Institutes of Health

### S20 State level implementation of contingency management for stimulant use disorders

#### Sara Parent

##### Washington State University, Pullman, WA, USA

###### **Correspondence:** Sara Parent (sara.parent@wsu.edu)

*Implementation Science 2023,* **18(Suppl 3):**S20

**Background:** Contingency Management (CM) is behavioral evidence-based practice (EBP) for substance use disorder, showing particular promise targeting stimulant use. Nationwide methamphetamine-related morbidity and mortality currently drive widespread interest in CM, creating a need for CM implementation support. The aims of this presentation are to: describe large-scale implementation initiatives in Montana and Washington in terms of the EPIS (exploration, preparation, implementation, sustainment) framework; report preliminary data on common barriers; and discuss lessons learned in translating research designed protocols to real-world settings.

**Methods:** In partnership with Montana and Washington state healthcare authorities and behavioral health training experts, we co-designed and provided implementation support for a CM intervention for stimulant use disorders. The EPIS preparation phase included both outer and inner context activities. Outer context (state-wide) efforts included meeting with contract providers to secure funding, create an infrastructure to standardize the CM program, and provide group-learning environments that facilitated inter-organizational communication. The inner context (site-level) work accomplished through group and 1-on-1 coaching calls identified site-specific characteristics that facilitated or challenged implementation. During the implementation phase, we provided tracking tools to promote program fidelity and adherence to federal anti-kickback regulations, which allowed for remote fidelity monitoring and data collection. Our findings based on these data as well as detailed process notes and verbal feedback from sites will inform how these state systems move onto the sustainment phase.

**Findings:** To date, 33 clinical sites have completed our didactic CM training; a total of 44 coaching calls have been conducted. 13 sites have implemented the intervention, as measured by at least one CM visit with an eligible patient. Implementation challenges identified included some common to other EBP implementation efforts (e.g. high staff turn-over, protocol fidelity, incorporating into existing structures/workflow), as well as CM-specific barriers (e.g. federal anti-kickback regulations, use of point of care urine testing).

**Implications for D&I Research:** Lessons learned and data collected from our CM implementation initiative yield valuable insight for future largescale CM initiatives, including that underway in California for up to 200 clinics. By identifying solutions to common implementation barriers, we hope to advance the uptake of this life-saving EBP.

**Primary Funding Source**

State block grants

## Building the Future of D&I Science: Training, Infrastructure, and Emerging Research Areas

### S21 The implementation planning assessment tool: a tool to encourage researchers and trialists to be more proactive, nimble and responsive to implementation science needs

#### Christine Kowalski^2^, Linda Kawentel^2^, Tassos Kyriakides^3^, Lori Davis^4^, Nicholas Bowersox^2^, Amy Kilbourne^5^, Grant Huang^6^, Andrea Nevedal^1,5^

##### ^1^VA Ann Arbor Healthcare System, Ann Arbor, MI, USA; ^2^VA QUERI Center for Evaluation and Implementation Resources (CEIR), Ann Arbor, MI, USA; ^3^Veterans Health Administration, West Haven, CT, USA; ^4^Tuscaloosa VA Medical Center, Tuscaloosa, AL, USA; ^5^VA Center for Clinical Management Research Ann Arbor, MI, USA; ^6^VHA Office of Research & Development Washington, D.C., USA

###### **Correspondence:** Christine Kowalski (Christine.Kowalski@va.gov)

*Implementation Science 2023,* **18(Suppl 3):**S21

**Background**

Without a proactive plan to implement clinical trial findings, it can take decades for one-fifth of effective interventions to be adopted into routine care settings. There is a dearth of pragmatic tools to prepare trialists for implementing effective treatments or programs across the translation spectrum. To address knowledge gaps, we introduce a newly developed Implementation Planning Assessment (IPA) Tool that any trialist or researcher may use throughout initial studies to support future trials or research to understand implementation of effective interventions.

**Methods**

The tool was developed through a systematic process by an interdisciplinary team with expertise in implementation science, clinical trials, program evaluation, and qualitative methods; team meetings with an organized set of agendas were used to develop and refine the tool.

**Findings**

The tool emphasizes three phases to accelerate the adoption of interventions into routine clinical care: Phase 1, “Planning, Framing, and Aligning Interested Parties,” involves identification and garnering of input from multilevel interested parties who have a vested interest in the trial’s results and potentially the leverage to incorporate results or effective treatments into routine practice via organizational changes. Phase 2, “Implementation Process Data Collection,” involves planning and assessment by clinical and research leaders that will promote uptake of the intervention, if found effective, and the enactment of an implementation plan. Phase 3 “Planning for Sustainment for Effective Trials,” takes results from phases 1-2 to outline a process by which trial results and interventions will be adopted in routine practice. Throughout all phases, the assessment team should also plan for dissemination, which involves sharing information about the intervention, implementation, and trial results to increase uptake among key interested parties.

**Implications for D&I Research**

This tool, anchored in implementation science principles, provides a much-needed, practical guide for trialists and researchers working across the translation spectrum to spread effective interventions that would improve the healthcare of patients. This tool brings a ready-made list of necessary steps for trialists and researchers aiming to improve implementation, including scale-up and spread, of effective, clinical-trial-tested interventions in health care settings. It can also be utilized by clinicians and health services researchers who are new to the field of implementation science.

### S22 Learning from missed opportunities through retrospective application of the implementation planning assessment (ipa) tool in a va clinical trial

#### Lori Davis^1^, Tassos Kyriakides^2^

##### ^1^Tuscaloosa VA Medical Center, Tuscaloosa, AL, USA; ^2^Veterans Health Administration, West Haven, CT, USA

###### **Correspondence:** Lori Davis (lori.davis@va.gov)

*Implementation Science 2023,* **18(Suppl 3):**S22

**Background**

The Veterans Individual Placement and Support Toward Advancing Recovery (VIP-STAR; CSP#589) was a VA Cooperative Studies Program (CSP) multicenter, randomized clinical trial that compared the effectiveness of Individual Placement and Support (IPS) supported employment vs. usual care (transitional work program) in terms of securing and maintaining competitive employment among unemployed Veterans with a diagnosis of post-traumatic stress disorder (PTSD).

**Methods**

The study operations were built on a collaborative partnership between the clinical and research team(s) at each of the twelve participating sites. While the CSP trial process/protocol did not include any implementation expertise per se, the study IPS trainers, fidelity monitors, program evaluators, PTSD clinicians, and vocational rehabilitation experts successfully executed the trial with essential implementation components. Even though the IPA tool was not available at the time of the trial launch, it was retrospectively applied to assess its utility.

**Findings**

The VIP-STAR study concluded that more Veterans in the IPS group became steady workers and earned more income from competitive jobs over the 18-month follow-up compared with the usual care group. Implementation of findings at several local VAMCs was assisted by the pre-existing partnership and the study operations at large. However, despite its strong evidence base, implementation of IPS for PTSD in VA has not been widespread, which is possibly due to our lack implementation specialists working at the front end with program office leadership to develop a roadmap for future sustainment of the most effective services. The application of the IPA tool to this trial provides a retrospective illustration of its components and clearly shows the utility of this tool and highlights the need to consider implementation issues a priori.

**Implications for D&I Research**

The IPA Tool would have allowed the VIP-STAR team to assess stakeholder concerns and solve challenges within the VA that would impact efficient and timely implementation of the trial results. Even though no benchmarks of successful implementation were drafted during the trial, the retrospective application of the developed IPA tool strongly points to utilization of this tool in trials going forward.

**Primary Funding Source**

VA Cooperative Studies Program

### S23 Proactive application of the implementation planning assessment tool in a multi-site va clinical trial

#### Whitney Mills^1,2^, Kali Thomas^2^, Yuan Huang^3^, Michael Wininger^3^, Scott Hummel^4^

##### ^1^Brown University, Providence, RI, USA; ^2^VA Providence Healthcare System, Providence, RI, USA; ^3^CSP Coordinating Center (WH-CSPCC), VA CT Healthcare System ,West Haven, CT, USA; ^4^VA Ann Arbor Healthcare System, Ann Arbor, MI, USA

###### **Correspondence:** Whitney Mills (whitney.mills@va.gov)

*Implementation Science 2023,* **18(Suppl 3):**S23

**Background**

The VA Cooperative Studies Program (CSP) study “Geriatric OUt-of-hospital Randomized MEal Trial in heart failure – Veterans Affairs” (GOURMET-VA; CSP #2025) is a randomized, single-blind, multi-center, clinical trial investigating the effects of home-delivered meals and enhanced dietary education in Veterans discharged from hospitalization for heart failure. GOURMET-VA is one of the first CSP studies to integrate implementation scientists during protocol planning. This presentation describes how the Implementation Planning Assessment (IPA) Tool was used proactively to guide planning and integration of an implementation evaluation in the trial protocol.

**Methods**

The three IPA phases guided development of an implementation evaluation oriented towards future adoption and sustainment of this intervention into routine clinical practice.

**Findings**

Phase 1 (“Planning, Framing, & Aligning Interested Parties”): During early protocol development, the implementation team helped identify and facilitated meetings with relevant VA national leadership including the Office of Geriatrics and Extended Care, Office of Nutrition and Food Services, and Office of Nursing Services. These meetings resulted in letters of support outlining engagement throughout the trial. Phase 2 (“Implementation Process Data Collection”): Guided by IPA, the implementation evaluation focuses on understanding the context into which the intervention is being implemented, the experiences of Veterans and clinicians with the intervention, and identifying barriers and facilitators to implementation at the patient, healthcare provider, and leadership levels. The evaluation includes mixed methods analysis of data collected as part of the trial and through prospective qualitative interviews. Phase 3 (“Planning for Sustainment of Effective Trials”): A preliminary dissemination and sustainability plan was drafted, guided by the conversations with our VA leadership partners. As the trial results are not yet known, we also drafted a plan for how the implementation evaluation data may be used to inform adaptations of the GOURMET-VA intervention to improve effectiveness and uptake.

**Implications for D&I Research**

The IPA provides structured guidance for integrating dissemination and sustainment into trial protocols. Furthermore, the structured guides that comprise the IPA allowed the implementation scientists and the trial team to find common ground in terms of rationale, methods, and language. The IPA is valuable for both new and experienced implementation scientists.

**Primary Funding Source**

VHA Cooperative Studies Program

### S24 Vha efforts to develop organizational structured to support consistent implementation evaluation capacity within clinical trials

#### Tassos Kyriakides^1^, Nicholas Bowersox^2^, Grant Huang^3^

##### ^1^Veterans Health Administration, West Haven, CT, USA; ^2^VA QUERI Center for Evaluation and Implementation Resources (CEIR), Ann Arbor, MI, USA; ^3^VHA Office of Research & Development, Office of Research and Development Washington, D.C., USA

###### **Correspondence:** Tassos Kyriakides (tassos.kyriakides@va.gov)

*Implementation Science 2023,* **18(Suppl 3):**S24

**Background**

High Reliability Organization (HRO) and Learning Healthcare System (LHS) integration within large healthcare organizations such as the Veterans Affairs (VA) requires significant reorganization of resources, methods, and workflows. HRO and LHS concepts are essential to effective patient care by ensuring that research identifies cutting-edge treatments which can be effectively utilized within standard healthcare settings and providers can rapidly incorporate these treatments into regular clinical practice. Within VA, one major movement to support HRO and LHS has focused on the incorporation of implementation planning within large-scale clinical trials, supporting fluid and realistic efforts to support practice adoption in the post-trial period. While initial VA efforts in this area have focused on trial-by-trial efforts, efforts have also focused on the creation of standardized internal clinical trial assessment capacities for all new VA clinical trials.

**Methods**

A workgroup of VA implementation scientists and VA Cooperative Studies Program (CSP) leadership reviewed lessons learned from trial-specific efforts to incorporate implementation-relevant factor assessment within CSP trials. These lessons were then used to develop best-practice recommendations to support the development of internal CSP staffing, processes, and workflows which would allow for the collection of data related to practice implementation as a standard aspect of all future VA large-scale clinical trials.

**Findings**

The development of systemic capacities related to implementation assessment within large-scale clinical trials includes considerations funding, supervision and oversight, recruitment and training of staff with specialized research skills, and the development of new workflows to support effective collaboration between new and existing CSP staff. Additional considerations focused on both the final desired “new normal” to be achieved as well as interim approaches that would allow CSP to incorporate implementation data collection while also building sustainable long-term capacity.

**Implications for D&I Research**

The standardization of large-scale assessment of implementation factors within large-scale clinical trials will improve the direct applicability of clinical trial results to patient care. This process will also open up opportunities for implementation researchers to help inform the identification of clinical breakthroughs and highlights the essential role of implementation science in supporting HRO and LHS within large integrated healthcare systems such as VA.

**Primary Funding Source**

VHA Cooperative Studies Program

### S25 Designing for dissemination of treatments for covid-19: the fit to context framework and an engagement methods navigator tool

#### Bethany Kwan^1^, Matt DeCamp^1^, Chelsea Sobczak^1^, Jenna Reno^2^, Adit Ginde^1^, Hillary Lum^1^

##### ^1^University of Colorado Anschutz Medical Campus, Aurora, CO, USA; ^2^RTI International, Denver, CO, USA

###### **Correspondence:** Bethany Kwan (Bethany.kwan@cuanschutz.edu)

*Implementation Science 2023,* **18(Suppl 3):**S25

**Background:** Designing for dissemination and sustainability (D4DS) includes use of participatory engagement methods and application of D&I theories to guide the process of ensuring innovation “fit to context” and planning for dissemination. We used capacity building resources and tools to design, enact, and evaluate dissemination strategies for evidence-based monoclonal antibody (mAb) treatments for COVID-19 outpatients. Intravenous mAb treatment was available by December 2020 but uptake was slow and inequitable.

**Methods:** The Fit to Context (F2C) Framework for D4DS is a process framework with four phases – conceptualization, design, dissemination, and impact – used to guide Colorado mAb dissemination strategies. Diffusion of innovation theory underpinned mixed methods assessment of contextual factors related to mAb use, a key aspect of *conceptualization*. The DICEMethods.org stakeholder engagement navigator tool yielded methods for engaging partners and communities in *design* of dissemination messages and products. Conceptualization and design phase insights informed *dissemination* channels. Real-world data from a statewide referral system showed *impact* on mAb referral patterns.

**Findings:** Community and clinician surveys, focus groups, and interviews revealed the need to simplify messaging and centralize mAb treatment systems and processes. A multisector stakeholder advisory panel and community engagement studios oriented dissemination planning to COVID-19 geographic, racial/ethnic, and socioeconomic disparities. Dissemination products are at mAbColorado.org. Communication channels included social media, radio, and television, webinars, and distribution by “regional health connectors.” Over 10 months, total mAb referrals increased by 539% from 369/month (March-July 2021) to 1989/month (August-December 2021), and unique referring clinicians increased by 483% from 159/month (March-July 2021) to 769/month (August – December 2021) following the July launch of the mAb Colorado dissemination campaign. Treatment sites increased from 18 to 58, many of which were in rural and underserved communities.

**Implications for D&I Research:** D4DS involves a complex set of activities to assess context, build partnerships, co-design dissemination products, leverage and build system capacity for distribution, and evaluate impact. Use of the Fit to Context Framework can systematize rapid conceptualization, design, dissemination, and evaluation of strategies for promoting adoption of evidence-based health care innovations during pandemics and beyond. D&I tools such as DICEMethods.org complement use of D4DS process and context frameworks.

**Primary Funding Source**

National Institutes of Health

### S26 Development of a generalizable designing for dissemination and sustainability tool for implementation scientists and practitioners

#### Maura Kepper^1,2^, Bethany Kwan^3^, Russell Glasgow^4^, Douglas Luke^5^, Andrea Graham^6^, Ana Baumann^2^, Thembekile Shato^2^, Brad Morse^3^, Ross Brownson^7^

##### ^1^National Cancer Institute’s Consortium for Cancer Implementation Science, USA; ^2^Washington University in St. Louis, St. Louis, MO, USA; ^3^University of Colorado Anschutz Medical Campus, Aurora, CO, USA; ^4^University of Colorado School of Medicine, Aurora, CO, USA; ^5^Center for Public Health Systems Science, Washington University in St. Louis, Saint Louis, MO, USA; ^6^Northwestern University Feinberg School of Medicine, Chicago, IL, USA; ^7^Washington University in St. Louis, Brown School, St. Louis, MO, USA

###### **Correspondence:** Maura Kepper (kepperm@wustl.edu)

*Implementation Science 2023,* **18(Suppl 3):**S26

**Background:** The use of Designing for Dissemination and Sustainability (D4DS) principles ensures that the products of research (interventions, tools, materials, findings) are developed to match well with the needs and context of the target problem, audience and setting. Thereby, dissemination efforts can be well-received and implementation and sustainability can be best supported to increase impact. However, D4DS principles are often not used during the various stages of research.

**Methods:** We are using a user-centered design (UCD) process to develop a digital tool that will facilitate the use D4DS with particular emphasis on the first two phases (conceptualization and design) of the Fit to Context Framework (see abstract 1). Our UCD design process: 1) identifies the need and demand for key issues this tool should address through focus groups and an environmental scan; 2) builds a tool using an iterative design process; and 3) tests the tool for usability. The iterative design process allows end-users to give feedback on design options (e.g., wireframes) in successive versions. Final designs will be built into a digital tool by professional developers and tested for usability.

**Findings:** End-users were defined as implementation science teams conducting a health-related project, which may include academics (e.g., researchers, students), practitioners (e.g., clinicians, health departments) and non-governmental organizations. An environmental scan confirmed that no such tool exists and gathered D4DS methods and resources that will be delivered via the tool. Focus groups confirmed a need for and excitement about this tool. Practitioners particularly felt a tool that walked them through key steps in design and planning would be useful for community-based research projects. Beyond use in their own work, end-users also wanted to use this tool for teaching and consulting. Users wanted the ability to save their work and work interactively with a team throughout design, which facilitates academic-community partnership. We will present the prototype tool.

**Implications for D&I Research:** The D4DS tool will provoke users to think critically and engage stakeholders to develop a plan for active and equitable dissemination and sustainability—ultimately resulting in more effective translation of research to practice and policy.

### S27 Building dissemination capacity through the colorado clinical and translational sciences institute dissemination consultation service

#### Heather Gilmartin^1^, Justin Shrader^2^, Robert Thompson^2^, Demetria McNeal^3^, Bethany Kwan^2^

##### ^1^Denver/Seattle Center of Innovation, Rocky Mountain Regional VA Medical Center, Aurora, CO, USA; ^2^University of Colorado Anschutz Medical Campus, Aurora, CO, USA; ^3^University of Colorado School of Medicine, Denver, CO, USA

###### **Correspondence:** Heather Gilmartin (heather.gilmartin@va.gov)

*Implementation Science 2023,* **18(Suppl 3):**S27

**Background:** Scientific dissemination is an active, tailored process of communicating complex research or technical information to a wide audience. Dissemination efforts should be guided by theories or frameworks and the information should be made simple and engaging to convey key messages to non-scientific audiences. Reasons to actively disseminate science beyond posters, presentations, and papers include to connect to those who may benefit from research findings sooner than the 17-year average, to raise the profile of your work and organization, and to engage diverse partners in current and future work. Most scientists are not trained in dissemination, limiting their awareness of best practices, tools, and resources. To build dissemination capacity and provide training, mentorship, and guidance to members of the Colorado Clinical and Translational Sciences Institute (CCTSI), a free dissemination consultation service was launched within the Dissemination and Implementation (D&I) Research Core. In this panel discussion we will describe the creation of the consultation service and review and discuss the most common consultation topics, tips, and best practices.

**Methods:** The dissemination consultation service launched in 2021 and is staffed by six, part-time faculty with expertise in the fields of health communication, D&I, health services, nursing, social psychology, and broadcast media. Virtual consults occur on Zoom. Consultation discussions and evaluations are documented, stored in a secure database, and descriptively analyzed in Excel.

**Findings:** Over 18 months, 37 consultations with basic, clinical, and public health scientists and practitioners have been provided. The most common topics include dissemination planning guidance, developing an online presence, engaging in online communities, and message creation. Best practice guidance includes use of D&I frameworks, introduction to online profile sites and social media platforms, messaging tips and creation of visual abstracts, infographics, and press releases. Participants have been “extremely satisfied” and report broad application of dissemination best practices within their programs of research.

**Implications for D&I Research:** The evidence-based guidance provided through the CCTSI dissemination consult service has increased awareness, application, and capacity for dissemination science with researchers across the clinical and translational spectrum. This model could be scaled across academic settings to increase the impact of D&I science.

**Primary Funding Source**

National Institutes of Health

### S28 Innovation corps™ (i-corps™) at the colorado clinical translational sciences institute: fostering innovation across an academic medical campus

#### Demetria McNeal^1^, Cathy Bodine^2^

##### ^1^University of Colorado School of Medicine, Denver, CO, USA; ^2^University of Colorado, Denver, CO, USA

###### **Correspondence:** Demetria McNeal (demetria.mcneal@cuanschutz.edu)

*Implementation Science 2023,* **18(Suppl 3):**S28

**Background:** A key barrier to translation of biomedical research discoveries is a lack of understanding among scientists regarding the complexity and process of implementation. Academic Medical Centers (AMC) that have teaching, patient care, research, and service engrained in their mission are well poised to translate these discoveries to real-world clinical and community practice. NIH adapted the NSF I-Corps™ entrepreneurial training program for life-science researchers to help bridge the so called “valley of death”– the schism between research development and market application. The objectives of the I-Corps program are to a) develop the workforce by catalyzing an academic entrepreneurial culture and skillset; b) develop discoveries and commercialization potential, and, c) demonstrate impact by connecting researchers to resources for commercialization, domain expertise, and accelerator funding. Colorado has a vibrant local startup ecosystem, state support for commercialization and entrepreneurship as well as critical mass of product development expertise. University of Colorado Anschutz Medical Campus, as a major AMC, is an engine for growth for the region.

**Methods:** This team-based experiential three-week training is taught by faculty with business and entrepreneurial experience, many having prior industry experience. From 2016 to spring of 2022, I-Corps@CCTSI has trained over 10 cohorts, more than 70 teams and greater than 200 participants from diverse backgrounds. The customer discovery interview process has resulted in more than 1700 interviews to date. Content includes customer discovery, value proposition, and validating needs.

**Findings:** Innovations related to medical devices (33%), drugs/biologics (20%), software applications (16%), and diagnostics (8%) have completed to the program. An average of 24 interviews was conducted Teams reported increased readiness for commercialization over time (83%, 9 months; 14%, 3 months). Thirty-nine percent met with institutional technology transfer to pursue licensing/patents and 24% pursued venture capital/investor funding following the short courses.

**Implications for D&I Research:** I-Corps training provided at the University of Colorado AMC provide teams with a rigorous and repeatable process to aid development of a business model based on customer needs.

**Primary Funding Source**

National Institutes of Health

### S29 Monitoring for unintended consequences of ehr-based implementation strategies: a novel approach using ehr audit logs and machine learning

#### Jinying Chen^1,2^, Sarah L. Cutrona^3^, Ajay Dharod^4^, Adam Moses^2^, Aaron Bridges^2^, Brian Ostasiewski^2^, Kristie L. Foley^4^, Thoms K. Houston^2^

##### ^1^UMass Chan Medical School, Worcester, MA, USA; ^2^Wake Forest School of Medicine, Winston-Salem, NC, USA; ^3^University of Massachusetts Medical School, Worcester, MA, USA; ^4^Wake Forest University School of Medicine, Winston-Salem, NC, USA

###### **Correspondence:** Jinying Chen (jinchen@wakehealth.edu)

*Implementation Science 2023,* **18(Suppl 3):**S29

**Background:** Clinical decision support (CDS) tools in electronic health records (EHRs) are used to support quality improvement. Monitoring for both intended and unintended consequences of CDS on clinic workflow is important. We report a novel monitoring approach using EHR audit logs and machine learning.

**Methods:** We compared distributions of cancer providers’ EHR activities in 4 clinics from 2019-2020 related to CDS for tobacco cessation. Monitoring was conducted within a 4.5-hour window of patient visits before (2,633 visits) and after (1,070 visits) implementing the CDS alert. We used topic modeling, a latent-variable statistical machine learning approach, to analyze EHR audit logs recording low-level events (e.g., loading a ‘visit navigator’ template, lab review, completing alerts). A topic model, trained using EHR log segments, identified 12 topics, with each assigned a clinical activity by consulting 4 domain experts. We then applied the topic model to EHR logs for during-Visit and before-Visit sessions to obtain the time a provider spent on each clinical activity for each session (estimated from topic distribution and session length). Mean time spent on each activity pre- and post-alert-implementation was estimated by using the inverse-probability-weighted regression adjustment method to adjust for imbalance between pre- and post-alert-implementation data. The study was IRB-approved.

**Findings:** Within the 12 clinical activities, 3 were CDS-related (Topic/T2: reviewing patient records and addressing alerts, T8: acting on CDS, T10: bypassing/postponing CDS), 3 focused on reviewing information (T5: vital signs, T6: snapshot of patient reports, T12: outside organization records), 5 involved modifying EHR (T1: documenting patient visit, T3: modifying diagnosis and problem lists, T4: reviewing and writing clinical notes, T7: using Visit Navigator to manage clinical care and bill, T11: placing and documenting orders), and one (T9) was searching patient chart. Providers spent more time addressing CDS (T2, 8, and 10; 32-35 more seconds) during-Visit post alert-implementation (vs. pre-implementation). We found compensatory unintended reductions in time spent reviewing patient vital data (T5; 61 seconds less) and modifying EHR (T1, 3, and 11; 7-24 seconds less). A smaller magnitude in changes was seen before-Visit.

**Implications for D&I Research:** Our novel method can monitor impact of CDS-based implementation strategies, including unintended consequences hard to document and measure.

**Primary Funding Source**

National Institutes of Health

### S30 Community-engaged implementation science: key lessons from 'ending the hiv epidemic’ supplement awards

#### Laura Beres^1^, Jessica Corcoran^2^, Amy Corneli^3^, Audrey Harkness^4^, Phillip Marotta^5^, Jonathan Ross^6^, Gabriela Betancourt^6^, Pedro Serrano^7^, Sheree Schwartz^8^, Stefan Baral^9^, Christopher Kemp^10^, Christopher Hoffmann^9^, Katherine Rucinski^9^, Debbie Humphries^11^

##### ^1^Johns Hopkins Bloomberg School of Public Health, Baltimore, MD, USA; ^2^University of Alabama at Birmingham School of Nursing, Birmingham, AL, USA; ^3^Duke University, Durham, NC, USA; ^4^University of Miami, Miami, FL, USA; ^5^Veterans Health Administration, St. Louis, USA; ^6^Latino Commission on Aids, New York, USA; ^7^Independent Consultant, Chicago, USA; ^8^Johns Hopkins University, Washington, DC, USA; ^9^Johns Hopkins University, Baltimore, MD, USA; ^10^University of Washington, Seattle, WA, USA; ^11^Yale School of Public Health, New Haven, CT, USA

###### **Correspondence:** Laura Beres (laura.beres@jhu.edu)

*Implementation Science 2023,* **18(Suppl 3):**S30

**Background:** High quality, community-engaged research (CER) is fundamental to advancing effective, equitable implementation of evidence-based interventions. However, authentic community engagement is challenging within academic settings. The Ending the HIV Epidemic (EHE) supplement awards funded community-engaged HIV implementation science (IS). We offer lessons from EHE-supported research to strengthen CER within IS.

**Methods:** Among 2021-2022 EHE awardees, seven academic-community research partnerships emphasized community-based outreach strategies within CER. Representatives from six partnerships engaged in three semi-structured reflection sessions and documented experiences in an online form. The reflections highlighted the academic perspective, as representatives were all university-based researchers, except for one county health system-employee. Key opportunities and challenges were identified through iterative dialogue and qualitative analysis of documentation.

**Findings:** Most awardees were engaged in new partnerships developed for the supplement application, with two ongoing for >1 year. Partnership typologies spanned ‘involvement’ to ‘shared leadership’^1^ with engagement activities including community advisory boards, collaborative protocol and implementation plan writing, and community-led recruitment. Partnership outcomes included immediate improved implementation plans and measurement and additional concepts to guide future research. Thematic analysis revealed multiple barriers to effective CER: 1) Requirements for IRB approval prior to issuance of formal funding agreements limited opportunities for meaningful community partner input; 2) Delayed payments to community partners due to contingencies (e.g., CFAR renewal) and slow university administrative subcontracting undermined equitable collaboration; 3) Burdensome university systems consumed time and effort of community partners; and 4) Lack of widely-recognized scholarship products from relationship building limits the ability of researchers to invest in effective partnership development. Opportunities include investigators focusing short award periods on exploratory aims that lay partnership foundations respecting competing partner priorities, collaboratively identify appropriate implementation strategies, and approach implementation logic models iteratively and collaboratively.

**Implications for D&I Research:** We identified system re-design opportunities to foster impactful CE-IS. These may include longer funding horizons with time and financial support for building relationships; encouraging community partner co-investigators on applications; flexible grant management that can leverage university resources to facilitate funded, equal participation throughout the full work period; fast-tracked community partner subcontracts; practice-to-research partnership development small grants; and academic recognition of long-term benefits of investing in relationships.

**Primary Funding Source**

National Institutes of Health

**Reference**

1. Yuen T. 2015 doi: 10.2105/AJPH.2015.302811

### S31 Assessing the purveyor-strategy fit of strategies to support the integration of evidence-based substance use disorder interventions within hiv service settings: results of a national stakeholder-engaged real-time delphi

#### Sheila Patel^1^, Heather Gotham^2^, Thomas Donohoe^3^, Hannah Knudsen^4^, Stephen Tueller^1^, Michael Bradshaw^1^, Sarah McDaniel^1^, Bryan Garner^5^

##### ^1^RTI International, Research Triangle Park, NC, USA; ^2^Stanford University, Palo Alto, CA, USA; ^3^UCLA, Los Angeles, CA, USA; ^4^University of Kentucky, Lexington, KY, USA; ^5^The Ohio State University, Columbus, OH, USA

###### **Correspondence:** Sheila Patel (svpatel@rti.org)

*Implementation Science 2023,* **18(Suppl 3):**S31

**Background:** To help end the HIV epidemic there is a need to improve integration of substance use disorder (SUD) services within HIV service organizations (HSOs). The Substance Treatment Strategies for HIV Care (STS4HIV Project) sought to understand the fit of three exploration phase, four preparation phase, and three implementation phase strategies the AIDS Education and Training Centers (AETC) purveyor network may use to help address this need.

**Methods:** Sixty-four AETCs (70% response rate) participated in a stakeholder-engaged Real-Time Delphi (SE-RTD) to assess the purveyor-strategy fit of 10 discrete strategies (i.e., that the strategy is (1) important and (2) feasible to offer, that they are (3) ready to offer it, that it can be offered at (4) scale, and that they face (5) pressure to offer it). The SE-RTD was completed over a two-week period, which involved learning about the strategies, rating them on the above five dimensions, explaining their initial responses, reviewing others’ responses and comments, and changing their final responses if inclined. The purveyor-strategy fit (PSF) score was computed for each strategy by summing the dimension scores.

**Findings:** Nearly all AETCs (97%) perceive a need to help HSOs integrate SUD services, yet only 36% have begun to or routinely support HSOs to do it. Disseminating information about an evidence-based intervention was the only strategy with a fit score (7.92 out of 15) significantly different from all the other strategies; it had the highest score in each of the five dimensions of our fit index. Conducting a formal assessment, developing an implementation plan, providing access to asynchronous training, and conducting synchronous training all scored relatively high in importance and feasibility, but respondents did not believe their AETCs were ready to offer these strategies. Only disseminating information about an evidence-based intervention and providing access to asynchronous training were considered somewhat scalable. Overall, AETCs reported facing little pressure to offer these strategies.

**Implications for D&I Research:** The AETC purveyor network reported that several strategies for improving SUD integration within HSOs were important and feasible, yet the AETCs themselves may need additional support to get ready to offer them and offer them at scale.

**Primary Funding Source**

National Institutes of Health

### S32 Building better healthcare systems through defining implementation facilitation key steps

#### Katherine Dollar^1^, JoAnn Kirchner^2^, Jeff Smith^3^, Mona Ritchie^4^, Eva Woodward^3^

##### ^1^ Center for Integrated Healthcare, Syracuse, NY, USA; ^2^Central Arkansas Veterans Healthcare, North Little Rock, AR, USA; ^3^North Little Rock, AR, USA; ^4^VA Behavioral Health Quality Enhancement Research Initiative (QUERI), Central Arkansas Veterans Healthcare System, North Little Rock, AR, USA

###### **Correspondence:** Katherine Dollar (katherine.dollar@va.gov)

*Implementation Science 2023,* **18(Suppl 3):**S32

**Background**

Implementation Facilitation (IF), an evidence-based implementation strategy, has been applied in multiple contexts to implement innovations with varying complexity. Although IF has become a widely used implementation strategy, it is complex and difficult to describe, and the core components can be challenging to define and apply with fidelity. Further, inconsistent use of terms and operational definitions describing the process of IF are challenges within the IF literature. To build better healthcare systems and enhance implementation science through IF, comprehensive and precise resources that clarify the “key steps” involved in applying IF strategies are needed.

**Methods**

To identify key steps in the IF process, the authors engaged facilitation practitioners, researchers, and trainees from clinical (e.g., primary care, mental health, other) and community settings in a multi-stage expert panel and consensus development process.

First, one author drafted the steps and incorporated feedback from other authors. Second, we piloted a description of the steps with IF training participants to obtain feedback on their usefulness for understanding IF. Third, we recruited an expert panel of 9 IF practitioners, researchers, and regional and national clinical leaders from our IF Learning Collaborative (IFLC), conducted 2 rounds of review, and incorporated the panel’s feedback. Next, we reviewed and made further revisions to the steps, presented them to the full IFLC, and incorporated their additional suggestions. Lastly, the expert panel reviewed and approved the final version of the IF Key Steps document.

**Findings**

The resulting “Key Steps in Implementation Facilitation” document is framed within two principles: ensuring the ongoing presence of the facilitator across all phases of implementation and providing a “safe” facilitator environment. The document includes 8 steps, each with brief guidance for their application along with links to additional resources provided within a comprehensive, publicly available IF Training Manual.

**Implications for D&I Research**

The “Key Steps” document provides enhanced clarity about the process of IF. Through enhanced definitional clarity, it offers greater precision for novice facilitators, operational partners, and investigators seeking to describe implementation facilitation strategies within the literature. Thereby, better defining the core features of IF within D&I research literature.

**Primary Funding Source**

Department of Veterans Affairs

### S33 Identifying core activities for assessing fidelity of implementation facilitation strategies

#### Jeff Smith

##### Behavioral Health QUERI ,North Little Rock, AR, USA

###### **Correspondence:** Jeff Smith (Jeffrey.Smith6@va.gov)

*Implementation Science 2023,* **18(Suppl 3):**S33

**Background**

To transfer successful implementation strategies from research to practice, it is important to be able to measure and support fidelity to an implementation strategy’s core components. Unfortunately, this aspect of implementation science has been underdeveloped and infrequently applied. Implementation facilitation (IF) is an interactive process of problem-solving and support to assist stakeholders in their efforts to adopt clinical innovations into routine practice that occurs in the context of a recognized need for improvement and supportive interpersonal relationships. Our objective was to identify core activities for IF that may be used to assess fidelity to the strategy.

**Methods**

First, we conducted a scoping literature review to identify the range of activities applied in IF strategies. PubMed, CINAHL, and Thompson Scientific Web of Science databases were searched for English-language articles that included the term “facilitation” or other commonly used terms for the strategy published from January 1996 – December 2015. Initially, 1,471 citations/abstracts were identified and screened for relevance by two expert reviewers. Ultimately, 135 articles (from 94 unique studies) were identified for data abstraction on IF activities, frequency with which IF activities were identified as ‘core’ by study authors, and study outcomes. Next, we engaged an expert panel in a rigorous 3-stage modified Delphi consensus development process to identify core IF activities for examples of high and low complexity clinical innovations across three implementation phases (Pre-implementation, Implementation, and Sustainment phases).

**Findings**

Based on the scoping review, 32 distinct IF activities were identified and definitions/examples were developed for each. The expert panel process identified 8 core activities for the Pre-Implementation Phase, 8 core activities for the Implementation Phase, and 4 core activities for the Sustainment Phase. Prototype tools for assessing IF fidelity based on the core activities through quantitative and qualitative measures are currently being piloted (early results will be reported).

**Implications for D&I Research**

Core IF activities were identified based on a comprehensive scoping literature review followed by a rigorous, multi-stage expert panel process. This work to develop tools to help ensure fidelity to core activities is foundational for supporting the effective transfer of successful IF strategies from research to practice.

**Primary Funding Source**

Department of Veterans Affairs

### S34 Enhancing reach and efficiency of implementation facilitation training through novel educational delivery approaches

#### Michele A. Crisafulli^1^, Jessica N. Martin^2^, Christina B. Shook^3^, Jeffery A. Pitcock^4^, Katherine M. Dollar^1^, JoAnn E. Kirchner^5^, Jeffrey L. Smith^6^, Eva N. Woodward^7^

##### ^1^Center for Integrated Healthcare Syracuse, NY, USA; ^2^VA Center for Integrated Healthcare, Syracuse, NY, USA; ^3^Veterans Health Administration, Lebanon, PA, USA; ^4^Veterans Health Administration, North Little Rock, AR, USA; ^5^University of Arkansas for Medical Sciences, Little Rock, AR, USA; ^6^VA Behavioral Health Quality Enhancement Research Initiative (QUERI)Central Arkansas Veterans Healthcare System, North Little Rock, AR, USA; ^7^University of Arkansas for Medical Sciences, Little Rock, AR, USA

###### **Correspondence:** Michele A. Crisafulli (Michele.Crisafulli@va.gov)

*Implementation Science 2023,* **18(Suppl 3):**S34

**Background**

As Implementation Facilitation (IF) is increasingly recognized as evidence-based and effective, demand for expert facilitators grows exponentially. Supporting scale-up and spread of high-fidelity IF since 2011, our Veterans Health Administration (VHA) grant-funded learning hub trains facilitators in complex IF skills, continually evaluating and adapting training to meet learners’ needs. Within VHA and more broadly, demand for IF training now outpaces availability, requiring programs to creatively leverage resources to maximize training efficiency and reach. Here we report on three proactive and responsive adaptations: 1) shifting from in-person to virtual training, 2) selective application of a Virtual Flipped Classroom (VFC), and 3) development of independent learning opportunities.

**Methods**

In 2017, we began administering electronic surveys of learners’ IF knowledge and confidence in skills 2 weeks *before,* and 2 weeks and 6 months *after,* training. Beginning in 2018, training incorporated virtual participants with a hybrid virtual/in-person platform. In 2020, COVID-19 resulted in a pivot to 100% virtual training. In 2022, in consultation with an adult education expert, we decided to apply a VFC to specific portions of the training (learners independently review certain content, then deepen engagement through virtual in-class activities). After reviewing literature for best practices in VFC, we reached consensus on initial training content to “flip,” with stepwise implementation allowing continual integration of learner feedback.

**Findings**

For hybrid trainings, we found no significant differences in pre-post changes in knowledge and confidence in IF skills for those attending in person vs. virtually at 2 weeks and 6-month follow-up (*p* > .05). Preliminary analysis for those attending 100% virtual trainings also revealed no statistical difference. We will present data from in-person, hybrid, and 100% virtual cohorts, then discuss transitioning to a partial VFC through modularizing select IF content.

**Implications for D&I Research**

Findings indicate virtual IF training increases knowledge and confidence in skills, and format does not degrade effectiveness. We are unaware of IF training implemented rigorously with a model involving synchronous, asynchronous, and independent learning components. Hence, evaluation of our model will yield important information about effective transfer of IF knowledge/skills in platforms best suited to learner needs to support the expansion of IF training capacity and reach.

**Primary Funding Source**

Department of Veterans Affairs

### S35 Costs of implementing context factor assessments into pain clinics

#### Carol Greco^1^, Rani Elwy^2^, Simone Taubenberger^3^, Nathan Dodds^4^, Rebecca DeSensi^4^, Andrea Gillman^4^, Ajay Wasan^5^

##### ^1^University of Pittsburgh, Pittsburgh, PA, USA; ^2^Veterans Health Administration, Providence, RI, USA; ^3^Magee-Womens Research Institute, Pittsburgh, PA, USA; ^4^UPMC, Pittsburgh, PA, USA; ^5^University of Pittsburgh School of Medicine, Pittsburgh, PA, USA

###### **Correspondence:** Carol Greco (grecocm@upmc.edu)

*Implementation Science 2023,* **18(Suppl 3):**S35

*Background*: The Healing Encounters and Attitudes Lists (HEAL) patient-reported measures consist of several separate context-factor questionnaires, including Treatment Expectancy, Patient-Provider Connection, Positive Outlook, and Attitudes towards Complementary/Alternative Medicine. These context factors assessments predict patients’ pain improvements. Our PCORI-funded implementation project added these context factors assessments into the workflows of seven UPMC Pain Medicine clinics. The implementation project demonstrated success in using HEAL data during clinic consultations to enhance patient engagement, improve patient outcomes, and reduce opioid prescribing.

The current project focused on determining costs associated with our implementation of HEAL context factors assessments into the Pain Medicine clinics. The reach of the original implementation project was 24,018 patients and 74 clinic personnel. Based upon the experience in the original implementation project, we aimed to determine the resources needed for additional sites to implement HEAL to improve pain care treatment.

*Methods*: This project was an observational study conducted from March 1 to November 30, 2021. We assessed implementation cost data from invoices, time and salary requirements for clinic personnel training, estimates of non-site-based costs and one-time resource development costs. The Stages of Implementation Completion checklist guided documentation of pre-implementation, implementation, and sustainment activities of HEAL pain clinic operations. These informed the calculations of the costs of implementation.

*Findings*: Total time for HEAL implementation is 7 months: pre-implementation and implementation phases (4 months) and sustainment (3 months). One hour of HEAL implementation involving a future clinical site consisting of 2 attending physicians, 1 midlevel provider, 1 nurse manager, 1 nurse, 1 radiology technician, 2 medical assistants, and 1 front desk staff will cost $572. A ten-minute time increment for all clinic staff is $95. Total implementation costs based on hourly rates over 7 months, including non-site-based costs of consultations, materials, and technology development costs, is $28,187.

*Implications for D&I Research:* Documenting our implementation costs clarifies the resources needed for additional new sites to implement HEAL to measure pain care quality and to engage patients and clinic personnel.

**Primary Funding Source**

Patient-Centered Outcomes Research Institute

### S36 The cost of rethink the strip: de-implementing a low value practice in primary care

#### Lisa Spees^1^, Laura Young^1^, Jennifer Leeman^2^, Jennifer Rees^1^, Marcella H. Boynton^3^, Erica Richman^4^, Kathleen Mottus^5^, Maihan Vu^1^, Annie Chen^6^, Katrina Donahue^1^

##### ^1^University of North Carolina at Chapel Hill, Chapel Hill, NC, USA; ^2^Lineberger Comprehensive Cancer Center, Chapel Hill, NC, USA; ^3^University of North Carolina Chapel Hill, Chapel Hill, NC, USA; ^4^Cecil G. Sheps Center for Health Services Research at the University of North Carolina at Chapel Hill, Chapel Hill, NC, USA; ^5^North Carolina Network Consortium – Practice Based Research Network, Chapel Hill, USA; ^6^UNC School of Medicine, Chapel Hill, NC, USA

###### **Correspondence:** Lisa Spees (lspees21@live.unc.edu)

*Implementation Science 2023,* **18(Suppl 3):**S36

*Background*: Routine self-monitoring of blood glucose (SMBG) is a low value practice that provides limited benefit for patients with non-insulin treated type 2 diabetes (T2DM). We estimated the costs of Rethink the Strip (RTS), multi-strategy approach to the de-adoption of SMBG in primary care.

*Methods*: RTS was evaluated in 20 primary care clinics across North Carolina. We estimated the non-clinic-based and clinic-based costs of the five RTS strategies (practice facilitation, audit and feedback, provider champions, educational meetings, and educational materials) from the analytic perspective of an integrated healthcare system. We selected a 27 month time horizon to align with the length of the RTS intervention. Material costs were tracked through project records, and personnel costs were assessed using micro-costing. Specifically, the practice facilitator tracked their time spent delivering facilitation, audit and feedback, and educational materials (i.e., traveling to clinics, contacting clinic personnel), and the RTS study staff tracked their time performing tasks to develop implementation strategies (i.e., updating educational materials, assembling audit and feedback reports). Clinic staff (e.g., provider champions) were tracked time spent on RTS activities for a total of 3 months. We used hourly wages from the US Bureau of Labor (BLS) Statistics were used to estimate personnel costs.

*Findings*: From the healthcare system perspective, total RTS costs equaled $96,790. Specifically, non-clinic-based costs comprised $20,564. The majority of non-clinic-based costs ($17,564) were from the foundational programming and coding updates to the EHR data develop the audit and feedback reports. The non-clinic-based costs of educational meetings, practice facilitation, and educational materials were substantially lower, ranging between $700 to $1500. Total clinic-based costs equaled $3,811 for a single clinic (or $76,226 for 20 clinics). Educational meetings were the most expensive strategy, costing on average $1,401 per clinic. Practice facilitation and provider champions clinic-based costs were approximately $1000 each per clinic. Lastly, clinic-based costs for audit and feedback and educational materials were markedly lower, at an average of, respectively, $270 and $101 per clinic.

*Implications for D&I Research:* This study provides detailed cost information of implementation strategies used in community-based primary care clinics and demonstrates the affordability of interventions employing implementation strategies to healthcare systems.

**Primary Funding Source**

Patient-Centered Outcomes Research Institute

### S37 Costs associated with the implementation of a patient decision aid for uterine fibroids in multiple healthcare settings

#### Stephanie C. Acquilano^1^, Rachel Forcino^2^, Danielle Schubbe^2^, Jaclyn Engel^3^, Marisa Tomaino^4^, Lisa C. Johnson^2^, Marie-Anne Durand^1^, Glyn Elwyn^1^

##### ^1^The Dartmouth Institute for Health Policy and Clinical Practice, Lebanon, NH, USA; ^2^Dartmouth, Lebanon, NH, USA; ^3^The Dartmouth Institute of Health Policy and Clinical Practice, Lebanon, NH, USA; ^4^The Dartmouth Institute for Health Policy & Clinical Practice, Lebanon, NH, USA

###### **Correspondence:** Stephanie C. Acquilano (stephanie.c.acquilano@dartmouth.edu)

*Implementation Science 2023,* **18(Suppl 3):**S37

*Background*: Decisions to implement innovations in clinical practice, such as patient decision aids, are often cost-dependent. Research on the costs of implementing patient decision aids is limited and based on very little empirical data. Within a multisite study based at five gynecology clinics, we estimated the cost of implementing a patient decision aid designed to facilitate shared decision-making for individuals diagnosed with uterine fibroids.

*Methods*: We followed a time-driven activity-based costing approach, which requires information on who accomplishes specific actions, when, and how often. We gathered data on pre-implementation steps, integration of the tools into the electronic health record (EHR), implementation steps, and sustainability costs. Data were obtained primarily by conducting qualitative interviews with key stakeholders and by examining internal documentation.

*Findings*: We interviewed 41 stakeholders and analyzed 56 documents. Initial planning and EHR integration were the highest contributors to costs. These costs were largely personnel expenses (staff activities to accomplish these tasks), and, in this project, were absorbed by existing staff. Costs varied across sites based on several factors, such as the clinic’s capacity for EHR integration, whether they already used other decision aids, and how they typically shared information with patients. Utilization of the tools in clinical workflows requires very little marginal work and time: most systems will be able to absorb these costs. Most health systems will outsource the work of developing and updating patient-facing materials and the tasks of training and maintaining the skills required to use them. [*Note: this section will be updated with specific cost findings.*]

*Implications for D&I Research:* There are predictable costs incurred with implementing patient decision aids, which depend on the complexity of workflow integration proposed. The intangible costs associated with cultural change and sustained modification of workflow issues are more difficult to calculate. Many of the costs of implementing patient decision aids to support shared decision-making are predictable, and our findings help elucidate those. Costs associated with changing culture, practice patterns, and workflow are more elusive, and these factors will affect sustained implementation.

*Timeline for Complete Data* Data collection is complete; analysis is in process. Analysis will be complete and data ready for presentation by September 2022.

**Primary Funding Source**

Patient-Centered Outcomes Research Institute

## Clinical Care Settings: Patient-level Interventions

### S38 Care coordination among rural dementia service providers

#### Beth Prusaczyk

##### Washington University School of Medicine in St. Louis, St Louis, MO, USA

###### **Correspondence:** Beth Prusaczyk (beth.prusaczyk@wustl.edu)

*Implementation Science 2023,* **18(Suppl 3):**S38

**Background**

Older adults in rural areas are at a higher risk for developing dementia than their urban counterparts, few dementia-related services are available in rural areas, and there are significant barriers to accessing them. Both dementia caregivers and care providers in rural areas cite a lack of integration among services. This fragmented rural dementia care network has been cited as one reason for the delay from symptom onset to diagnosis to treatment. Given the documented need for improved care coordination among rural dementia service providers, an important next step is identifying the barriers to care coordination so that interventions can be developed specifically to address these barriers.

**Methods**

Care coordination is a systemic property of healthcare and thus, systems science approaches are needed to understand care coordination. In this study, a network analysis will be conducted in two rural dementia service networks to identify providers in the network, existing care coordination patterns, and perceived barriers and facilitators to care coordination. An online survey will be distributed, using snowball sampling, to dementia health and social service providers in the rural areas. Survey questions will include: (1) awareness of and contact frequency with other providers, (2) types of care coordination (e.g., patient referrals, sharing resources), and (3) facilitators and barriers to care coordination.

**Findings**

The network’s structure will be analyzed, including measuring the network’s size, density, centralization, and modularity, which will result in information on the importance of specific providers and how different providers (i.e., network nodes) group together. Exponential random graph models will also be used to predict the likelihood of two providers coordinating based on provider-level and network-level characteristics. This type of modeling results in powerful network structural knowledge that will be useful for future intervention development and implementation.

**Implications for D&I Research**

Utilizing a network science approach to understand current care coordination patterns among rural dementia providers can provide unique insights that traditional methods cannot reveal. This systems-level view can be used to design future care coordination interventions that, by their nature, are systems-level interventions. Importantly, it can also provide valuable information on how these interventions can be implemented across the rural dementia provider networks.

**Primary Funding Source**

National Institutes of Health

### S39 Developing and implementing a care partner intervention in home-delivered meal systems

#### Lisa Juckett

##### The Ohio State University, Columbus, OH, USA

###### **Correspondence:** Lisa Juckett (lisa.juckett@osumc.edu)

*Implementation Science 2023,* **18(Suppl 3):**S39

**Background:** Over one-quarter of people living with dementia (PLWD) in the community are malnourished, leading to the devastating loss of independence. To combat malnourishment, home-delivered meal programs provide nutritional support to nearly 700,000 PLWD – over half of whom live with care partners. Evidence-based care partner interventions have been effective for reducing care partner stress and improving the quality of life of PLWD, but these interventions have not been tailored to the home-delivered meal context. Accordingly, the purpose of this study is to develop a care partner intervention – NU-CARE (Nutritional support Upskilling for CARE partners) – designed to support the nutritional health of PLWD. We will also identify strategies to support NU-CARE’s future implementation in home-delivered meal systems.

**Methods:** Based on tenets of Intervention Mapping, NU-CARE will be developed in two phases. In Phase 1, we will complete observational assessments around mealtime with PLWD-care partner dyads (N = 40) as well as semi-structured interviews with home-delivered meal providers and dementia care professionals (e.g., dietitians, speech-language pathologists, occupational therapists). In Phase 2, we will use a modified Delphi approach (N = 25) to develop the core functions of NU-CARE, its goals, and strategies to support its implementation.

**Findings:** Results of the observational assessments and semi-structured interviews completed in Phase 1 (completed by Nov 2022) will allow our team to identify the modifiable factors influencing the nutritional health of PLWD and the role of care partners in supporting nutritional needs. The most important and feasible factors to target through NU-CARE will be determined using our three-round Delphi approach, culminating in the Delphi panel’s selection of NU-CARE’s core theory- and evidence-based functions. Delphi panelists will also identify strategies to support NU-CARE’s implementation in real-world home-delivered meal settings.

**Implications for D&I Research:** This study will use a systematic approach to develop an intervention with its future implementation in mind. Too often, interventions are designed only with regard to efficacy – not implementation. This study serves as a prime example of how implementation science methods can be integrated into the early phases of intervention development while also advancing support for PLWD as well as their care partners in the community.

**Primary Funding Source**

National Institutes of Health

### S40 Applying d & i science to support dementia care in skilled nursing facilities

#### Natalie Douglas

##### Central Michigan University, Mt. Pleasant, MI, USA

###### **Correspondence:** Natalie Douglas (natalie.douglas@cmich.edu)

*Implementation Science 2023,* **18(Suppl 3):**S40

**Background:** People living with dementia (PLWD) can experience ineffective communication with care partners resulting in rejection of care and responsive behaviors such as yelling and lashing out verbally or physically. Certified nursing assistants (CNAs) provide the majority of care for PLWD in skilled nursing facility settings (SNF), yet they lack effective training and tools for communicating with PLWD.

**Methods:** A pre-implementation study in 5 SNFs using the *Acceptability of Intervention Measure*, *Intervention Appropriateness Measure*, *Feasibility of Intervention Measure*, a perceived knowledge and self-efficacy measure, the *Cohen-Mansfield Agitation Inventory*, and qualitative data analyzed according to the Consolidated Framework for Implementation Research (CFIR), was employed to assess the feasibility of a 6-week communication strategy intervention administered by a speech-language pathologist (SLP) to a PLWD and CNA.

**Findings:** Immediately post intervention, participants (n=18; n=6 SLPs, n=12 CNAs) rated the intervention as acceptable (M = 4.5, SD = .48), appropriate (M = 4.4, SD = .58) and feasible (M = 4.28, SD = .49) where a rating of ‘5’ equals more acceptable, appropriate, and feasible, and ‘1’ equals less acceptable, appropriate, and feasible. There was a significant improvement in perceived knowledge and self-efficacy from pre-intervention (M = 3.9, SD = .70) to post-intervention (M = 4.24, SD = .47); t(16)=-2.23, p = .02, where a rating of ‘5’ represents more knowledge and self-efficacy and a rating of ‘1’ represents less knowledge and self-efficacy. There was a significant improvement in scores on the *Cohen-Mansfield Agitation Inventory* for PLWD (n=10) from pre-intervention (M = 73.10, SD = 29.98) to post-intervention (M = 58.6, SD = 18.82); t(9)=2.83, p = .01, where a score of ‘29’ is the least number of negative, responsive behaviors and a score of ‘203’ is the highest number of negative, responsive behaviors. Analysis of 40% of written information in intervention manuals revealed themes of relative advantage, networks and communications, culture, tension for change, and leadership engagement.

**Implications for D&I Research:** D & I science, particularly a nuanced understanding of the complex context and provider perceptions, can support the development of feasible interventions to support dementia care in SNFs.

**Primary Funding Source**

National Institutes of Health

### S41 Implementing the individualized positive psychosocial intervention (ippi) in nursing home communities: a quality improvement project

#### Katherine Abbott^1^, Alexandra Heppner^2^, Reese Moore^3^, Miranda Corpora^1^, Megan Kelley^1^, Dr. Kimberly VanHaitsma^4^

##### ^1^Miami University, Oxford, OH, USA; ^2^Scripps Gerontology Center at Miami University, Oxford, OH, USA; ^3^Miami University's Scripps Gerontology Center, Oxford, OH, USA; ^4^The Pennsylvania State University, University Park, PA, USA

###### **Correspondence:** Katherine Abbott (abbottkm@miamioh.edu)

*Implementation Science 2023,* **18(Suppl 3):**S41

**Background**

Over 75% of people living with dementia (PLWD) experience behavioral and psychological symptoms of distress (BPSD). Expressions of distress (e.g., wandering, persistent vocalizations, and refusal of care) can be upsetting to the individual and care providers. Evidence-based interventions that take a person-centered approach to addressing these concerns are limited. The Individualized Positive Psychosocial Intervention (IPPI) is an evidence-based program that engages PLWD in brief (i.e.,10 minute) one-to-one preference-based activities 2 times a week. The goal of this quality improvement project (QIP) was for nursing home (NH) providers to implement the IPPI with 3 to 5 of their residents living with dementia.

**Methods**

Provider participants were recruited between July and December 2021. Sixty providers expressed interest, 31 completed the virtual one-hour orientation, and 18 started implementation. The implementation team was asked to complete a 2.5 hour online self-paced training, received virtual on-demand coaching, and provided data back to the research team.

**Findings**

Of the 11 providers who have finished the project to date, 100% completed the online training in emotion focused communication. Completers engaged 42 residents in 536 IPPIs (an average of 12 IPPIs per resident (SD 12.39).

Residents involved in the IPPI programs had a mean age of 84 (SD 8.8), 82% were female, 80% were white, with an average Brief Inventory of Mental Status (BIMS) of 6.5 (SD 4.2) out of a possible 15. In addition, 50% experienced BPSD in the month prior to starting the IPPI program.

Of the IPPI activities performed, resident mood improved during 40%, stayed the same in 59%, and declined in 0.4%. The majority (99%) of residents said that they would like to engage in the IPPI again. The majority (94%) of staff reported that the IPPI activity was a meaningful use of their time with the resident.

**Implications for D&I Research**

We attribute the drop off in participation to the spread of the omicron COVID-19 variant that disrupted many provider initiatives during Fall 2021 and the continued staffing crisis in NH settings. Even in the face of these major barriers, 58% of providers are on track to complete the project by the end of September 2022.

**Primary Funding Source**

Ohio Department of Medicaid

### S42 Integrating implementation science methodology into hybrid effectiveness-implementation studies of digital/mobile health interventions: toward a proactive and scalable approach

#### Jacqueline Hodges^1^, Wendy Cohn^1^, Tabor Flickinger^1^, Sylvia Caldwell^1^, Chloe Garofalini^2^, Olivia Kirby^3^, Rebecca Dillingham^1^, Amanda Castel^2^, Karen Ingersoll^4^

##### ^1^University of Virginia, Charlottesville, VA, USA; ^2^The George Washington University, Washington, DC, DC, USA; ^3^The George Washington University, Washington, DC, USA; ^4^University of Virginia, Charlottesville, VA, USA

###### **Correspondence:** Jacqueline Hodges (jchodges03@gmail.com)

*Implementation Science 2023,* **18(Suppl 3):**S42

**Background:** Digital health and mobile health (mHealth) interventions (DH/mHi) range from standalone commercial products to multi-faceted adjunctive tools requiring significant inputs from organizations deploying them. Guidance on the integration of implementation science (IS) methods into hybrid effectiveness- implementation studies of DH/mHi is limited within the literature.

**Methods:** We conducted a pre-trial preparation phase for the implementation evaluation arm of a hybrid effectiveness-implementation study, specifically a cluster randomized trial to test the mHealth intervention PositiveLinks, a clinic-associated smartphone platform, to support people with HIV in the DC Cohort in Washington, DC against usual outpatient care (n=6 clinics per arm). This multi-component intervention requires engagement from many stakeholders for implementation at the individual, clinic, and systems levels. We conducted literature review on DH/mHi, relevant theoretical IS frameworks, hybrid studies, and epidemiological cohort studies to develop our implementation evaluation aims: a) define and measure implementation outcomes of interest and b) elucidate determinants of intervention implementation.

**Findings:** We refined the necessary steps to duplicate this effort as follows: 1) Define components of the intervention and implementation strategy 2) Select appropriate IS frameworks to accomplish evaluation aims specific to prioritized components of the DH/mHi 3) Map framework domains/constructs to strategy steps and planned trial procedures 4) Modify/create instruments for IS data collection in conjunction with trial procedures and 5) Develop a data collection/management plan compatible with desired implementation outcome measures. We selected the Reach Effectiveness Adoption Implementation Maintenance (RE-AIM) framework to define implementation outcomes of interest, and integrated constructs of the Consolidated Framework for Implementation Research (CFIR) relevant to DH/mHi into trial follow-up assessments for rapid dissemination among a large sample of participants. We found that cohort studies may allow for examination of previously understudied IS framework components by tracking cohort patients approached but declining an intervention, including representativeness, true ‘denominators’ of stakeholders eligible for intervention uptake, and richer qualitative data on non-participation.

**Implications for D&I Research:** We provide a roadmap for pre-trial preparation toward precise application of theoretical IS frameworks within hybrid clinical trials testing complex DH/mHi. Our process yields novel insight on the potential benefits of embedding hybrid trials within epidemiological cohort studies.

**Primary Funding Source**

National Institutes of Health

### S43 Leveraging user-centered design and implementation science to co-design and implement a telehealth-enhanced hybrid cardiac rehabilitation program in a real-world clinical setting

#### Andrea T. Duran^1^, Adrianna Keener-DeNoia^1^, Kimberly Stavrolakes^2^, Emily Fleisch^3^, Nicole Pieszchata^3^, Charles Keys McKay^1^, Emma Whittman^4^, Diane Cannone^5^, Rachel C. Shelton^6^, Donald Edmondson^1^, Nathalie Moise^1^

##### ^1^Columbia University Irving Medical Center, New York, NY, USA; ^2^New York Presbyterian Hospital, New York, NY, USA; ^3^NewYork-Presbyterian, New York, NY, USA; ^4^Columbia Mailman School of Public Health, New York, USA; ^5^Columbia University Irving Medical Center, New York, USA; ^6^United States, Columbia Mailman School of Public Health, New York, NY, USA

###### **Correspondence:** Andrea T. Duran (atd2127@cumc.columbia.edu)

*Implementation Science 2023,* **18(Suppl 3):**S43

**Background:** Innovative program designs and strategies are needed to support the uptake of cardiac rehabilitation (CR) programs in the post-COVID19 era. We combined user-centered design (UCD) principles and implementation science (ImSci) methods to design a novel telehealth-enhanced hybrid (home and clinic-based) CR (THCR) program.

**Methods:** As part of a New York Presbyterian Hospital (NYPH) quality improvement initiative (March 2020-February 2022), we designed a THCR program as an alternative to traditional clinic-based CR. To achieve this goal, we engaged in a theory-informed, iterative three step UCD process guided by ImSci methods to: 1) identify user and contextual factors (semi-structured stakeholder interviews), 2) design intervention prototype (design workshops and journey mapping), and 3) review and refine intervention prototype (usability-testing). The process was informed by the Theoretical Domains Framework and Consolidated Framework for Implementation Research with an overall goal of optimizing both usability and implementation outcomes.

**Findings:** Step 1: Previously described semi-structured interviews with key stakeholders (n=9) at 3 geographically diverse, academic medical centers revealed behavioral (e.g., self-efficacy, knowledge) and contextual (e.g., social distancing guidelines, physical space/staffing, staff capacity, reimbursement) barriers to CR. Step 2: Design workshops (n=20) and journey-mapping sessions (n=3) with multi-disciplinary NYPH stakeholders (e.g., digital health team, CR clinicians, creative directors) yielded a THCR prototype that leveraged NYPH’s investment in Philips Healthcare’s remote patient monitoring (RPM) platform to optimize feasibility of home-based sessions. Step 3: “Live” usability testing with CR clinicians (n=2) administering and CR patients (n=3) participating in the home-based sessions revealed key usability challenges (RPM devices/exercise equipment set-up; Wi-Fi/Bluetooth connectivity/syncing; patient safety/knowledge and protocol flexibility). We simultaneously engaged in an iterative series of design workshops (n=24) and journey-mapping sessions (n=3) that yielded design solutions (e.g., onboarding sessions, safety surveys, fully supervised remote sessions) and a refined THCR prototype.

**Implications for D&I Research:** The refined THCR intervention is being implemented at a NYPH CR clinic in a pilot randomized controlled trial. Our study has implications for whether combining ImSci and UCD methods will result in a more acceptable, effective, and feasible THCR program, potentially maximizing our ability to reduce the evidence-to-practice gaps in CR implementation.

**Primary Funding Source**

National Institutes of Health

### S44 Implementation of electronic health record-based interventions to address social determinants of health in pediatric primary care: clinician, staff, and parent perspectives

#### Jennifer LeLaurin^1^, Jacqueline De La Cruz^1^, Ryan Theis^1^, Lindsay Thompson^2^, Ji-Hyun Lee^3^, Elizabeth Shenkman^2^, Ramzi Salloum^1^

##### ^1^University of Florida College of Medicine, Gainesville, FL, USA; ^2^Department of Health Outcomes and Biomedical Informatics, College of Medicine, University of Florida, Gainesville, FL, USA; ^3^University of Florida, Gainesville, FL, USA

###### **Correspondence:** Jennifer LeLaurin (jlelaurin@ufl.edu)

*Implementation Science 2023,* **18(Suppl 3):**S44

**Background:** Social determinants of health (SDH) screening and referral to resources are effective in improving child health and social outcomes. Electronic health record (EHR) modules are available to support these interventions, but are infrequently used. This ongoing mixed-methods study aims to identify approaches for implementing interventions using the Epic EHR SDH module in pediatric primary care.

**Methods:** We conducted three focus groups with clinicians/staff (n=24) and interviews with parents (n=15) from four pediatrics clinics. We used the Consolidated Framework for Implementation Research and Theoretical Framework of Acceptability to guide the study and focused on EHR-based intervention components. Focus group participants completed the Acceptability of Intervention Measure (AIM), Intervention Appropriateness Measure (IAM), and Feasibility of Intervention Measure (FIM). We used a template analysis approach and inductive coding to analyze qualitative data. The study will be completed in August 2022.

**Findings:** Mean (SD) scores for AIM, IAM, and FIM (range 1-5) were 4.48 (0.59), 4.52 (0.64), and 3.94 (0.70). SDH interventions were acceptable to all participants. Some clinicians/staff were aware of the SDH module, but never received training on its use. Disadvantages to using EHR- versus paper-based screening included clinicians being less likely to review results. Most parents felt patient portal screening was feasible; clinic/staff response varied by clinic, with some noting parents with SDH-related needs would be least likely to use this method. Some parents and clinicians/staff thought portal-based screening would reduce stigma, while others felt in-person screening would elicit more honest responses. Many parents were comfortable with EHR documentation of SDH, although some worried it could be used as evidence of neglect. Clinicians/staff suggested engaging clinical support staff in SDH interventions, but parents typically preferred pediatricians or nurses. Clinicians/staff recommended providing resource lists to reduce social worker burden, but most parents preferred help navigating services. Using text messaging, reducing stigma, and reducing duplicative screening and documentation were identified as important strategies by both parents and clinicians/staff.

**Implications for D&I Research:** While areas of convergence were identified, findings highlight conflicting views among clinicians, staff, and patients on approaches to implementing EHR-based SDH interventions. Future work should use implementation strategies that are acceptable and feasible for all stakeholders.

**Primary Funding Source**

Florida Department of Health

### S45 Patient-level responsiveness to an electronic health record-integrated electronic symptom management program deployed in the routine-care setting across six health systems

#### Raymond Osarogiagbon^1^, Christine Cronin^2^, Michael Hassett^2^, Angela Tramontano^2^, Jessica Bian^3^, Don Dizon^4^, Hannah Hazard-Jenkins^5^, Sandra Wong^6^, Deborah Schrag^7^

##### ^1^Baptist Memorial Health Care Corporation, Memphis, TN, TN, USA; ^2^Dana-Farber Cancer Institute, Boston, MA, USA; ^3^Veterans Health Administration, Portland, ME, USA; ^4^LifeSpan, Providence, RI, USA; ^5^Veterans Health Administration , Morgantown, WV, USA; ^6^Dartmouth Hitchcock Medical Center, Lebanon, NH, USA; ^7^Memorial Sloan Kettering Cancer Center, New York, NY, USA

###### **Correspondence:** Raymond Osarogiagbon (rosarogi@bmhcc.org)

*Implementation Science 2023,* **18(Suppl 3):**S45

**Background**

Electronic patient-reported outcomes (ePRO) improve cancer care quality and outcomes, but implementation is challenging. We evaluated patient-level responsiveness (PLR) to an ePRO-based symptom management program (eSyM) deployed in the routine-care setting across six health systems via a pragmatic cluster randomized trial.

**Methods**

All patients receiving medical (MO) or surgical (Surg) care for gastrointestinal, gynecologic or thoracic malignancies were assigned to eSyM as part of routine care. Using standard statistical methods, we examined patient characteristics associated with PLR. The primary outcome was patient response to at least one eSyM symptom questionnaire.

**Findings**

From September 2019 to June 2022, 8042 MO and 8673 Surg patients were invited to report their symptoms; 44% and 49%, respectively, responded at least once. For both MO and Surg patients, factors significantly associated with PLR were younger age, female, non-Hispanic, White, employed, private insurance, and diagnosis of gynecologic cancer (all, p<.0001). In multivariate analysis (Table 1), all factors except insurance remained significant.

**Implications for D&I Research**

PLR to an ePRO-based program deployed as part of routine care demonstrated moderate reach, but varied significantly by sociodemographic and clinical characteristics, emphasizing the need to adapt implementation to improve patient engagement and avoid exacerbating pre-existing disparities.

**Primary Funding Source**

National Institutes of Health

**Table 1 (abstract A45).** Factors independently associated with eSyM responsiveness

	MO (N=8042)			Surg (N=8673)		
	% (mean, IQR)	OR	p-value	% (mean, IQR)	OR	p-value
**Age**	(67, 15)	0.97	<0.0001	(68, 19)	0.98	<0.0001
**Sex**						
Male (reference)	46			33		
Female	54	1.26	<0.0001	68	1.26	<0.0001
**Race**						
White (reference)	80			91		
Black	15	0.72	<0.0001	3	0.38	<0.0001
**Ethnicity**						
Not Hispanic (reference)	92			95		
Hispanic	3	0.58	0.002	3	0.63	0.002
**Marital Status**						
Married (reference)	54			58		
Single	19	0.61	<0.0001	19	0.61	<0.0001
Divorced	11	0.66	<0.0001	11	0.69	<0.0001
Widowed	13	0.71	<0.0001	8	0.53	<0.0001
**Employment**						
Employed (reference)	21			34		
Retired	48	0.89	0.13	38	0.74	<0.0001
Disabled	11	0.57	<0.0001	8	0.42	<0.0001
Not employed	11	0.53	<0.0001	12	0.52	<0.0001
**Condition**						
Gastrointestinal (reference)	36			42		
Gynecologic	13	1.3	0.001	28	1.44	<0.0001
Thoracic	27	0.95	0.36	19	1.15	0.03
**Insurance**						
Private (reference)	50			61		
Medicaid	3	0.60	0.0005	6	1.04	0.68
Medicare	40	1.21	0.001	23	1.03	0.61

### S46 Assessing implementation strategies for the uptake of bundled interventions to improve culturally relevant care for black women with hiv

#### Serena Rajabiun

##### University of Massachusetts, Lowell, Lowell, MA, USA

###### **Correspondence:** Serena Rajabiun (serena_rajabiun@uml.edu)

*Implementation Science 2023,* **18(Suppl 3):**S46

**Background:** The Health Resources and Services Administration, HIV/AIDS Bureau, Ryan White HIV/AIDS Program, Part F- Special Projects of National Significance initiative entitled *Improving Care and Treatment Coordination for Black women with HIV* funds 12 clinical and community based organizations and one evaluation center to adapt, implement, and assess the uptake of evidence based/informed (EB/EI) bundled interventions to improve health outcomes and well-being for Black women with HIV. This study examines the successes, challenges and adaptations to implementation strategies to support intervention uptake and cultural responsiveness for Black women with HIV.

**Methods:** Guided by the Expert Recommendations for Implementing Change (ERIC) compilation, we selected five implementation strategies for intervention uptake: tailoring interventions to local context, train/educate stakeholders, evaluation and iterative strategies, engaging consumers and changing infrastructure.^1^ Modifications to strategies were documented using FRAME-IS core modules gathered through monthly coaching calls with sites (n=12), and annual site visits with funder, evaluation center coaches and site implementation teams (n=24). Modifications occurred in pre-implementation and implementation phases. Adaptations to interventions were analyzed with Chambers & Norton’s framework. Interview transcripts and reports were managed using the NVivo 12.0 qualitative software. Data was analyzed utilizing a directed content analysis approach. ^2^ (Hsieh & Shannon, 2005).

**Findings:** Training modifications included: delivery via virtual formats; revision of curricula language to reduce stigma; use of images in educational materials that reflect Black women; and content focused on their needs including housing support, gender affirming care and mentoring for women who are caregivers. Using incentives and soliciting feedback on intervention content and delivery were important for engaging women to facilitate intervention uptake. For evaluation strategies, site implementers modified screening forms to include topics such as intimate partner violence and trauma. Challenges with intervention uptake and implementation strategies included: staff turnover, identification and recruitment of cisgender and transgender women, and workflow for and timing of implementing the bundled interventions within the organization and with partners.

**Implications for D&I Research:** This study contributes to the adaptations of strategies for promoting and implementing EB/EI interventions that are culturally relevant, include feedback mechanisms and meet the social and medical needs of Black women.

**Primary Funding Source**

Health Resources and Services Administration

### S47 Characterizing the use of community engagement strategies used to implement evidence informed bundled interventions for black women with hiv

#### Linda Sprague Martinez

##### Boston University School of Social Work, Boston, MA, USA

###### **Correspondence:** Linda Sprague Martinez (lsmarti@bu.edu)

*Implementation Science 2023,* **18(Suppl 3):**S47

**Background:** Client and community engagement (CE) can inform the implementation of evidence-informed interventions.^1^ Little is known about implementation strategies related to community engagement for delivering evidence informed bundled interventions for Black women in HIV care. This research seeks to fill this gap by characterizing implementation CE strategies employed as part of the Black Women First Initiative (BWF). BWF was launched to implement culturally responsive, evidence informed bundled interventions to meet the unique health needs of diverse Black women with HIV in 12 demonstration sites in the United States.

**Methods:** Mixed methods were employed to evaluate implementation of evidence informed bundled interventions within and across the demonstration sites. For this analysis we draw on qualitative data collection including initial interviews (n=12) conducted via Zoom with implementation sites and document review. Documents included monthly site call minutes (n=110) and site visit reports (n=24). Interviews and documents were managed using NVivo 12.0 qualitative software. Deductive codes were developed drawing on the Expert Recommendations for Implementing Change (ERIC) project,^2^ which developed a common language for implementation strategies and encourages their consistent use.^3^ Drawing on the strategy “prepare patients/consumers to be active participants” a code focused on patient/client engagement was developed and drawing on “network weaving” a code focused on community engagement was developed. The “advisory board” strategy was also included as part of each code definition. Directed content analysis was employed.^4^

**Findings:** We found sites were using four distinct CE implementing strategies: client advisory boards, community advisory boards, peer leadership and outreach/network-weaving. Client advisory boards inform patient engagement and the development of outreach materials and messages. Community advisory boards contribute to the development of training and technical assistance. Meanwhile, peer leaders, like client boards play important ambassadorial roles, endorsing intervention uptake as well as informing adaptation. Meanwhile, site staff engaged in focused outreach, that involved network weaving to promote information sharing across organizations to advance implementation.

**Implications for D&I Research:** This research advances our understanding of the ways in which CE is both an implementing strategy and indirectly facilitates implementation by enhancing other strategies such as education and training and media messaging.

**Primary Funding Source**

Health Resources and Services Administration

### S48 Advancing the uptake of implementation science in cbos: lessons learned adapting interventions and implementation strategies to improve care for black women with hiv

#### Andrea Dakin

##### AIDS Foundation Chicago, Chicago, IL, USA

###### **Correspondence:** Andrea Dakin (adakin@aidschicago.org)

*Implementation Science 2023,* **18(Suppl 3):**S48

**Background:** Women Evolving (WE) is an engagement in care program designed to serve HIV-positive cis- and transgender Black women. Funded through the HIV/AIDS Bureau of the Health Resources and Services Administration, WE launched in Spring 2021 and is focused on improving HIV outcomes and addressing the social determinants of participant health through the implementation of bundled evidence-based interventions (EBI). Using implementation science tools tailored specifically for use at the community-based organization (CBO) level, this evaluation identifies the modifications made to the EBIs both pre-launch and throughout the initial implementation for the specific population, setting and partnership model of the WE program.

**Methods:** The FRAME-IS framework was employed to systematically capture pre- and post-implementation modifications made to the EBIs at the CBO level. An EBI fidelity tracking matrix was designed by site staff to document specific program elements. This tool crosswalks the original elements of the EBI with organizational conceptualization of said elements at the program level. Data elements in the matrix include the specific strategy or activity modified, rationale for modification, intended impact of the change, and the extent to which the modified activity deviates from the original EBI. Modification data for the tracker were identified through monthly internal meetings, bi-monthly partnership meetings, and monthly feedback sessions with the technical assistance provider.

**Findings:** WE staff made changes to implementation strategies and adapted the EBIs to best meet the needs of the population, both pre- and during initial program implementation. Adaptations included layering trauma-informed care approaches onto the EBIs. Additionally, Covid-19 protocols in various settings combined with an increased use on telehealth approaches prevented successful utilization of known effective EBI implementation approaches such as in-person outreach and engagement. Changes were made to program recruitment and retention strategies, new approaches to client communication were developed, and complimentary services were incorporated in response to emergent client needs.

**Implications for D&I Research:** CBOs often modify EBIs for specific populations and adapt strategies in response to local context. These changes are rarely reported on in the literature due to resource constraints. Accessible implementation science tools can support CBO efforts to monitor, evaluate and dissemination adaptation, advancing the knowledge base.

**Primary Funding Source**

Health Resources and Services Administration

### S49 Pivoting quickly: testing implementation support for colorectal cancer screening in newly eligible 45–49-year-old population following guideline change

#### Meghan O'leary^1^, Alison Brenner^2,3^, Sara Correa^4^, Alexis Moore^4^, Teri Malo^1^, Daniel Reuland^2,5^

##### ^1^University of North Carolina at Chapel Hill, Carrboro, NC, USA; ^2^University of North Carolina at Chapel Hill, Chapel Hill, NC, USA; ^3^University of North Carolina School of Medicine, Chapel Hill, NC, USA; ^4^The University of North Carolina at Chapel Hill, Chapel Hill, NC, USA; ^5^The University of North Carolina School of Medicine, Chapel Hill, NC, USA

###### **Correspondence:** Meghan O'leary (mcoleary@live.unc.edu)

*Implementation Science 2023,* **18(Suppl 3):**S49

**Background:** When screening recommendations change, health organizations, especially lower-resource settings like Federally Qualified Health Centers (FQHCs), face implementation and resource challenges translating the new evidence into practice. In May 2021, the U.S. Preventive Services Task Force extended colorectal cancer (CRC) screening recommendations to adults aged 45-49. We responded by leveraging an existing partnership and adapting an existing intervention protocol to 1) implement mailed fecal immunochemical testing (FIT) outreach to support screening, and 2) experimentally test mailing materials for improving mailed FIT uptake in this population. We describe effectiveness outcomes here.

**Methods:** Building on an ongoing academic/community partnership and implementation research study, we implemented an expansion of a centralized mailed FIT outreach intervention to include this newly-eligible age group (45-49-year-olds) at a single FQHC clinic. We assessed uptake (effectiveness) by determining the proportion who returned completed FITs within 60 days of mailing. We also conducted a nested randomized trial to compare uptake in two intervention groups: 1) FIT mailed in a bright padded envelope with messaging stickers, versus 2) FIT mailed in a plain envelope. Finally, we determined the change in overall CRC screening in this population between baseline and 6-months post-intervention.

**Findings:** In January 2022, we mailed FITs to 316 patients: 57% female, 58% non-Hispanic Black, 27% non-Hispanic White, 8% Hispanic; 51% privately insured, 29% uninsured, and 13% Medicaid enrollees. Overall, 54/316 (17%) returned a FIT within 60 days. By study arm, 34/158 (22%) patients who received the enhanced envelope returned a FIT within 60 days compared to 20/158 (13%) who received the plain envelope (difference 8.9 percentage points, p=0.037, 95% CI: 0.6-17.2). Overall CRC screening among 45–49-year-olds at this site increased by 16.4 percentage points (from 26.0% at baseline to 42.4% at 6 months).

**Implications for D&I Research:** Our established partnership, as well as our centralized outreach approach, allowed us to quickly (within eight months) implement and evaluate approaches for promoting CRC screening uptake in a rural FQHC clinic after screening recommendations changed. We demonstrated that CRC screening rates increased following a mailed FIT intervention among diverse FQHC patients aged 45-49, especially when using more visually salient mailers.

**Primary Funding Source**

National Institutes of Health

### S50 Effects of modality, duration, and delivery features on reach and representativeness of diabetes shared medical appointments

#### Bethany Kwan^1^, Dennis Gurfinkel^2^, Natalie Ritchie^3^, Martha Sajatovic^4^, Madeline Carter^1^, Jeanette Waxmonsky^1^

##### ^1^University of Colorado Anschutz Medical Campus, Aurora, CO, USA; University of Colorado & Children's Hospital Colorado, Aurora, CO, USA; ^3^Denver Health and Hospital Association, Denver, CO, USA; ^4^University Hospitals Cleveland Medical Center, Cleveland, OH, USA

###### **Correspondence:** Bethany Kwan (Bethany.kwan@cuanschutz.edu)

*Implementation Science 2023,* **18(Suppl 3):**S50

**Background:** Diabetes shared medical appointments (SMAs) are an evidence-based approach to provide diabetes self-management support and education, yet patient participation is often suboptimal. It is unknown what features of SMAs can best promote participation. This analysis explores the reach and representativeness of diabetes SMAs among primary care practices in the Invested in Diabetes study.

**Methods:** Diabetes SMAs were delivered in 6 sessions using the Targeted Training in Illness Management curriculum. Twenty-six practices were randomized to a standardized (STD) delivery model (single health educator using the curriculum “as is”) or a patient-driven (PTD) model (team of health educators, behavioral health providers, and peer mentors, and patients picking their own topic order and emphasis). Using the RE-AIM framework, we tested the hypothesis that PTD features would better promote patient engagement and retention in SMAs. We also compared differences in patient characteristics and attendance by delivery modality, as the COVID-19 pandemic led most practices to change from in-person to virtual SMAs (vSMAs) midway through implementation.

**Findings:** Twenty-two practices reported data on 148 cohorts with 1085 patients. STD practices (n=11) had 73 cohorts with 577 patients, PTD practices (n=11) had 75 cohorts with 508 patients. Both groups were primarily female (62% STD; 55% PTD, p=.015), white (78% STD; 70% PTD, p=.004), non-Hispanic (74% STD; 63% PTD, p<.001), and younger (66% <65 years STD; 60% PTD, p=.2). Attendance was similar between STD and PTD practices (3.98 vs. 3.92 sessions, p=.81), and did not reach significance between in-person and vSMAs (3.88 vs. 4.08, p=.07). Overall attendance to early sessions was relatively high (81%, 75%, 67% of participants attended sessions 1, 2, and 3 respectively), and declined at later sessions (58%, 59%, 56% at sessions 4 5, 6, respectively).

**Implications for D&I Research:** Overall, a simpler delivery model of diabetes SMAs appears equally engaging as a more complex model, and may also be easier for practices to implement. Briefer interventions (<3 sessions) may be more widely appealing and limit declining attendance over time. Practices may also consider virtual delivery to reduce barriers to participation such as lacking transportation.

**Primary Funding Source**

Patient-Centered Outcomes Research Institute

### S51 Bundling colorectal cancer screening outreach with screening for social risk in federally qualified health centers: a stepped-wedge implementation-effectiveness pilot study

#### Gina Kruse^1,2^, Sanja Percac-Lima^2^, Daniel Gundersen^3^, Deepinder Singh^1^, Lynnette Mascioli^4^, Mehezbin Munshi^4^, Madeline Davies^1^, Karen Emmons^5^, Jennifer Haas^2^

##### ^1^Massachusetts General Hospital, Boston, MA, USA; ^2^Harvard Medical School, Boston, MA, USA; ^3^Dana Farber Cancer Institute, Boston, MA, USA; ^4^Massachusetts League of Community Health Centers, Boston, MA, USA; ^5^Harvard University, Boston, MA, USA

###### **Correspondence:** Gina Kruse (GKRUSE@mgh.harvard.edu)

*Implementation Science 2023,* **18(Suppl 3):**S51

**Background:** Bundling outreach for colorectal cancer (CRC) screening by fecal immunochemical test (FIT) with screening for social determinants of health (SDOH) may enable patients to engage in preventive care by addressing SDOH and produce efficiencies in resource-constrained settings.

**Methods:** In this clustered stepped-wedge trial, four Massachusetts federally-qualified health centers (FQHCs) were randomized to start implementation of an intervention over 8-week “steps” (10/2020-11/2021). The intervention bundled outreach to 50-75 yr-olds not up-to-date on CRC screening to offer FIT and SDOH screening. The implementation strategy used external facilitation and technical assistance for data reporting for each FQHC over two phases: 1) initial implementation (months 1-4) and 2) data-guided adaptation examining inequities in reach and effectiveness (months 5-8). The primary outcome was CRC screening by any guideline-based method. We compared screening rates in intervention and control FQHCs in each step by fitting generalized linear mixed effects models with random intercepts for FQHCs and patients to account for clustering of observations within FQHCs and multiple measurements among patients.

**Findings:** Each FQHC had a slightly different implementation model depending on their unique infrastructure, workflows, capacity and pandemic-related staffing demands. Two implemented the pilot using population health approaches with outreach calls and two integrated the intervention within established programs, such as pairing with high-risk patient community health worker programs and pre-visit planning. Of 34,588 eligible patients in the 4 FQHCs, 54% were female; 23% Black, 13% Latino, 12% Asian, and 52% white; 48% public insurance, 33% private insurance, and 11% uninsured. The average rate of CRC screening orders was higher among intervention FQHCs than among control FQHCs. The conditional odds ratios comparing CRC screening orders and completions in intervention to control FQHCs was 3.0 (p<0.001) and 2.7 (p<0.01), with average marginal effects of 2.8 and 0.9 percentage points increase, respectively. Sensitivity analysis excluding pre-covid baseline periods found a slight attenuation of effect but the same sign and significance at p<0.05.

**Implications for D&I Research:** This bundled intervention represents an effective solution to increase cancer screening with consideration of the impacts of individual social risks. This strategy has potential to address CRC screening deficits associated with the pandemic.

**Primary Funding Source**

National Institutes of Health

### S52 Implementing an evidence-based postpartum hemorrhage (PPH) bundle across the military health system (MHS)

#### Faye Curran, Daniella Kanyer

##### Department of Defense, Falls Church, VA, USA

###### **Correspondence:** Faye Curran (fay.curran@cognosante.com)

*Implementation Science 2023,* **18(Suppl 3):**S52

**Background**

The Military Health System (MHS) previously lacked practices for implementing clinical recommendations across its network of medical facilities. In 2020, the Research & Engineering Implementation Science Branch (ISB) at the Defense Health Agency (DHA), along with Women’s Health experts, leveraged implementation science best practices to facilitate the standardization, dissemination, and implementation of an eight-component, evidence-based postpartum hemorrhage (PPH) patient safety bundle at 12 Military Treatment Facilities (MTFs). Currently, PPH is the leading cause of preventable maternal death worldwide with MHS rates at 7.4 deaths per 100,000 live births (TRICARE, 2021).

**Methods**

While preventing PPH is not always possible, risk assessment and early treatment of PPH has been demonstrated to decrease the severity of maternal sentinel events, and practice standardization has improved overall medical readiness and outcomes (ACOG, 2017). From December 2020 to October 2021, a comprehensive, systematic, and targeted implementation approach was used to execute the bundle across the MTFs, including: conducting organizational capacity and baseline assessments, identifying MTF leaders and champions, facilitating peer work groups, developing standardized resources and materials, and creating a centralized web platform to share information. Additionally, a self-report data collection tool was designed to track each MTF’s progress toward implementation of each of the PPH bundle’s components as well as overall compliance.

**Findings**

In December 2020, prior to bundle implementation, a baseline implementation assessment measured an average compliance of 63% across all MTFs. By October 2021, all MTFs reached implementation scores above the pre-determined target of 80%, with an overall MHS-wide average of 97%. The self-report data and lessons from the initial 12 MTFs helped inform a larger-scale implementation effort across an additional 31 MTFs worldwide. As of July 2022, the average compliance across all 43 MTFs was 80.4%.

**Implications for D&I Research**

Adopting the evidence-based bundle offers a recognizable and reproducible process of care, ensuring the MHS is providing ready, reliable care for mothers and their babies. The next step is to understand the mediating influence of this bundle on clinical outcomes and gain an even better understanding of real-world impact.

### S53 Evaluating a hybrid type iii implementation trial of an integrated multidisciplinary clinic to address unsafe use of opioids among veterans living with chronic pain

#### Amanda Midboe^1^, Taryn Perez^1^, Shayna Cave^2^, Sara Edmond^3^, William Becker^3^

##### ^1^Center for Innovation to Implementation (Ci2i), Menlo Park, CA, USA; ^2^Center for Innovation to Implementation (Ci2i), VA Palo Alto Healthcare System Menlo Park, CA, USA; ^3^Veterans Health Administration, West Haven, CT, USA

###### **Correspondence:** Amanda Midboe (amanda.midboe@va.gov)

*Implementation Science 2023,* **18(Suppl 3):**S53

**Background:** While opioid overdose deaths continue to climb in the United States, chronic pain also remains prevalent. Preventing overdose deaths and supporting the unique needs of patients who are living with chronic pain requires not only evidence-based treatments options, but also improving access to them through implementation science. The Opioid Reassessment Clinic (ORC) model relies on a multidisciplinary team applying evidence-based practices in the treatment of those living with chronic pain and complex psychiatric comorbidities. The ORC model is integrated within primary care, with the objective of providing longitudinal co-management of pain and opioid use disorder while promoting engagement in non-pharmacological pain treatments (NPTs). As part of a hybrid type III trial, we evaluated the effect of Implementation Facilitation (IF) on the implementation of an ORC at three Veterans Health Administration (VA) sites.

**Methods:** This mixed-methods evaluation relied on the Consolidated Framework for Implementation Research (CFIR) and the RE-AIM framework to evaluate the use of IF across 18 months to implement an ORC at three VA sites in different regions of the United States. The external IF team comprised clinical and implementation experts. The IF activities were informed by regular meetings with the Opioid Addiction and Recovery – Veteran Engagement Board (OAR-VEB), which included a diverse panel of eleven men and women veterans in recovery.

**Findings:** Initial analyses indicate that 532 patients living with chronic pain and complex psychiatric comorbidities (81-91% of patients) were treated across 3 ORC sites. A total of 127 providers adopted the ORC or were trained on the model and its associated evidence-based treatment approach. Reductions in morphine equivalent daily doses (MEDD) have ranged from 34% to 75% decrease in MEDD at sites. Prescribing of buprenorphine, a safer alternative to full agonist opioids, increased significantly alongside referrals to NPTs. A formative qualitative evaluation revealed several relevant CFIR-related determinants for tailoring of IF and informing future dissemination efforts.

**Implications for D&I Research:** At three VA implementation sites, Implementation Facilitation was an effective implementation strategy to improve care for veterans living with chronic pain and complex psychiatric comorbidities. Veterans in recovery (OAR-VEB) played a role in shaping the IF activities throughout the implementation phase.

**Primary Funding Source**

Department of Veterans Affairs

### S54 Is there bias in delivery mode of a social care intervention for parents of a hospitalized child? Adaptation of the communityrx-hunger intervention during the covid-19 pandemic

#### Emily Abramsohn^1^, Victoria Winslow^1^, Amy Carter^2^, Mellissa Grana^1^, Jyotsna Jagai^1^, Doriane Miller^1^, Christine O'Malley^2^, Jennie Ott^2^, Eva Shiu^1^, Stacy Tessler Lindau^1^

##### ^1^University of Chicago, Chicago, IL, USA; ^2^University of Chicago Comer Children's Hospital, Chicago, IL, USA

###### **Correspondence:** Emily Abramsohn (eabramsohn@bsd.uchicago.edu)

*Implementation Science 2023,* **18(Suppl 3):**S54

**Background:** Pandemic-related restrictions imposed on the healthcare setting prompted a rapid shift in the delivery of evidence-based social care interventions previously evaluated in in-person settings. Adaptations of evidence-based interventions (EBIs) are often not documented sufficiently to study their impact on important outcomes of interest. CommunityRx-Hunger is an evidence-based social care intervention to address food insecurity among parents of hospitalized children by providing tailored resource referrals evaluated in a double-blind, randomized controlled trial.

**Methods:** Trained Navigators delivered three evidence-based components (EBCs) of the CommunityRx-Hunger intervention: education, resource activation and boosting. CommunityRx-Hunger was modified from in-person to remote delivery to accommodate pandemic-related restrictions on hospital visitors and researchers. Adaptations were enabled by engaging hospital staff and aligning with clinical workflows. The three EBCs of the intervention were retained; timing and mode of delivery was adapted and documented. Synchronous delivery, facilitated in-hospital through dispatch of a web-enabled tablet and technical support by Child Life Specialists (CLS), included all EBCs delivered by navigator via videoconference or phone during hospital admission and automated digital delivery by text and email post-discharge. Asynchronous delivery only included automated digital delivery of the EBCs post-discharge. All parents in the intervention group automatically received “booster” intervention text messages three months post-discharge. Using significance testing, we compared sociodemographic and health characteristics of parents who received synchronous vs. asynchronous intervention delivery, hypothesizing that no differences would exist.

**Findings:** Most parents were the mother of the hospitalized child (94%), median age 34 years (IQR: 28-40) and African American/Black (80%). All 319 parents randomized to the intervention arm received all three EBCs. Almost half (46%) of these parents received synchronous delivery of the intervention, and 78% of synchronous deliveries were facilitated by CLS. Sociodemographic (age, gender, race, ethnicity, partnership, income, education or household composition) and health characteristics (parent and child health, length of hospital stay or food security) were not significantly different between the two groups.

**Implications for D&I Research:** Navigator training and the double-blind trial design likely mitigated bias in delivery of the CommunityRx-Hunger intervention. Documentation of intervention adaptations allows for longitudinal analyses to better understand the impact of intervention delivery types on child and parent health and psychosocial outcomes.

**Primary Funding Source**

National Institutes of Health

### S55 Evaluating community health center social care activities

#### Emilia De Marchis^1^, Benjamin Aceves^2^, Na'amah Razon^3^, Laura Gottlieb^1^

##### ^1^University of California, San Francisco, San Francisco, CA, USA; ^2^San Diego State University, San Diego, CA, USA; ^3^University of California, Davis, Sacramento, CA, USA

###### **Correspondence:** Emilia De Marchis (emilia.demarchis@ucsf.edu)

*Implementation Science 2023,* **18(Suppl 3):**S55

**Background:** Though social risk data collection is expanding in community health centers (CHC), little research examines how increased availability of social risk information influences care delivery. We studied the collection and use of social data in Texas CHCs and facilitators/barriers to social care activities in pre/peri-COVID-19 pandemic periods.

**Methods:** Mixed methods: 1) provider and staff semi-structured interviews; 2) provider surveys; 3) electronic health records (EHR) review. Interviews and surveys explored perspectives on integrating social care activities; EHR data was used to assess social screening reach. Interviews were analyzed using thematic analysis and inductive coding; survey and EHR data were analyzed with chi-square descriptive statistics.

**Findings:** In four CHCs, we conducted 27 interviews (15 providers; 12 staff), collected 97 provider surveys, and reviewed EHR data (2/4 CHCs provided EHR data). Interviews and surveys indicated support for integrating social care, which increased peri-pandemic. 90% of survey respondents reported incorporating social screening into patient conversations; 46% of those screening used standardized screening tools. 29% of all survey respondents were unaware their clinic had an embedded standardized screening tool. EHR data showed numbers of screens per month and screens/encounters increased (4% of patient encounters in 8/2019 to 44% in 2/2021) after the pandemic began. We found significant differences in screening rates by race/ethnicity and language (p<0.001). Provider and staff reported social care barriers included: lack of time/staffing, language and cultural barriers, limited community resources, and poor coordination/communication about implementation efforts. Beyond making referrals to social services, providers used social data in care decision-making to improve medication affordability and change follow-up care planning.

**Implications for D&I Research:** Study CHCs were in the early stages of standardizing social care, focused more on awareness and assistance than adjustment or population health-level evaluations. Differences in screening reach by patient demographics raise concerns that social care might exacerbate disparities. Future research should explore equity in implementation and effectiveness of these interventions. Overcoming barriers to reach, sustainability, and equity will require supports targeted to program design and development, workforce capacity, quality improvement, and advocacy for community social service investments.

**Primary Funding Source**

Episcopal Health Foundation

## Clinical Care Settings: System-level Interventions

### S56 Implementation barriers to suicide prevention-focused clinical decision support

#### Colin Walsh

##### Departments of Biomedical Informatics, Medicine, Psychiatry, Vanderbilt University, Nashville, TN, USA

###### **Correspondence:** Colin Walsh (colin.walsh@vanderbilt.edu)

*Implementation Science 2023,* **18(Suppl 3):**S56

*Background:* Adolescent suicide prevention depends on risk identification, prognostication, and effective intervention. Scaling effective prevention might benefit from clinical decision support to improve decision-making, especially in settings in which suicide prevention care is uncommon. In ambulatory Neurology at Vanderbilt University, we have piloted Vanderbilt SafeCourse, a CDS suite comprised of the Vanderbilt Suicide Attempt and Ideation Likelihood (VSAIL) risk model linked to a custom Best Practice Advisory delivered in real-time to prompt clinical attention for those at elevated predicted risk of thirty-day suicide attempt. In launching the pilot pragmatic trial in Neurology, multiple key implementation barriers arose with relevance for similar efforts in the D&I community. These barriers were explored with a mixed-methods study.

*Methods:* Mixed methods cohort study. Quantitative evaluation of risk model performance at academic health system scale from June 2019 to the present. Qualitative analyses of current workflow (Think aloud, cognitive walkthrough). Human-centered design of CDS. Usability testing.

*Findings:* From June 2019 to April 2020, VSAIL generated 115,905 predictions for 77,973 (42490 [54%] men, 35404 [45%] women, 60 586 [78%] White, 12 620 [16%] Black). Numbers needed to screen in highest risk quantiles were 23 and 271 for suicidal ideation and attempt, respectively. Performance was maintained across demographic subgroups. Model C statistics were, across the health system: 0.836 (95% CI, 0.836-0.837); adult hospital: 0.77 (95% CI, 0.77-0.772); emergency department: 0.778 (95% CI, 0.777-0.778); psychiatry inpatient settings: 0.634 (95% CI, 0.633-0.636). Qualitative analyses highlighted key concerns across multiple domains: ethical; legal; equity; clinical. Ethical concerns related to informed consent requirements (for both patients and providers). Legal concerns related to liability and documentation requirements to prevent litigation. Equity concerns related to disparate resource allocation and access, notably in rural settings. Clinician comfort with suicide prevention varied widely as did comfort with CDS use in day-to-day clinical workflow.

*Implications for D&I Research:* Implementation barriers to uptake and deployment of scalable suicide prevention CDS reflects on barriers in other domains. Multistakeholder engagement early in implementation planning might mitigate some concerns, but the complexity of suicide and prevailing risk management sensitivity to this clinical problem necessitates iterative identification and surmounting of implementation barriers.

**Primary Funding Source**

National Institutes of Health

### S57 Clinician perspectives on suicide safety planning & behavioral nudge implementation strategies

#### Mira Bajaj

##### Johns Hopkins Medical Institute, Baltimore, MD, USA

###### **Correspondence:** Mira Bajaj (mbajaj1@jhmi.edu)

*Implementation Science 2023,* **18(Suppl 3):**S57

*Background:* Safety planning for suicide prevention has demonstrated effectiveness, and has been incorporated into best-practice treatment guidelines, interventions, and policies. Despite evidence that higher-quality safety planning is associated with improved patient outcomes, implementation has proved challenging in real-world settings. For example, at the participating research site: only 27% of patients identified as at risk for suicide had any documentation of safety planning in their charts. Understanding providers’ perspectives and practices surrounding safety planning, as well as their views on informatics-based behavioral nudge strategies, is critical to informing efforts to increase its implementation.

*Methods:* Pedaitric care professionals in an academic medical center were recruited via email to complete an online survey. The survey assessed participants’ level of training and comfort with safety planning, what elements of safety planning they utilize regularly, and thoughts and feedback on a behavioral nudge strategy. Questions were adapted from the Consolidated Framework for Implementation Research (CFIR) Barrier Buster Tool and assessed potential facilitators and barriers to utilizing safety planning, as well as, modifications to porposed implementation strategy.

*Findings:* A total of 93 participants responded to the survey. Participants included 23 physicians, 13 nurses, 13 psychologists, 13 social workers, and 31 in other professional roles. Approximately half of the participants (48.9%) endorsed having formal training with safety planning. On average participants reported a high level of comfort (mean = 74.8/100; SD = 25.3) with safety planning. Sixty-five percent of participants endorsed using all six core elements of safety planning. The top implementation facilitators were the team valuing patient needs and safety planning being a relative priority for leadership. The top barriers to safety planning were having enough time and open lines of communication. Reactions to the behavioral nudge strategy were favorable, with 52% of providers emphasizing that it would help increase safety planning.

*Implications for D&I Research:* Results from our survey indicate a strong need to study implementation strategies to increase the use and quality of safety planning in pediatric clinical care settings. Strategies could leverage behavioral nudge approaches, but also aim to increase clinician knowledge, skills, and competency, as well as organizational level climate and culture.

### S58 Equitable implementation of S.A.f.e. firearm: A multi-method pilot study

#### Katelin Hoskins

##### University of Pennsylvania School of Nursing, Philadelphia, PA, USA

###### **Correspondence:** Katelin Hoskins (hoskinsk@upenn.edu)

*Implementation Science 2023,* **18(Suppl 3):**S58

*Background:* Attention to health equity is critical in the implementation of suicide prevention efforts, particularly given rising rates of suicide among racially and ethnically minoritized youth. We present our operationalization of equity-oriented recommendations in preparation for launch of a hybrid effectiveness-implementation trial focused on firearm safety promotion in pediatric primary care as a universal suicide prevention strategy.

*Methods:* In Step 1 of our process, pre-trial engagement with stakeholders and a review of the literature alerted us that delivery of an evidence-based firearm safety program may vary by patients’ medical complexity, race, and ethnicity. In Step 2, we selected the Health Equity Implementation Framework to inform our understanding of contextual determinants (i.e., barriers and facilitators). In Step 3, we leveraged an implementation pilot across 5 pediatric primary care clinics in 2 health system sites to study signals of inequities. We analyzed EHR data with GEE logistic regression models and clinician interview data with rapid qualitative techniques. In Step 4, we interrogated equity considerations. In Step 5, we will develop a plan to monitor and mitigate potential inequities related to race and ethnicity and sex over the trial.

*Findings:* Eligible well-child visits for 694 patients and 47 clinicians were included in the analysis. Our results suggested that medical complexity was not associated with reach (OR 1.24, 95% CI [0.77, 2.01], *P*=0.38). Although the type III test for race and ethnicity was non-significant (*P*=0.196), the odds of documented reach differed between patients from NH-White and NH-Other groups (OR 1.72, 95% CI [1.02, 2.88], *P*=0.04). The odds of documented reach by all other race and ethnicity comparisons (e.g., Hispanic/Latino and NH-Black/AA) were non-significantly different. Though we did not initially plan to examine differences by sex, we learned that the odds of reach for females was 32.6% less than males (OR 0.67, 95% CI [0.47, 0.97], *P*=0.03). Seven qualitative interviews with pilot clinicians provided additional context.

*Implications for D&I Research:* Our innovative process demonstrates that prospective, rigorous, exploratory work is vital for equity-informed implementation trials. Implementation trials must focus on disaggregating implementation outcomes, like reach, across disadvantaged subgroups in order to understand distributional effects (i.e., who ultimately benefits from implementation).

**Primary Funding Source**

National Institutes of Health

### S59 Anticipating adaptation: tracking the impact of planned and unplanned adaptations during the implementation of a complex population-based genomic screening program

#### Caitlin Allen^1^, Daniel Judge^1^, Paul Nietert^1^, Amy Jackson^1^, Kelly Hunt^2^, Sam Gallegos^1^, Katherine Sterba^1^, Cathy Melvin^1^, Leslie Lenert^1^

##### ^1^Medical University of South Carolina, Charleston, SC, USA; ^2^Ralph H. Johnson VA Medical Center, Charleston, SC, USA

###### **Correspondence:** Caitlin Allen (allencat@musc.edu)

*Implementation Science 2023,* **18(Suppl 3):**S59

**Background:** Limited strategies exist to support tracking of adaptations made during program implementation; however, rigorous assessment of modifications can support responsive and evidence-informed enhancements. In this presentation we will: 1) Describe the impact of adaptations made during the implementation of a complex, large-scale population genomic screening intervention delivered in a learning health system on Reach and Implementation outcomes, and 2) Discuss methodological lessons-learned and recommendations for tracking, analyzing, and modifying programs based on real-time adaptation feedback.

**Methods:** We used a modified version of the Framework for Modification and Adaptations (FRAME) to code adaptations made during the three-month pilot phase of *In Our DNA SC*. Adaptations were documented in real-time using a REDCap database and we completed proactive and reactive coding using the RE-AIM framework. We conducted segmented linear regression models to test hypotheses about the impact of each adaptation on Reach (number of invitation recruitment messages viewed and number enrolled) and Implementation (number of DNA samples collected).

**Findings:** Ten adaptations were made during the three-month pilot phase. Forty percent of adaptations were planned as part of the program. The primary goal of the adaptations was to increase Reach to potential participants (60%). The nature of changes included adding a component (30%) to the intervention, condensing a component (20%), and tailoring to individuals (10%). Most adaptations were made based on knowledge and experience (40%) or from quality improvement data, summary information, or results (30%). Of the three adaptations designed to increase Reach, shortening the outreach message significantly increased the average rate of invitations viewed pre (32.7%) to post (42.0%) adaptation (p=0.0106). There was no effect of adaptations on Implementation (number of DNA samples collected).

**Implications for D&I Research:** Our approach to tracking adaptations of *In Our DNA SC* in real-time allowed our team to quantify the utility of modifications, make decisions about pursuing the adaptation, and understand the long-term consequences of the change. Streamlining tools for tracking and responding to adaptations can help plan and monitor the incremental impact of interventions to support continued learning and problem solving for complex interventions being delivered in health systems based on real-time data.

**Primary Funding Source**

K00CA253576

### S60 Applying learning health system strategies to cancer research: the citizen scientist cancer research curriculum

#### Janet Brishke, Zachary Jones, Elizabeth Shenkman

##### Department of Health Outcomes and Biomedical Informatics, College of Medicine, University of Florida, Gainesville, FL, USA

###### **Correspondence:** Janet Brishke (jbrishke@ufl.edu)

*Implementation Science 2023,* **18(Suppl 3):**S60

**Background:** A central tenet of the University of Florida Clinical and Translational Science Institute (UF CTSI) Learning Health System (LHS) Initiative is a focus on multidisciplinary teams. On these teams, community stakeholders, called Citizen Scientists (CSs), partner with researchers and clinicians to provide real-world perspectives on various research topics and projects. To better understand the content they were facing, CSs initiated and co-developed a curriculum about the fundamentals of clinical research. Following the success of that project, CSs have been integrated into research studies, implementation science oversight committees, and leadership teams at the CTSI.

**Methods:** The UF Health Cancer Center (UFHCC) recognized the strengths of this approach to research and sought to replicate it in its research programs. After gauging the needs of the CSs, researchers, and clinicians, the UFHCC used the ADDIE (Analysis, Design, Development, Implementation, Evaluation) instructional design model to create the CS Cancer Curriculum (CSCC). This course is a companion piece to the CTSI clinical research fundamentals course, and completing the CTSI course was a prerequisite for participating in the CSCC implementation pilot study. The CSCC focuses solely on cancer topics and consists of a mix of didactic lessons and supplemental videos, including a multi-part case study with a pediatric cancer survivor. The didactic lessons are guided by learning objectives, and each lesson contains an instructional video and learning assessment.

**Findings:** The average score for all nine CSs who completed the CSCC through the implementation pilot study was 95.6%. Eighty percent of the post-test respondents (n=8) felt confident in their ability to apply the course content to their work as a CS, and one responded that “The curriculum provided me with more information to become more effective in my advocacy as a stakeholder involved in cancer research.”

**Implications for D&I Research:** The partnership between CSs and researchers has helped the CTSI impact patient care more quickly, both in Florida and nationwide, through studies like ADAPTABLE and ACTIV-6. By extending LHS principles to cancer research, we may soon see an impact on patient care related to the second leading cause of death in the United States.

**Primary Funding Source**

Institutional funding- see description

### S61 Just ask: identifying barriers and facilitators to inform implementation strategies for smoking assessment and treatment in cancer care settings

#### Jamie Ostroff^1^, Lisa Allison^2^, Rob Adsit^3^, Daniel Boffa^4^, Jessica Burris^5^, Asa Carter^6^, Audrey Darville^7^, Michael Fiore^8^, Ellen Hahn^7^, James Harris^9^, Laurie Kirstein^10^, Danielle McCarthy^11^, Heidi Nelson^6^, Eileen Reilly^12^, Erin Reuter^13^, Rachel Shelton^14^, Sarah Shafir^15^, Elisa Tong^16^, Graham Warren^17^, Timothy Mullett^5^

##### ^1^Memorial Sloan Kettering Cancer Center, New York, USA; ^2^Commission On Cancer, Chicago, IL, USA; ^3^School of Medicine & Public Health, University of Wisconsin, Center for Tobacco Research and Intervention, Madison, WI, USA; ^4^Yale School of Medicine, New Haven, CT, USA; ^5^University of Kentucky, Markey Cancer Center, Lexington, KY, USA; ^6^American College of Surgeons, Commission on Cancer, Chicago, IL, USA; ^7^University of Kentucky, Lexington, KY, USA; ^8^School of Medicine and Public Health, University of Wisconsin, Center for Tobacco Research and Intervention, Madison, WI, USA; ^9^University of Nevada School of Medicine, Western Surgical Group, Reno, NV, USA; ^10^Memorial Sloan Kettering Cancer Center, New York, NY, USA; ^11^University of Wisconsin, Center for Tobacco Research and Intervention, Madison, WI, USA; ^12^American College of Surgeons, Commission On Cancer, Chicago, IL, USA; ^13^American College of Surgeons, Commission on Cancer, Chicago, USA; ^14^Columbia University Mailman School of Public Health, New York, NY, USA; ^15^American Cancer Society, Atlanta, GA, USA; ^16^Internal Medicine, University of California, Davis, Sacramento, CA, USA; ^17^Medical University of South Carolina, Charleston, SC, USA

###### **Correspondence:** Jamie Ostroff (ostroffj@mskcc.org)

*Implementation Science 2023,* **18(Suppl 3):**S61

**Background:** Smoking by cancer patients causes adverse outcomes and smoking cessation is associated with improved cancer and all-cause survival. Despite this, adoption of national clinical practice guidelines has been slow and inconsistent in cancer care settings.

**Methods:** Guided by an Implementation Mapping approach, the “Just ASK” project examines the context of smoking assessment and treatment in cancer care settings. The project was led by the American College of Surgeons Cancer Programs Commission Cancer (CoC) and the National Accreditation Program for Breast Centers (NAPBC) representing approximately 2,000 programs nationwide that provide cancer treatment for approximately 70% of all newly diagnosed cancer patients. All CoC/NAPBC programs were invited to participate in the “Just ASK” project, consisting of educational webinars, practical online resources, and three REDCap surveys. Program participation was incentivized to fulfill three annual accreditation standards for either the CoC or NAPBC. Surveys collect information on current smoking assessment and treatment practices, organizational priority, implementation barriers, and feasibility and effectiveness of potential implementation strategies. Baseline survey data are reported.

**Findings:** There were 776 participating programs; 731 representing CoC and 45 representing NAPBC. Programs reported strong endorsement of the importance of addressing smoking among cancer patients. The overwhelming majority of programs reported routinely assessing (91%) and documenting (87%) current smoking. However, 54% of programs could not extract data from the EMR needed to determine patients’ smoking prevalence. Additionally, routine delivery of evidence-based smoking cessation interventions (e.g., quitline referral, medication prescription, counseling services) was low (< 20%). Lack of staff training (70%), and lack of designated smoking cessation champions (63%) were the top two implementation barriers reported. Developing patient education materials was identified as the most feasible and staff education was identified as the most effective potential implementation strategies.

**Implications for D&I Research:** The high number of participating programs demonstrate strong national interest in addressing smoking among cancer patients. Baseline findings reveal key gaps in organizational capacity to obtain information about patients’ current smoking status, suggesting the need for systems-level strategies for improving patient assessment, clinical workflow and documentation. These findings highlight challenges and opportunities for implementing smoking assessment and treatment in cancer care settings.

### S62 New delivery models improve access to precision oncology for patients with advanced prostate cancer

#### Maren Scheuner^1^, Paloma Sales^1^, Katherine Hoggatt^1^, Samuel Washington^1^, Eva Ferino^1^, Christine Serway^2^, Sara Ahmed^2^, Michael Kelley^2^

##### ^1^San Francisco VA Health Care System, San Francisco, CA, USA; ^2^National Oncology Program Office Washington, DC, USA

###### **Correspondence:** Maren Scheuner (maren.scheuner@va.gov)

*Implementation Science 2023,* **18(Suppl 3):**S62

**Background**

The Department of Veterans Affairs (VA) launched a clinical pathway including both tumor and germline testing to inform targeted treatment for advanced prostate cancer on 5/3/2021. Anticipating increased germline testing demand, new delivery models for germline testing were created to augment the existing traditional model of referring patients to genetics providers. This included a non-traditional model where oncologists perform all pre- and post-test activities and consult genetics when needed, and a hybrid model where oncologists obtain informed consent and place genetic consults for germline test ordering, results disclosure, and if needed, genetics follow-up. We sought to assess germline testing by delivery model.

**Methods**

Data sources included the VA National Precision Oncology Program dashboard and reports from contracted germline testing laboratories. Patient inclusion criteria: living as of 5/2/2021 with VA oncology visits after 5/2/2021. Multivariate regression assessed associations between patient characteristics and germline testing between 5/3/2021 (pathway launch) and 5/2/2022, accounting for clustering of patients within ordering clinicians.

**Findings**

We identified 16,041 patients with advanced prostate cancer from 129 VA facilities with average age 75 (SD=8.2,36-102), 29% Black, and 60% White. Throughout the first year, 896 (6%) patients had germline testing ordered by 60 clinicians at 67 facilities with 52% of orders by the hybrid model, 32% the non-traditional model, and 16% the traditional model. Patient characteristics positively associated with germline testing included receiving care at hybrid model (OR=6.03,95%CI:4.62-7.88) or non-traditional model facilities (OR=5.66,95%CI:4.24-7.56) compared with the traditional model, completing tumor molecular testing (OR=5.80,95%CI:4.98-6.75), and Black compared with White race (OR=1.24,95%CI:1.06-1.45). Compared to patients aged <66, patients aged 66-75 and 76-85 were less likely to have germline testing (OR=0.74,95%CI:0.60-0.90 and OR=0.67,95%CI:0.53-0.84, respectively).

**Implications for D&I Research**

Though only 6% of advanced prostate cancer patients had germline testing since pathway launch, the new delivery models were instrumental to improving access. Evaluation is ongoing to understand observed demographic differences in germline testing and effectiveness of implementation strategies to promote adoption of the new delivery models. Continued spread and uptake of the hybrid and non-traditional models for germline testing should result in improved outcomes for patients by increasing use of treatments targeting molecular findings and informing clinical trials eligibility.

**Primary Funding Source**

Department of Veterans Affairs

### S63 Evaluating progress and updating the learning health systems scientific and engineering research agenda

#### Joshua Rubin

##### Joseph H. Kanter Family Foundation, Miami, FL, USA

###### **Correspondence:** Joshua Rubin (josh@joshcrubin.com)

*Implementation Science 2023,* **18(Suppl 3):**S63

**Background:** In 2012, the Joseph H. Kanter Family Foundation sponsored the inaugural multi-stakeholder Learning Health System (LHS) Summit that helped catalyze an incipient global grassroots LHSs movement anchored in shared consensus *Core Values* for LHSs. One outcome of that meeting was a realization that a transdisciplinary science underpinning LHSs needed development.

**Methods:** In 2013, the National Science Foundation (NSF), one of the participants in the LHS Summit, convened an invitational workshop to identify the fundamental scientific and engineering research challenges to achieving LHSs at large scales. The 45 workshop participants, representing prominent researchers spanning diverse disciplines, collectively identified 106 research questions organized around four system-level requirements that a high-functioning LHS must satisfy. The workshop participants also identified a new cross-disciplinary integrative science of cyber-social learning systems (CSLSs) that will be required to address these challenges. The results were published in a peer reviewed paper in late 2014.

**Findings:** The resulting research agenda for LHSs underpinned subsequent transdisciplinary workshops on LHSs and CSLSs funded by NSF and the Computing Community Consortium (CCC) in Hawaii, Michigan, Washington state, and Washington, DC, as well as synergistic working meetings and collaborative efforts spanning several continents worldwide. This work also informed the foundations of a first-of-its-kind academic Department of Learning Health Sciences, a novel peer reviewed scientific journal of *Learning Health Systems*, and further developments inside and outside of academic medicine.

**Implications for D&I Research:** The aforementioned consensus *Core Values* for LHSs informed federal health IT strategic planning calling for the realization of a nationwide LHS by 2024. With public and private LHSs initiatives worldwide, the COVID-19 global pandemic illuminated further the need to take this vision global; data, knowledge, and learning needed to combat public health threats must move faster and more freely than diseases themselves. As 2024 approaches, these and related developments call for an assessment of progress addressing the LHSs research challenges identified a decade prior as well as an update of the components of this research agenda. These developments also call for reassessing progress on a complementary *LHSs Consensus Action Plan* and on a related framework for integrating health equity into LHSs.

### S64 Identifying requisite learning health system competencies: a scoping review

#### Paige McDonald

##### The George Washington University School of Medicine and Health Sciences, Washington, DC, USA

###### **Correspondence:** Paige McDonald (paigem@gwu.edu)

*Implementation Science 2023,* **18(Suppl 3):**S64

**Background:** Learning health systems (LHS) integrate knowledge and practice through cycles of continuous quality improvement and learning to increase healthcare quality. LHS have been conceptualized through multiple frameworks and models. Our aim was to identify and describe the requisite individual competencies (knowledge, skills, and attitudes) and system competencies (capacities, characteristics, and capabilities) described in existing literature in relation to operationalizing LHS.

**Methods:** A scoping review was conducted with descriptive and thematic analysis to identify and map competencies of LHS for individuals/patients, health system workers, and systems. Articles until April 2020 were included based on a systematic literature search and selection process. Themes were developed utilizing a consensus process until agreement was reached among team members.

**Findings:** Eighty-nine articles were included with most studies conducted in the United States (68 articles). The largest number of publications represented competencies at the system level, followed by health system worker competencies. Themes identified at the individual/patient level were knowledge and skills to understand and share information with an established system and the ability to interact with the technology used to collect data. Themes at the health system worker level were skills in evidence-based practice, leadership and teamwork skills, analytical and technological skills required to use a "digital ecosystem," data-science knowledge and skill, and self-reflective capacity. Researchers embedded within LHS require a specific set of competencies. Themes identified at the system level were data, infrastructure, and standardization; integration of data and workflow; and culture and climate supporting ongoing learning.

**Implications for D&I Research:** The identified individual stakeholder competencies within LHS and the system capabilities of LHS provide a solid base for the further development and evaluation of LHS. International collaboration for stimulating LHS will assist in further establishing the knowledge base for LHS, thereby increasing the responsiveness of health care systems.

### S65 Case example of a quality improvement program in a lhs: using feedback on patient health outcomes to improve orthopedic physical therapist practice

#### Philip Van der Wees

##### George Washington University, Washington, DC, USA

###### **Correspondence:** Philip Van der Wees (vanderwees@gwu.edu)

*Implementation Science 2023,* **18(Suppl 3):**S65

**Background:** Measuring health outcomes can play an important role in patient-centered healthcare. When aggregated across patients, outcomes provide data for quality improvement (QI). However, most physical therapists are not familiar with QI methods based on patient outcomes. We aimed to develop and evaluate a QI program in outpatient physical therapy care based on routinely collected health outcomes of patients with low-back pain and neck pain**.**

**Methods:** The QI program was conducted within the network of Clinical Rehab Services (CRS) from the University of Pittsburgh Medical Center (UPMC). UPMC has a large rehabilitation network in western Pennsylvania in which health outcomes are collected in a joint database. Three teams of 5-6 physical therapists from outpatient settings used PDSA cycles for a QI project based on self-selected goals. Monthly feedback reports of process and outcomes of care guided the QI efforts

**Findings:** The QI program was feasible and well accepted by pilot teams of physical therapists. Clinician active participation in developing the QI program supported the use, feasibility, and value of outcome measurement for clinical practice. The QI program improved the use of outcome measures, allowing for comparing treatment outcomes over time within and between practices.

**Implications for D&I Research:** The results are promising for further evaluation and implementation of using treatment outcomes for QI efforts in physical therapist practice.

**Primary Funding Source**

CoHSTAR pilot study grant

### S66 The learning health system toolkit: developing a toolkit for knowledge translation and implementation toward more responsive systems of care

#### Sarah Gilman

##### The George Washington University, Washington, DC, USA

###### **Correspondence:** Sarah Gilman (sarahgilman@gwmail.gwu.edu)

*Implementation Science 2023,* **18(Suppl 3):**S66

**Background:** A learning health system (LHS) strives to continually improve outcomes and experience through the application of science, informatics, incentives, and culture to create and use knowledge in care delivery^1^. The LHS toolkit aims to enable knowledge-sharing and communities of practice around LHS by providing guidance and tools for developing, implementing, and sustaining LHSs. This abstract details the development process for the toolkit, the resulting toolkit structure, and the intended next steps for piloting and dissemination.

**Methods:** An international steering committee of 12 individuals in the field of LHS and quality improvement guided toolkit development. Adopting a team-based, interdisciplinary approach, members of the committee provided recommendations on the first 70 tools added, including resources from areas such as implementation science, quality improvement, knowledge translation, complexity science, value-based health care, informatics, and team science. Inclusion criteria were developed for toolkit content and tools eligible for inclusion were added. Tools were drawn from peer-reviewed articles, case studies, organizational websites, and methods papers. Steering committee members made recommendations for improvement on the initial version through a web-based survey and several rounds of feedback in steering committee meetings

**Findings:** The LHS toolkit has three primary sections: 1) "Find a tool" helps users identify tools for developing and implementing LHS, tailored to stage in the LHS process; 2) "All tools" for users who want a searchable list of all tools available; and 3) "Submit a tool," in which users can contribute to the learning community by submitting a tool for inclusion in the toolkit. Next steps include piloting the tool with a broader audience, continuing to add resources and make improvements, and developing a dissemination plan.

**Implications for D&I Research:** Based on an interdisciplinary, team science approach, the LHS toolkit is designed to amplify and accelerate the positive impact of LHS by sustaining a community of practice aimed toward building more proactive systems of care that are responsive to the diverse needs of systems and populations.

**Reference**

1. Foley T. Realising the potential of - learning healthcare project. https://learninghealthcareproject.org/wp-content/uploads/2021/05/LHS2021report.pdf. Accessed July 19, 2022.

### S67 Using the vha diffusion marketplace to track the spread of innovations across the veterans health administration

#### George Jackson^1,2^, Madison Burns^3^, Blake Henderson^4^, Alex Seelig^5^, Brandolyn White^3^, Jennifer Lindquist^3^, Gemmae Fix^6,7^, Andrea Nevedal^8^, Joshua Drumm^5^, Brad Johnson^5^, Kathryn DeLaughter^6^, Caitlin Reardon^8^, Sarah Cutrona^9,10^, Laura Damschroder^8^, Marilla Widerquist^8^, Maria Arasim^8^, Allen Gifford^7,11^, Blaine Fitzgerald^4^

##### ^1^Durham Veterans Affairs Health Care System, Durham, NC, USA; ^2^Duke University, Durham, NC, USA; ^3^Durham VA Health Care System, Durham, NC, USA; ^4^VHA Innovation Ecosystem, Washington, DC, USA; ^5^Agile Six Applications, Inc., San Diego, CA, USA; ^6^VA Bedford Healthcare System, Bedford, MA, USA; ^7^Boston University, Boston, MA, USA; ^8^VA Ann Arbor Healthcare System, Ann Arbor, MI, USA; ^9^Center for Healthcare Organization and Implementation Research (CHOIR), VA Bedford Healthcare System, Bedford, MA, USA; ^10^University of Massachusetts Chan Medical School, Worcester, MA, USA; ^11^VA Boston Healthcare System, Boston, MA, USA

###### **Correspondence:** George Jackson (george.l.jackson@duke.edu)

*Implementation Science 2023,* **18(Suppl 3):**S67

**Background:** The Veterans Health Administration (VHA) has >1,200 sites of care organized in 141 individual parent/component facilities/healthcare systems. Developed by the VHA Innovation Ecosystem to encourage spread of innovative clinical and administrative practices, the VHA Diffusion Marketplace is a discovery and collaboration tool that allows for the sharing of information about successful healthcare innovations and tracking of their spread across the nationwide VHA. We present data on the concentration of adopting innovations across the VHA.

**Methods:** The Diffusion Marketplace (https://marketplace.va.gov/) was launched internally to the VHA in February 2020 and made available publicly in October 2021. Searchable by topic, curated promising clinical, operational, and strategic innovations that have been successfully piloted or implemented in one or more VHA facilities are submitted from the VHA Innovation Ecosystem and other VHA sources, such as the VHA Quality Enhancement Research Initiative. Key factors related to innovation objectives, core and adaptable components, implementation process, and evidence of impact are provided. This provides a tool to record adoption of fully implemented, in progress, and unsuccessful adoption of innovations. We report the concentration of recorded innovation adoptions (fully implemented and in-process) among 141 parent facilities/healthcare systems, included through the calculation of a Geni coefficient indicating the degree to which innovation adoption is evenly distributed across the VHA.

**Findings:** The Diffusion Marketplace has 184 published innovations and has had 54,754 page views since its inception. There are 3,375 recorded adoptions among 133 of 141 parent facilities/health systems (mean per facility = 23.9 for all facilities; 25.4 for facilities reporting adoptions). While more complex facilities tend to have more innovation adoptions, qualitatively, there is broad participation in innovation adoption, with innovations spread widely across the nationwide VHA. The top 10 facilities (i.e. 7% of facilities) had 481/3,375 adoptions (14%) and top 20 facilities (i.e. 14% of facilities) had 942/3,375 adoptions (28%). The Geni coefficient is 0.27, indicating a reasonably, but not completely, even distribution of innovation adoptions across the VHA.

**Implications for D&I Research:** The VHA Diffusion Marketplace is a scalable tool that is being used to address the implementation challenge of tracking a considerable number of innovations/healthcare practices spread across large healthcare systems.

**Primary Funding Source**

Department of Veterans Affairs

### S68 Creating a relational playbook for cardiology teams to enhance employee well-being and veteran care

#### Heather Gilmartin^1,2^, Brigid Connelly^1^, Edward Hess^1^, Plomondon Meg^3^, Stephen Waldo^3^, Catherine Battaglia^1,2^

##### ^1^Denver/Seattle Center of Innovation, Aurora, CO, USA; ^2^Rocky Mountain Regional VA Medical Center, Washington, DC, DC, USA; ^3^CART Program, VA Office of Quality & Patient Safety, Washington, D.C., DC, USA

###### **Correspondence:** Heather Gilmartin (heather.gilmartin@va.gov)

*Implementation Science 2023,* **18(Suppl 3):**S68

**Background**

The Veterans Health Administration (VA) set a goal to become a learning health system and high reliability organization (LHS-HRO) to enhance employee well-being by delivering high-quality, equitable care and creating a learning culture. Learning environments are the educational approaches and settings which learning happens and are fundamental to LHS-HROs. Supportive learning environments (SLEs) empower teams to trial and adapt innovations while using highly-reliable practices (e.g., checklists) to ensure patient safety. It is unknown if SLEs exist in the VA and what strategies create SLEs within teams. We partnered with the 81 VA cardiac catheterization labs (CCLs) to identify SLEs, assess their creation, the impact on staff, and the evidence-based strategies in use. These data and the literature will inform the creation of a Relational Playbook of customized tools to spread SLE best practices across CCLs.

**Methods**

Longitudinal, sequential, mixed-methods design, guided by LHS-HRO frameworks. The Learning Environment Survey was administered to VA staff in 2018 and 2020. Linear regression and Bayesian models ranked CCLs and characterized relationships between learning environments and employee engagement, retention, and safety climate. Staff from high-ranking CCLs were interviewed. Data were analyzed using rapid, qualitative analysis. The Playbook was developed from these data and literature reviews. Feedback, acceptability, appropriateness, and feasibility ratings (1-5 ascending Likert scale) were requested from an expert review panel.

**Findings**

The 2018 survey (N=296; 68 CCLs; 84% response rate) detected national and CCL level (N = 29) variation that persisted through 2020 (N=231; 67 CCLs; 83%). SLEs were associated with higher employee engagement, retention, and safety climate. Interviews with 13 staff from 6 CCLs revealed five LHS-HRO concepts to create SLEs: positive culture, build a team, lead the team, joy in work, communication, and high-reliability practices. The Playbook rated high for acceptability (4.37/5), appropriateness (4.28/5) and feasibility (3.94/5).

**Implications for D&I Research**

The identification of SLEs and the strategies used by VA CCLs informed a Relational Playbook training tool designed by and for CCL teams that will be tested and evaluated in a pragmatic implementation trial to understand the impact of SLEs on employee well-being and Veteran care prior to national spread and scale-up.

**Primary Funding Source**

Department of Veterans Affairs

### S69 Barriers and enablers to implementing peer specialists in veterans health administration primary care: The role of external facilitation

#### Amanda Peeples^1^, Anjana Muralidharan^3^, Sharon McCarthy^2,4^, Karin Daniels^5^, Richard Goldberg^3^, Lorrianne Kuykendall^3^, Natalie Vineyard^3^, Matthew Chinman^5^

##### ^1^Center for Health Equity Research and Promotion (CHERP), Michael J. Crescenz VAMC, Philadelphia, PA Baltimore, MD, USA; ^2^Center for Health Equity Research and Promotion, Pittsburgh, PA, USA; ^3^VISN 5 Mental Illness Research, Education and Clinical Center (MIRECC) Baltimore, MD, USA; ^4^VISN 4 Mental Illness Research, Education and Clinical Center (MIRECC) Pittsburgh, PA, USA; ^5^Center for Health Equity Research and Promotion, Pittsburgh, PA, USA

###### **Correspondence:** Amanda Peeples (amanda.peeples@va.gov)

*Implementation Science 2023,* **18(Suppl 3):**S69

**Background**

The Veterans Health Administration (VHA) employs over 1100 Peer Specialists (PSs), mostly in behavioral health. PSs are Veterans with lived experience of recovery in mental illness who are trained to use their experience to help other Veterans. In response to a 2014 White House Executive Action, two PSs were reassigned to primary care at 25 VHA sites. A mixed-methods evaluation, guided by the integrated Promoting Action on Research Implementation in Health Services (i-PARIHS) framework, was undertaken to assess the impact of external facilitation provided to 12 sites compared to 13 control sites who implemented PSs on their own, and to identify the barriers and enablers encountered.

**Methods**

Administrative data from 25 participating sites was analyzed to characterize the level and reach of PS services delivered. Rapid analysis of qualitative interviews with 25 PSs and 27 of their supervisors was conducted to identify barriers and enablers to PS integration, as well as to examine the role of external facilitation in implementation experiences.

**Findings**

Quantitative analyses found that sites receiving external facilitation had significantly shorter time starting PS service delivery and more unique Veterans served and higher number of PS visits in the first year. Qualitative analysis identified ten themes and twelve subthemes as barriers and enablers, organized by three i-PARIHS framework constructs: INNOVATION – themes PS role clarity, PS role constraints (subthemes: co-location, workload, autonomy), and (mis)match of services needed/offered; RECIPIENTS – providers (buy-in, burden), PSs (individual qualities, skills), supervisors (burden, relationship with PSs), Veterans (engagement), and champions; and CONTEXT – support (leadership, funding) and primary care structure/culture. Barriers and enablers were consistent across external facilitation and control sites, yet qualitative and quantitative results indicate that external facilitation sites may have been able to overcome barriers, and shore up enablers, more efficiently than control sites to improve PS integration and services.

**Implications for D&I Research**

Using i-PARIHS, results describe how the characteristics of the innovation, recipients, and context impact successful implementation of PSs in primary care settings. The identification of barriers and enablers, as well as indications that external facilitation was a useful implementation strategy, holds promise for improving future efforts to embed PSs in primary care.

**Primary Funding Source**

Department of Veterans Affairs

### S70 Dynamic interplay between available resources and implementation climate across phases of implementation: a qualitative study of a va national population health tool

#### Ying-Jen Lin^1^, Allison A. Ranusch^2^, F. Jacob Seagull^1^, Jeremy Sussman^1,2^, Geoffrey Barnes^1^

##### ^1^University of Michigan, Ann Arbor, MI, USA; ^2^VA Ann Arbor Healthcare System, Ann Arbor, MI, USA

###### **Correspondence:** Geoffrey Barnes (gbarnes@med.umich.edu)

*Implementation Science 2023,* **18(Suppl 3):**S70

**Background**

Resource availability can impact the success of an intervention. However, few studies have investigated how the resources required for implementation may change over time. Using stakeholder interviews from a national population health tool implementation effort, we examined the changes in and interactions between available resources and implementation climate in the early and late phases of a nationwide implementation effort.

**Methods**

We interviewed 20 anticoagulation professionals at 17 clinical sites in the VA health system about their experiences with a population health dashboard for anticoagulant management. Interview transcripts were coded using CFIR constructs and according to the phase of implementation (Pre-implementation, Implementation, and Sustainment) as defined by the VA QUERI Roadmap. We analyzed the co-occurrence patterns between *available resources* and *implementation climate* across different phases. To illustrate the variations in determinants across phases, we aggregated and scored coded statements using a previously published CFIR scoring system (-2 to +2). The key relationships between *available resources* and *implementation climate* were identified and summarized using thematic analysis.

**Findings**

The resources necessary to support successful implementation of an intervention are not static; instead, both the quantity and types of resources shift based on the phases of the intervention. Increased resource availability does not guarantee the sustainment of intervention success. Users need different types of support that go beyond the technical aspects of an intervention and vary over time. Specifically, resources in the form of technological support and social/emotional support help users establish trust in a new technological-based intervention during the implementation phase. Resources that foster and maintain collaboration between users and other stakeholders help them stay motivated during sustainment.

**Implications for D&I Research**

Our findings highlight the dynamic nature of *available resources* and their impacts on the success across different phases of implementation. A better understanding of the dynamics of *available resources* over time from the users’ perspectives will allow adaptation of the resources to better meet the needs of the intervention stakeholders.

**Primary Funding Source**

Agency for Healthcare Research and Quality

### S71 The influence of outer setting systems-level characteristics on wraparound care coordination implementation processes

#### Jonathan Olson^1^, Kimberly Estep^2^, Kimberly Coviello^2^, Denise Sulzbach^2^, Eric Bruns^3^

##### ^1^University of Washington, Seattle, WA, USA; ^2^University of Maryland, Baltimore, MD, USA; ^3^UW SMART Center, UW SMART Center, Seattle, WA, USA

###### **Correspondence:** Jonathan Olson (jro10@uw.edu)

*Implementation Science 2023,* **18(Suppl 3):**S71

**Background**

Recent data suggest that states using Care Management Entities (CMEs) to implement and administer Wraparound care coordination demonstrate higher levels of implementation fidelity and shorter times to full implementation than states relying on traditional Community Mental Health Centers (CMHCs). Such findings underscore the impact that system-level fiscal and administrative structures and associated organization-level practices have on Wraparound implementation. The purpose of this study was to identify implementation processes and tasks associated with implementation completion and duration within CMEs and CMHCs using the Stages of Implementation Completion (SIC).

**Methods**

Wraparound implementation processes in 8 states (4 CME; 4 CMHC) were tracked across 8 SIC stages. We calculated two scores: 1) Proportion of tasks completed within each stage; and 2) Time to completion. We compared SIC scores across administrative structures and to national SIC means for other EBPs. Finally, we conducted an item-level analysis to identify tasks with the longest completion times.

**Findings**

Nearly 100% of SIC tasks were completed across the first 5 stages for CME and CMHC structures, although completion rates declined during stages 6 – 8 among CMHCs. CMEs were faster to completion in all but the first stage (*d* > .77). Compared to a national sample of EBPs, Wraparound implementation took nearly twice as long in CME states and nearly three times as long in CMHC states. Item-level analyses suggest that CMHCs took more time than CMEs to complete systems-level tasks: Setting fiscal structures, agreeing on populations of focus, and establishing external messaging to engage stakeholders; and organization-level tasks: Establishing skill-building expectations, beginning staff training, and engaging in fidelity reviews (*d* > .78).

**Implications for D&I Research**

Although states employing CME structures were more efficient than CMHCs in completing implementation processes, both took considerably longer to implement Wraparound when compared to other types of EBPs. Such findings suggest that “outer setting,” systems-level coordination that characterizes Wraparound takes time to fully implement. Item-level results suggest that future implementation support should target systems-level processes such as establishing fiscal structures, and organization-level processes such as training, staff recruitment and retention, and fidelity monitoring.

### S72 How inner setting factors relate to high fidelity implementation of wraparound care coordination

#### Tony Bonadio^1^, Kimberly Coviello^2^, Kimberly Estep^2^, Jonathan Olson^3^, Eric Bruns^4^

##### ^1^University of Maryland School of Social Work, Baltimore, MD, USA; ^2^University of Maryland, Baltimore, MD, USA; ^3^University of Washington, Seattle, WA, USA; ^4^UW SMART Center, UW SMART Center, Seattle, WA, USA

###### **Correspondence:** Tony Bonadio (fbonadio@ssw.umaryland.edu)

*Implementation Science 2023,* **18(Suppl 3):**S72

**Background**

Wraparound care coordination has been shown to improve outcomes for youth and families with complex needs and multi-system involvement. However, a recent meta-analysis of 17 controlled studies shows that implementing Wraparound with adherence to the prescribed model, while challenging, is associated with more positive youth outcomes. Experience supporting Wraparound implementation in states and systems of care nationally point to the importance of organizational or “inner setting” factors to achieving “high-fidelity” Wraparound. To support data-driven coaching and technical assistance, the National Wraparound Implementation Center (NWIC) developed a pragmatic measure of inner setting factors that impact Wraparound implementation. The current study used data from this measure to explore the hypothesis that inner setting factors are associated with Wraparound fidelity.

**Methods**

Inner setting and fidelity data were collected from 15 organizations implementing Wraparound. Fidelity was assessed using the Wraparound Fidelity Index-Short Form (WFI-EZ), a self-report measure of adherence to the Wraparound model completed by caregivers. Inner setting factors were assessed with the Wraparound Implementation Standards for Programs (WISP), a pragmatic instrument completed by NWIC coaches to guide technical assistance. The WISP includes several subscales across organizational leadership, supervisors, and staff factors.

**Findings**

Despite the small sample size, results indicated that fidelity was correlated with three inner setting factors assessed by the WISP: Organizational Leadership (*r*=0.51, *p*=.054), Staff Satisfaction (*r*=0.51, *p*=.052), and Care Coordinator Engagement (*r*=0.52, *p*=.049). Organization Leadership was significantly correlated with Care Coordinator Engagement (*r*=0.59, *p*=.022), but neither of these factors were correlated with Staff Satisfaction.

**Implications for D&I Research**

These trends suggest that Wraparound implementation is improved when organizational leaders translate Wraparound philosophy in policies and engage supervisors and staff in conversation around planning and addressing barriers of implementation. Additionally, results suggest that monitoring and addressing staff satisfaction may by key targets for improving implementation outcomes. These provide targets for data-driven coaching and technical assistance that could be continuously monitoring using this pragmatic measure.

### S73 Proactive use and collection of data around inner and outer setting constructs enhance wraparound installation efforts

#### Kimberly Estep^1^, Kimberly Coviello^1^, Denise Sulzbach^1^, Tony Bonadio^2^

##### ^1^University of Maryland, Baltimore, MD, USA; ^2^University of Maryland School of Social Work, Baltimore, MD, USA

###### **Correspondence:** Kimberly Estep (kestep@ssw.umaryland.edu)

*Implementation Science 2023,* **18(Suppl 3):**S73

**Background**

As states, organizations, and providers work under increasingly resource-constrained conditions, purposeful and explicit use of evidence-informed implementation strategies are essential to ensure that practice installation efforts maximize resources and improve supports and services for children, youth, young adults, and their families. While installation of a practice model requires staff to build skill around the elements of the practice itself, successfully installing a practice requires more than attention to practice/process elements. Practice experts/coaches supporting installation of any practice must understand the broader context in which the practice/process is being installed and implemented. Outer and inner setting constructs are decision support factors that inform coaching approaches and to whom coaching is focused. Coaches need to be able to assess the situation and develop responses that fit the complexity of the installation effort.

**Methods**

Data collected from the Wraparound adapted Stages of Implementation Completion (Wrap-SIC) and the Wrapround Implementation Standards – Program (WISP) were used to inform and target coaching sessions that included discussing and reviewing implementation experiences and challenges with implementing staff as well as organization and state leadership.

**Findings**

Use of the Wrap-SIC and the WISP at the start of an initiative supported targeted and proactive planning and technical assistance around factors shown to positively impact Wraparound installation. Ongoing review and prioritization of the outlined indicators of both tools allowed expert coaches to target their TA to those items that were shown to have the greatest impact on youth and family outcomes and practice sustainability.

**Implications for D&I Research**

Proactive use of tools designed to target outer and inner settings positively supported staff’s ability to implement practices with higher quality and fidelity. Expert coaches can collect data across settings, strategically address indicators from the onset of the TA partnership, and track progress over time.

### S74 Implementing intimate partner violence screening programs in vha primary care clinics: implementation and effectiveness outcomes from a hybrid ii trial

#### Christopher Miller^1^, Kelly Stolzmann^2^, Julianne Brady^2^, Omonyele Adjognon^3^, Melissa Dichter^4^, Galina Portnoy^5^, Megan Gerber^6^, Samina Iqbal^7^, Sally Haskell^8^, Katherine Iverson^9^

##### ^1^Harvard Medical School, Boston, MA, USA; ^2^VA Boston Healthcare System, Boston, MA, USA; ^3^CHOIR, VA Boston Healthcare System, Boston, MA, USA; ^4^Associate Professor, Temple University, Philadelphia, PA, USA; ^5^VA Connecticut Healthcare System West Haven, CT, USA; ^6^Albany Medical College, Albany, NY, USA; ^7^VA Palo Alto Healthcare System, Palo Alto, CA, USA; ^8^VA Connecticut Healthcare System, West Haven, CT, USA; ^9^National Center for PTSD, VA Boston Healthcare System, Boston, MA, USA

###### **Correspondence:** Christopher Miller (christopher.miller8@va.gov)

*Implementation Science 2023,* **18(Suppl 3):**S74

**Background:** Intimate partner violence (IPV) is a population health problem that disproportionately impacts women veterans. The Veterans Health Administration (VHA) recommends evidence-based IPV screening in primary care to identify women who may benefit from support services. We partnered with VHA operational offices to evaluate implementation facilitation (IF) to enhance the uptake of IPV screening programs in primary care clinics. This investigation examines IF’s impact on implementation (i.e., reach of IPV screening) and clinical effectiveness (i.e., disclosure rates and post-screening psychosocial service use) outcomes.

**Methods:** A cluster randomized, stepped wedge, hybrid-II design compared IF to implementation as usual, with nine VHA sites participating in two study waves. IF included an external facilitator working with a facility-funded internal facilitator within participating primary care clinics for six months. Using RE-AIM to guide analyses, we examined medical records to identify changes in reach (i.e., IPV screening rates) and effectiveness (i.e., disclosure rates and post-screening psychosocial service use) associated with IF. All women receiving care at participating clinics in the 3 months prior to IF (pre-IF period; n=2,272) and 9 months following the start of IF (IF period; n=5,149) were included in generalized estimating equation (GEE) analyses. An Institutional Review Board approved this study.

**Findings:** In terms of reach, women seen during the IF period were nearly 3 times more likely to be screened for IPV compared to the pre-IF period (OR=2.70; p<.0001). Regarding disclosure, among all screened women, those screened during the IF period were not more likely to disclose IPV compared to those screened during the pre-IF period (OR=1.14; p=.36). However, among all eligible women, those seen during the IF period were more likely to disclose IPV compared to those seen in the pre-IF period (p<.0001). Additionally, among all screened women, those screened during the IF period were more likely to receive psychosocial services within 60 days post-screen than the pre-IF screened women, adjusting for pre-screening psychosocial service use (OR=1.29; p=.01).

**Implications for D&I Research:** This study demonstrates the value of operations-led IF to increase the reach of IPV screening programs in busy primary care clinics. These efforts increased detection of IPV and strengthened connections to potentially life-saving support services.

**Primary Funding Source**

Department of Veterans Affairs

### S75 Examining multi-level stakeholder perspectives of barriers and facilitators to implementing an evidence-based community health worker model

#### Simone Schriger^1^, Molly Knowles^2^, Shreya Kangovi^3^, Rinad Beidas^2^

##### ^1^University of Pennsylvania, Philadelphia, PA, USA; ^2^Perelman School of Medicine at the University of Pennsylvania, Philadelphia, PA, USA; ^3^Perelman School of Medicine - Penn Center for Community Health Workers, Philadelphia, PA, USA

###### **Correspondence:** Simone Schriger (schriger@sas.upenn.edu)

*Implementation Science 2023,* **18(Suppl 3):**S75

**Background:** Community health worker (CHW) programs are socio-behavioral interventions designed to address structural and social determinants of health that have been proven to improve health outcomes and reduce cost of care. The IMPaCT (Individualized Management of Patient-Centered Targets) CHW model has been tested in multiple randomized trials and shown to improve health outcomes, increase quality of life, and reduce hospitalizations, while also exhibiting persistence of effect and a 2.47:1 return on investment within the fiscal year. However, knowledge is limited on how to best scale evidence-based CHW models in new health systems and of stakeholder perspectives on implementation barriers and facilitators.

**Methods:** In this qualitative study, we assessed multi-level stakeholder perspectives on implementing the IMPaCT CHW model at five geographically distinct health systems. We carried out 39 semi-structured interviews with system leaders (n=11), CHW supervisors (n=4), CHWs (n=12) and patients (n=12), querying around domains from the Consolidated Framework for Implementation Research (CFIR). Interviews were transcribed and coded using a rapid qualitative analytic technique to identify key themes within and across stakeholder groups. Themes were categorized into barriers and facilitators associated with CFIR domains: intervention characteristics, inner setting, outer setting, and implementation process.

**Findings:** Overarching barriers across stakeholder groups included difficulties with clinical integration (particularly in larger health systems), resource limitations (often exacerbated by COVID-19), program cost, logistical issues associated with documentation, limited mental health training, challenges associated with meeting needs of undocumented and/or non-English speaking patients, and unsupportive system leadership. Overarching facilitators included strong empirical evidence for the model, extensive time and individualized attention given to patients, comprehensive training and implementation support, support from larger state bodies, adaptability of the model in various settings and patient populations, and strong CHW passion and motivation.

**Implications for D&I Research:** Insights gleaned from stakeholders will inform design and implementation of future CHW programs and aid in the development of best practices in CHW program implementation. Future D&I research can leverage multi-level stakeholder input to bolster facilitators and reduce barriers. These findings can support the design and testing of implementation strategies to support widespread implementation and sustainment of CHW programs nationally and internationally to increase health equity and improve population health.

**Primary Funding Source**

Patient-Centered Outcomes Research Institute

### S76 Cost and activity analysis for a city wide patient navigation program to engage underserved patients in breast cancer treatment: findings from the translating research into practice (trip) study

#### Serena Rajabiun^1^, Victoria Xiao^2^, Sharon Bak^3^, Clara Chen^4^, Maha Ashraf^3^, Erika Christenson^3^, Howard Cabral^5^, Rachel Freedman^6^, Stephenie Lemon^7^, Jennifer Haas^8^, Karen Freund^9^, Tracy Battaglia^2^

##### ^1^University of Massachusetts, Lowell, Lowell, MA, USA; ^2^Boston University School of Medicine, Boston, MA, USA; ^3^Boston Medical Center, Boston, MA, USA; ^4^Boston University School of Public Health, Boston, MA, USA; ^5^Boston University School of Public Health, Boston, USA; ^6^Dana Farber Cancer Institute, Boston, MA, USA; ^7^University of Massachusetts Chan Medical School, Worcester, MA, USA; ^8^Harvard Medical School, Boston, MA, USA; ^9^Tufts Medical Center, Boston, MA, USA

###### **Correspondence:** Serena Rajabiun (serena_rajabiun@uml.edu)

*Implementation Science 2023,* **18(Suppl 3):**S76

**Background:** There is a paucity of economic evaluations of cancer patient navigation programs, and even fewer published methods for estimating costs. Given variability in navigation programs with respect to staff background, roles and scope of services, economic analyses are needed to inform reimbursement, replication and sustainability.

**Methods:** Using participatory methods and a micro-costing approach, each site completed two data collection tools to capture costs and activities associated with patient navigation in a multi-site implementation study of newly diagnosed breast cancer patients. A time motion survey, completed by navigators and their respective supervisor collected navigation activities over 10 consecutive days. Activities included identifying patients at risk for delays in care, conducting social needs assessments, following patients lost to follow up or missed appointments, and communicating with the oncology team. Second, an administrative worksheet for each site captured fixed and variable labor and non-labor costs at startup, intervention implementation, and maintenance phases of the program. Measures included average costs per patient served, and costs per navigation activity.

**Findings:** Ten staff (7 navigators and 3 supervisors) across five programs participated in the time motion survey. Average caseload for each program was 20 patients (range 5-51 patients) during the survey period. For navigators, administrative tasks (e.g., identifying at-risk patients, tracking patients, and monitoring caseloads) required an average of 94 minutes/day (range-0-135), followed by direct patient contact to conduct social needs assessments and referrals with a mean of 54 minutes/day, (range 0-240). For supervisors, administrative supervision averaged 27 minutes/day (range 0-60) and communicating with the oncology team 14 minutes/day (range 0-30). The average cost of navigation services per patient was $2,341 for start-up costs and $4,797 for intervention implementation and maintenance. The mean costs per navigation activity were $3,417 for administrative tasks and $2,217 for direct patient contact.

**Implications for D&I Research:** The findings contribute to the evidence for program costs to engage vulnerable patients in cancer care and treatment post diagnosis. This study may be useful for program managers and hospital administrators to describe tasks, budget estimates, and to make the business case for navigation in health systems.

**Primary Funding Source**

National Institutes of Health

### S77 Implementing best-practice COVID-19 infection control in nursing homes through project echo: A qualitative study using the consolidated framework for implementation research

#### William Calo^1^, Laura Felix^1^, Erica Francis^1^, Gail D'Souza^1^, Liza Behrens^2^, Emily Heilbrunn^1^, Jennifer Kraschnewski^1^

##### ^1^Penn State College of Medicine, Hershey, PA, USA; ^2^The Pennsylvania State University, University Park, PA, USA

###### **Correspondence:** William Calo (wcalo@phs.psu.edu)

*Implementation Science 2023,* **18(Suppl 3):**S77

**Background:** Nursing homes in the US were hit hard by COVID-19, with 710,000 cases and 138,000 deaths through 2021. Little is known about the implementation of best-practice COVID-19 infection control in nursing homes during the pandemic. In a randomized controlled trial (RCT) with 136 nursing homes, our team tested Project ECHO (Extension for Community Health Outcomes), an evidence-based telementoring model, to support infection control guideline implementation. Guided by the Consolidated Framework for Implementation Research (CFIR), this study sought to answer the research questions of how best-practice infection control guidelines were implemented in nursing homes and how Project ECHO, our implementation strategy, facilitated that process.

**Methods:** We conducted in-depth interviews with RCT participants (n=21) from Pennsylvania, Ohio, Vermont, Connecticut, Wisconsin, and Illinois. Virtual interviews occurred between June-September 2021 and lasted 45 minutes each. The interview guide consisted of seventeen CFIR-oriented questions with probes. Interviews were audio-recorded and transcribed verbatim. Coders used rapid qualitative analysis (RQA) techniques to systematically summarize transcripts based on CFIR domains and constructs (italicized). Summaries were consolidated into matrices identifying aspects of best practices for infection control implementation.

**Findings:** Participants were nursing home administrators (71%) and other nursing staff (29%) with an average of 19 years of experience. Our CFIR-guided RQA showed that: 1) *external policies and incentives* sometimes facilitated (e.g., received personal protective equipment) or slowed down (e.g., excessive reporting, conflicting information from federal/state regulators) the implementation of guidelines, 2) nursing homes reported high levels of *readiness for implementation*, supportive *implementation climate*, and *leadership engagement*, 3) the *relative advantage, adaptability, complexity,* and *cost* of best practices were important factors for implementation decisions, and 4) participants appreciated that Project ECHO provided *access to knowledge and information*, fomented a positive *learning climate* and *cosmopolitanism*, and offered opportunities to *reflect on and evaluate* implementation practices.

**Implications for D&I Research:** Integrating CFIR throughout RQA was helpful for evaluating the implementation of best-practice COVID-19 infection control in the context of nursing homes. Our qualitative data indicate that participants valued Project ECHO as a highly acceptable implementation strategy to provide continued education and trusted guidance on infection control guidelines and implementation procedures.

**Primary Funding Source**

Patient-Centered Outcomes Research Institute

### S78 Evaluation of multifaceted strategies to improve postoperative vte chemoprophylaxis adherence in a statewide quality improvement collaborative using coincidence analysis

#### Anthony Yang^1^, Jennifer Slota^1^, Reiping Huang^1^, Ying Shan^1^, Remi Love^2^, Lindsey Kreutzer^3^, Brianna D'Orazio^1^, Julie Johnson^4^, Karl Bilimoria^1^

##### ^1^Northwestern University Feinberg School of Medicine, Chicago, IL, USA; ^2^Surgical Outcomes and Quality Improvement Center- Northwestern University Feinberg School of Medicine, Chicago, USA; ^3^Northwestern Medicine, Chicago, IL, USA; ^4^Northwestern University, Chicago, IL, USA

###### **Correspondence:** Anthony Yang (yangad@iu.edu)

*Implementation Science 2023,* **18(Suppl 3):**S78

**Background**

Appropriate chemoprophylaxis reduces venous thromboembolism (VTE) risk in postoperative patients. This study utilized a novel analytic method (Coincidence Analysis) incorporating both quantitative and qualitative data to examine factors associated with successful local implementation of a statewide quality improvement (QI) project to improve adherence to postoperative VTE chemoprophylaxis best practices among 32 hospitals participating in the Illinois Surgical Quality Improvement Collaborative (ISQIC).

**Methods**

ISQIC hospitals participated in a statewide QI project to improve adherence to postoperative VTE chemoprophylaxis using a comprehensive process measure. ISQIC provided 21 strategies to support local surgical QI teams. Hospitals were considered to have successfully improved if they achieved >50% of their potential improvement in adherence. Coincidence Analysis (CNA), a configurational comparative method, was applied to explore causality between this multifaceted intervention, which was carried out in varying local contexts, and QI success. In total, 25 QI-focused variables were considered in the CAN.

**Findings**

Among 32 participating hospitals, 56% (n=18) successfully improved VTE chemoprophylaxis adherence. The most common condition predicting success was retention of the same local QI leader, the Surgeon Champion (SC), throughout the entire project (53%, n=17). Two pathways to successful improvement were identified: (1) Agreement between the hospital QI team and their process improvement coach on project progress, and (2) a site visit by the ISQIC Coordinating Center to hospitals where the SC was retained. Of 20 hospitals that manifested either pathway, 15 improved (consistency=75%). These two pathways combined explained 15 of the 18 hospitals who successfully improved (coverage=83%).

**Implications for D&I Research**

This study successfully compared a combination of qualitative and quantitative data to identify hospital contextual factors and supportive strategies associated with successful local implementation of a statewide QI project to improve postoperative VTE chemoprophylaxis. Consistent local QI team physician leadership, utilization of site visits from the collaborative Coordinating Center, and effective engagement of local teams in the QI project were all associated with local success in QI project implementation. These findings provide understanding of local contextual factors that support effective QI locally, and can guide where to direct limited resources in support of effective QI.

**Primary Funding Source**

National Institutes of Health

### S79 Implementation factors affecting adoption of electronic patient-generated data for chronic disease management in safety-net settings

#### Elaine Khoong, Faviola Garcia, Kristan Olazo, Billy Zeng, Urmimala Sarkar, Courtney Lyles

##### University of California San Francisco, San Francisco, CA, USA

###### **Correspondence:** Elaine Khoong (elaine.khoong@ucsf.edu)

*Implementation Science 2023,* **18(Suppl 3):**S79

**Background:** Use of patient-generated data (PGD) is an evidence-based approach to improve chronic disease management. Patients with barriers to accessing in-person care, such as safety net populations, may benefit from remote care. This study aimed to understand patient and clinician perspectives on barriers to using electronic PGD in safety net settings.

**Methods:** We used semi-structured interviews with and observations of safety-net patients and clinicians (e.g., providers, nurses, pharmacists) to understand the barriers and facilitators to using electronic PGD in clinical care. We purposively sampled participants to ensure diversity in perspective and experience. We used two implementation science models (COM-B [capability, opportunity, and motivation] and CFIR [consolidated framework for implementation research]) to guide analyses. Three CFIR constructs (outer setting, inner setting, and process) were used to better delineate opportunity-related factors. Interviews were conducted in participants’ preferred language and transcribed. We used deductive coding based on COM-B and CFIR. Research team members iteratively coded the same transcripts until agreement was reached on a final codebook. Remaining transcripts were coded by two members of the research team who had substantial agreement (kappa = 0.73). Themes and representative quotes were identified.

**Findings:** Across four safety-net systems, we observed 18 patient-clinician interactions and interviewed 17 clinical team members and 10 patients (5 English-speaking; 3 Spanish-speaking; 2 Chinese-speaking). Additional patient interviews are planned. Most clinical team members and patients agreed that these factors impact PGD implementation: capability (knowledge to accurately collect and report PGD); motivation (preferences for data sharing; patient-clinician trust; perceptions of the importance of PGD collection to health; patient resiliency; impact on healthcare access); and opportunity (social support). Clinicians also reported the following impacted opportunity: outer setting (pay for performance programs; care in other systems); inner setting (resources; quality of team-based care; health system priorities); and process (development of workflows for triaging/reviewing PGD).

**Implications for D&I Research:** Despite the potential of PGD to improve care experience and outcomes, successful implementation in safety net systems requires alignment of patient, clinician, health system, and policy level factors. If implementation is pursued without addressing all these levels concurrently, barriers to adoption will persist for both clinicians and patients, especially in under-resourced settings.

**Primary Funding Source**

National Institutes of Health

## Global Dissemination and Implementation Science

### S80 Evaluating the sustainment of outputs and outcomes past a program’s end: An assessment of an infant and young child feeding initiative in Bangladesh from 2010-2017

#### Corrina Moucheraud^1^, Adrienne Epstein^2^, Haribondhu Sarma^3^, Sunny Kim^4^, Phuong Hong Nguyen^4^, Mahfuzur Rahman^5^, Md. Tariquijaman^6^, Jeffrey Glenn^7^, Denise Payán^8^, Purnima Menon^9^, Thomas Bossert^10^

##### ^1^Health Policy and Management, UCLA Fielding School of Public Health, Los Angeles, CA, USA; ^2^Liverpool School of Tropical Medicine, Liverpool, United Kingdom; ^3^the National Centre for Epidemiology and Population Health (NCEPH), Australian National University (ANU), Canberra, Acton, ACT, Australia; ^4^International Food Policy Research Institute, Washington, DC, USA; ^5^icddr,b, Dhaka, Bangladesh; ^6^International Centre for Diarrhoeal Diseases Research, Bangladesh (icddr,b), Dhaka, Bangladesh; ^7^Brigham Young University, Provo, UT, USA; ^8^University of California, Irvine, Irvine, CA, USA; ^9^International Food Policy Research Institute, New Delhi, India; ^10^Harvard University, Boston, MA, USA

###### **Correspondence:** Corrina Moucheraud (cmoucheraud@ucla.edu)

*Implementation Science 2023,* **18(Suppl 3):**S80

**Background:** The Alive & Thrive initiative in Bangladesh (implemented 2009-2014) was highly successful at increasing health workers’ infant and young child feeding (IYCF) service delivery and knowledge, as well as job satisfaction and readiness. We evaluate whether these outcomes endured past the program’s end. This study addresses an important implementation science gap, namely whether program outputs and outcomes are sustained over time once external funding ceases.

**Methods:** Alive & Thrive trained and incentivized health workers in 10 sub-districts of Bangladesh to deliver intensified IYCF counseling and participate in social mobilization activities. Leveraging the evaluation’s cluster-randomized controlled trial design, we use data from repeated cross-sectional surveys with randomly-selected health workers in intervention and comparison areas collected in 2010 (baseline, n=290), 2014 (endline, n=511) and 2017 (post-endline, n=600). Outcomes include: IYCF messages delivered during counseling, IYCF knowledge, job satisfaction, and job readiness. Multivariable difference-in-difference linear regression models compared health workers in intervention and comparison areas over time.

**Findings:** The main program effect was attenuated by 2017: although health workers in intervention areas discussed significantly more IYCF topics than those in comparison areas in 2014 (4.9 versus 4.0 topics, p<0.001), by post-endline the difference was no longer significant (4.0 versus 3.3 topics, p=0.067). Higher levels of community support and more comprehensive refresher trainings were protective against this degradation. Health worker IYCF knowledge remained higher in intervention areas post-endline versus comparison areas, suggesting a sustained program impact on knowledge. Job satisfaction and readiness both exhibited a “voltage drop,” i.e., despite having improved during the program period, outcomes were similar in intervention and comparison areas by 2017.

**Implications for D&I Research:** We find a sustained impact on health workers’ IYCF knowledge—but, critically, not on service delivery, which suggests that “know-do” gaps persist, or may widen, over time. We identify potential protective factors (e.g., community support, comprehensive refresher trainings), which merit further exploration as intermediate activities for programs seeking sustainment of key outcomes like behavior change. Our results highlight the importance of conducting impact evaluations that collect post-endline data, to continue refining and testing concepts of sustainment to advance implementation science measures and methods.

**Primary Funding Source**

Bill and Melinda Gates Foundation

### S81 Results of a cluster randomized trial testing the systems analysis and improvement approach (SAIA) as a strategy to improve cervical cancer screening in family planning clinics in mombasa county, Kenya

#### McKenna Eastment^1^, George Wanje^2,3^, Barbra Richardson^4^, Emily Mwaringa^5^, Shem Patta^5^, Kenneth Sherr^6^, Ruanne Barnabas^7,8^, Kishorchandra Mandaliya^1^, Walter Jaoko^2^, R. Scott McClelland^1^

##### ^1^University of Washington, Seattle, WA, USA; ^2^University of Nairobi, Nairobi, Kenya; ^3^University of Washington, Seattle, USA; ^4^Fred Hutchinson Cancer Research Center, Vaccine and Infectious Disease Division, Seattle, WA, USA; ^5^Mombasa County Department of Health, Mombasa, Kenya; ^6^University of Washington, SEATTLE, WA, USA; ^7^Massachusetts General Hospital, Boston, MA, USA; ^8^Harvard Medical School, Boston, MA, USA

###### **Correspondence:** McKenna Eastment (mceast@uw.edu)

*Implementation Science 2023,* **18(Suppl 3):**S81

**Background:** Despite being preventable with appropriate screening and treatment, cervical cancer remains the most common cancer in sub-Saharan Africa. The objective of this cluster randomized trial was to test an implementation strategy to increase cervical cancer screening in family planning (FP) clinics in Mombasa County, Kenya.

**Methods:** Twenty FP clinics were randomized 1:1 to an intervention arm implementing the Systems Analysis and Improvement Approach (SAIA) or control arm with usual procedures beginning 1/2020. SAIA is an evidenced-based multi-component implementation strategy focused on improving care cascades. Step 1 uses a “cascade analysis” tool (CAT) to quantify cascade step completion and identify priority steps for improvement. Step 2 involves flow mapping to identify modifiable system bottlenecks. Step 3 develops and implements targeted workflow modifications to address bottlenecks. Step 4 assesses the modifications’ impact and recalculates the CAT. Step 5 repeats the cycle. Prevalence rate ratios (PRRs) were calculated using Poisson regression to compare SAIA’s effect versus control conditions on rates of cervical cancer screening.

**Findings:** Over the 18-month study, 4.2% of visits with eligible FP clients involved screening in intervention clinics compared to 2.3% in control clinics, resulting in significantly more FP clients being screened for cervical cancer in intervention versus control clinics (PRR 1.84, 95%CI 1.54-2.20). There was substantial variability in screening quarter to quarter in both intervention and control clinics from the SARS-CoV-2 pandemic and a 5-month healthcare worker go-slow and strike. The primary intent-to-treat analysis was based on screening in the last quarter of the trial. During that quarter, 2.5% of visits with eligible FP clients included cervical cancer screening in intervention clinics compared to 3.7% in control clinics (PRR 0.67, 95%CI 0.45-1.00). Because this analysis focused on the proportion of visits with cervical cancer screening, it is not possible to directly assess the proportion of women screened for cervical cancer from these data.

**Implications for D&I Research:** Despite the variability from quarter to quarter, the overall rate of screening in intervention clinics was nearly twice the rate in control clinics. These results highlight SAIA’s potential to further improve cervical cancer screening coverage in this important population.

**Primary Funding Source**

National Institutes of Health

### S82 Developing sustainable strategies for delivery and evaluation of an evidence-based violence prevention program in Dominican Republic secondary schools

#### Heidi Luft^1^, Kathleen Stevens^2^, Jonathan Pettigrew^3^, Betty Reyes^4^, Julio Arturo Canario Guzmán^5^, Cassandra Gamble^5^, Abdel Montoya^5^, Alexandria Sedar^6^, Ryann Fierro^1^, Jeff Temple^1^

##### ^1^University of Texas Medical Branch, Galveston, TX, USA; ^2^University of Texas Health San Antonio, San Antonio, TX, USA; ^3^Arizona State University, Tempe, AZ, USA; ^4^Dominican Republic Ministry of Education, Santo Domingo, Dominican Republic; ^5^Institute of Mental Health and Telepsychology-Etikos Bioethics Foundation, Santo Domingo, Dominican Republic; ^6^Independent Consultant, Chicago, IL, USA

###### **Correspondence:** Heidi Luft (heluft@utmb.edu)

*Implementation Science 2023,* **18(Suppl 3):**S82

**Background:** Preventing injury (violence, teen pregnancy, unintentional injury) among adolescents is a priority for the future of every society. Evidence-based interventions (EBIs) have been implemented in the USA, but guidance for adapting implementation into low-resource settings is scant. Using implementation science (IS) approaches, we aimed to develop strategies for delivery of a holistic violence prevention EBI^3^ in public secondary schools throughout the Dominican Republic (DR). The DR is a particularly important context, because it has among the greatest burden of “injury excess” worldwide.^1^

**Methods:** Implementation mapping^2^ is a novel IS method that provides a systematic process for developing context-specific implementation strategies for EBIs in new settings. Mapping includes a needs assessment coupled with an analysis of implementation structures available in a given setting. In the DR, mapping occurred in collaboration with the DR Ministry of Education for a violence prevention EBI^3^ in public schools. It involved extensive engagement with multisectoral stakeholders (policy makers, public education leaders, teachers/students, caregivers, health workers, religious leaders, violence prevention activists, lawyers, first responders). Using mixed methods data collection and analysis, we identified implementation actors, outcomes, performance objectives, and determinants. We organized this information into a change matrix. Finally, we framed the findings with the Consolidated Framework for Implementation Research (CFIR)^4^ model and used a matching tool to select implementation strategies.

**Findings:** We present findings as an implementation mapping logic model that charts the EBI to the selected implementation methods and strategies, behavioral/contextual determinants of implementation, tasks enabling implementation, implementation outcomes, and effectiveness outcomes.

**Implications for D&I Research:** Findings will guide the next mapping tasks: production of implementation protocols/materials and evaluation of implementation outcomes through a rigorous pragmatic trial. This investigation paves the way for development of best practice for implementation of adolescent violence prevention EBIs in low-resource school settings. Particularly, in a region and country where the need is great and rigorous, resource-sensitive implementation approaches are most limited. This represents the first application of implementation mapping in Latin America and the Caribbean and adds to what is known about the utility of contemporary IS approaches to global IS.

**Primary Funding Source**

US Department of State

**Reference**

1. DOI:10.1016/S0140-6736(16)00579-1
2. doi:10.3389/fpubh.2019.00003
3. DOI: 10.1001/archpediatrics.2009.69
4. doi:10.1186/1748-5908-4-50

### S83 Selecting implementation strategies to test to improve implementation of integrated PrEP for pregnant and postpartum populations in Kenya

#### Sarah Hicks^1^, Felix Abuna^2^, Ben Odhiambo^2^, Julia Dettinger^1^, Nancy Mwongeli^2^, Lauren Gomez^1^, Joseph Sila^2^, George Oketch^2^, Enock Sifuna^2^, Bryan Weiner^1^, Grace John-Stewart^3^, John Kinuthia^2^, Anjuli Wagner^1^

##### ^1^University of Washington, Seattle, WA, USA; ^2^Kenyatta National Hospital, Nairobi, Kenya; ^3^University of Washington, Seattle, WA, USA

###### **Correspondence:** Sarah Hicks (smd722@uw.edu)

*Implementation Science 2023,* **18(Suppl 3):**S83

**Background**

In high HIV prevalence settings, women are at elevated risk for HIV during pregnancy and postpartum, and pre-exposure prophylaxis (PrEP) is recommended during this period. Integration of PrEP into maternal and child health (MCH) clinics requires implementation optimization.

**Methods**

The PrEP in Pregnancy, Accelerating Reach and Efficiency study (PrEPARE; NCT04712994) engaged stakeholders to identify determinants of PrEP implementation, and identify and prioritize PrEP delivery implementation strategies at 55 facilities in Kenya through quantitative surveys and a stakeholder workshop. Determinants were assessed using Likert scores related to impact on PrEP delivery. Strategies were prioritized using two quantitative ranking surveys and visual go-zone plots of stakeholders’ perceived feasibility and effectiveness of the strategies. A stepwise elimination process was used to identify seven strategies for empirical testing. Facilitator debriefing reports from the workshop were used to qualitatively assess the decision-making process.

**Findings**

Among 146 health care workers, the strongest reported barriers to PrEP delivery were: insufficient providers and inadequate training, insufficient space, and volume of patients. Sixteen strategies were assessed, 14 of which were included in the final analysis. Using rankings from 182 healthcare workers and 44 PrEP policymakers and implementers, seven strategies were eliminated based on low post-workshop ranking scores (bottom 50^th^ percentile) or falling outside the go-zone (low perceived feasibility and effectiveness) for at least 50% of the workshop groups. The top three strategies included 1) dispensing PrEP within MCH clinics instead of pharmacies, 2) fast tracking PrEP clients to reduce waiting time, and 3) delivering PrEP-related health talks in waiting bays. All top seven ranked strategies were grouped into bundles for subsequent testing per conversations with study staff. Facilitator debriefing reports generally aligned with go-zone rankings but noted how stakeholders’ decision-making changed when considering the impact of strategies on facility staff and non-PrEP clients.

**Implications for D&I Research**

The most impactful barriers to integrated PrEP delivery in MCH clinics focused on insufficient staffing and space. Implementation strategies prioritized through multiple methods of stakeholder input focused on co-location of services and increasing clinic efficiency. Future testing of these stakeholder-prioritized strategy bundles will be conducted to assess effectiveness and implementation outcomes.

**Primary Funding Source**

National Institutes of Health

### S84 Advancing integrated alcohol-HIV training of frontline providers across South Africa

#### Kira DiClemente-Bosco^1,2^, Caroline Kuo^3,4^, Goodman Sibeko^5^, Shaheema Allie^6^, Nurain Tisaker^5^, Mziwabantu Fosi^5^, Warren Cornelius^5^, Sara Becker^1,2^

##### ^1^Brown School of Public Health, Providence, RI, USA; ^2^Northwestern University, Chicago, IL, USA; ^3^Brown University, Providence, RI, USA; ^4^American University, Washington, DC, USA; ^5^University of Cape Town, Cape Town, South Africa; ^6^South Africa International Technology Transfer Centre (UCT), Cape Town, South Africa

###### **Correspondence:** Sara Becker (sara_becker@brown.edu)

*Implementation Science 2023,* **18(Suppl 3):**S84

**Background:** In South Africa, rates of HIV and alcohol-use are among the highest globally, and these epidemics have a detrimental synergistic relationship. Screening, Brief Intervention, and Referral to Treatment (SBIRT) is an evidence-based, cost-effective approach to identifying people with or at risk of substance use issues to deliver early intervention. This study examines whether we can harness the power of a cascading SBIRT train-the-trainer model to identify and prevent substance use issues in HIV care settings with ease and efficiency at scale despite high patient-to-provider workloads.

**Methods:** This implementation trial is partnered with a national healthcare organization in South Africa to design and implement a cascading train-the-trainer model that includes a scalable training resource suite and digital SBIRT tools to build the capacity of the HIV workforce to identify risky alcohol use, deliver brief interventions in real time, and refer to treatment when needed. Screening is conducted via the Alcohol Use Disorders Identification Test (AUDIT), a tool developed by the World Health Organization and tested in South Africa. Here we present preliminary indicators of the effect of the SBIRT train-the-trainer model on implementation outcomes measured at the trainer-, provider- and patient-level.

**Findings:** Eleven trainers trained 211 lay providers. Fidelity monitoring indicated 98.9% of training elements were covered in full with an average skill rating of 2.88 (scale of 1-3). An estimated 33.4% of all patients entering the clinic received screening, and as of June 1, 2022, a total of 24,077 patients have been screened. Roughly 5,000 patients reported any alcohol use and 642 reported risky use. Roughly 9,500 patients received brief intervention and 555 patients received a referral to treatment, levels of reach that were far higher than indicated. Additional trainer-, provider- and patient-level outcomes will be presented.

**Implications for D&I Research:**

A train-the-trainer cascade model developed with a national organization has demonstrated preliminary evidence of feasibility, high trainer fidelity, promising levels of screening reach, and higher than indicated reach for brief intervention and referral to treatment. This research demonstrates the feasibility of a highly scalable train-the-trainer strategy to advance integrated alcohol-HIV services, which can extend to other low resource and high clinical care burden settings.

**Primary Funding Source**

National Institutes of Health

### S85 Comparison of methods to engage diverse stakeholder populations in prioritizing implementation strategies for testing in resource-limited settings

#### Sarah Hicks^1^, Felix Abuna^2^, Ben Odhiambo^2^, Julia Dettinger^1^, Nancy Mwongeli^2^, Lauren Gomez^1^, Joseph Sila^2^, George Oketch^2^, Enock Sifuna^2^, Bryan Weiner^1^, Grace John-Stewart^1^, John Kinuthia^2^, Anjuli Wagner^1^

##### ^1^University of Washington, Seattle, WA, USA; ^2^Kenyatta National Hospital, Nairobi, Kenya

###### **Correspondence:** Sarah Hicks (smd722@uw.edu)

*Implementation Science 2023,* **18(Suppl 3):**S85

**Background**

There is a lack of consensus about how to prioritize potential implementation strategies. We compared several prioritization methods for their agreement and pragmatism in practice.

**Methods**

We engaged stakeholders (national- and county-level PrEP implementers, healthcare workers, and PrEP users) across 55 facilities in Kenya to prioritize 16 HIV pre-exposure prophylaxis (PrEP) delivery implementation strategies. We compared four strategy prioritization methods: 1) surveys with experienced practitioners reflecting on implementation experience (N=182); 2 & 3) relative ranking surveys (1-16) before and after small group discussion with diverse stakeholders (N=44 & 40); 4) “go-zone” quadrant plots of perceived effectiveness vs feasibility. Kendall’s correlation analysis was used to compare the 4 resultant strategy prioritization profiles. Additionally, the participants grouped strategies in three bundles with up to 4 strategies each by phone and online survey.

**Findings**

The strategy ranking correlation was strongest between the pre- and post-small group rankings (Tau = 0.648; p<0.001). There was moderate correlation between go-zone plots and post-small group rankings (Tau = 0.363; p=0.079) and between past-experience surveys and post-small group rankings (Tau = 0.385; p-value = 0.062). Strategy rankings remained similar between pre- and post-small group discussions; exceptions were in cases of feasibility concerns raised during discussions by experienced stakeholders.

In both strategy bundle formats, participants primarily chose bundles of strategies in the order in which they appeared in the list, reflecting option ordering bias. Individuals who completed the phone survey with oversight from study staff were more likely to select the correct number of strategies per bundle.

**Implications for D&I Research**

Both experienced and inexperienced stakeholder participants’ strategy rankings tended to prioritize strategies that had been previously tested. Small group discussions focused on feasibility and effectiveness revealed moderately different priorities than individual rankings. The strategy bundling approach tested a less time- and resource-intensive method but was not effective. Future research should compare the relative agreement and pragmatism of methodologies to prioritize implementation strategies.

**Primary Funding Source**

National Institutes of Health

### S86 Centering community health worker insights for better program design: Adapting a diffusion of innovations model in Pakistan

#### Svea Closser^1^, Erin Finley^2^, Marium Sultan^3^

##### ^1^Johns Hopkins Bloomberg School of Public Health, Baltimore, MD, USA; ^2^South Texas Veterans Health Care System, San Antonio, TX, USA; ^3^Johns Hopkins University School of Medicine, Baltimore, MD, USA

###### **Correspondence:** Svea Closser (sclosser@jhu.edu)

*Implementation Science 2023,* **18(Suppl 3):**S86

**Background**

Across many LMICs, Community Health Workers (CHWs), the majority of whom are women, are the bedrock of primary care provision. CHWs are often employed at the bottom of health hierarchies where they have little voice; in state programs and vertical initiatives, accountability has generally flowed downwards. Yet, many programs might function better if their needs were considered in program design.

We adapted the “VHA Shark Tank” competition, part of the Diffusion of Excellence program at the US VHA, and implemented it with female CHWs working on polio vaccination in Peshawar, Pakistan. Despite more than 20 years of dedicated effort, Pakistan is one of the last two countries on Earth with endemic wild polio. The goal of our process was to identify promising practices for polio elimination developed by CHWs, and diffuse them across Pakistan.

**Methods**

We held a facilitated competition for teams of CHWs to propose improvements to the process of vaccinating children. We worked with teams in brainstorming sessions to develop promising practices. We then worked with local policymakers to shortlist the best ideas; shortlisted ideas were presented to a panel of provincial and national-level policymakers who selected ideas for nationwide dissemination. We conducted interviews with polio workers and policymakers throughout the process to understand their experiences (n=82).

**Findings**

We received 181 idea submissions; 9 were chosen for implementation. CHWs valued the process enormously; most said it was the first time their insights had ever been considered, and they wanted more opportunities for such input. The second round of the process was more effective than the first, with workers generating more complex ideas and program staff running the process themselves. Overall, the innovations selected for implementation were tweaks rather than major programmatic changes—reflective of the power dynamics within the program. Still, CHWs reported that the changes made were helpful, and said it was very meaningful to have contributed to program policy.

**Implications for D&I Research**

Power relations matter in the diffusion and dissemination of effective interventions; structured processes can allow the least powerful actors in global health interventions to suggest innovations that work for them.

**Primary Funding Source**

Bill and Melinda Gates Foundation

### S87 Designing for dissemination: crowdsourcing open call to identify public preferences for health information dissemination

#### Alexis Engelhart^1^, Idia Thurston^2^, Chisom Obiezu-Umeh^3^, Dr. Ucheoma Nwaozuru^4^, MaryClaire Pavlick^1^, Stacey Mason^3^, Onyekachukwu Anikamadu^3^, Titilola Gbaja-Biamila^5^, Whitney Howie^2^, Christian Herrera^2^, Sheryl Monks^4^, Nnenna Kalu Makanjuola^6^, Jessica Lake^1^, Juliet Iwelunmor^1^

##### ^1^Saint Louis University, St. Louis, MO, USA; ^2^Texas A&M University, College Station, TX, USA; ^3^Saint Louis University, Saint Louis, MO, USA; ^4^Wake Forest University School of Medicine, Winston-Salem, NC, USA; ^5^College for Public Health & Social Justice, Saint Louis University, Saint Louis, MO, USA; ^6^Radiant Health Magazine, Atlanta, GA, USA

###### **Correspondence:** Alexis Engelhart (alexis.engelhart@slu.edu)

*Implementation Science 2023,* **18(Suppl 3):**S87

**Background:** Effectively disseminating and communicating health information to the public is crucial for intervention adoption and impact. The majority of research findings are disseminated through academic journals, with limited focus on preferred dissemination strategies for the public. The objective of this study was to describe the responses to a crowdsourcing contest focused on using creativity to disseminate public health information.

**Methods:** The LIGHT (Leaders Igniting Generational Healing and Transformation) crowdsourcing open call was held online from February to May 2022, and asked the general public to respond to the prompt: “How might we recreate public health as art, letters, stories, and poetry?” Crowdsourcing open calls involve a group of individuals solving all or part of a problem and then sharing solutions with the public. Each submission was judged by four independent individuals on a 1–10 scale, assessing innovation, clarity of expression, originality and creativity in expression, the appeal of content, and relevance to public health. Ideas and perceptions generated from the crowdsourcing contest were qualitatively analyzed using thematic content analysis.

**Findings:** The crowdsourcing open call received a total of 192 submissions via Submittable. After ineligible submissions were removed, there were 155 submissions evaluated. The top ideas recipients were from Trinidad and Tobago, the UK, South Africa, Canada, and the US. Most entries focused on dissemination strategies to promote mental health and COVID-19 prevention/management. Three main unique dissemination strategies emerged for health promotion: a) use of interactive illustrations and arts, b) use of written words like poetry and letters to communicate health information to the public, and c) documentation of lived experiences in the form of stories. Collectively, there was a consensus to innovate on dissemination strategies, to enhance the reach of research findings and health communication.

**Implications for D&I Research:** The LIGHT crowdsourcing contest engaged a broad audience to generate ideas and perspectives on promoting health information dissemination to the public. These findings can inform the reimagining of dissemination strategies to prioritize the public’s preferences and voices.

### S88 Adapting and testing organizational readiness for change measures in south african clinics

#### Hannah Leslie^1^, Sheri Lippman^1^, Alastair van Heerden^2^, Mbali Nokuluna Manaka^3^, Bryan Weiner^4^, Wayne Steward^1^

##### ^1^University of California, San Francisco, San Francisco, CA, USA; ^2^Human Sciences Research Council, Sweetwaters, South Africa; ^3^Human Sciences Research Council, Pretoria, South Africa; ^4^University of Washington, Seattle, WA, USA

###### **Correspondence:** Hannah Leslie (hannah.leslie@ucsf.edu)

*Implementation Science 2023,* **18(Suppl 3):**S88

**Background:** Organizational readiness for change (ORC) is theorized as important for program implementation. ORC is grounded in constructs reflecting individuals’ commitment to and sense of efficacy in effecting change, which have strong predictive value in western cultures. A more collectivist orientation, paired with resource constraints and hierarchical power structures, may limit such constructs’ relevance in other cultural contexts. We aimed to adapt and test measures of readiness for change in South African primary care clinics.

**Methods:** We convened a panel of South African experts in social science and HIV care delivery and presented implementation domains informed by ORC and the Consolidated Framework for Implementation Research as well as prior work in South Africa. Based on panel input, we prioritized contextual domains and adapted candidate items. We conducted 27 cognitive interviews with providers in KwaZulu-Natal Province to refine measures. We then sampled 16 clinics and 5-20 providers per clinic to administer measures. We assessed reliability using Cronbach’s alpha and calculated interrater agreement (r*wg) and intraclass correlation (ICC) at the clinic level.

**Findings:** Panelists emphasized contextual factors over individual agency; we therefore focused on elements of clinic leadership, stress, cohesion, and collective problem solving (critical consciousness). Cognitive interviews confirmed difficulty separating personal commitment from shared understanding of duties and values. All scales except coordination demonstrated reliability ≥0.70, and all but stress showed agreement within clinic (r*wg ≥0.70). ICC was low for most leadership measures and moderate for others.

**Implications for D&I Research:** As theorized, understanding ORC in the South African health system requires attention to overall clinic characteristics. Adapted measures show good reliability at individual and clinic levels, with exceptions. Further testing against implementation outcomes and revision of existing theory to suit this context is warranted.

**Primary Funding Source**

National Institutes of Health

**Table 1 (abstract S88).** Performance of seven organizational readiness measures

	Providers (N=185)		Clinics (N=16)	
	Average ± SD	Alpha	Average r*wg	ICC
Leadership: engagement	2.97 ± 0.30	0.83	0.72	0.00
Leadership: feedback	3.09 ± 0.29	0.77	0.74	0.03
Leadership: Resource mobilization	2.81 ± 0.38	0.73	0.70	0.03
Leadership: Coordination	3.02 ± 0.27	0.66	0.80	0.11
Stress	2.73 ± 0.39	0.85	0.61	0.25
Cohesion	2.88 ± 0.37	0.83	0.80	0.14
Critical consciousness	2.95 ± 0.23	0.84	0.70	0.22

### S89 Organizational climate and implementation fidelity in a scale-up study of the systems analysis and improvement approach for prevention of mother-to-child hiv transmission (saia-scale)

#### Kristjana Ásbjörnsdóttir^1^, Sarah Gimbel^2^, Mery Agostinho^3^, Fernando Amaral^3^, Joana Coutinho^4^, Jonny Crocker^2^, Maria Cruz^3^, Maria de Fatima Cuembelo^5^, Aneth Dinis^2^, Isaias Ramiro^6^, Kenneth Sherr^7^

##### ^1^University of Iceland, Reykjavik, Iceland; ^2^University of Washington, Seattle, WA, USA; ^3^Comité para Saúde de Moçambique, Chimoio, Mozambique; ^4^Comité para Saúde de Moçambique, Beira, Mozambique; ^5^Eduardo Mondlane University, Maputo, Mozambique; ^6^Comité para Saúde de Moçambique, Biera, Mozambique; ^7^University of Washington, SEATTLE, WA, USA

###### **Correspondence:** Kristjana Ásbjörnsdóttir (kha@hi.is)

*Implementation Science 2023,* **18(Suppl 3):**S89

**Background**

As evidence-based interventions are scaled up through healthcare systems, external inputs often become diluted and implementation fidelity varies across clinics. In this context, organizational readiness, culture and climate may be important determinants of fidelity, which in turn mediates intervention effectiveness. We assessed the association between organizational characteristics and implementation fidelity in SAIA-Scale (NCT03425136), a stepped-wedge trial scaling up a package of systems engineering tools (SAIA) to improve service quality for prevention of mother-to-child transmission of HIV (PMTCT) in Manica Province, Mozambique.

**Methods**

A survey containing the Organizational Readiness for Implementing Change (ORIC), Organizational Citizenship Behavior Checklist, and selected constructs from the Organizational Climate Measure and Implementing Computerized Technology was translated, piloted and adapted prior to use in the trial. Healthcare personnel involved in PMTCT were invited to complete the survey within 60 days of the introduction of SAIA at their facilities. Linear regression was used to assess associations between facilities’ mean scores on each domain and implementation fidelity, defined as the number of a) unique micro-interventions proposed and b) micro-interventions implemented as planned in Year 1, with clustering by district.

**Findings**

A total of 208 respondents across 36 intervention facilities in 12 districts completed the survey. Scores across constructs were generally high, ranging from a mean of 71.3% (std.dev. 15.5%) on the domain quantifying Personnel Effort to a mean of 95.9% (std.dev. 7.9%) on ORIC. Facilities proposed a mean of 10.3 unique micro-interventions in Year 1 (range 4-12), and a mean of 8.2 were implemented as planned (range 4.5-11). Relative Priority scores were associated with a greater number of unique micro-interventions (0.65/10% increase, p=0.009), while Management Support was associated with a greater number successfully implemented (0.55/10% increase, p=0.02). Other constructs were not associated with either measure of fidelity.

**Implications for D&I Research**

We assessed many domains of organizational culture and climate and found most were not associated with implementation fidelity. Possibly, external support from the trial in Year 1 was sufficient to reduce the impact of organizational factors, or selected outcome measures failed to capture variations in implementation fidelity. Future work is planned to assess the direct association with intervention effectiveness and sustainment.

**Primary Funding Source**

National Institutes of Health

### S90 Development of standards for reporting scaling studies of health interventions: a systematic review

#### Amédé Gogovor^1,2,3,4^, Hervé Tchala Vignon Zomahoun^5^, Ali Ben Charif^6^, Robert McLean^7^, David Moher^8,9^, Andrew J Milat^10,11^, Luke Wolfenden^12^, Karina Prévost^13^, Emmanuelle Aubin^13^, Paula Rochon^14,15^, Giraud Ekanmian^16,17^, Nathalie Rheault^16,18^, France Légaré^4,16,17,19^

##### ^1^Vitam – Centre de recherche en santé durable, Quebec, QC,, Quebec, Canada; ^2^Université Laval, Québec, QC, Canada; ^3^Canada Research Chair in Shared Decision Making and Knowledge Translation, Quebec, Canada; ^4^Unité de soutien SSA Québec, Quebec, Canada; ^5^Université Laval, Quebec City, Canada; ^6^CubecXpert, Quebec City, Canada; ^7^International Development Research Centre, Toronto, Canada; ^8^University of Ottawa, Ottawa, ON, Canada; ^9^Ottawa Hospital Research Institute, Ottawa, ON, Canada; ^10^Centre for Epidemiology and Evidence, NSW Ministry of Health, Sydney, Australia; ^11^School of Public Health, University of Sydney, Sydney, Australia; ^12^School of Medicine and Public Health, The University of Newcastle, Callaghan, NSW, Australia; ^13^Patient Partner, Quebec, Canada; ^14^Women's College Hospital, Toronto, ON, Canada; ^15^University of Toronto, Toronto, Canada; ^16^Vitam – Centre de recherche en santé durable, Quebec, Canada; ^17^Université Laval, Quebec, Canada; ^18^Unité de soutien SSA Québec, Quebec, QC, Canada; ^19^Tier 1 Canada Research Chair in Shared Decision Making and Knowledge Translation, Quebec, QC, Canada

###### **Correspondence:** Amédé Gogovor (amede.gogovor.1@ulaval.ca)

*Implementation Science 2023,* **18(Suppl 3):**S90

**Background**

Adequate reporting of studies assessing the impact of scaling of health interventions can facilitate their replication and translation in practice and policy. We sought to identify relevant items for a reporting guideline for scaling studies of health interventions.

**Methods**

We performed a systematic review of studies that met the following criteria: any guide or document that provides instructions or recommendations, e.g., reporting guideline, checklist, guidance, framework, standard. We searched MEDLINE, EMBASE, PsycINFO, Cochrane Library, CINAHL, Web of Science through 2020 with no language restriction. We also searched the website of relevant organizations. The search strategy was based on a combination of free and controlled vocabularies of these main concepts: reporting standard, implementation, scaling, health. After pilot testing the eligibility criteria on a randomly selected sample of records, screening for titles, abstracts and full texts was performed independently by pairs of four reviewers using Covidence. Discrepancies were resolved by consensus or by a third reviewer. Data from extraction and assessment of the quality (based on three criteria) of included guidelines were synthesized narratively using descriptive statistics and a list of items divided into main categories of reporting checklist was generated. The number of items was reduced based on constructs relevant to the science and practice of scaling to be used in a Delphi study.

**Findings**

A total of 37 guidelines from 56 reports were included. They were published between 1999 and 2019 mainly from USA (17 out of 37). Of the 37 guidelines, 22 were developed to report implementation interventions and 15 to design scaling interventions; none was developed to report scaling interventions. Only one guideline included patients in the development process. In terms of evidence-based development of the included guidelines, 57% (21/37) were of high quality and 43% (16/37) of low quality. The 37 guidelines yielded 736 unique items, organized by main categories of a checklist from the ‘title’ to ‘other information’.

**Implications for D&I Research:** The review will inform the development of a reporting guideline for scaling studies of evidence-based health interventions, thus contributing to quality reporting in the science of implementation and scaling.

**Primary Funding Source**

CIHR through Unité de soutien SSA Québec

### S91 The sustainability of health interventions implemented in sub-saharan africa: an updated systematic review on evidence and future research perspectives

#### Juliet Iwelunmor^1^, Patrick Murphy^1^, Ashley Richard^1^, Dr. Ucheoma Nwaozuru^2^, Chisom Obiezu-Umeh^1^, Alexis Engelhart^1^, Titilola Gbaja-Biamila^3^, Stacey Masn^1^, Ifeoma Obionu^1^, Thembekile Shato^4^, Onyekachukwu Anikamadu^1^, Pranali Patel^1^, Victor Ojo^5^, Victoria Carter^1^, David Oladele^5^, Jessica Lake^6^

##### ^1^Saint Louis University, St. Louis, MO, USA; ^2^Wake Forest University School of Medicine, Winston-Salem, NC, USA; ^3^College for Public Health & Social Justice, Saint Louis University, Saint Louis, MO, USA; ^4^Washington University in St. Louis, St. Louis, MO, USA; ^5^Clinical Sciences Department, Nigerian Institute of Medical Research, Lagos, Nigeria; ^6^Veterans Health Administration, Saint Louis, USA

###### **Correspondence:** Patrick Murphy (patrick.w.murphy@slu.edu)

*Implementation Science 2023,* **18(Suppl 3):**S91

**Background:** Sustaining evidence-based interventions in resource-limited settings is critical to optimize gains in health outcomes. We previously published a review of the sustainability of health interventions in sub-Saharan Africa (SSA) in 2015, highlighting a gap in the measurement and conceptualization of sustainability in SSA. This review provides an update and expands upon the original review to account for developments in the past few years and recommendations for promoting sustainability.

**Methods:** We searched five databases for studies published between 2015 and 2022 in accordance with Preferred Reporting Items for Systematic Review and Meta-Analysis guidelines. Studies were included if they reported on the sustainability of health interventions implemented in SSA. Two researchers independently extracted information from each article using a validated data extraction tool.

**Findings:** Twenty-nine publications with 27 distinct interventions were included in the review. Twelve countries were represented in this review, with Uganda (n=7) having the most representation of available studies examining sustainability. Compared to the 2015 review, a slightly higher proportion of studies had a clear definition of sustainability (69% in the current review versus 51% in the 2015 review). However, only seven studies discussed framing their sustainability assessment using a theory or conceptual framework. Four key factors emerged as important determinants of sustainability: a) *people* (individuals who were involved in the sustainability process), b) *learning* (collaborative and iterative problem solving to enhance intervention acceptability and fit ), c) *adaptation* (thoughtful and deliberate alteration of intervention delivery to improve fit in a given context), and d) *nurturing factors* (contextual and supportive influences likely to contribute to the long-term maintenance of evidence-based practices within particular contexts). The most prevalent facilitators of sustainability were related to micro-level factors (e.g., intervention fit, stakeholder engagement), while salient barriers were related to structural level factors (e.g., limited financial resources).

**Implications for D&I Research:** This review highlights some progress in the documentation of sustainability in evidence-based intervention in SSA. It also emphasizes the importance of factors such as *people, learning, adaptation, and nurturers* in promoting sustainability. This calls for the development of contextually relevant sustainability conceptual frameworks that emphasize these salient determinants of sustainability in the region.

### S92 Characterizing telemedicine barriers and preferences to promote acceptable implementation strategies in central uganda

#### Kelly Hirko

##### Michigan State University, East Lansing, MI, USA

###### **Correspondence:** Kelly Hirko (hirkokel@msu.edu)

*Implementation Science 2023,* **18(Suppl 3):**S92

**Background:** The lack of knowledge about specific challenges and preferences for telehealth in African countries limits the ability to implement effective and appropriate telehealth approaches to address healthcare access barriers in Africa. Thus, the purpose of this study was to conduct a formative evaluation to identify barriers and facilitators for implementing telehealth approaches in central Uganda.

**Methods:** Using a mixed-methods design, we distributed surveys and conducted focus groups and in-depth semi-structured interviews of 150 key partners, including providers, patients, healthcare administrators, and health information technology staff in central Uganda. The assessment was informed by the Technology Acceptance Model (TAM), and evaluated predictors of technology acceptance (perceived usefulness, social influences, and attitudes). We used descriptive statistics to characterize telehealth acceptance and barriers.

**Findings:** Nearly 79% of 61 providers surveyed had used telehealth and perceptions were generally favorable. While 92% reported that telehealth adds value to clinical practice, less than half felt that telehealth was more efficient than in-person visits. Provider-reported barriers to telehealth included technology challenges for the patient (55.7%), low patient engagement (41%), and lack of implementation support (39.3%). Only 19.8% of the 91 patients surveyed had used telehealth. Perceptions of telehealth were less favorable among patients compared to providers, with 59.3% of patients reporting satisfaction with telehealth services. Although 74.7% reported telehealth saving time, nearly 33% of patients reported that telehealth made them feel uncomfortable and 42.9% reported concerns about confidentiality with telehealth. Providers cited usefulness of telehealth for communicating with patients, but expressed concerns around ease of use given low digital literacy and the need for infrastructure to support telehealth.

**Implications for D&I Research:** Results from this study will inform the implementation of acceptable and sustainable telehealth systems to address healthcare disparities propagated by healthcare access barriers in central Uganda. This research provides a replicable and scalable model to address healthcare access barriers in other under-resourced settings.

**Primary Funding Source**

Alliance for African Partnership, Michigan State University

### S93 Health care providers’ view on a couple-based hiv care and treatment program using the consolidated framework for implementation research (cfir) 2.0

#### Carolyn Audet^1^, Hannah Brooks^2^, Erin Graves^2^, Almiro Emilio^3^, Ariano Matino^3^, Arifo Aboobacar^4^, Caroline DeSchacht^5^

##### ^1^Health Policy, Vanderbilt University Medical Center, Nashville, TN, USA; ^2^Vanderbilt University Medical Center, Nashville, TN, USA; ^3^Friends In Global Health, Quelimane, Mozambique; ^4^Ministry of Health, Mozambique, Quelimane, Mozambique; ^5^Friends In Global Health, Maputo, Mozambique

###### **Correspondence:** Carolyn Audet (carolyn.m.audet@vanderbilt.edu)

*Implementation Science 2023,* **18(Suppl 3):**S93

**Background**

In Mozambique, 15% of HIV-exposed infants seroconvert by 18 months of age. One of the barriers to prevention of mother-to-child transmission has been male partner behavior inhibiting women from adhering to treatment. We implemented a cluster-randomized controlled trial studying the effect of a couple-based treatment strategy for expectant HIV-seroconcordant couples on retention to HIV care and vertical transmission. This abstract seeks to describe elements of intervention implementation: (1) factors that facilitated provider support for this intervention, (2) providers’ perceptions of implementation challenges, and (3) strategies proposed to facilitate implementation success.

**Methods**

We conducted 100 in-depth interviews with health care providers (51 at intervention sites and 49 at control sites) across seven districts (24 rural health facilities total) in Zambézia province, between January 2020 and July 2021. Interview questions and data analysis were guided by the Consolidated Framework for Implementation Research (CFIR 2.0). Thematic analysis was conducted by two analysts using MAXQDA 2022.

**Findings**

The CFIR constructs that drove provider attitudes toward implementation included intervention characteristics (*Relative Advantages*), roles (*Intervention Deliverers*), individuals (*Need*), outer setting (*Local Attitudes, Conditions*), and inner setting factors (*Available Resources, Compatibility*). Providers (both in control and intervention sites) felt couple-based care was more efficient for providers (saving time, providing information about the relationship that aids care delivery), accelerated intervention by health care providers (via community outreach) if a patient abandoned care, and facilitated couples counseling if mistrust or disagreement were expressed. Providers felt that the program fit well with patient needs and community characteristics, however, expressed concern that women in difficult relationships may not feel comfortable speaking truthfully in front of their partner. They suggested a limited number of women-only visits as needed. Providers at intervention and control sites expressed potential concerns regarding inadequate space to implement the program and ensure privacy. Small tents and out-buildings at health facility locations were seen as suitable adaptations for program delivery.

**Implications for D&I Research**

Providers working in rural health facilities perceived a substantial benefit to delivering a novel couple-based care program to expectant couples living with HIV, despite structural space limitations and concerns that some male partners could limit a woman’s uptake of desired services.

**Primary Funding Source**

National Institutes of Health

### S94 How does brazil’s criança feliz early childhood development program work? A combined pip-cfir analysis

#### Gabriela Buccini^1^, Keishmer Cardoso^2^, Sonia Isoyama Venancio^3^, Rafael Pérez-Escamilla^4^

##### ^1^University of Nevada Las Vegas, Las Vegas, NV, USA; ^2^UNLV, Las Vegas, NV, USA; ^3^Instituto de Saúde, Sao Paulo, Brazil; ^4^Yale School of Public Health, New Haven, CT, USA

###### **Correspondence:** Gabriela Buccini (gabriela.buccini@unlv.edu)

*Implementation Science 2023,* **18(Suppl 3):**S94

**Background:** To address inequities preventing children from reaching their full developmental potential, Brazil implemented the largest worldwide early childhood development (ECD) program, Programa Criança Feliz (PCF) aims to (1) provide evidence-based home visits and (2) coordinate multisectoral actions to reduce families’ vulnerabilities, and ultimately benefit parenting and ECD outcomes. PCF has been scaled up to 2,934 of 5,570 Brazilian municipalities in rural and urban areas. However, after five years of implementation, the implementation pathways required to achieve PCF's intended goals are unclear. We conducted a program impact pathway (PIP) analysis to identify PCF functions and mechanisms associated with implementation success.

**Methods:** The PIP analysis was informed by: (a) document review; (b) 23 in-depth interviews with key stakeholders; (c) a workshop with stakeholders PCF National Coordination teams. Program functions and mechanisms identified through the PIP analysis were mapped into the CFIR (Consolidated Framework for Implementation Research) to identify implementation constructs that can explain “why” PCF implementation is or is not successful.

**Findings:** A PIP diagram identified the functions and mechanisms from the federal to municipal level by which the PCF is expected to impact families' vulnerabilities, parenting, and, ultimately early childhood outcomes. Six critical quality control pathways (CCP) were identified through the PIP analysis: training and continuing education, quality and intensity of home visits, quality and intensity of intersectoral actions, quality of technical assistance and supervision, process evaluation and monitoring. Whereas quality and intensity of home visits were the CCP most detailed by stakeholders and documents reviewed, intersectoral actions implementation pathways and intended outcomes were unclear. Barriers and facilitators identified through the PIP analysis were mapped across the five constructs of CFIR.

**Implications for D&I Research:** The PIP analysis clarified mechanisms through which PCF activities are linked to intended parenting and ECD outcomes. Understanding how the implementation of CCPs happens across different municipalities is critical to determining PCF barriers and facilitators for implementation success. The matching PIP-CFIR analysis generated a matrix that can be used to explain whether PCF implementation was successful or not across different municipalities.

**Primary Funding Source**

National Institutes of Health

### S95 Implementation of a multi-component intervention to reduce health worker bias toward young people seeking family planning services in Burkina Faso, Pakistan, and Tanzania

#### Corrina Moucheraud^1^, Alexandra Wollum^2^, Willa Friedman^3^, Manisha Shah^4^, William Dow^5^, Zachary Wagner^6^

##### ^1^Health Policy and Management, UCLA Fielding School of Public Health, Los Angeles, CA, USA; ^2^Fielding School of Public Health, University of California, Los Angeles, Los Angeles, CA, USA; ^3^University of Houston, Houston, TX, USA; ^4^Luskin School of Public Affairs, University of California, Los Angeles, Los Angeles, CA, USA; ^5^University of California, Berkeley, Berkeley, CA, USA; ^6^RAND Corporation, Santa Monica, CA, USA

###### **Correspondence:** Corrina Moucheraud (cmoucheraud@ucla.edu)

*Implementation Science 2023,* **18(Suppl 3):**S95

**Background:** Beyond Bias was an intervention to address health workers’ biases toward young women seeking family planning services in Burkina Faso, Pakistan, and Tanzania, by shifting hypothesized drivers of bias including negative attitudes, workplace norms, and low motivation. We sought to understand experiences with implementation of project activities: a large in-person gathering where meaningful stories were shared; ongoing knowledge- and support-building groups; and non-financial awards given quarterly to top-performing facilities.

**Methods:** In 2021, we conducted qualitative interviews at participating health facilities (n=63 clinicians and n=10 managers) and with program implementers (n=38). We gathered data through semi-structured interviews that included questions about experiences with implementation and opinions about scale-up and sustainability. We used the ExpandNet framework to inform coding and analysis, and additional themes emerged from the data. Ethical review was obtained in all study countries and at participating U.S. institutions.

**Findings:** In all three countries, providers found the intervention acceptable and feasible. There were some areas where implementation was less smooth—for example, in Pakistan where the ongoing small group sessions were administered via WhatsApp, this format was seen as burdensome; and some providers who did not win an award were frustrated and felt the criteria were unclear. Particularly in Burkina Faso and Tanzania, there was frequent mention of structural constraints that limited participants’ ability to “translate” lessons from the intervention into real-world changes in their behavior. Examples included lack of space so they could not have adolescent-only waiting areas, stockouts of certain contraceptive commodities; and staff shortages. In all three countries, intervention participants and implementers were enthusiastic about taking the program to scale but had some hesitations including whether there would be sufficient buy-in from communities and other stakeholders. Respondents expressed that scale-up would be more successful and sustainable if the activities were institutionalized or integrated into routine, government-led programs.

**Implications for D&I Research:** Intervention participants in these three very different contexts expressed remarkably consistent views about implementation of the Beyond Bias program. This suggests areas in which complementary investments may facilitate implementation: health system infrastructure and engagement of diverse stakeholders (both pro and anti) even during the “proof of concept” phase.

**Primary Funding Source**

Bill and Melinda Gates Foundation

## Health Policy Dissemination and Implementation Science

### S96 Assessing implementation readiness and sustainability capacity for adopting data disaggregation policies for race/ethnicity variables in new york state datasets

#### Matthew Lee^1^, Lan Ðoàn^2^, Matthew Chin^2^, Farah Kader^2^, Jennifer Pomeranz^3^, Jonathan Purtle^4^, Simona Kwon^5^, Stella Yi^6^

##### ^1^NYU Grossman School of Medicine, New York, NY, USA; ^2^New York University Grossman School of Medicine, New York, NY, USA; ^3^New York University, New York, NY, USA; ^4^Health Management &Policy, New York University School of Global Public Health, New York, NY, USA; ^5^NYU Grossman School of Medicine, New York, USA; ^6^NYU School of Medicine, New York, NY, USA

###### **Correspondence:** Matthew Lee (matthew.lee@nyulangone.org)

*Implementation Science 2023,* **18(Suppl 3):**S96

**Background:** Disaggregated race/ethnicity data are needed to better document and address the health effects of racism, and to measure whether progress is being made towards advancing and sustaining health equity. New York recently passed state law S.6639-A/A.6896-A, requiring state agencies to: 1) collect disaggregated data on Asian Americans/Native Hawaiians/Pacific Islanders; 2) collect data on primary language spoken at home; and 3) report these granular data annually. Research is needed to assess and understand the readiness and capacity of agencies to reach full implementation of the law and identify factors that impede/facilitate implementation success.

**Methods:** Using a community-partnered, participatory approach, we are examining the adoption and implementation of the NY law along multiple dimensions (development, implementation, data cleaning and reporting, documentation). We have completed a review of other state disaggregation efforts, a systematic review of retrospective methods for “cleaning” race/ethnicity in secondary data, and held ongoing meetings with state/local agencies to gather feedback, assess context, and provide technical assistance (TA). We are concurrently conducting focus groups to gather community-level feedback on racial identity formation/self-identification in surveys and trust in completing demographic items.

**Findings:** Our review of state models identified several challenges, including: 1) data suppression guidelines related to privacy protections and release of disaggregated data; 2) limited capacity of current infrastructure to capture and store additional racial/ethnic categories; and 3) inconsistent data practices across agencies. The systematic review of retrospective cleaning methods identified six retrospective “cleaning” methods and the “Asian” and “Hispanic/Latinx” categories as the main populations of focus to apply them. We have conducted >10 TA meetings with the state health department, and >450 individuals have been screened to participate in upcoming focus groups. We find that current data practices and infrastructure systematically exclude and “other” minoritized groups; and meaningful translation of data disaggregation policies into implementable and sustainable systems-level modifications is feasible, but requires significant capacity building.

**Implications for D&I Research:** Our findings build the evidence-base needed to drive future research on scaling and sustaining data disaggregation/data equity infrastructure, policies, and practices across systems, ultimately addressing the dearth of disaggregated data needed to conduct equity-focused implementation research.

**Primary Funding Source**

NYU Global Center for Implementation Science Pilot Awards

### S97 Supporting the inclusion of newly eligible gay, bisexual and all men who have sex with men in plasma donation in canada: a community and health system stakeholder participatory approach

#### Elisabeth Vesnaver^1,2^, Amelia Palumbo^1^, Emily Gibson^1^, Gisell Castillo^1^, Kyle Rubini^3^, Richard MacDonagh^3^, Glenndl Miguel^4^, Marco Reid^4^, Andrew Rosser^3^, Paul MacPherson^1^, Terrie Butler-Foster^5^, Mindy Goldman^5^, Nolan Hill^4,6^, Don Lapierre^5^, Taylor Randall^3^, Wil Osbourne-Sorrell^3^, Joanne Otis^7^, Mark Greaves^4^, Taim Al-Bakri^3^, Max Labrecque^4^, Marc Germain^8^, Shane Orvis^4^, Andrew Clapperton^4^, Dana Devine^9^, Justin Presseau^10^

##### ^1^Ottawa Hospital Research Institute, Ottawa, Canada; ^2^University of Ottawa, Ottawa, Canada; ^3^London community advisory group, London, Canada; ^4^Calgary community advisory group, Calgary, Canada; ^5^Canadian Blood Services, Ottawa, Canada; ^6^Centre for Sexuality, Calgary, Canada; ^7^Université du Québec à Montréal, Montreal, Canada; ^8^Héma-Québec, Québec, Canada; ^9^Canadian Blood Services, Vancouver, Canada; ^10^Ottawa Hospital Research Institute, Ottawa, ON, Canada

###### **Correspondence:** Elisabeth Vesnaver (evesnaver@ohri.ca)

*Implementation Science 2023,* **18(Suppl 3):**S97

**Background:** Increasing domestic collection of blood plasma is a critical public health issue in Canada. Recent sex between men is an exclusion criterion for plasma donation in Canada and elsewhere. Canadian policy has been evolving to include some sexually active men who have sex with men (including but not limited to gay and bisexual men) in plasma donation. While policy changes are necessary for increased donation opportunity in this population, they may not be sufficient to support successful implementation. In this program of research, we aimed to understand which barriers and enablers would impact on plasma donation and to work with stakeholders to co-develop strategies to encourage donation that addresses their needs and concerns.

**Methods:** Rooted in community engagement and integrated knowledge translation, this research was a collaboration between university-based researchers, Canadian Blood Services (blood operator) and community advisors who identified as being impacted by these policies. Following French’s model for designing theory-informed implementation interventions, we used the Theoretical Domains Framework (TDF) and qualitative interviews to identify donation barriers/enablers among men identifying as gay, bisexual or as having sex with men (N=27) and implementation barriers/enablers among donor centre staff (N=28). TDF-linked barriers/enablers were mapped to behaviour change techniques (BCTs). With community advisors, we co-developed suitable strategies to operationalize identified BCTs delivered using feasible and acceptable channels.

**Findings:** BCTs were operationalized using a website and video, and a set of strategies recommended for delivery by Canadian Blood Services spanning staff training, recruitment, communications, stakeholder engagement, and donor centre processes. For example, for delivery by video, 7 TDF domains were mapped to 11 BCTs (e.g. Barrier: concern of being allowed but not welcome in centre [domain: Beliefs about consequences] addressed by showing positive authentic clinic staff interaction with newly eligible male donor [BCT: information about social consequences]).

**Implications for D&I Research:** We co-developed multimodal interventions to support plasma donation by newly eligible men who have sex with men and new criteria implementation by staff. A participatory approach, rooted in theory and lived experience, can support the development of theory-informed interventions that are acceptable and feasible to support a new policy implementation within a contentious policy context.

**Primary Funding Source**

Canadian Blood Services

### S98 Developing strategies to improve the use of evidence for health policymaking: outputs of a stakeholder engagement in nigeria

#### Ejemai Eboreime^1,2^, Adaobi Ezeokoli^3^

##### ^1^University of Alberta, Edmonton, AB, Canada; ^2^National Primary Healthcare Development Agency, Abuja, Nigeria; ^3^Harvard Kennedy School of Government, Cambridge, MA, USA

###### **Correspondence:** Adaobi Ezeokoli (ada_ezeokoli@hks.harvard.edu)

*Implementation Science 2023,* **18(Suppl 3):**S98

Previously Published: https://www.panafrican-med-journal.com/content/article/43/140/full/

### S99 Factors influencing the implementation of mental health recovery into services: findings from a systematic mixed studies review

#### Myra Piat^1^, Megan Wainwright^2^, Eleni Sofouli^3^, Brigitte Vachon^4^, Tania Deslauriers^4^, Cassandra Prefontaine^5^, Francesca Fratti^3^

##### ^1^The Montreal West Island Integrated University Health and Social Services Center (Douglas Mental Health University Institute), Montreal, QC, Canada; ^2^Durham University, Durham, United Kingdom; ^3^McGill University, Montreal, QC, Canada; ^4^University of Montreal, Montreal, QC, Canada; ^5^University of Quebec at Trois Rivieres, Trois Rivières, QC, Canada

###### **Correspondence:** Myra Piat (myra.piat@douglas.mcgill.ca)

*Implementation Science 2023,* **18(Suppl 3):**S99

**Background:** Mental health recovery is increasingly the focus of mental health policy, guidelines, and action plans worldwide. However, no known systematic review, to date, has been published on how recovery has been implemented into services from an implementation science perspective.

**Methods:** We conducted a systematic mixed studies review following a convergent qualitative synthesis design to address the question: How has mental health recovery been implemented into services for adults, and what factors influence the implementation of recovery-oriented services? We applied the best-fit framework synthesis method using the Consolidated Framework for Implementation Research (CFIR). Librarians ran searches in seven databases including Ovid- MEDLINE, Cochrane Library, and Scopus. Two reviewers independently screened studies for inclusion or exclusion. Qualitative, quantitative, and mixed methods peer-reviewed studies published since 1998 were included if they reported a new effort to transform adult mental health services towards a recovery orientation internationally, and reported findings related to implementation experience, process, or factors. The Mixed Methods Appraisal Tool was used to critically appraise all included studies. Data was extracted in NVivo12 to the 38 constructs of CFIR. The synthesis included a within-case and a cross-case thematic analysis of data coded to each CFIR construct. Cases were types of recovery-oriented innovations.

**Findings:** Seventy studies met our inclusion criteria. These were grouped into seven types of recovery-oriented innovations. Common CFIR implementation factors across innovations are: Intervention Characteristics (flexibility, relationship building, lived experience); Inner Setting (traditional biomedical vs. recovery-oriented approach, the importance of organizational and policy commitment to recovery-transformation, staff turnover, lack of resources to support personal recovery goals, information gaps about new roles and procedures, interpersonal relationships), Characteristics of Individuals (variability in knowledge about recovery, characteristics of recovery-oriented service providers); Process (the importance of planning, early and continuous engagement with stakeholders). Very little data was extracted to the outer setting domain, and therefore, we present only some initial observations and note that further research on outer setting implementation factors is needed.

**Implications for D&I Research:** The CFIR required some adaptation for use as an implementation framework in this review. The common implementation factors presented are an important starting point for stakeholders to consider when implementing recovery-oriented services.

**Primary Funding Source**

Canadian Institutes for Health Research Project #KRS 144043

### S100 Enabling responsive and adaptive implementation of evidence-based health policies by assessing implementation readiness of key stakeholders

#### Itzhak Yanovitzky^1^, Cynthia Blitz^2^

##### ^1^Rutgers University, New Brunswick, NJ, USA; ^2^Rutgers University, Somerset, NJ, USA

###### **Correspondence:** Itzhak Yanovitzky (itzhak@rutgers.edu)

*Implementation Science 2023,* **18(Suppl 3):**S100

**Background:** Adoption of evidence-based health policies is often hampered or delayed due to ambiguity regarding potential unintended effects of implementation. Research that documents, assesses, and synthesizes evidence regarding the views and concerns of implementation stakeholders can guide sound decisions regarding adoption and implementation of health policies. We report findings of a comprehensive mixed-methods research project designed to produce and disseminate to state policymakers evidence regarding the acceptability and feasibility of implementing an evidence-based policy—universal screening for adolescent depression—in schools statewide from the perspective of key stakeholders.

**Methods:** Key policy implementation stakeholders were identified in collaboration with a community advisory board. Interviews with samples of school administrators and health professionals (N=15 key-informant interviews and N=70 school psychologists and social workers surveyed) assessed school personnel’s implementation readiness. Inputs from prominent mental health advocacy organizations and professional associations active on this policy were extracted from content analyzing state legislative hearings, reports and testimonials (N=99), local news stories (N=213), and official statements (N=27). A state representative sample of parents of adolescents (N=678) were surveyed to assess their views and concerns regarding implementation.

**Findings:** All implementation stakeholders recognize the preventive utility of screening adolescents for depression but raise different concerns regarding implementation. School personnel are mostly concerned about feasibility of implementation without adequate resources, support and training. Mental health advocates/professionals express significant concerns regarding the suitability of a standard instrument for screening diverse groups of students and about lack of explicit provisions for connecting screening with follow up diagnosis and treatment. Parents are primarily concerned about potentially adverse effects of screening on privacy, treatment, and stigmatizing of students; parents from underserved groups are additionally concerned about not being able to afford the cost of additional evaluation and services. There is direct evidence from tracking different iterations of the legislation that policy was incrementally revised to be responsive to these concerns.

**Implications for D&I Research:** Producing and disseminating research evidence that assesses the policy implementation readiness of key stakeholders can facilitate policy adoption of evidence-based public health guidelines and ensuring responsive and adaptive implementation.

**Primary Funding Source**

William T. Grant Foundation

### S101 Policymakers’ unmet desire for science

#### Adam Levine

##### Johns Hopkins University, Baltimore, MD, USA

###### **Correspondence:** Adam Levine (adamseth@jhu.edu)

*Implementation Science 2023,* **18(Suppl 3):**S101

**Background:** Relationships between researchers and policymakers are vital for research dissemination and evidence-based health policy. Scientific studies find that while some policymakers regularly engage with researchers, many do not. What remains unclear is whether policymakers currently have the relationships they want to be having, or if there is unmet desire to engage with researchers on scientific research relevant to policy challenges they are facing (and, if so, why demand remains unmet and how to meet it). Here I show the value of this approach by focusing on local policymakers and their unmet desire to engage with researchers at colleges and universities in their region.

**Methods:** I fielded a national survey (N=541) of local policymakers via CivicPulse, a nonprofit organization that maintains a dynamically-updated panel of policymakers covering local government positions associated with all townships, municipalities, and counties in the United States, with populations of 1000 or more (98% coverage). I measure prevalence of existing collaborative relationships, desired new ones, and concerns about interacting with local researchers. I also examine results across several theoretically-relevant subgroups: partisanship, gender, age, and degree of local autonomy.

**Findings:**

Unmet desire: Although 73.5% (69.3, 77.3%) had no interaction with local researchers over the past year, 57.0% (52.0, 62.0%) said that they want more collaborative relationships (including a majority of all subgroups).

Why desire remains unmet: Top concerns were that researchers would push a political agenda (47.5% [42.7, 52.3%]), lack practical information (34.1% [29.6, 38.9%]), and not value their expertise as policymakers (17.8% [14.5, 21.7%]).

How to meet desire: Although 78.2% (74.3, 81.7%) report that no local researchers reached out over the past year, 77.6% (72.9, 81.7%) welcome unprompted contact (including a majority of all subgroups).

**Implications for D&I Research:** This study provides substantial evidence that local policymakers have an unmet desire to engage with local researchers to discuss research related to policy challenges they are facing. Meeting this unmet desire is important for using scientific research to address governance challenges. More generally, I argue that we should be explicitly measuring unmet desire among a wider set of decision-makers and using the results to inform dissemination strategies.

**Primary Funding Source**

Johns Hopkins University

### S102 A novel policy alignment and enhancement process to improve sustainment of school-based physical activity programming

#### Penelope Friday^1^, Lexie Beemer^1^, Anna Schwartz^2^, Tiwaloluwa Ajibewa^1^, Michele Marenus^1^, Diane Martindale^3^, Amy Wassmann^4^, Andria Eisman^5^, Thomas Templin^1^, Ronald Zernicke^1^, Lynn Malinoff^6^, Rebecca Hasson^1^

##### ^1^University of Michigan, Ann Arbor, MI, USA; ^2^University of Michigan School of Kinesiology, Ann Arbor, MI, USA; ^3^Birch Run Area Schools, Birch Run, MI, USA; ^4^Saginaw Intermediate School District, Saginaw, MI, USA; ^5^Wayne State University School of Education, Detroit, MI, USA; ^6^Eastern Michigan University, Ypsilanti, MI, USA

###### **Correspondence:** Penelope Friday (Fridaype@umich.edu)

*Implementation Science 2023,* **18(Suppl 3):**S102

**Background:** Strong and comprehensive district wellness policies facilitate the widespread adoption of physical activity (PA) programming in schools. Yet, putting policies into place without aligning them with the context will likely result in poor implementation. Implementation science frameworks can guide the process of aligning policies with the context to support success and sustainment of PA practices. The purpose of this study was twofold: (1) to determine the degree to which a district wellness policy aligned with current PA practices, and (2) to pilot a novel policy alignment and enhancement process to improve policy strength and comprehensiveness through technical assistance.

**Methods:** One school district in central Michigan was selected for local district policy alignment and enhancement. A six-step process was developed that followed the Exploration, Preparation, Implementation, and Sustainment framework phases. *Exploration* included: (1) policy evaluation using the Physical Education (PE) & PA section of the Wellness School Assessment Tool 3.0, and (2) school district self-assessment where the district discussed the current PE and PA practices. *Preparation* included: (3) adding tailored policy language to align the PA policy with current practices, and (4) a district partner workshop to address the feasibility of the policy updates with key partners. *Implementation* included: (5) policy approval and district wide policy implementation. *Sustainment* included: (6) monitoring policy implementation and creating accountability measures for continued improvement.

**Findings:** Initial evaluation of the PA policy revealed a strength score of 19/100 and 38/100 for comprehensiveness. The policy was not aligned with current practices as it included strong language related to PE, but information related to recess, activity breaks, PE substitutions, and after school PA programming was absent despite schools implementing these programs. After completing the enhancement process, alignment of the PA policy with current practices resulted in an 100% increase in strength (score=38/100), and 132% increase in comprehensiveness (score=88/100).

**Implications for D&I Research:** Implementation science frameworks can help guide policy enhancement processes to sustain district-wide PA programming in schools. Future research should examine the adoption and implementation of the policy enhancement process to promote district-wide increases in student PA.

**Primary Funding Source**

Michigan Health Endowment Fund

### S103 Policy and guidance development during the covid-19 pandemic: a systems approach to understanding key dissemination strategies, decision-making processes, and feedback loops between stakeholders

#### Sapna Mendon-Plasek^1^, Elaina Montague^2^, Ana Stefancic^3^, Ana Florence^2^, Iruma Bello^3^, Sapana Patel^4^, Lisa Dixon^5^

##### ^1^RAND Corporation, Boston, MA, USA; ^2^Columbia University, New York, NY, USA; ^3^The New York State Psychiatric Institute and Columbia University, New York, NY, USA; ^4^Columbia University Department of Psychiatry, New York, NY, USA; ^5^New York State Psychiatric Institute, Columbia University Medical Center, New York, NY, USA

###### **Correspondence:** Sapna Mendon-Plasek (smendon@rand.org)

*Implementation Science 2023,* **18(Suppl 3):**S103

**Background:** In response to the COVID-19 crisis, state and local authorities rapidly developed and disseminated guidance to community mental health agencies (CMHAs). While tailored communication and targeted strategies facilitate policy dissemination,^1^ insight into factors that influence decision-making and strategies used to disseminate and facilitate the uptake of guidance amidst a rapidly evolving public health crisis is not well understood. This project sought to understand factors informing decision making and guidance development, and strategies used to disseminate and facilitate guidance uptake among system-level stakeholders in early psychosis programs.

**Methods:** As part of a COVID-19 supplement to capture adaptations to Coordinated Specialty Care services,^2^ semi-structured interviews were conducted with state and local mental health authority leaders (n=9), OnTrackNY implementation specialists (n=12), and OnTrackNY program directors (n=4) to explore changes in guidance. Interviews were analyzed using content analysis.^3^ Code reports relevant to guidance decision-making and dissemination were reviewed to identify emerging themes.

**Findings:** For mental health authorities, decision-making was influenced by changing COVID-19 risk levels, need for alignment between federal and local guidance, and balancing support for CMHA operational continuity with on-going needs for oversight. For OnTrackNY agencies, decision-making was influenced by internal infrastructure and processes (e.g., program autonomy), availability of resources (e.g., technology), and perspectives on managing risk and uncertainty (e.g., COVID-19, regulatory waiver expiration). Dissemination of guidance comprised active (e.g. daily calls with state authority) and passive strategies (e.g., FAQ sheets). Information flow was bidirectional such that top-down dissemination of guidance (e.g., from state mental health authorities to providers) was informed and refined with bottom-up feedback (e.g., from providers to state leadership) through surveys, town halls, and direct communication) to facilitate guidance uptake.

**Implications for D&I Research:** Unlike engaging usual planned strategies to disseminate new policies, public health emergencies warrant a deeper understanding of how guidance may be reconciled, refined, and adapted to fit rapidly evolving stakeholder needs to facilitate their use. Findings may inform efforts to identify mechanisms and processes that contribute to a feedback loop and the adaptation of guidance during future public health emergencies.

**Primary Funding Source**

National Institutes of Health

## Models, Measures, and Methods

### S104 A scoping review of frameworks in empirical studies and a review of dissemination frameworks

#### Ana Baumann^1^, Cole Hooley^2^, Emily Kryzer^3^, Alexandra Morshed^4^, Cassidy Gutner^5^, Sara Malone^6^, Callie Walsh-Bailey^1^, Meagan Pilar^7^, Brittney Sandler^3^, Rachel Tabak^8^, Stephanie Mazzucca^9^

##### ^1^Washington University in St. Louis, St. Louis, MO, USA; ^2^Brigham Young University, Provo, USA; ^3^Washington University in St. Louis, St. Louis, USA; ^4^Emory University, Decatur, GA, USA; ^5^ViiV Healthcare, Research Triangle Park, NC, USA; ^6^Washington University in St. Louis, Saint Louis, MO, USA; ^7^Washington University in St. Louis, St Louis, MO, USA; ^8^Washington University in Saint Louis, Saint Louis, MO, USA; ^9^Prevention Research Center, Washington University in St. Louis, St. Louis, MO, USA

###### **Correspondence:** Ana Baumann (abaumannwalker@wustl.edu)

*Implementation Science 2023,* **18(Suppl 3):**S104

**Background**

The field of dissemination and implementation (D&I) research has grown in recent years. However, the field of dissemination research has not coalesced to the same degree as the field of implementation research. To advance the field of dissemination research, this review aimed to: (1) identify the extent to which dissemination frameworks are used in dissemination empirical studies, (2) examine how scholars define dissemination, and (3) identify key constructs from dissemination frameworks.

**Methods**

To achieve aims 1 and 2, we conducted a scoping review of dissemination studies published in D&I science journals. The search strategy included manuscripts published from 1985 to 2020. Articles were included if they were empirical quantitative or mixed methods studies about the dissemination of information to a professional audience. Studies were excluded if they were systematic reviews, commentaries or conceptual papers, scale up or scale out studies, qualitative or case studies, or descriptions of programs. To achieve aim 1, we compiled the frameworks identified in the empirical studies. To achieve aim 2, we compiled the definitions from dissemination from frameworks identified in aim 1 and from dissemination frameworks identified in a 2021 review. To achieve aim 3, we compile the constructs and their definitions from the frameworks.

**Findings**

Out of 6017 studies, 89 studies were included for full-text extraction. Of these, 45 (51%) used a framework to guide the study. Across the 45 studies, 34 distinct frameworks were identified, out of which 13 (38%) defined dissemination. There is a lack of consensus on the definition of dissemination. Altogether, we identified 48 constructs, divided into 4 categories: Process, Determinants, Strategies, and Outcomes. Constructs in the frameworks are not well defined.

**Implications for D&I Research**

This study provides a critical step in the dissemination research literature by offering suggestions on how to define dissemination research, and by cataloging and defining dissemination constructs. We will provide a critique and reflection about the dissemination literature and offer suggestions on how to strengthen these definitions and distinctions between D&I research to advance the field of dissemination research.

**Primary Funding Source**

National Institutes of Health

### S105 The priority aims and testable hypotheses (path) for implementation research: a scoping review

#### Bryan Garner^1^, Sheila Patel^2^, Sarah McDaniel^2^, Jackie Mungo^2^

##### ^1^The Ohio State University, Columbus, OH, USA; ^2^RTI International, Research Triangle Park, NC, USA

###### **Correspondence:** Bryan Garner (bryan.garner@osumc.edu)

*Implementation Science 2023,* **18(Suppl 3):**S105

**Background:** Eccles and Mittman (2006) defined implementation research as “the scientific study of methods to promote the systematic uptake of research findings and other evidence-based practices into routine practice, and, hence, to improve the quality and effectiveness of health services and care.” Similarly, the National Institute of Health has defined implementation research as “the scientific study of the use of strategies to adopt and integrate evidence-based health interventions into clinical and community settings in order to improve patient outcomes and benefit population health.” Guided by these definitions, existing implementation research, general principles of data reduction, and a general framework for moderated mediation, we identified three priority aims and four priority testable hypotheses to advance generalizable knowledge. This presentation will present results of a scoping review to identify articles that have addressed the priority aims and testable hypotheses (PATH) for implementation research.

**Methods:** Using the five-stage approach developed by Arksey and O’Malley (2005) and advanced by Levac, Colquhoun, and O’Brien (2010), we conducted a scoping review of all research articles and short reports published between 2006 and 2020 in either *Implementation Science, Implementation Science Communications,* and *Implementation Research and Practice.*

**Findings:** Of the 862 articles identified and coded, 43 (5%) assessed a PATH for implementation research. Advancing generalizable knowledge about the relationship between an implementation strategy and a health or health-related outcome (path c) was the most addressed priority aim, with 32 articles identified. Regarding the priority testable hypotheses, we identified 34 articles that tested an effectiveness hypothesis from a superiority trial, and 1 article that tested a cost-effectiveness hypothesis from a non-inferiority trial.

**Implications for D&I Research:** The PATH for implementation research were examined by few articles identified in key implementation-focused journals. To help the field develop one or more scientific theories as defined by the National Academy of Sciences (i.e., a comprehensive explanation of the relationship between variables that is supported by a vast body of evidence), there is an urgent need for more PATH-centered implementation research.

**Primary Funding Source**

National Institutes of Health

### S106 Toward a more comprehensive understanding of organizational influences on implementation: The organization theory for implementation science (OTIS) framework

#### Sarah Birken^1^, Jennifer Leeman^2,3^, Linda Ko^4^, Alexandra Peluso^1^, Cheyenne Wagi^1^, Mary Wangen^5^, Maria E. Fernandez^6^, Manal Masud^4^, Terry Huang^7^, Matthew Lee^8^, Grace Ryan^9^, Prajakta Adsul^10^, Mimi Choy-Brown^11^, Jure Baloh^12^, Michelle C. Kegler^13^, Hannah Arem^14^

##### ^1^Wake Forest University School of Medicine, Winston-Salem, NC, USA; ^2^School of Nursing, University of North Carolina, Chapel Hill, NC, USA; ^3^Lineberger Comprehensive Cancer Center, Chapel Hill, NC, USA; ^4^University of Washington, Seattle, WA, USA; ^5^University of North Carolina, Chapel Hill, NC, USA; ^6^The University of Texas Health Science Center at Houston School of Public Health, Houston, TX, USA; ^7^The City University of New York, New York, NY, USA; ^8^NYU Grossman School of Medicine, New York, NY, USA; ^9^University of Massachusetts Chan Medical School, Worcester, MA, USA; ^10^University of New Mexico, Albuquerque, NM, USA; ^11^University of Minnesota, St. Paul, MN, USA; ^12^University of Arkansas for Medical Sciences, Little Rock, AR, USA; ^13^Rollins School of Public Health, Atlanta, GA, USA; ^14^MedStar Health Research Institute, Hyattsville, MD, USA

###### **Correspondence:** Sarah Birken (sbirken@wakehealth.edu)

*Implementation Science 2023,* **18(Suppl 3):**S106

**Background:** Theoretical frameworks contribute to understanding and addressing evidence-based practice (EBP) implementation by synthesizing multiple theories’ constructs. For example, the Theoretical Domains Framework synthesizes constructs from 33 psychological theories for implementation scientists’ use. Similar frameworks do not exist for organization theories, which explain how and why organizations adopt, implement, and sustain EBP use. Although their utility is increasingly acknowledged, organization theories remain underused in implementation science. To advance their use among implementation scientists, we synthesized organization theory constructs in the Organization Theory for Implementation Science (OTIS) framework.

**Methods:** We recruited organization and implementation scientists to participate in an online concept mapping exercise in which they sorted 70 constructs from 9 theories identified in our previous work into domains representing similar theoretical concepts. Participants also used a five-point scale to rate each construct’s influence on implementation and potential for modification. Multidimensional scaling and hierarchical cluster analyses were used to produce visual representations of the relationships among the constructs in concept maps. To interpret concept maps, we engaged members of the Cancer Prevention and Control Research Network OTIS workgroup.

**Findings:** Twenty-five experts participated in concept mapping. OTIS workgroup members selected the 10-cluster solution based on included construct groupings’ coherence. Workgroup members then reorganized clusters to increase coherence, yielding 8 final OTIS framework domains: organizational dynamics (e.g., inertia); organizational structure (e.g., size); internal processes (e.g., feedback loops); tasks and technology (e.g., transaction costs); knowledge/insight (e.g., sensemaking); interorganizational relationships (e.g., coercive pressure); organizational field characteristics (e.g., selection pressure); networks/ties (e.g., cohesion).

**Implications for D&I Research:** We will present a detailed description of our synthesis of 70 constructs from 9 organization theories into 8 domains. The OTIS framework has the potential to increase awareness and use of key concepts from organization theories among implementation scientists. Applications of the OTIS framework will enhance understanding of organizational influences on EBP implementation, promote theory-driven strategies for organizational change, and allow for refinement of the framework, which we view to be a living tool to be improved through application. Next steps include testing the OTIS framework in implementation research and adapting it for use among policymakers and practitioners.

**Primary Funding Source**

Centers for Disease Control and Prevention

### S107 The implementation in context (icon) framework: a meta-framework of context for dissemination and implementation science

#### Janet E. Squires^1^, Ian D. Graham^1,2^, Alison M. Hutchinson^3^, Wilmer J. Santos^4^, Shelly A. Li^5^, Melissa Demery Varin^6^, ICON Team^6^

##### ^1^Ottawa Hospital Research Institute, Ottawa, Canada; ^2^University of Ottawa, Ottawa, Canada; ^3^Barwon Health, Geelong, Australia; ^4^The Ottawa Hospital Research Institute, Ottawa, ON, Canada; ^5^Al & Malka Green Artists' Health Centre, Toronto, Canada; ^6^University of Ottawa, Ottawa, ON, Canada

###### **Correspondence:** Janet E. Squires (janet.squires@uottawa.ca)

*Implementation Science 2023,* **18(Suppl 3):**S107

**Background:** Context modifies the effects of dissemination and implementation strategies to increase healthcare professionals' use of research evidence in clinical practice. However, conceptual clarity about what comprises "context" is lacking. The purpose of this study was to develop a meta-framework of context domains, attributes, and features relevant to dissemination and implementation.

**Methods:** We conducted a meta-synthesis of data from three interrelated studies on context: 1) a concept analysis of published literature on context (n=70 studies), 2) a secondary analysis of healthcare professional interviews (n=145) examining context across 11 unique studies, 3) a descriptive qualitative study comprised of interviews heath system stakeholders (n=39) to elicit their tacit knowledge on the attributes and features of context that are important for improved research use by healthcare professionals. A rigorous protocol was followed for the meta synthesis. Following this synthesis across studies, ICON was further refined through feedback from experts in context and implementation science.

**Findings:** In ICON, context is conceptualized in 3 levels: micro (individual), meso (organizational), and macro (external). The three levels are comprised of 6 contextual domains: 1) actors (micro), 2) organizational climate and structures (meso), 3) organizational social behaviour (meso), 4) organizational response to change (meso), 5) organizational processes (meso), and 6) external influences (macro). These 6 domains contain 22 core attributes of context and 108 features that illustrate these attributes.

**Implications for D&I Research:** ICON is the only implementation meta-framework of context available to guide dissemination and implementation efforts of knowledge users and researchers. It provides a comprehensive and critically needed understanding of the context domains, attributes and features relevant to healthcare professionals’ use of research in clinical practice. ICON will assist with the development of common assessment tools to measure context to tailor dissemination and implementation intervention design and delivery. It can also be used to better interpret the effects of dissemination and implementation interventions, and to pragmatically guide knowledge users in their implementation efforts.

**Primary Funding Source**

Canadian Institutes of Health Research (CIHR)

### S108 Iterative prism (i-prism): background, rationale, key functions and early results

#### Russell Glasgow

##### University of Colorado School of Medicine, Aurora, CO, USA

###### **Correspondence:** Russell Glasgow (russell.glasgow@cuanschutz.edu)

*Implementation Science 2023,* **18(Suppl 3):**S108

**Background:** There have been consistent calls for implementation science to be more conceptually based, pragmatic, rapid and nimble, evidence-based, and have high levels of engagement and multi-sector collaboration. It is difficult to simultaneously meet all these objectives. The Iterative Practical Robust Implementation and Sustainability Model (PRISM which includes the Reach, Effectiveness, Adoption, Implementation, Maintenance (RE-AIM) outcomes) that we refer to as I-PRISM is designed to address many of these aspirational goals in a feasible implementation package that can be used to guide adaptations during planning, implementation, and sustainment.

**Methods:** Preliminary work in multiple VA settings demonstrated that an iterative approach based on RE-AIM was feasible, well received, and applicable across a wide range of different projects, teams, and content areas. We then developed I-PRISM which is a contextual expansion of Iterative RE-AIM that specifies key contextual factors to consider when evaluating data on RE-AIM outcomes. The key functions involved in I-PRISM are: educate teams on use of PRISM to set priorities and evaluate progress; obtain independent input from team members; summarize results in visual displays showing differences between priorities and progress; facilitate team discussion and goal setting; collaboratively develop and evaluate adaptations; and periodically repeat this process.

**Findings:** We will summarize three types of results across the presentations: RE-AIM outcomes prioritized by implementation teams; areas of greatest gaps between priorities and progress; and data on PRISM contextual factors related to RE-AIM outcomes. Across projects, the RE-AIM outcome with the greatest gap between priority and progress was Reach; the areas in which adaptations were made most often were Reach and Implementation.

**Implications for D&I Research:** Based upon feasibility work in multiple settings we have developed a conceptually based and data driven implementation strategy bundle that aids implementation teams in responding to changing context, priorities, and level of progress on different outcomes. This I-PRISM package, or variants of it, have been applied in several ongoing projects described in other panel presentations and integrated into an interactive webtool described in the final presentation. There are needs for improvement, replication, and comparative effectiveness research, but I-PRISM appears to address many of the implementation science challenges outlined above.

**Primary Funding Source**

Department of Veterans Affairs

### S109 Iterative use of re-aim/prism in a hypertension control trial in guatemala

#### Meredith Fort

##### Colorado School of Public Health, Aurora, CO, USA

###### **Correspondence:** Meredith Fort (meredith.fort@cuanschutz.edu)

*Implementation Science 2023,* **18(Suppl 3):**S109

**Background**

Uncontrolled hypertension presents a substantial burden in Guatemala and other low- and middle-income countries. In 2019, the Guatemalan Ministry of Health (MOH) began implementing a multi-component program to improve hypertension control in rural communities, using a type 2 hybrid effectiveness-implementation design. RE-AIM/PRISM was selected as the guiding D&I framework.

**Methods:** Prior to implementation, we conducted a multi-methods needs assessment to capture perspectives at different levels within the Guatemalan public primary care system and rural communities. We developed implementation tracking forms that were filled out by implementers (MOH staff; primarily auxiliary nurses). Local-level evaluators captured data using forms to assess key aspects of context within health posts (availability of medications, blood pressure monitors, and staff turnover). The study team met regularly with the MOH to be aware of broader contextual changes. During the COVID-19 pandemic the study team made phone calls to implementers and patients to gain insight into their experiences and to inform adaptations. Qualitative assessment of PRISM domains and RE-AIM outcomes prior to, during and post-implementation complemented routine implementation and patient assessments.

**Findings:** Routine assessment of medication availability was identified as a top priority. The study team reviewed and reflected on changes in implementation and medication availability, and discussed staff turnover and implications for the PRISM Implementation and sustainability infrastructure domain; these discussions usually led to reaching out to different actors in the MOH at the central, provincial, or local levels. We reviewed Reach during initial meetings and determined it would be difficult to influence in the short-term. The COVID-19 pandemic resulted in restrictions to public transportation, reduction in face-to-face meetings with providers, and additional responsibilities for health workers. Priority adaptations included: a change in how training was conducted and increased flexibility in providing medications. Broader contextual factors were also discussed by implementers.

**Implications for D&I Research:** To capture changes in the context and program implementation, it was important to assess RE-AIM and PRISM components on a regular basis. While some components such as reach, representativeness, and system-level capacity may be challenging to influence in the short term, they are important to capture and understand to promote equitable long-term participation and delivery.

**Primary Funding Source**

National Institutes of Health

### S110 Using i-prism with re-aim dashboard to speed implementation of lung ultrasound in the management of patients with covid-19

#### Anna Maw

##### University of Colorado School of Medicine, Aurora, CO, USA

###### **Correspondence:** Anna Maw (anna.maw@cuanschutz.edu)

*Implementation Science 2023,* **18(Suppl 3):**S110

**Background:** In early 2020, the pandemic heightened the need for rapid implementation of new inpatient practices to cope with the high volume of patients admitted for COVID-19. In this context, point of care lung ultrasound (LUS) was seen as a promising alternative to traditional radiology-performed chest imaging.

**Methods:** We performed an implementation pilot study at a single academic center to rapidly implement LUS among hospitalists caring for patients admitted with COVID-19. Given the urgency of the pandemic, we sought an approach that would: 1) offer rapid real time data to monitor the progress of implementation, and 2) rapid assessment of contextual barriers using I-PRISM to guide adaptations to our implementation strategies. Using a convergent mixed methods design, we developed a novel ‘RE-AIM Dashboard’ which displayed quantitative RE-AIM outcomes prioritized by our hospitalist implementers using data extracted from the EHR and was automatically updated every 48 hours. In addition, we used I-PRISM to qualitatively assess contextual barriers to implementation through hospitalist interviews. In bi-weekly implementation team meetings, we jointly considered emerging trends in quantitative Reach and Adoption rates and qualitative I-PRISM barriers to guide decisions on planned adaptations to our implementation strategies.

**Findings:** Over a one-year period, n=24 meetings were conducted. Over this period, Reach ranged from 0% to 2%, and order Adoption rose from 0% to 50%. Key I-PRISM barriers such as limited dedicated time for hospitalist training led to the subsequent deployment of six sequential implementation strategies and modest increases of LUS integration into clinical practice. Once built by our information technology team, the Iterative RE-AIM Dashboard provided automated updates regarding the extent and representativeness of Reach and Adoption without additional staff resources.

**Implications for D&I Research:** We found I-PRISM in conjunction with a RE-AIM operations Dashboard was a highly feasible and low-burden way to rapidly and repeatedly evaluate implementation progress, assess for new or persistent barriers, and identify any disparities in Reach. Given the growing availability of dashboards to display health system data, our findings suggest I-PRISM used in conjunction with a RE-AIM dashboard is a promising and feasible means of monitoring implementation progress and informing mid-course adaptations.

**Primary Funding Source**

National Institutes of Health

### S111 An interactive, visual webtool to guide the pragmatic and iterative use of I-PRISM

#### Katy Trinkley

##### University of Colorado Anschutz Medical Campus, Aurora, CO, USA

###### **Correspondence:** Katy Trinkley (Katy.trinkley@cuanschutz.edu)

*Implementation Science 2023,* **18(Suppl 3):**S111

**Background:** To speed research translation, many have called for ways to make implementation science methods and models more accessible and to provide more concrete guidance for researchers and practitioners. In response to this need, we created an interactive webtool to guide both English and Spanish speaking users from diverse backgrounds through the process of applying PRISM to nimbly adapt programs during planning, implementation, and sustainment.

**Methods:** We used a human-centered design process and iteratively engaged potential users who were in various phases of implementing different types of programs. Multisector engagement included native English and Spanish speaking individuals and implementation teams from government, community, public health, academic, and healthcare settings. The goal was to create a user-friendly, interactive tool that facilitated systematic and flexible assessment of a program’s contextual alignment using the PRISM context domains and the pragmatic RE-AIM outcomes. Based on this information, the tool would then guide development of feasible and impactful adaptations across all implementation phases. Iterative user testing using low fidelity mockups and the think aloud method supported co-creation of the content, wording, navigation, visual displays, and overall usability of the webtool.

**Findings:** We will provide a demonstration of the webtool. The I-PRISM webtool is designed to be used by individuals or implementation teams and includes a set of assessment items aligned with PRISM context and RE-AIM outcomes that were refined through multisectoral engagement. The output based on answers to the assessment items is presented in graphical and tabular visual displays. After reviewing the output, users are prompted to develop and select adaptation action plans they estimate to be both feasible and impactful. Users are encouraged to download their results and use the webtool iteratively over a program’s life cycle. Considerations for equity are integrated throughout the webtool.

**Implications for D&I Research:** The I-PRISM webtool is a public good co-created with multisector engagement to guide the use of PRISM for iterative adaptations across the life cycle of a program. The webtool was designed to be generalizable across diverse settings and programs and will be refined over time to maximize ease of use.

**Primary Funding Source**

National Institutes of Health

### S112 Commonality and co-occurrence of discrete strategies within implementation strategy bundles: results from a living review of global hiv implementation research

#### Sita Lujintanon

##### Johns Hopkins School of Public Health, Baltimore, MD, USA

###### **Correspondence:** Sita Lujintanon (slujint1@jhu.edu)

*Implementation Science 2023,* **18(Suppl 3):**S112

**Background**

Health services and innovations are delivered through implementation strategy bundles that are often complex, comprising numerous discrete strategies. Detailed assessment of the usage patterns of different types of discrete strategies within real-world strategy bundles would enable classification of discrete strategies based on commonality and co-occurrence. We leveraged the Living Database of HIV Implementation Science (LIVE) to describe patterns of discrete strategy usage within published implementation strategy bundles.

**Methods**

A systematic review was conducted to include any studies published from 2004-2021 in any low- and middle-income country (LMIC) that described implementation, including strategies, and reported ≥1 HIV cascade outcome. Implementation strategies were inductively specified according to actor, action, and action target.

**Findings**

Between January 2014-July 2022, 44,126 abstracts were screened, 555 studies met inclusion criteria in which 3,315 discrete implementation strategies were identified. The median number of reported strategies per study was 4 (1-13); 88.8% of studies reported using multiple strategies. The most common actors were researchers (48.8%), unspecified health providers (42.0%), and health associate professionals (e.g. counselors, community health workers, lay health workers; 41.8%). The most common action targets were people living with HIV (78.6%), health system (54.1%), and unspecified provider (29.5%). The most common action was providing education on a health innovation/service/behavior (365 studies; 65.8%). Many studies using this action also used training to learn a new skill (35.1%), providing community-based services (32.3%), and providing psychosocial support counseling (22.7%). The second most common action was training to learn a new skill (180 studies; 32.4%). Many studies using this action also used providing education on a health innovation/service/behavior (71.1%), supervising/mentoring/coaching/facilitating (37.8%), and providing community-based services (33.9%).

**Implications for D&I Research**

This large and comprehensive review of HIV-related implementation research from LMICs found that discrete implementation strategies are very frequently used in combination and feature multiple actors, actions, and action targets. This expands our understanding of how strategies are being reported and used in published research, and calls for improved strategy bundle specification and taxonomy. Further research should also assess and optimize strategy bundles, including uncommon and underused strategies, to inform effective, transferable health service delivery approaches.

**Primary Funding Source**

Bill and Melinda Gates Foundation

### S113 Methods for implementation science systematic reviews

#### Noelle Le Tourneau

##### Washington University in St. Louis, St. Louis, MO, USA

###### **Correspondence:** Noelle Le Tourneau (lnoelle@wustl.edu)

*Implementation Science 2023,* **18(Suppl 3):**S113

**Background**

Evidence synthesis tools have been primarily designed for trials evaluating efficacy; there are no guidelines for the synthesis of implementation research. Implementation science relies on measures and study designs that reflect real-world scenarios that are often mixed methods, including pragmatic trials, observational, preference, and qualitative evidence. To inform implementation guidelines and policies, evidence syntheses should incorporate a broad set of study designs, appraisal tools, and implementation science frameworks.

**Methods**

We searched the literature, conferred with experts, and tested tools to create a method for conducting implementation systematic reviews, incorporating implementation science frameworks into routine systematic review methods. We compiled a set of tools to characterize implementation strategies, assess implementation outcomes, implementation trial types, and evaluate and classify pragmatism in RCTs. We assembled tools to assess the methodological quality of RCTs, natural experiments, cohort, cross-sectional, qualitative, preference, and mixed methods studies within implementation science reviews.

**Findings**

We identified 10 tools that, in combination, assessed implementation and methodological quality of implementation research, as well as provided mixed method systematic reviews synthesis guidelines. Four tools characterized implementation components: a modified version of Proctor and TIDieR tools to classify implementation strategies, the Proctor et. al framework to characterize and assess implementation outcomes, a modified version of the PRECIS-2 tool to evaluate pragmatism in trials, and Curran’s framework to characterize implementation trials. We also identified five tools to assess methodological quality of all study designs central to implementation science reviews. These include the Cochrane Risk of bias tools (ROB-2) for RCTs, the Newcastle Ottawa scale for observational (cohort, cross-sectional, natural experiments, quasi-experimental) studies, the Joanna Briggs Institute (JBI) critical appraisal checklist for qualitative research, the ISPOR checklist for preference studies, and the mixed methods appraisal tool (MMAT). We further identified the JBI guidelines for data synthesis and integration in mixed methods systematic reviews.

**Implications for D&I Research**

This set of tools will allow investigators to assess methodological quality and synthesize evidence from a variety of implementation science study designs to appropriately inform implementation. Establishing best practices for implementation science evidence synthesis including consistent methodology, language, and reporting standards in implementation systematic reviews is crucial to advance the field of implementation science.

**Primary Funding Source**

Bill and Melinda Gates Foundation

### S114 Reporting and measures of implementation outcomes from hiv-related implementation research grants

#### Sheree Schwartz

##### Johns Hopkins University, Washington, DC, USA

###### **Correspondence:** Sheree Schwartz (sschwartz@jhu.edu)

*Implementation Science 2023,* **18(Suppl 3):**S114

**Background**

While the landscape of HIV-related implementation research (IR) funding has grown in recent years, less is known about reporting frequency and measures of implementation outcomes (IOs) within HIV IR.

**Methods**

We leveraged a previous landscaping analysis of all NIH-funded, HIV-related IR grants from 2013-2017. All publications linked to these awards in NIH RePORTER through January 1, 2021 were screened for whether they were original research publications reporting data emanating directly from the funded grant. Publications derived from the awards were reviewed and IOs identified per Proctor’s Implementation Outcomes taxonomy, as well as the ‘Reach’ outcome from RE-AIM. We describe grant- and paper-level findings.

**Findings**

Among 215 HIV-related IR NIH-funded grants, 59.0% (n=127) had published original research results by January 2021, resulting in 431 publications. Overall, 119/431 (27.6%) publications reported any IOs, representing IOs from 61/215 (28.4%) funded grants and 61/127 (48.0%) grants with publications. On average, grants with any publications reported a mean of 0.9 [sd:1.4] IOs. Among the 119 publications reporting IOs, the mean number per publication was 1.7 (sd:0.9, range:1-5). The outcomes most commonly reported were acceptability (n=75 papers; 35.4% [45/127] of grants with publications), appropriateness (n=39 papers; 19.7% [25/127] grants), feasibility (n=29 papers; 15.8% [20/127] grants), cost (n=20 papers; 5.5% [7/127] grants), adoption (n=16 papers; 11.0% [14/127] grants), and fidelity (n=13 papers; 9.5% [12/127] grants); penetration, sustainability and reach were reported in ≤5 papers each. Among the three most reported IOs, most acceptability (71%) and appropriateness (85%) outcomes were measured qualitatively, whereas 69% of feasibility outcomes were assessed quantitatively. The proportion of papers reporting IOs varied by EPIS phases, with IO reporting by 7.3% (n=16/220), 42.4% (n=50/118), 57.1% (n=48/84) and 55.6% (n=5/9) of papers at the ‘Exploratory’, ‘Preparatory’, ‘Implementation’ and ‘Sustainment’ phases, respectively (p<0.001). When considering grant mechanisms, 22% of R34-awards reported an IO, 27% of K-awards, 29% of R01-awards and 47% of R21-awards (p=0.14).

**Implications for D&I Research**

Overall, fewer than one-third of papers and grants reported IOs, though further publications may be forthcoming. Increased reporting of IOs including adoption, fidelity and other later-stage IOs improves the interpretation of effectiveness data and ultimately, supports the optimal impact of real-world implementation.

**Primary Funding Source**

Bill and Melinda Gates Foundation

### S115 Adaptation of implementation strategies: a mechanistic framework based on literature review

#### Elvin Geng

##### Washington University in St. Louis, St. Louis, MO, USA

###### **Correspondence:** Elvin Geng (elvin.geng@wustl.edu)

*Implementation Science 2023,* **18(Suppl 3):**S115

**Background**

Many implementation targets (e.g., health care workers, patients or organizations) differ from each other but yet at the same time are not absolutely unique. While design of implementation strategies can account for some of these differences at the onset, changes over time (and differences in those changes across settings) are unavoidable. Implementation strategies may need to adapt to optimize their intended effects.

**Methods**

We use a mechanistic review in which we conducted a search for adaptations in implementation strategies and extracted the components and causal relationships of these adaptations. We use a simple directed acyclic graph (DAG) to represent concepts as nodes and potential causal effects as arrows. We adhere to the convention in which two arrows pointing into a node implies effect modification on at least one scale.

**Findings**

Our diagram suggests adaptation of implementation strategies require three necessary steps. First, implementation strategies have effects on intended implementation outcomes. Second, these effects in turn modify, alter, intensify, or change in other ways the strategy used. Third, the modified strategy then itself has an effect on implementation outcomes. These three steps must be present for adaptation to be present, and may or may not be accompanied by initial responses’ effects on final responses independently of the changes incurred or the initial strategy’s effect on *the effects* of the changed strategy – that is to say that an altered strategy’s effects could be constrained or potentiated by the initial approach. The initial strategy also has effects on the final outcomes that are in part independent of change in the strategy.

**Implications for D&I Research**

A mechanistic examination of the adaptation of implementation strategies provides several novel conceptual insights. First, the effects of an *adapted* strategy differ from the effects of an *adaptive* strategy together. Second, adaptive implementation strategies may also be accompanied by non-adaptive pathways. Third, adaptations are themselves a consequence of initiation strategies deployed, and the effects of adaptations are influenced by the initial actions. The diagram also points out classes of research questions about adaptations of implementation strategies such as dosing, mechanism of adaptation, thresholds for change, and interaction.

**Primary Funding Source**

Bill and Melinda Gates Foundation

### S116 Illuminating the impacts of outer context on implementation

#### Ms. Gracelyn Cruden^1^, Holle Schaper^1^, Shelley Crawford^1^, Dylan Wong^2^, Lisa Saldana^1^

##### ^1^Oregon Social Learning Center, Eugene, OR, USA; ^2^University of South Carolina, Columbia, SC, USA

###### **Correspondence:** Gracelyn Cruden (gracelync@oslc.org)

*Implementation Science 2023,* **18(Suppl 3):**S116

**Background**

The role of outer context on implementation success repeatedly has been theorized and demonstrated. Yet, most theories and frameworks refer to an amorphous “outer context” without further specification Unsurprisingly then, the impact of outer context on implementation often is not measured or tracked during implementation, limiting opportunities to proactively design implementation strategies that might mitigate known or unforeseen outer context events. This study sought to: 1) identify common outer context dimensions in implementation frameworks and define additional dimensions; 2) specify and track how outer context is impacting ongoing implementation efforts using a new Outer Context Module to accompany the Stages of Implementation Completion (SIC), a validated measure of implementation fidelity.

**Methods**

A preliminary outer context taxonomy was created by reviewing implementation frameworks (e.g., CICI, EPIS, CFIR). Next, a scoping review was undertaken to expand the taxonomy. The review explored how outer context impacts are theorized, evaluated, and described in the peer-reviewed and grey literature.

The SIC Outer Context Module was informed by the preliminary taxonomy, experts in implementation fidelity monitoring with the SIC, and data from a pilot module. The Module includes: Topics (to categorize Outer Context Events within the Outer Context taxonomy), levels at which Events occur (i.e., National, regional, local, service system), and implementation Effects (e.g., in-person trainings paused).

**Findings**

Eight dimensions initially were identified: implementation processes at other sites; resources from other implementations; eligible population in community; policy and politics; funding, contracting; natural disaster; social, ethical, cultural; leadership. The scoping review identified a new dimension—Infrastructure. Pilot Outer Context Module data includes 49 Effects from 37 Events across 10 implementations. Two Topics emerged from pilot data: Infectious Disease Outbreak; Workforce Challenges. Most Events were categorized as policy or politics (33%), 85% of which related to COVID-19. Events had positive, neutral, and higher barrier Effects. Effects often entailed modifying implementation timelines (e.g., delaying training, delaying in-person site visit).

**Implications for D&I Research**

Increasing specificity in how outer context is defined can improve monitoring and generalizable measurement of how context affects implementation. Measuring outer context impacts can increase understanding of how implementation efforts can plan for and successfully overcome outer context challenges with targeted strategies.

**Primary Funding Source**

National Institutes of Health

### S117 Using the framework for reporting adaptations and modifications to evidence-based implementation strategies (frame-is) to document modifications to an adaptive implementation strategy

#### Rose Garza-Hennessy^1^, Nicholas Schumacher^2^, Nora Jacobson^3^, Andrew Cohen^4^, Jilian Landeck^3^, Paul Hunter^3^, Morgan Burns^5^, Andrew Quanbeck^5^

##### ^1^University of Wisconsin-Madison, Madison, WI, USA; ^2^University of Wisconsin - Madison, MADISON, WI, USA; ^3^University of Wisconsin, Madison, WI, USA; ^4^Bellin Health, Green Bay, WI, USA; ^5^Department of Family Medicine and Community Health, University of Wisconsin – Madison, Madison, WI, USA

###### **Correspondence:** Andrew Quanbeck (arquanbe@wisc.edu)

*Implementation Science 2023,* **18(Suppl 3):**S117

**Background:** The strategies used to implement evidence-based practices (EBPs) often require modifications. A systematic approach to documenting such modifications has not yet been widely adopted. The 2021 FRAME-IS is a novel framework that allows researchers to characterize both proactive and reactive changes to implementation strategies. Few publications have demonstrated the application of the FRAME-IS. The Balanced Opioid Initiative, an NIH-funded trial that tested the use of systems consultation to promote adherence to the CDC Guideline for Prescribing Opioids for Chronic Pain, provided a timely opportunity to assess the utility of the FRAME-IS.

**Methods:** An interdisciplinary team of researchers and implementers met to document modifications across core modules of the FRAME-IS for the four ISs that make up the package of systems consultation used in the Balanced Opioid Initiative: (1) Audit & Feedback; (2) Educational Meetings; (3) Practice Facilitation; and (4) Prescriber Peer Consulting. Modifications were necessary due to COVID-19, the rise of telemedicine, the changing landscape of opioid prescribing, and variations between healthcare systems.

**Findings:** The Balanced Opioid Initiative was implemented in 32 clinics within two healthcare systems in a Midwestern state using a Sequential, Multiple-Assignment Randomized Control Trial (SMART). One to three modifications were described for each IS. All seven core modules of the FRAME-IS were completed for each module. The team concluded that the FRAME-IS is practical, comprehensive, and easy to use. It works well to document modifications across levels of influence (i.e.- system-wide, clinic-wide, etc.) and promotes reflection to raise critical questions regarding implementation. Challenges include determining what defines a distinct “modification,” the blurring of roles (i.e.- researcher/implementer/manager), and how to differentiate modifications to ISs versus those to the study design or EBP. Recommendations to advance the FRAME-IS are provided.

**Implications for D&I Research:** Considerations and recommendations from this case study can be used to enhance the FRAME-IS, assist other scholars to utilize this framework, and improve the research community’s ability to systematically measure the dynamic evolution of implementation strategies in various settings. Future research is needed to study how documented modifications influence implementation outcomes.

**Primary Funding Source**

National Institutes of Health

### S118 Leveraging economic tools in the context of the stages of implementation completion (sic) to inform implementation and plan for sustainment

#### Piper Block, Mark Campbell, Lisa Saldana

##### Oregon Social Learning Center, Eugene, OR, USA

###### **Correspondence:** Piper Block (piperb@oslc.org)

*Implementation Science 2023,* **18(Suppl 3):**S118

**Background**

Implementation science addresses the inherent tensions between research and practice by developing rigorous tools for processes that might otherwise happen haphazardly or inconsistently. For economic considerations, such tools are paramount for comprehensively accounting for financial and resource costs, funding streams, and issues of billing and financial sustainability. The Stages of Implementation Completion (SIC) is both a framework and measure for implementing evidence-based practices. Economic considerations are an important theme woven throughout the SIC. This presentation provides an overview of economic tools employed within the context of the SIC for the Families Actively Improving Relationships (FAIR) program, an evidence-based treatment for parents involved in the child welfare system due to substance use, to inform financial planning strategies and achievement of program sustainment.

**Methods**

During the Pre-Implementation Phase, the Costs of Implementing New Strategies (COINS), a cost mapping tool which maps onto the SIC, assisted in collecting and presenting precise information on the staffing and financial resources necessary to move into the Implementation Phase. The FAIR Cost Calculator created site-specific reimbursement profiles to share with site decision-makers to inform the optimal staffing ratios and caseload sizes to achieve financial balance. The Cost Calculator also elucidated unbillable costs to clinics which is critical for program sustainability. This information was presented to implementing decision-makers to better understand the necessary investment for successful implementation.

**Findings**

In the constantly changing landscape of COVID-19, employing a series of explicit economic tools allowed the FAIR developer team to respond swiftly and flexibly to changing clinic needs. COVID-19 especially exacerbated two financial issues: clinician turnover (averaging $25,000 per new clinician in training and lost revenue) and unbillable supply runs to clients (averaging $284 per client per month). COVID-19 also presented new funding opportunities to address those issues, which the FAIR team was able to capitalize on given the consistent use of such economic tools.

**Implications for D&I Research**

By actively attending to economic considerations through the implementation process, we can better plan for sustainment. Economic tools like those previewed here can be useful for clinic-level financial sustainability, emerging funding opportunities, increasing cost transparency for community partners, and eventual economic evaluations for research purposes.

**Primary Funding Source**

National Institutes of Health

### S119 “what we have here, is a failure to [replicate]”: ways to solve a replication crisis in implementation science

#### Matthew Chinman^1^, Joie Acosta^2^, Patricia Ebener^3^, Amy Shearer^4^

##### ^1^RAND Corporation, Pittsburgh, PA, USA; ^2^RAND Corporation, Arlington, VA, USA; ^3^RAND Corporation, Santa Monica, CA, USA; ^4^RAND, Santa Monica, CA, USA

###### **Correspondence:** Matthew Chinman (chinman@rand.org)

*Implementation Science 2023,* **18(Suppl 3):**S119

**Background:** Replication, key to the open science movement, is needed to strengthen the validity of findings in Implementation science (IS)—yet has been neglected in IS in favor of novel discovery, like in other fields. For example, reviews of implementation strategies vary so much across content domains, settings, and strategy use, it is challenging to draw conclusions about strategy replicability. The purpose of this presentation is to review what is known about replication of implementation trials and identify the gaps and offer recommendations to continue increasing the transparency, openness, and replicability of implementation research.

**Methods:** This presentation will review how study replication has (or has not) been approached in IS. We will discuss how different types of replications (e.g., direct, conceptual) can benefit the IS field. We will then describe our Implementation Replication Framework (IRF)—developed incorporating elements from Proctor (strategy description), Damschroder (i.e., CFIR), and Fixsen (implementation core components) to guide implementation researchers in their replication efforts. Using the IRF, we will present a case study of how to design a replication study and interpret the results using the implementation strategy called Getting To Outcomes© (GTO), which was used to facilitate two different youth-focused prevention evidence-based practices (EBPs) in two different studies.

**Findings:** This presentation will argue that replication should not be binary—replicated, or not—but fall on a continuum, leading to a progressive research program in which non-replicated findings can yield new theories sufficiently broad to include both replicated and non-replicated findings. Using the IRF, we will also share multiple elements to consider when designing and interpreting replication studies (e.g., Participants, Setting, Intervention, Outcome measures, and Analyses) and explanations (e.g., varying levels of EBP intensity) for why implementation findings were replicated in the GTO case study, but youth outcomes were not.

**Implications for D&I Research:** The presentation will end with multiple recommendations implementation scientists could consider to improve the likelihood and quality of replication studies, including how to improve IS replication reporting and how IS can enable researchers and practitioners to work together in real-world contexts to encourage wide replication of implementation studies and advance both practice and theory in public health.

### S120 Identifying barriers to implementing a care coordination intervention in the veterans health administration: the brainwriting premortem focus group approach

#### Roman Ayele^1^, Marina McCreight^3^, Marcie Lee^4^, Gretchen Stage^5^, Lauren Mckown^5^, Brianne Morgan^5^, Deisy Hernandez Lujan^3^, Heidi Sjoberg^6^, Heather Gilmartin^2^, Catherine Battaglia^2^

##### ^1^VA Eastern Colorado Healthcare System, Aurora, CO, USA; ^2^Denver/Seattle Center of Innovation, Rocky Mountain Regional VA Medical Center; ^3^Eastern Colorado Healthcare System, Aurora, CO, USA; ^4^Department of Veteran Affairs, Denver-Seattle Center of Innovation Aurora, CO, USA; ^5^Denver-Seattle Center of Innovation, Department of Veterans Affairs, Eastern Colorado Health Care System, Denver, CO, Aurora, CO, USA; ^6^Veterans Health Administration, Denver Seattle Center of Innovation for Veteran-Centered and Value Driven Care Aurora, CO, USA

###### **Correspondence:** Roman Ayele (roman.ayele@va.gov)

*Implementation Science 2023,* **18(Suppl 3):**S120

**Background**

Engaging clinical and administrative partners early in the implementation process is necessary to improve uptake of healthcare interventions. One of the most helpful elements of engaging partners is to rapidly identify barriers during pre-implementation period. We sought to understand barriers to implementing a care coordination intervention aimed at improving transitions of care outcomes using brainwriting premortem sessions.

**Methods**

We conducted brainwriting premortem exercises, a novel focus group method, with participants from six Veterans Administration Medical Centers (VAMCs) implementing an evidence-based care coordination intervention. The brainwriting premortem method is the silent sharing of written ideas about why an intervention failed, prior to implementation of the program. Participants are asked to imagine the program was implemented and failed. They then write about the reasons the program failed. Using IdeaBoardz online platform, participants anonymously and silently typed their responses. The group was given time at the end to reflect and build off of each other’s responses. The written data were collected and exported for thematic analysis and returned to stakeholders for further discussion.

**Findings**

Participants indicated the program could fail due to multiple perceived barriers: (1) Lack of buy-in from staff; (2) Lack of collaboration between stakeholders; (3) Inadequate time allocated for the Lead Coordinator; (4) Competing priorities that would make this initiative unsustainable; (5) Perceived challenges engaging Veterans; (6) Case management Issues; (7) Poor rollout of the initiative in educating stakeholders and monitoring; (8) Overall staffing challenges such as turnover and protected time to implement the initiative; and (9) Inadequate support and resources. Participants were given opportunities to discuss strategies to address these barriers during program implementation.

**Implications for D&I Research**

The brainwriting premortem exercise allowed us to capture insights from stakeholders that could inform efficient implementation. This is a novel approach that can be applied in various settings to quickly understand barriers to program implementation.

**Primary Funding Source**

VA Health Services Research and Development

### S121 Use of concept mapping across stakeholder groups to prioritize importance and feasibility of evidence-based strategies for hpv vaccination within safety-net settings

#### Jennifer Tsui^1^, Michelle Shin^1^, Kylie Sloan^1^, Tom Mackie^2^, Benjamin Crabtree^3^, Lawrence Palinkas^1^

##### ^1^University of Southern California, Los Angeles, CA, USA; ^2^SUNY Downstate Health Science University, Brooklyn, NY, USA; ^3^Rutgers Robert Wood Johnson Medical School, New Brunswick, NJ, USA

###### **Correspondence:** Jennifer Tsui (Tsuijenn@usc.edu)

*Implementation Science 2023,* **18(Suppl 3):**S121

**Background:** HPV vaccination rates remain below target levels among adolescents in high-risk communities served by safety-net clinics. While multiple evidence-based strategies (EBS) for promoting HPV vaccination have emerged, identification and prioritization of EBS within safety-net settings to align with context and fit are understudied. Use of concept mapping to assess diverse stakeholders’ views and priorities of EBS can inform selection of strategies within the local context.

**Methods:** We conducted a concept mapping activity in Los Angeles and New Jersey with 20 participants, including: (1) internal clinical leaders/administrators, staff [MA, RN] and providers [MD, NP] and (2) external advocacy and policy representatives; both groups were previously interviewed (guided by Practice Change Model) about their experiences with EBS for HPV vaccination. Thirty-eight EBS statements, derived from qualitative data and HPV vaccine advocacy sources, were presented through the GroupWisdom Concept Mapping program. Participants sorted statements into clusters and rated each statement by importance and feasibility for increasing HPV vaccination in safety-net clinics. We compared common and divergent ratings across internal and external stakeholders.

**Findings:** Eight clusters emerged: provider recommendation/communication, reducing missed opportunities, nurse/staff workflow and training, improving patient opportunities/access for vaccination, data and QI monitoring, community education and outreach, community/cultural engagement, and advocacy/policy. Provider recommendation/communication, nurse/staff workflow and training, and improving opportunities/access were rated as the most important clusters. Both internal and external stakeholders rated provider recommendation/communication and reducing missed opportunities highest for feasibility. Internal stakeholders, however, rated feasibility of nurse/staff workflow and training below other clusters. Two EBS, both from the provider recommendation/communication cluster, emerged as the most important and feasible (go-zone): providers giving parents/adolescents time to ask about HPV vaccine during visits and providers focusing on HPV vaccine as cancer prevention and not on sexual transmission.

**Implications for D&I Research:** We identified consistency between internal and external groups in high prioritization of provider- and clinic-team focused strategies and increasing vaccine access. Our findings suggest concept mapping can respond to the complexities of implementation at the inner and outer context by assisting in the prioritization and selection of EBS across stakeholders and identifying how context influences strategy prioritization.

**Primary Funding Source**

National Institutes of Health

### S122 Multifaceted implementation strategy in the NIDA clinical trials network CTN-0097 trial

#### Miranda Greiner^1^, Matisyahu Shulman^1^, Onumara Opara^1^, Kenzie Potter^1^, Kathryn Hefner^2^, Lauren Dresser^2^, Christina Scheele^2^, Rachel Ho^2^, Eve Jelstrom^3^, Udi Ghitza^4^, Edward Nunes^1^, Adam Bisaga^1^

##### ^1^New York State Psychiatric Institute and Columbia University Irving Medical Center, New York, NY, USA; ^2^Emmes Company, LLC, Rockville, MD, USA; ^3^Emmes Company, LLC, Rockville, NY, USA; ^4^National Institute on Drug Abuse, Bethesda, MD, USA

###### **Correspondence:** Miranda Greiner (miranda.greiner@nyspi.columbia.edu)

*Implementation Science 2023,* **18(Suppl 3):**S122

**Background**

Implementation research studies are limited for evidence-based opioid use disorder (OUD) interventions and most studies lack sufficient details around implementation strategies limiting scientific or real-world replication. There is an urgent need to better understand how best to implement treatment with medications for opioid use disorder (MOUD) in the community amid the ongoing opioid crisis. Despite existing effective MOUD options, most patients admitted to inpatient detoxification units are discharged without MOUD. The NIDA Clinical Trials Network CTN-0097 trial provides an opportunity to examine implementation of a rapid intervention for initiating extended-release injectable naltrexone (XR-NTX) among OUD patients admitted to inpatient detoxification programs.

**Methods:** CTN-0097 is a hybrid type I effectiveness-implementation stepped-wedge randomized trial that compares the effectiveness of the standard (10-14 days) to the rapid procedure (5-7 days) for XR-NTX initiation across six community inpatient detoxification programs while developing an implementation package. A multifaceted implementation strategy is used in CTN-0097 that includes: 1) site needs assessment, 2) forming a local implementation team, 3) development of a formal implementation blueprint, 4) training and education, and 5) audit and feedback that includes coaching by expert addiction-trained clinicians. In each step, implementation strategies are evaluated and adapted in response to site-level barriers, facilitators, or staff feedback. Adaptations (and rationale for changes) to implementation strategies are captured using the Framework for documenting Modifications to Implementation Strategies (FRAME-IS). Qualitative data collection and analysis on barrier and facilitators are guided by the Consolidated Framework for Implementation Research (CFIR).

**Findings:** Preliminary findings from qualitative interviews with site-level stakeholders suggest that identification and education of a local clinical champion who trains local staff in a train-the-trainer model improved adoption and fidelity to the rapid intervention for XR-NTX. Site-level clinical champions found audit and feedback or “coaching” sessions while actively implementing the rapid intervention to be more helpful than the preliminary didactics or web-based materials.

**Implications for D&I Research**

To our knowledge, this is the first time FRAME-IS has been used in a multisite national trial. Implementation findings from this trial may provide a blueprint of implementation strategies that can help shift practices and improve MOUD initiation rates (apart from XR-NTX) across community inpatient detoxification programs.

**Primary Funding Source**

National Institutes of Health

### S123 The rapid implementation feedback (rif) report: real-time synthesis of qualitative data for proactive implementation planning and rollout

#### Erin Finley^1^, Joya Chrystal^2^, Alicia Gable^3,4^, Anneka Oishi^4^, Karen Dyer^4^, Rebecca Oberman^5^, Rachel Lesser^6^, Ismelda Canelo^2^, La Shawnta Jackson^2,4^, Tanya Olmos-Ochoa^5^, Tannaz Moin^4^, Bevanne Bean-Mayberry^4^, Melissa Farmer^2,4^, Alison Hamilton^2^

##### ^1^UT Health San Antonio, San Antonio, TX, USA; ^2^Center for the Study of Healthcare Innovation, Implementation, & Policy (CSHIIP),Los Angeles, USA; ^3^Center for Healthcare Innovation, Implementation and Policy, Los Angeles, CA, USA; ^4^Greater Los Angeles Healthcare System, Los Angeles, CA, USA; ^5^Greater Los Angeles VA, MIRECC, Los Angeles, CA, USA; ^6^Veterans Health Administration, Los Angeles, CA, USA

###### **Correspondence:** Erin Finley (finleye@uthscsa.edu)

*Implementation Science 2023,* **18(Suppl 3):**S123

**Background:** The VA EMPOWER 2.0 QUERI is conducting a hybrid type 3 effectiveness-implementation trial comparing the impact of Replicating Effective Programs and Evidence-Based Quality Improvement as strategies for implementing three evidence-based practices (EBPs) for women Veterans: Virtual Diabetes Prevention Program; Telephone Lifestyle Coaching to reduce cardiovascular risk; and Reach Out, Stay Strong Essentials to prevent postpartum depression. We describe an innovative, pragmatic, team-based approach for the rapid synthesis of qualitative data to aid implementation planning, tailoring, and rollout across strategies, interventions, and sites.

**Methods:** Trained qualitative staff conducted pre-implementation interviews with site- and regional-level partners to assess content domains reflecting the Consolidated Framework for Implementation Research and behavior design. EMPOWER 2.0’s implementation and qualitative teams met to agree upon high-priority domains related to implementation planning (e.g., critical roles; concerns related to EBPs). Following each interview, the qualitative team reviewed interview notes and summarized key points for each domain, producing a structured Rapid Implementation Feedback (RIF) report organized by site, region, and EBP. Information was added cumulatively to the RIF, with emergent findings highlighted in weekly emails and meetings with the implementation teams.

**Findings:** 82 semi-structured interviews were completed with frontline staff, providers, and leadership across 11 sites in three regions, November 2021-June 2022. The qualitative team’s weekly updates on the RIF supported continuous communication about key findings, particularly questions and concerns raised by participants related to the three EBPs and their expected impact for sites. The implementation teams drew upon findings in real time to refine and tailor implementation planning, including by: developing a frequently asked questions (FAQ) document to support clear communication; tailoring site rollout activities to address local needs, resources, and concerns; and informing design and tailoring of a quality dashboard for sites. Findings have been used to tailor implementation planning and rollout at six sites to date.

**Implications for D&I Research:** Rapid qualitative methods are a critical tool for enhancing implementation planning, communication, and tailoring. The RIF report provides a structured strategy for distillation of early findings, allowing continuous communication between qualitative and implementation teams, and supporting effective tailoring of implementation rollout in real time.

**Primary Funding Source**

Department of Veterans Affairs

### S124 Consensus-based mixed methods for identifying and describing implementation strategies across multiple research studies in the accelerating colorectal cancer screening through implementation science (accsis) initiative

#### Prajakta Adsul^1^, Nidhi Kanabar^2^, Melinda Davis^3^, Borsika Rabin^4^

##### ^1^University of New Mexico, Albuquerque, NM, USA; ^2^University of New Mexico Comprehensive Cancer Center, Albuquerque, USA; ^3^Oregon Health & Science University, Portland, OR, USA; ^4^Veterans Health Administration, La Jolla, CA, USA

###### **Correspondence:** Prajakta Adsul (padsul@salud.unm.edu)

*Implementation Science 2023,* **18(Suppl 3):**S124

**Background**

Multi-level contextual factors influence implementation of interventions that can be addressed by implementation strategies. Methods to identify and describe these strategies are essential to advance the science of implementation. The Accelerating Colorectal Cancer Screening through Implementation Science (ACCSIS) Cancer Moonshot initiative provides a unique opportunity to study implementation strategies used in diverse primary care settings and examine the similarities and differences within the selection and operationalization of these strategies across participating research studies. We describe the consensus-based, mixed methods used to identify and describe implementation strategies used across the ACCSIS initiative.

**Methods**

Seven of the eight research studies completed an Excel-based data collection form that was developed using Proctor’s guidance for specifying implementation strategies. We approached data collection using the overarching question - “What activities were undertaken in support of implementation of colorectal cancer screening in clinical settings?” Study team members provided lists of activities that were reviewed by three independent experts who then matched each described activity to strategies per the Experts Recommending Implementation Change (ERIC) taxonomy. Disagreements were resolved through discussions to reach consensus and final validation was sought from each participating site.

**Findings**

Each study reflected activities used in primary care settings representing diverse geographic regions and patient- and provider-level characteristics. Sites initially reported between 3-17 activities, which when matched on to the ERIC taxonomy resulted in 77 primary strategies. Additional strategies that overlapped with the primary strategy were considered as secondary strategies (approximately 1-2 per primary strategy). The identification and matching of strategies required extensive review, a thorough knowledge of the operationalization of the strategies, and used a consensus approach between three experts. We noted the lack of direct alignment between ERIC strategies and activities used in practice, identified several opportunities for improved operationalization of the target and actions associated with each strategy, and identified common combinations of strategies as used in practice.

**Implications for D&I Research**

Classifying and matching strategies in an iterative and systematic process that involved expert reviewers and input from participating ACCSIS research teams, ensured rigor and validity to the study data, showcasing a low respondent-burden methodology that could be applicable to diverse contexts.

**Primary Funding Source**

National Institutes of Health

### S125 Lessons learned and future directions for strategy tracking in research consortia using the longitudinal implementation strategy tracking system (lists) method

#### Justin Smith^1^, Wynne Norton^2^, Lisa DiMartino^3^, Sandra A Mitchell^4^, Michael Hassett^5^, Christine Cronin^5^, Jennifer Ridgeway^6^, James Merle^7^, Jennifer Bannon^8^, Theresa Walunas^9^

##### ^1^Room 1N410, University of Utah School of Medicine, Salt Lake City, UT, USA; ^2^National Cancer Institute, Bethesda, MD, USA; ^3^RTI International, Research Triangle Park, NC, USA; ^4^Natinal Cancer Institute, National Institutes of Health, Bethesda, USA; ^5^Dana-Farber Cancer Institute, Boston, MA, USA; ^6^Mayo Clinic, Rochester, MN, USA; ^7^University of Utah, Salt Lake City, USA; ^8^Northwestern University, Chicago, IL, USA; ^9^Northwestern University, Feinberg School of Medicine, Chicago, IL, USA

###### **Correspondence:** Justin Smith (jd.smith@hsc.utah.edu)

*Implementation Science 2023,* **18(Suppl 3):**S125

**Background**

Systematic approaches are needed to describe, measure, and track implementation strategies across multiple studies in research consortia. We describe a novel methodology for characterizing implementation strategies over time, discuss its use across several research consortia, identify challenges and benefits of the methodology, and describe how and when it might be adapted for use in future research consortia.

**Methods**

We used the Longitudinal Implementation Strategy Tracking System (LISTS) methodology to characterize strategies across multi-site, multi-study research consortia over time. LISTS includes a set of procedures, a strategy assessment, and a data capture tool to collect common strategy data elements, including specificity, timing, and reason for adding, modifying, or discontinuing any of the 73 discrete implementation strategies included in the Expert Recommendations for Implementation Change (ERIC) compilation. LISTS was first developed and is currently in use in three hybrid effectiveness-implementation trials testing symptom management interventions in ambulatory oncology care as part of the NCI Cancer Moonshot^SM^ Research Consortium*, I*mproving the *M*anagement of Sym*P*toms during *A*nd following *C*ancer *T*reatment (IMPACT). LISTS is also being used in two ambulatory primary care studies that are part of the AHRQ EvidenceNOW program.

**Findings**

Preliminary use of LISTS indicates it is feasible, acceptable, and provides an accurate methodology for characterizing implementation strategies across studies within research consortia over time. Benefits of using LISTS include a systematic approach for prospective data collection and common data elements that foster opportunities for cross-study analyses of detailed contextual information. Challenges include time required for strategy entry and updates, technical infrastructure to manage the data capture tool, and validation of data entry. Suggestions for adaptation of LISTS include variations of procedures, level of detail of strategy assessment (e.g., 73 discrete strategies vs. 9 categories of strategies), and options for data capture software (e.g., REDCap, R Shiny).

**Implications for D&I Research**

LISTS represents an advancement in characterizing implementation strategies over time. This system facilitates collection of common data elements and synthesis across multi-site, multi-study research consortia that span diverse content areas and delivery settings. Future use of LISTS in additional research consortia will inform refinement and adaptation to increase validity and reduce reporting burden.

**Primary Funding Source**

National Institutes of Health

### S126 A centralized implementation core for tracking implementation strategies across a VA-wide queri program

#### Rani Elwy^1,^, Bo Kim^2^, Keith McInnes^2^ ,Thomas Byrne^2^, Megan McCullough^2^, David Smelson^2^, Beth Ann Petrakis^2^, Elizabeth Maguire^3^, Angela Kyrish^3^, Justeen Hyde^3^, Samantha Sliwinski^4^, Sarah Javier^5^, Amanda Midboe^5^, Vera Yakovchenko^6^

##### ^1^Veterans Health Administration, Providence, RI, USA; ^2^Center for Healthcare Organization and Implementation Research (CHOIR), Boston, MA, USA; ^3^VA Bedford Healthcare System, Bedford, MA, USA; ^4^Boston Healthcare System, Boston, MA, USA; ^5^Center for Innovation to Implementation, Menlo Park, CA, USA; ^6^BridgeQUERI, CHOIR Pittsburgh, PA, USA

###### **Correspondence:** Rani Elwy (rani_elwy@brown.edu)

*Implementation Science 2023,* **18(Suppl 3):**S126

**Background**

The Bridging the Care Continuum for Vulnerable Veterans Quality Enhancement Research Initiative (BridgeQUERI) program tests three evidence-based practices that target Veterans with mental health and substance use treatment needs, delivered by VA staff and peer support specialists. We describe BridgeQUERI Implementation Core centralized efforts to track the use, adaptation and effectiveness of low- and high-intensity implementation strategies in these three Hybrid Type III trials across 20 sites, guided by the Dynamic Sustainability Framework (DSF) and the QUERI Implementation Roadmap.

**Methods**

Each trial staggers the introduction of implementation strategies at each site, moving between low-intensity strategies (educational outreach, academic detailing) and high-intensity strategies (implementation facilitation) over 12 months. Trial teams conducted formative evaluations comprising implementation measures and qualitative interviews, documenting contextual factors related to implementation readiness. Fidelity-consistent adaptations to implementation strategies, based on these data, were created. Prior to moving between low- and high-intensity strategies, repeated implementation measurement and process evaluation interviews identified continued challenges and needed strategy adaptations. Trial teams document pre-implementation, implementation and sustainment activities, including strategy adaptations, through the Stages of Implementation Completion (SIC) Checklist combined with the QUERI Facilitation Tracker. De-identified REDCap survey data, qualitative data, SIC and facilitation information are centrally available in our BridgeQUERI implementation dashboard, organized by DSF and Roadmap domains.

**Findings**

The deployment of different implementation strategies to increase uptake of evidence-based practices at many sites requires the use of consistent methods and a combination of tools to identify, track and determine the effectiveness of these strategies. Multiple adaptations to pre-determined implementation strategies were needed, based on differences in readiness, acceptability, appropriateness, feasibility, and availability of implementers at project sites. BridgeQUERI Implementation Core created new tools to track implementation strategy use, to ensure each trial was adhering to low-intensity and high-intensity strategy definitions. Frequent meetings with trial teams were necessary, outside of regular Implementation Core meetings, to share insights across teams and ensure accurate classifying and tracking of strategies.

**Implications for D&I Research**

Tracking the use, adaptation and effectiveness of strategies is necessary, to move evidence-based practices into routine care settings. This requires a central unit, such as an Implementation Core, to provide oversight, guidance and coordination among projects.

**Primary Funding Source**

Department of Veterans Affairs

### S127 Using agent-based modeling to understand and address mis-implementation in u.s. state health departments

#### Matt Kasman^1^, Ross Hammond^2^, Rob Purcell^3^, Louise Farah Saliba^4^, Stephanie Mazzucca^5^, Margaret Padek^5^, Peg Allen^4^, Douglas Luke^6^, Sarah Moreland-russell^5^, Paul Erwin^7^, Ross Brownson^4^

##### ^1^Brookings Institution, Washington, DC, USA; ^2^Washington University in St Louis, St Louis, MO, USA; ^3^Independent Consultant,Washington, DC, USA; ^4^Brown School, Prevention Research Center, Washington University in St. Louis, St. Louis, MO, USA; ^5^Prevention Research Center, Washington University in St. Louis, St. Louis, MO, USA; ^6^Center for Public Health Systems Science, Washington University in St. Louis, Saint Louis, MO, USA; ^7^University of Alabama at Birmingham, Birmingham, AL, USA

###### **Correspondence:** Matt Kasman (mkasman@brookings.edu)

*Implementation Science 2023,* **18(Suppl 3):**S127

**Background**

Our research goal was to explore why mis-implementation—ending effective activities prematurely or continuing ineffective ones—sometimes occurs in public health and how it can be reduced. Mis-implementation contributes to wasted resources and sub-optimal health outcomes. Early termination of effective policies, environmental changes, and behavioral interventions perpetuates suboptimal health outcomes, including continued early onset or inadequate management of diabetes and other chronic conditions. Continuation of interventions that do not reaching priority population groups as intended can exacerbate health disparities.

**Methods**

We created an agent-based model (ABM) that represents how information flow, filtered through organizational structure, capacity, culture, and leadership priorities shapes continuation decisions in public health departments. This ABM was co-designed with stakeholders, and parameterized and tested using survey responses and interviews with state health department personnel across the U.S. between 2014 and 2020. After determining that the model had sufficient explanatory power to reproduce observed levels of mis-implementation, we used it experimentally to identify potential approaches for reducing mis-implementation.

**Findings**

Analyses of data from simulations indicate that increasing public health department employees’ evidence-based decision-making capacity or willingness to share information with other employees and responsiveness to information that is shared can reduce mis-implementation. Shifting leadership priorities away from considerations other than evidence supporting intervention effectiveness results in the largest reduction. Organizational restructuring, such as reducing the number of hierarchical layers in the organization (i.e., “flattening”) does not reduce mis-implementation.

**Implications for D&I Research**

We identified factors and dynamic pathways most likely driving mis-implementation, and suggest actionable strategies for reducing mis-implementation. Priorities for training the public health workforce include evidence-based decision-making and effective intra-organizational communication. Organizations will also benefit from an intentional shift in leadership decision-making processes. Notably, effectively removing intervention age from leadership’s continuation decisions had a large positive impact. Efforts to effect such a change might include intentional changes in organizational policies and practices that support viewing activities with fresh eyes, circumventing organizational inertia, and avoiding sunk cost mentality in favor of prioritizing effective interventions. Our presentation will be supported with an animation, which we intend as an example of effectively disseminating findings from a highly technical model to a wide audience of stakeholders.

**Primary Funding Source**

National Institutes of Health

### S128 Human-centered design methods to enhance intervention and implementation strategy usability: cross-project outcomes from the uw alacrity center

#### Aaron Lyon^1^, Sean Munson^1^, Michael Pullmann^2^, Emily Friedman^1^, Katie Osterhage^1^, Ryan Allred^1^, Brittany Mosser^3^, Alejandra Lopez^1^, John Fortney^4^, Ian Bennett^1^, Patrick Raue^1^, James Fogarty^1^, Patricia Arean^3^, UW ALACRITY Center Researchers^1^

##### ^1^University of Washington, Seattle, WA, USA; ^2^Department of Psychiatry and Behavioral Sciences Division of Public Behavioral h, University of Washington, Seattle, WA, USA; ^3^University of Washington, Seattle, USA; ^4^VA Puget Sound Health Care System, Seattle, WA, USA

###### **Correspondence:** Aaron Lyon (lyona@uw.edu)

*Implementation Science 2023,* **18(Suppl 3):**S128

**Background:** Implementation strategies and patient-facing interventions both tend to be complex psychosocial processes, the usability of which can be a major barrier to their adoption. Despite the emergence of a “science of adaptation,” few methods exist to inform systematic redesign to ensure usable psychosocial innovations. The NIMH-funded University of Washington ALACRITY Center (UWAC) applies human-centered design (HCD) methods to enhance the usability of implementation strategies and interventions for mental health in community settings. UWAC leverages the three-phase Discover, Design/Build, Test (DDBT) process to drive iterative innovation redesign. Using data from 13 separate UWAC projects, this presentation will describe the DDBT methods applied and report on resulting cross-project usability issues and redesign solutions.

**Methods:** All 13 projects employed mixed-methods user testing methods such as cognitive walk-throughs, behavioral rehearsals, asynchronous remote communities, and interviews. Project teams reported usability issues using UWAC’s common reporting framework, which Center researchers categorized through three rounds of consensus coding. Teams articulated redesign solutions to address high-priority usability issues via the Framework for Adaptations and Modifications to Evidence-based interventions/Implementation Strategies (FRAME/-IS). Teams also collected common quantitative instruments to measure usability and implementation outcomes and guide design decisions across DDBT phases. Using projects that collected data across multiple DDBT phases, we computed standardized effect sizes for phase-to-phase change.

**Findings:** Projects reported a total of 90 usability issues, coded into 12 categories (e.g., Complex or cognitively overwhelming; Over reliance on technology). Issues ranged from minor inconveniences to severe issues that undermined delivery of the intervention or strategy. The 12 categories were linked to 15 types of redesign solutions. Redesign most often included the FRAME/-IS category of tailoring or refining intervention/strategy content. Effect sizes across DDBT phases were heterogenous among projects (*d* = -.65 to 1.56). The most successful redesigns modified delivery *parameters*—such as content sequencing or modality changes to fit time constraints—and visual/digital *artifacts.*

**Implications for D&I Research:** Integration of data across 13 UWAC projects allowed for identification of common design problems and redesign solutions which can inform future initiatives aimed at ensuring the usability of complex psychosocial interventions. HCD methods have potential to improve intervention/strategy usability and advance the science of adaptation.

**Primary Funding Source**

National Institutes of Health

### S129 Is implementation support worth it? Costs and impacts on sustainability for substance abuse prevention programs

#### Joie Acosta^1^, Matthew Chinman^2^

##### ^1^RAND Corporation, Arlington, VA, USA; ^2^Center for Health Equity Research and Promotion, Pittsburgh, PA, USA

###### **Correspondence:** Joie Acosta (jacosta@rand.org)

*Implementation Science 2023,* **18(Suppl 3):**S129

**Background**

Problematic rates of substance abuse among US adolescents highlight the need for effective evidence-base programs (EBP). Yet schools and community organizations have a consistently low rate of EBP adoption and poor fidelity. While research has shown that implementation support can improve substance abuse prevention program implementation and outcomes in these low resource settings, little is known about whether implementation support is cost effective and results in sustained improvements. This presentation will summarize findings from two sets of analyses from a randomized controlled trial of CHOICE, an after-school substance abuse prevention EBP for adolescents, in 29 Boys and Girls Clubs (BGCs) across Southern California with and without an implementation support system called Getting To Outcomes© (GTO).

**Methods**

The analyses focused on cost effectiveness of the GTO implementation support over a two-year period (Analysis 1) and its impact on CHOICE sustainability (Analysis 2), two years after GTO ended. Analysis 1 used micro-costing methods to estimate the CHOICE and GTO costs. GTO support, labor and expense data were obtained from logs kept by the BGC and GTO technical assistance (TA) staff. GTO and BGC staff time were valued based on Bureau of Labor Statistics estimates. In Analysis 2, predictors of sustainability were identified for key GTO tasks (e.g., goal setting, evaluation, collectively called ‘GTO performance’) and for CHOICE fidelity using multiple path models.

**Findings**

In Analysis 1, the cost of implementing CHOICE at BGCs for two-years was $27 per attendee when CHOICE was offered by itself and $177 per attendee when CHOICE was offered with GTO implementation support. For this additional cost, analyses showed CHOICE was offered with more fidelity and offered more often after the 2-year intervention ended. Analysis 2 showed that two years after GTO support ended, GTO sites were significantly more likely to sustain CHOICE implementation when compared with control sites and better GTO performance predicted better CHOICE fidelity.

**Implications for D&I Research**

GTO could be a cost-effective option to support substance abuse prevention EBPs. Using an implementation support intervention like GTO can help low-resource settings continue to sustain their EBP implementation to help them get the most out of their investment.

**Primary Funding Source**

National Institutes of Health

### S130 The systems analysis and improvement approach (saia): specifying core components of an implementation strategy to optimize care cascades in public health

#### Sarah Gimbel^1^, Kristjana Ásbjörnsdóttir^1,2^, Kristin Banek^3^, Madeline Borges^1^, Joana Coutinho^4^, Jonny Crocker^1^, Vasco Cumbe^5^, Aneth Dinis^1^, McKenna Eastment^1^, Douglas Gaitho^6^, Carmen Hazim^1^, Barrot Lambdin^7^, R. Scott McClelland^1^, Ana Olga Mocumbi^8^, Alberto Muanido^9^, Ruth Nduati^10^, Irene Njuguna^11^, Onei Uetela^1^, Anjuli Wagner^1^, George Wanje^10^, Bradley Wagenaar^12^, Kenneth Sherr^13^

##### ^1^University of Washington, Seattle, WA, USA; ^2^University of Iceland, Reykjavik, Iceland; ^3^University of North Carolina, Chapel Hill, Chapel Hill, USA; ^4^Comité para Saúde de Moçambique, Beira, Mozambique; ^5^Ministério da Saúde, Direcção Provincial de Saúde de Sofala, Beira, Mozambique; ^6^Network of AIDS Researchers of East and Southern Africa, Nairobi, Kenya; ^7^RTI International, Berkeley, CA, USA; ^8^National Institute of Health, Maputo, Mozambique; ^9^Independent Consultant, Beira, Mozambique; ^10^University of Nairobi, Nairobi, Kenya; ^11^Kenyatta National Hospital, Nairobi, Kenya; ^12^Seattle, WA, USA; ^13^University of Washington, SEATTLE, WA, USA

###### **Correspondence:** Sarah Gimbel (sgimbel@uw.edu)

*Implementation Science 2023,* **18(Suppl 3):**S130

**Background:** Healthcare systems in low-resource settings need simple, low-cost interventions to improve services and address care gaps. Though routine data provide opportunities to guide these efforts, frontline healthcare workers (HCW) are rarely engaged in analyzing them for facility-level decision making. The Systems Analysis and Improvement Approach (SAIA) is an evidence-based, multi-component implementation strategy that engages HCW in use of facility-level data to promote systems-level thinking and quality improvement (QI) efforts within multi-step care cascades. SAIA was developed to address HIV care in resource-limited settings, but has since been adapted to a variety of clinical care systems including cervical cancer screening, mental health treatment, and hypertension management; and across a variety of settings in sub-Saharan Africa and the United States. We aimed to extend the growing body of SAIA research by defining the core elements of SAIA using established specification approaches, to improve reproducibility, guide future adaptations and lay groundwork to define its mechanism of action.

**Methods:** Over 12 months, a three-round, modified Delphi approach was employed by a panel of SAIA experts to name, define and operationalize SAIA using Proctor’s recommendation for specifying and reporting; then SAIA core components were matched to relevant Expert Recommendations for Implementing Change (ERIC) implementation strategies.

**Findings:** The core components of SAIA mapped to 13 ERIC strategies. SAIA strategy meetings encompassed external facilitation, organization of provider implementation meetings, and provision of ongoing consultation. Cascade analysis mapped to three ERIC strategies; facilitating relay of clinical data to providers, use of audit and feedback of routine data with healthcare teams, and modelling and simulation of change. Process mapping mapped to local needs assessment, local consensus discussions, assessment of readiness and identification of barriers and facilitators. Continuous quality improvement encompassed tailoring strategies, developing a formal implementation blueprint, cyclical tests of change and purposefully re-examining the implementation process.

**Implications for D&I Research:** SAIA is well-suited to quality improvement efforts in systems containing a defined care cascade and routinely available data, especially when modifications to workflows are within HCW control. Specifying the components of SAIA provides improved conceptual clarity to enhance reproducibility for other researchers and practitioners interested in applying the SAIA across novel settings.

**Primary Funding Source**

National Institutes of Health

## Prevention and Public Health

### S131 Patterns of program sustainability capacity among national dpp delivery organizations: a latent profile analysis

#### Lillian Madrigal^1^, Regine Haardörfer^2^, Michelle Kegler^1^, Linelle Blais^1^, Mary Beth Weber^1^

##### ^1^Emory University, Atlanta, GA, USA; ^2^Rollins School of Public Health, Emory University, Atlanta, GA, USA

###### **Correspondence:** Lillian Madrigal (lmadrig@emory.edu)

*Implementation Science 2023,* **18(Suppl 3):**S131

**Background:** The National Diabetes Prevention Program (DPP), a lifestyle intervention to delay the onset of diabetes, has been rigorously tested, adapted, and scaled nation-wide. Since 2012, there have been over 3000 organizations who have delivered the program, however in 2022 only around 2000 organizations are registered with the CDC, indicating challenges with organizational sustainability. Understanding patterns in sustainability capacity across program implementers may be useful in supporting the implementation of the National DPP. We explored patterns of sustainability capacity among current implementers using the Program Sustainability Assessment Tool (PSAT) in order to understand sustainability strengths and weaknesses and associated organizational characteristics. The PSAT explores 8 domains: environmental support, funding stability, partnerships, organizational capacity, program evaluation, program adaptation, communications, strategic planning.

**Methods:** This study analyzed organization characteristics and PSAT data from a 2021 cross-sectional online survey with 586 National DPP staff (lifestyle coaches, master trainers, program coordinators). Latent profile analysis (LPA) was employed to explore patterns of sustainability capacity. To estimate associations between derived latent classes and organization characteristics multivariable multinomial logistic regression was conducted. Multivariable linear regression with the PSAT score as the outcome was used to compare against the LPA model results. Our analysis included 440 program implementers with a calculable PSAT score.

**Findings:** The final LPA model included four classes: “low program sustainability,” 8% of the sample; “medium-low program sustainability,” 22%; “medium-high program sustainability,” 41.6%; and “high program sustainability,” 28.4%. All organizations, despite capacity level, tended to have the same areas of strength (Program Evaluation and Adaptation) and relative weakness (Funding Stability and Partnerships). Higher PSAT scores were associated with the number of staff, offering virtual delivery, grant funding sources, and specific organization types (e.g., government, academic).

**Implications for D&I Research:** There has been movement in recent years to better define, operationalize, and measure sustainability of public health evidence-based programs. The results of the LPA and regression models provide evidence to support the use of the PSAT score to identify organization sustainability capacity reliably. The National DPP and other prevention programs can benefit from using the PSAT to assess their sustainability capacity and as a first step toward sustainability planning.

### S132 Scaling up implementation support for violence prevention and resilience promotion in the air force

#### Matthew Chinman^1^, Amy Shearer^2^, Joie Acosta^3^

##### ^1^Center for Health Equity Research and Promotion, Pittsburgh, PA, USA; ^2^RAND, Santa Monica, CA, USA; ^3^RAND Corporation, Arlington, VA, USA

###### **Correspondence:** Matthew Chinman (chinman@rand.org)

*Implementation Science 2023,* **18(Suppl 3):**S132

**Background:** Closing the research-to-practice gap requires that organizations consistently incorporate best practices. The US Department of the Air Force (DAF) is attempting to increase adoption of evidence-based violence prevention and resilience promotion programs across the Department. To assist this effort, DAF is using Getting To Outcomes (GTO), an evidence-based implementation support that helps organizations plan, implement, and self-evaluate programs. Thus, DAF is scaling up prevention programs, and also embarking on the largest scale up of GTO ever.

**Methods:** Personnel from all Air Force installations (N=94) were trained to use GTO for their programs. GTO coaches helped installation personnel set goals, select and adapt evidence-based programs, and create Community Action Plans (CAPs) for implementation and evaluation (GTO Steps 1-6). Participating DAF personnel received a customized GTO guide and four supplemental sets of GTO tools addressing DAF-identified priority topics (suicide, sexual harassment, sleep health, stress management). The initiative was evaluated via GTO training questionnaires, a quality review of CAPs from individual installations (N=74) with the modified Plan Quality Index (PQI), and interviews with participants (N=27) representing installations ranging in CAP quality.

**Findings:** Training participants (N=300) were satisfied with almost all GTO training components. 63% of CAPs (n=47) were rated High quality on the PQI. 36% (n =27) were rated as Moderate quality because some GTO tools were missing, or the plan wasn’t logical, misstated benchmarks, or had limited implementation details. In interviews, installations reported GTO provided a useful, albeit lengthy, structure to complete their CAP; strengthened their prevention capacity; improved communication among prevention teammates; and improved CAP quality. Barriers to GTO use was lack of leader buy-in, access to data, and time.

**Implications for D&I Research:** This study suggests that GTO achieved this success via improved communication, a common and cross-cutting lexicon, and a more rigorous and standardized process for CAP development. This study builds on established implementation science frameworks for scaling up interventions by identifying critical tasks and unique supports needed to scale up evidence-based prevention. These findings suggest that establishing leadership buy-in, simplifying evidence-based program selection and adaptation, monitoring implementation and outcomes, and creating dedicated prevention practitioner roles are critical tasks to support scale-up of evidence-based prevention.

**Primary Funding Source**

Department of the Air Force

### S133 Virtual delivery of de-implementation strategies for inappropriate feeding practices in early care and education settings

#### Taren Swindle^1^, Virginia Mitchell^1^, Daphne Gaulden^1^, Janna Martin^1^, Susan Johnson^2^, Peyton Percle^3^, Megan Gremillion^4^, Julie Rutledge^3^

##### ^1^University of Arkansas for Medical Sciences, Little Rock, AR, USA; ^2^University of Colorado, Aurora, CO, USA; ^3^Louisiana Tech University, Ruston, LA, USA; ^4^Louisiana Tech, Ruston, LA, USA

###### **Correspondence:** Taren Swindle (tswindle@uams.edu)

*Implementation Science 2023,* **18(Suppl 3):**S133

**Background:** Inappropriate feeding practices occur frequently in early care settings and have negative effects on children’s eating behaviors and dietary intake. The study team and community partners co-designed a de-implementation strategy targeted at reducing inappropriate feeding practices while improving uptake of evidence-based ones. Necessitated by COVID-19, we co-adapted the de-implementation strategy package for virtual delivery. The strategy leveraged a peer learning collaborative, improvisation-based training, external facilitation, tailored educational resources, and audit and feedback. In a pilot mixed-method design, we examined feasibility, acceptability, appropriateness, and preliminary effectiveness of the virtual strategy package.

**Methods:** A pre-post design (24 classrooms) examined teachers’: (1) training evaluations, (2) perceptions of feasibility, acceptability, and appropriateness, and (3) changes in feeding practices via self-reported and observational measures. Ten teachers were purposively sampled for qualitative interviews. Baseline observations (March-May) and surveys (August) were collected in 2021 and follow-up data were collected in April-May of 2022.

**Findings:** Results indicated that the training was well-received and impactful (e.g., 97% agreed improv was a fun way to learn and helped them remember the concepts; qualitative comments indicated concrete memories of exercises). Indicators of feasibility, acceptability, and appropriateness of the intervention were stable from the post-training evaluation to the end-of-year survey and generally high. Qualitative data indicated there were challenges with platform engagement (e.g., time, login) which was supported by usage metrics. Changes in self-reported feeding practices were observed for verbal strategies that undermine autonomy (*t*(17)=4.37, *p*<.001) but not adult control, support behaviors, autonomy support, or social comparison behaviors. Changes in feeding practices (audio recordings) indicated significant decrease (56%) in inappropriate feeding practices (*t*(20)=3.10, *p*=.006) and significant increase (50%) in evidence-based feeding practices (*t*(20)=3.70, *p*=.001).

**Implications for D&I Research:** Some aspects of the virtual delivery of de-implementation strategies were memorable, well-received, and effective. However, teacher challenges with time to engage on the virtual platform mirrored facilitators’ struggles with relationship building in the virtual environment. Future work could evaluate a hybrid approach to offer both flexibility and to improve relationship building.

**Primary Funding Source**

National Institutes of Health

### S134 Designing for dissemination among clinical and public health practitioners in the united states

#### Thembekile Shato^1^, Russ Glasgow^2^, Maura Kepper^3^, Gabriella McLoughlin^4^, Rachel Tabak^5^, Ross Brownson^6^

##### ^1^Washington University in St. Louis, St. Louis, MO, USA; ^2^University of Colorado Anschutz Medical Campus, Aurora, CO, USA; ^3^National Cancer Institute’s Consortium for Cancer Implementation Science, USA; ^4^Washington University Implementation Science Center for Cancer Control (ISC3), St. Louis, MO, USA; ^5^Washington University in Saint Louis, Saint Louis, MO, USA; ^6^Washington University in St. Louis, Brown School, St. Louis, MO, USA

###### **Correspondence:** Thembekile Shato (shato@wustl.edu)

*Implementation Science 2023,* **18(Suppl 3):**S134

**Background:** Dissemination is critical for the effective adoption and implementation of evidence-based interventions (EBIs). The slow adoption of EBIs reflects gaps in effective dissemination of research evidence. Previous studies have examined Designing for Dissemination (D4D), a process that ensures that interventions and implementation strategies consider adopters’ needs, assets, and resources, primarily among researchers, with limited perspectives of practitioners. To address these gaps, this study examined the practice of D4D among practitioners in the United States.

**Methods:** We conducted a cross-sectional study among clinical and public health practitioners in spring 2022. Both groups were recruited through national-level rosters. The survey was informed by previous D4D studies and pre-tested using cognitive interviewing. We utilized descriptive analyses to examine the access and use of research evidence. Chi-square tests were used for sub-group analyses.

**Findings:** Among 578 respondents, 54.8% were clinical practitioners and 45.2% were public health practitioners. The most commonly ranked sources of research evidence were reading academic journals for clinical practitioners (37.9%) and email announcements for public health practitioners (43.7%). Compared to clinical practitioners, more public health practitioners used research evidence every time or almost every time to develop a new program/service (81.6% vs. 38.2%, p<0.001), evaluate existing programs/services (83.1% vs. 52.2%, p<0.001), and promote health equity (79.7% vs. 55.8%, p<0.001). Easy access to a brief summary of research evidence (30.3%) and easy access to original research evidence (35.7%) were the most important facilitators for using research by all practitioners. A significantly higher proportion of clinical practitioners compared to public health practitioners strongly agreed or agreed that within their work setting they had adequate financial resources (35.7% vs. 22.8%, p<0.001) and adequate staffing (35.7% vs. 24.0%, p=0.001) to implement research. Only 19.8% of all practitioners reported having a designated individual or team responsible for finding and disseminating research evidence.

**Implications for D&I Research:** D4D can be improved by addressing modifiable barriers, including organizational capacity to access and use research evidence and adopt EBIs. Our findings have implications for development and dissemination of strategies to better align the efforts of researchers with priorities and resources of practitioners—the adopters and implementers of EBIs.

**Primary Funding Source**

Brown School (Washington University in St. Louis) and Barnes Jewish Health Foundation

### S135 Application of consolidated framework for implementation research (cfir) mixed methods procedures to evaluate school wellness interventions – lessons learned from two ongoing studies

#### Gabriella McLoughlin

##### Washington University Implementation Science Center for Cancer Control (ISC3), St. Louis, MO, USA

###### **Correspondence:** Gabriella McLoughlin (gabriella.mcloughlin@temple.edu)

*Implementation Science 2023,* **18(Suppl 3):**S135

**Background:** Use of D&I research methods and frameworks enhances pragmatic decision making as school wellness interventions scale up over time. This presentation draws from two examples of ongoing programs: 1) The Game on Philly! intervention and 2) School Wellness Integration Targeting Child Health (SWITCH®) to highlight how the CFIR mixed methods protocols have been applied to school settings through a multi-step process.

**Methods:** For both studies (Game on Philly!: 6 schools, SWITCH: 52 schools), a multi-step method was adopted to adapt the CFIR constructs to school research. We decided on which constructs would be most relevant to schools based on literature and prior experience, adapted the CFIR interview guides to fit local contexts (i.e., K-12 settings, urban/rural), and developed a coding consensus document which facilitated inter-rater reliability among coders and quantitative scoring of CFIR constructs. Exploratory analyses (i.e., Pearson correlations, t-tests) and cross-case analyses were conducted to examine relations between determinants and outcomes.

**Findings:** Application of the CFIR procedures highlighted several key determinants of implementation that may not have been elucidated with traditional qualitative data collection measures. We discerned that leadership engagement (readiness) and knowledge and beliefs about intervention (individual characteristics) were the most positive determinants whereas available resources (readiness) and engaging parents (implementation process) were the most negative determinants. Challenges in applying CFIR methods to schools were shifting focus from “patients” to “students/parents”, changing how “provider” was used to highlight the school-level personnel involved in comprehensive programming, and trying to capture outer setting factors not common in clinical research while somewhat constrained by the available constructs.

**Implications for D&I Research:** CFIR methods facilitated decision-making to support implementation yet highlighted inherent challenges which must be addressed to advance the field of education D&I research. This research marks a key step in establishing shared measurement tools for use in education and community settings.

**Primary Funding Source**

United States Department of Agriculture; Office of Student Minority Affairs

### S136 Applying consolidated framework for implementation research (cfir) to policy implementation in school settings: reflections and examples from the national wellness policy study

#### Yuka Asada

##### Institute for Health Research and Policy, University of Illinois Chicago, Chicago, IL, USA

###### **Correspondence:** Yuka Asada (yasada2@uic.edu)

*Implementation Science 2023,* **18(Suppl 3):**S136

**Background:** The Healthy, Hunger-Free Kids Act of 2010 (P.L. 111-296) prompted the expansion of federal requirements in the U. S for the National School Lunch Program and local school wellness policies (hereafter, wellness policies), requiring that, effective 2017-2018, every school district receiving federal funds adopt a policy with provisions for physical activity and nutrition standards, amongst others. This presentation will discuss the application of the Consolidated Framework for Implementation Research (CFIR) to examine implementation of wellness policies, including the research team’s reflections on the adaptations and utility.

**Methods:** The National Wellness Policy Study was a mixed methods study that examined the impacts and implementation of the wellness policies; the qualitative component gathered data from multiple school professionals and student perspectives. This presentation draws from school superintendents (n=39) interview and focus group data, collected during the Association for School Superintendents (AASA) annual meeting in 2017. Data were team coded and organized in Atlas.ti software for hybrid analysis and generation of themes with the guidance of the CFIR.

**Findings:** CFIR offered a comprehensive framework to understand implementation in this complex setting; outer domain constructs, such as state ideologies, state policy, and external partnerships (adapted from ‘cosmopolitanism’) were the most salient. We paired the framework with education theory to conduct a more targeted examination of school superintendents and the influence of leadership on implementation. A missed opportunity was the framework’s lack of explicit focus on how inequities intersect with the implementation processes more broadly.

**Implications for D&I Research:** Adaptations to broaden the constructs in outer domain would better reflect the complexities of policy implementation in school settings embedded within local, state, and national settings. Explicit inclusion of equity and antiracism and how these constructs intersect with implementation processes are necessary for school settings.

**Primary Funding Source**

The Robert Wood Johnson Foundation

### S137 Building a “playbook” for future implementation: a qualitative secondary analysis of school meal program innovations during covid-19 across two states

#### Hannah Lane

##### Duke University School of Medicine, Department of Population Health Sciences, Durham, NC, USA

###### **Correspondence:** Hannah Lane (hannah.lane@duke.edu)

*Implementation Science 2023,* **18(Suppl 3):**S137

**Background:** Child nutrition insecurity is associated with myriad adverse health outcomes, particularly among children of color and in those rural areas. Federal school meals programs (SMPs) reduce nutrition insecurity during in-school months; however, stringent implementation mandates limit their reach during summer. During COVID-19 school closures, mandates were loosened to encourage innovations among local SMP implementers to improve reach. This Qualitative Secondary Analysis (QSA) investigates these innovations across two studies to recommend actionable implementation strategies to improve SMP reach in future summers.

**Methods:** QSA involves analyzing existing qualitative datasets with new research objectives. QSA reduces stakeholder burden, while increasing study output and policy/practice relevance. We re-analyzed 34 interviews conducted with SMP directors from April-September 2020 across two studies. We first aligned primary study instruments and codes through a data “crosswalk,” then developed a codebook to identify concepts present across both datasets. Two coders deductively coded transcripts with team de-briefs to identify actionable “best practices”, which were mapped to Expert Recommendations for Implementing Change (ERIC) strategies.

**Findings:** We assessed practices across five concepts: communication across stakeholders; partnerships; staffing; financial structures; partnerships. Practice themes aligned with 15 ERIC strategies. As examples, for communication, virtual platforms increased connectivity between local directors and state leaders (ERIC: Create learning collaborative), and more frequent conversations between directors and staff/families built confidence and mutual appreciation (ERIC: Organize implementation team meetings; Obtain family feedback). For partnerships, local partners eased supply-chain issues, filled operational gaps, and identified access points for hard-to-reach children (ERIC: Build a coalition; Develop resource sharing agreements).

**Implications for D&I Research:** QSA enabled re-investigation of operational innovations during COVID-19 without additional burden or costs to inform actionable guidance for future summer SMP implementation. Guidance is further strengthened through mapping to ERIC strategies. Implementation research should empirically test impacts of various implementation strategies on summer SMP reach to nutrition insecure children.

**Primary Funding Source**

National Institutes of Health

### S138 Improving fidelity of implementation of universal prevention initiatives in rural k-12 schools through external supports: testing mediational impacts on school team functioning, organizational readiness, and change commitment

#### Lindsey Turner

##### Boise State University, Boise, ID, USA

###### **Correspondence:** Lindsey Turner (lindseyturner1@boisestate.edu)

*Implementation Science 2023,* **18(Suppl 3):**S138

**Background:** Positive Behavioral Interventions and Supports (PBIS) is a multi-tiered framework for prevention and management of student behavior issues. Implementation uses a school-level team with 5-8 staff. An external support system may facilitate more-active school-level teams and, subsequently, improve fidelity of implementation.

**Methods:** We are conducting a Type 3 hybrid trial based on the Interactive Systems Framework, with an external support system providing technical assistance, coaching, and other implementation strategies, during phases of the Quality Implementation Framework (capacity building; creating structures; active implementation). In this parallel two-arm design, 20 schools were randomized to training-only control, and 20 were randomized to receive support during three school years (2019-20 to 2021-22). The primary outcome is fidelity, with a hypothesized mediational mechanism of action whereby supports improve school team functioning—measured with items from the Promoting School University Partnerships to Enhance Resilience (PROSPER) trial—and readiness, measured by the Organizational Readiness for Implementing Change (ORIC) scale. Analyses examined school team surveys, and the well-established PBIS Tiered Fidelity Inventory (TFI).

**Findings:** In 2020, team members at intervention schools reported higher scores for positive team culture, clear goals, and productive team meetings (*p*s<.05). Perceptions of organizational readiness and change commitment increased more at intervention schools relative to control (*p*s<.05). Multi-level structural equation models assessed relationships between condition, team survey constructs, and fidelity in 2020. The intervention had direct effects on team culture, which was significantly associated with subsequent fidelity (ps<.05), although the full indirect 2-1-2 path was marginally significant (p=.11). Similar results were noted for team perceptions of organizational readiness with significant direct condition effects on readiness, and readiness predicting TFI.

**Implications for D&I Research:** External support may improve team function and organizational readiness, subsequently benefitting fidelity. Discussion will explore how implementation strategies—provided through external support systems—may improve scaling of effective prevention initiatives in schools.

**Primary Funding Source**

National Institute of Justice

### S139 Consolidated framework for implementation research (cfir) factors predicting training engagement in a national dissemination of a faith-based online training for improving organizational practices related to physical activity and healthy eating

#### Sara Wilcox, Ruth P Saunders, Andrew T Kaczynski, Jessica Stucker, Kelsey R Day, Jasmin Parker-Brown

##### University of South Carolina, Columbia, SC, USA

###### **Correspondence:** Sara Wilcox (wilcoxs@mailbox.sc.edu)

*Implementation Science 2023,* **18(Suppl 3):**S139

**Background:** Faith-based organizations have significant potential for promoting population health, but few evidence-based programs are ready for dissemination. Faith, Activity, and Nutrition (FAN) is an evidence-based program to improve organizational practices related to physical activity (PA) and healthy eating (HE). This study reports CFIR factors predicting engagement with online training, a primary implementation strategy, in the first five cohorts of a national dissemination study.

**Methods:** US churches are recruited with assistance from faith-based and public health partners. FAN implementation strategies include 8 lessons delivered 1/week, a discussion board, and 12 months of resources. The coordinator from each church completes a pre-training survey that assesses baseline church practices for PA/HE along with items that map onto the CFIR. Committee members from each church register for the training and complete an online evaluation of each lesson and full training. Evaluation items, informed by two comprehensive models, have response options from 1 (strongly disagree) to 5 (strongly agree). Adoption is defined as the coordinator completing at least one lesson.

**Findings:** We have enrolled 57 churches (236 committee members) representing 14 states and 13 denominations; 72% with predominantly African American congregations. Of the 57 coordinators, 89% completed ≥1 lesson, and 79% completed all 8 lessons (69% and 52% across other committee members). Average lesson satisfaction, confidence to implement lesson strategies, and ease in navigating lessons were 4.4±0.6, 4.2±0.6, and 4.0±0.7, respectively. The full training was rated positively, with all items above 4.0 (e.g., interactive elements effective, would recommend the training, material can be taught virtually). Several CFIR items predicted greater lesson completion: belief that providing PA opportunities would benefit their churches (r=0.32, p=.02), greater age (r=0.30, p=.02), and report of ≥10 mins/week of PA (r=0.35, p<.01). Coordinators from the 51 adopting (vs 6 nonadopting) churches reported greater belief that providing PA opportunities would benefit their churches (p=.01) and that their pastors were more open to changes in church practices (p=.06).

**Implications for D&I Research:** We found high training engagement, favorable ratings, and several CFIR factors predicting engagement in this national dissemination study. The CFIR will be used to predict adoption, training engagement, and implementation in future analyses with the full sample.

**Primary Funding Source**

Centers for Disease Control and Prevention

### S140 Let’s learn as we go: the lago design for optimizing complex multi-component implementation strategy bundles

#### Donna Spiegelman^1^, Judith Lok^2^

##### ^1^Yale School of Public Health, New Haven, CT, USA; ^2^Boston University, Boston, MA, USA

###### **Correspondence:** Donna Spiegelman (donna.spiegelman@yale.edu)

*Implementation Science 2023,* **18(Suppl 3):**S140

**Background:** In the face of vast numbers of preventable deaths around the world and gaping disparities in their distribution, we cannot afford to run null effectiveness and implementation trials of efficacious interventions. At the start of a trial, it is challenging to specify which and how much of multiple multi-level components of an implementation strategy bundle should be included when investigating implementation of an evidence-based intervention comprised of multiple core components. Standard statistical methods do not allow for adaptation after a trial begins. LAGO facilitates systematic adaptation of implementation strategy bundles to increase equity across diverse contexts.

**Methods:** In LAGO trials (Nevo, Lok, Spiegelman, *Annals of Statistics,* 2021), the components of the complex package are repeatedly optimized in pre-planned stages, until the implementation strategy bundle is optimized for attaining the target outcome goal (e.g. 90% PrEP initiation among those at high HIV risk), subject to fixed power and cost.

**Findings:** We will illustrate key features of the LAGO design with the high profile BetterBirth study (Semrau et al, 2017, *New England Journal of Medicine*), a large Type I hybrid effectiveness-implementation trial aimed at reducing maternal and neonatal mortality through sustained uptake of WHO’s Safe Childbirth Checklist (SCC) among over 170,000 births. BetterBirth investigated a complex multi-level implementation strategy bundle that included health system, provider and patient-level components to improve SCC uptake, and, with post-baseline adaptations prohibited as standard, failed to find a clinically meaningful effect. We will show how the use of LAGO could have prevented this.

**Implications for D&I Research:** The LAGO design allows investigators to adapt, tailor and tweak the bundle of implementation strategies while testing the overall intervention effect, and maintaining or even improving power. The optimized LAGO design also produces an algorithm that allows future studies to tailor the implementation strategy to accommodate variation in background patient-, provider- and/or health system characteristics, thereby increasing equity of the intervention during scale-up and scale-out.

**Primary Funding Source**

National Institutes of Health

### S141 Prep implementation strategies for individuals receiving care from community health clinics in mississippi

#### Trisha Arnold^1^, Kayla K. Giorlando^1^, Laura Whiteley^2^, A. Rani Elwy^2^, Andrew Barnett^1^, James B. Brock^3^, Courtney Sims-Gomillia^3^, Avery Leigland^1^, Larry Brown^2^

##### ^1^Rhode Island Hospital, Providence, RI, USA; ^2^Warren Alpert Medical School of Brown University, Providence, RI, USA; ^3^University of Mississippi Medical Center, Jackson, MS, USA

###### **Correspondence:** Trisha Arnold (trisha_arnold@brown.edu)

*Implementation Science 2023,* **18(Suppl 3):**S141

**Background:** Uptake of Pre-exposure Prophylaxis (PrEP) by those at high risk for contracting HIV has been slow, particularly in Southern states like Mississippi (MS) and especially outside of academic centers. This study investigated facilitators and barriers to PrEP uptake among patients at risk for HIV at community health clinics (CHCs) in MS using components of the integrated Promoting Action on Research Implementation in Health Services (i-PARIHS) framework.

**Methods:** Interviews were conducted with CHC staff and PrEP-eligible patients in MS. The i-PARIHS framework guided interview content comprised of: *recipients* (facilitators and barriers to PrEP use, PrEP knowledge, motivation to take PrEP); *context* (CHC staff PrEP knowledge, structural barriers, and methods for expanding PrEP knowledge and services); and *innovation* (recommendations regarding PrEP care, PrEP education content, and forms of PrEP). Reflexive thematic analysis, organized within NVivo, was used to analyze the data deductively. The i-PARIHS framework and existing methods of delivering PrEP were used *a priori* to determine themes relevant to selecting appropriate implementation strategies from the Expert Recommendations for Implementing Change (ERIC) project and defining mechanisms of change for each strategy.

**Findings:** Thirty-five interviews (18 CHC staff and 17 PrEP-eligible patients from three CHCs) were completed between April 2021 and March 2022. The themes of PrEP knowledge, barriers, and motivation were mapped to i-PARIHS, and strategies from the ERIC project were selected for each implementation construct: PrEP Information Dissemination [conduct educational meetings, distribute educational materials, develop educational materials, involve patients/consumers and family members]; Increase Variety and Number of PrEP Providers [use train-the-trainer strategies, conduct educational meetings]; Enhance PrEP Provider Alliance and Trust [conduct educational meetings, distribute educational materials, chance service sites, develop resource sharing agreements]; and Increase Access to PrEP [change service sites, develop resource sharing agreements]. Mechanisms of change were to increase PrEP awareness, knowledge, acceptance, access, and adherence.

**Implications for D&I Research:** These findings will inform PrEP implementation protocols to increase access, engagement, and adherence to PrEP among individuals at risk for HIV living in MS receiving care from CHCs.

**Primary Funding Source**

National Institutes of Health

### S142 Generating the evidence-base for implementation strategies targeting colorectal cancer screening in the accelerating colorectal cancer screening through implementation science (accsis) research sites

#### Prajakta Adsul^1^, Nidhi Kanabar^2^, Melinda Davis^3^, Borsika Rabin^4^, Aaron Kruse-Diehr^5^, Jill Oliveri^6^, Mark Dignan^7^, Electra Paskett^8^, Dan Reuland^9^, Renée Ferrari^10^, Alexis Moore^11^, Blase Polite^12^, Karen Kim^13^, Samir Gupta^14^, Shiela Castaneda^15^, Maria Elena Martinez^16^, Peter Lance^17^, Jennifer Hatcher^18^, Jennifer Coury^3^, Erin Kenzie^3^, Amanda Petrik^19^, Gloria Coronado^19^, David Liebovitz^12^, Jessica Blanchard^20^, Wynne Norton^21^, Robin Vanderpool^22^, Sonja Hoover^23^, Kevin English^24^, Shiraz Mishra^1^

##### ^1^University of New Mexico, Albuquerque, NM, USA; ^2^University of New Mexico Comprehensive Cancer Center, Albuquerque, USA; ^3^Oregon Health & Science University, Portland, OR, USA; ^4^University of California San Diego, San Diego, CA, USA; ^5^University of Kentucky College of Public Health, Lexington, KY, USA; ^6^Division of Population Sciences, The Ohio State University Comprehensive Cancer Center, Columbus, OH, USA; ^7^University of Kentucky, Lexington, KY, USA; ^8^The Ohio State University, Columbus, OH, USA; ^9^UNC-Chapel Hill, NC, USA; ^10^University of North Carolina at Chapel Hill, Chapel Hill, NC, USA; ^11^The University of North Carolina at Chapel Hill, Chapel Hill, NC, USA; ^12^University of Chicago, Chicago, IL, USA; ^13^The University of Chicago, Chicago, IL, USA; ^14^University of California, San Diego Moores Cancer Center, San Diego, CA, USA; ^15^San Diego State University, San Diego, CA, USA; ^16^The University of California San Diego, La Jolla, CA, USA; ^17^University of Arizona Cancer Center, Tucson, AZ, USA; ^18^The University of Arizona, Tuscon, AZ, USA; ^19^Kaiser Permanente Center For Health Research, Portland, OR, USA; ^20^The University of Oklahoma, Center for Applied Social Research, Norman, OK, USA; ^21^National Cancer Institute, Division of Cancer Control and Population Sciences, Washington DC, WA, USA; ^22^National Cancer Institute, Rockville, MD, USA; ^23^RTI International, North Waltham, MA, USA; ^24^Albuquerque Area Southwest Tribal Epidemiology Center, Albuquerque, NM, USA

###### **Correspondence:** Prajakta Adsul (padsul@salud.unm.edu)

*Implementation Science 2023,* **18(Suppl 3):**S142

**Background**

Implementing evidence-based interventions such as colorectal cancer screening for population-level benefit can be challenging in resource-limited, primary healthcare settings. Part of the challenge is in identifying and studying strategies that address the multilevel, contextual influences on implementation in these healthcare settings. The purpose of this study was to examine the strategies utilized by multiple research sites, funded through the Accelerating Colorectal Cancer Screening through Implementation Science (ACCSIS) initiative that studies the implementation of colorectal cancer screening, follow-up, and referral to care, in primary healthcare settings.

**Methods**

ACCSIS sites completed Excel-based questionnaires within their teams and listed specific activities and approaches used in the implementation of colorectal cancer screening. Each site’s data was reviewed and validated by three implementation science experts that matched these activities to implementation strategies and domains as they have been operationalized and classified in the Experts Recommending Implementation Change (ERIC) study. All matched strategies were then reviewed and confirmed by the research teams, prior to final data analysis. Analyses examined similarities and differences among implementation strategies used by each site and tracked their use across the screening continuum.

**Findings**

Seven ACCSIS sites participated in this study; collectively, they identified 77 implementation strategies (range: 3-17 per site) that helped implement colorectal cancer screening, follow-up, and referrals in primary healthcare settings and matched the ERIC study. Several similarities were noted across sites, for example, four sites developed and distributed educational materials, while three used facilitation as an implementation strategy. Of the nine domains under which the ERIC strategies are classified, most strategies used by the ACCSIS sites fell under the domain of using evaluative and iterative strategies (e.g., conducting a local need assessment), followed by training and education (e.g. provider education). All sites used strategies focused on screening while six sites used strategies to ensure follow-up of screening, and only one site used strategies focused on access to treatment.

**Implications for D&I Research**

Collating strategies across ACCSIS sites enables the development of an evidence-base specifically focused on strategies that address the multi-level influences on colorectal cancer screening and follow-up. Our study findings provide important data to guide real-world implementation efforts, including future scale-up and sustainability.

**Primary Funding Source**

National Institutes of Health

### S143 Reducing mortality due to non-communicable diseases during climate-induced disasters: a participatory group model building approach

#### Saria Hassan

##### Yale School of Medicine, New Haven, CT, USA

###### **Correspondence:** Saria Hassan (saria.hassan@emory.edu)

*Implementation Science 2023,* **18(Suppl 3):**S143

**Background:** At least one third of the mortality after severe weather events such as hurricanes is due to poorly managed non-communicable diseases (NCDs). With the advent of climate change the world will experience more severe extreme weather events. There is an urgent need for implementation strategies to improve survival of people living with NCDs in the face of natural disasters. Our objective is to use participatory group model building (GMB) to identify strategies that can address this need.

**Methods:** GMB is a participatory method within system dynamics that engages stakeholders in elaborating complex problems and identifying potential places to intervene. Stakeholders in the US territories of Puerto Rico and the US Virgin Islands were convened virtually over the course of 6-months to develop causal loop diagrams for each island to understand the causes and consequences of poorly managed NCDs in disasters. These diagrams were used to identify and prioritize interventions. Stakeholders were identified based on their experience with the 2017 Hurricanes Irma and Maria and their current role in disaster preparedness/response and management of NCDs.

**Findings:** An average of 7 stakeholders attended each meeting. They represented Federally Qualified Health Centers (FQHC), federal emergency management agencies, housing authorities, faith-based organizations, and Department of Health. Common challenges to NCD management included inadequate access to medication, limited ability to self-manage disease, negative effect of mental health on NCDs, limited accessibility of healthy food options, and lack of adequate preparedness. Strategies to address challenges were identified at the following levels: individual -- strengthen capacity to self-manage disease; FQHC – educational material and outreach for mental health and psychosocial support; system/policy – payment/reimbursement for services and medication, and resources to access healthy food and safe shelter.

**Implications for D&I Research:** GMB is a structured and effective way of engaging multisectoral stakeholders to identify feasible and impactful strategies for complex systems such as that of managing NCDs in climate-induced disasters. We identified challenges common to both US territories and strategies that can be implemented to overcome them. This approach can be scaled out to other settings where climate change is bound to bring in more severe droughts, heat, and extreme weather events.

### S144 Assessing the implementation of portable air cleaners (pac) as an intervention to reduce wildfire smoke exposure through the consolidated framework for implementation research (cfir)

#### Tania Busch Isaksen^1^, Kathleen Moloney^2^

##### ^1^University of Washington, School of Public Health, Seattle, WA, USA; ^2^University of Washington, Seattle, WA, USA

###### **Correspondence:** Kathleen Moloney (kmoloney@uw.edu)

*Implementation Science 2023,* **18(Suppl 3):**S144

**Background:** As the frequency and severity of wildfires and associated wildfire smoke (WFS) exposure is expected to increase in the coming years, the application of implementation science to study interventions to reduce the negative impacts of WFS exposure is of critical importance. The Methow Valley, a rural Washington state community, has increasingly faced extended periods of poor air quality due to wildfire activity. In 2021, 2,000 HEPA portable air cleaners (PAC) were distributed to Methow Valley residents in response to a particularly severe WFS season. This research aims to assess the implementation of PAC as an intervention to reduce WFS exposure using the Consolidated Framework for Implementation Research (CFIR).

**Methods:** During the summer of 2021, a subset of PAC recipients completed a baseline survey that assessed perceived risk, health, and wellbeing impacts of WFS exposure and anticipated impacts of the PAC on their household. PAC recipients who indicated willingness to participate in future research were contacted for participation in two additional surveys in 2022, administered pre- and post-wildfire season, to assess implementation outcomes and determinants. The development of these surveys was guided by CFIR constructs focused within the domains of characteristics of individuals (knowledge and beliefs, self-efficacy) and process (executing).

**Findings:** A majority of the 2021 survey respondents (N=679) were highly or moderately concerned about WFS exposure impacts personally, for friends and family, and for the broader community. These respondents also reported anticipating positive impacts of the PAC on their household, including: reduced risk of long-term health impacts from smoke (86%), improved wellbeing (82%), help managing physical symptoms from smoke (80%), and reduced stress levels (69%). Surveys conducted pre- and post-2022 wildfire season provided follow-up data on how recipients used PACs, barriers and facilitators to use, and risk perceptions and health and wellbeing impacts related to WFS exposure.

**Implications for D&I Research:** This research demonstrates the application of implementation science to elucidate barriers and facilitators of a household-level intervention to reduce WFS exposure. This example can guide future implementation science research focusing on interventions to mitigate the negative impacts of exposure to climate change-related disasters in other contexts.

**Primary Funding Source**

National Institutes of Health

### S145 Lessons learned from cooling center initiatives to protect vulnerable populations from the arizona heat

#### Hsini Lin

##### Arizona Department of Health Services, Phoenix, AZ, USA

###### **Correspondence:** Hsini Lin (hsini.lin@azdhs.gov)

*Implementation Science 2023,* **18(Suppl 3):**S145

**Background:** Many communities in Arizona face challenges from chronic heat each summer. In 2021, Phoenix and Tucson experienced 104 days and 63 days over 100 degrees, respectively. These high temperatures contributed to 552 heat-related deaths, and another 2,873 heat-related emergency room visits statewide. Heat-related illness and death are preventable. Access to cool environments, even for a few hours, can be protective in mitigating harmful effects from extreme heat. Therefore, cooling centers can be an effective public health intervention in reducing heat risks. The CDC’s Building Resilience Against Climate Effect (BRACE) framework is used to evaluate and improve the effectiveness of cooling centers in Arizona.

**Methods:** An intervention evaluation process was implemented to advance public health’s understanding of Arizona cooling center networks, their operation and use, and feedback for expansion to better serve vulnerable populations. A team consisting of state and county health department staff and academia worked with partners and stakeholders to gather local perspectives on heat management to assist in planning cooling center operations. This cross-sector collaboration developed new partnerships to support free cooling center transportation, improved data infrastructure for sharing physical heat relief resources, centralized and standardized the mapping and on-boarding process for cooling centers, and improved cooling center signage. Additionally, an optimization analysis was performed to guide recruitment efforts to add cooling centers in locations with more vulnerable populations.

**Findings:** Arizona’s BRACE work showed that populations at greater risk to extreme heat are those experiencing homelessness, older adults, socially isolated, and individuals who cannot afford to cool their homes because of their frequent and prolonged exposure to heat, sensitivity to heat, and ability to adapt. The results from the field observations survey, visitor survey, and facility manager survey indicated that awareness, transportation, location, operating schedules, pets, possessions, and substance use all represent barriers to the use and efficacy of cooling centers.

**Implications for D&I Research:** This research demonstrates an effective use of cross-sector collaboration to evaluate interventions to better understand utilization and gaps in the knowledge base. This example can guide future studies determine how to evaluate climate and health interventions and engage a cross-disciplinary team.

**Primary Funding Source**

Centers for Disease Control and Prevention

## Promoting Health Equity and Eliminating Disparities

### S146 Design and pilot implementation of the achieving cancer equity through identification, testing, and screening (ace-its) program in an urban under-resourced population

#### Suzanne O'Neill^1^, Chiranjeev Dash^2^, Mary Mills^2^, Rhonda Hamilton^2^, Alejandra Hurtado de Mendoza^2^, Thelma Jones^3^, Ify Nwabukwu^4,5^, Jacqueline Beale^6,7^

##### ^1^Georgetown University, Washingtonn, DC, USA; ^2^Lombardi Comprehensive Cancer Center, Washington, DC, USA; ^3^Thelma D. Jones Breast Cancer Fund, Washington, DC, USA; ^4^African Women’s Cancer Awareness Association, Greenbelt, MD, USA; ^5^Community Navigator, Washington, DC, USA; ^6^Cancer to Jasmine and Butterflies Consulting, Glenn Dale, MD, USA; ^7^American Cancer Society, Washington, DC, USA

###### **Correspondence:** Suzanne O'Neill (sco4@georgetown.edu)

*Implementation Science 2023,* **18(Suppl 3):**S146

**Background:** The Achieving Cancer Equity through Identification, Testing, and Screening (ACE-ITS) program is a community-engaged quality improvement framework to improve mammography maintenance and rates of genetic risk assessment, counseling, and testing using a multi-level approach that enhances the currently existing patient navigation system through mHealth and community education.

**Methods:** The ACE-ITS program is based on the National Institute of Minority Health and Health Disparities (NIMHD) Research Framework that emphasizes multilevel models focused on the individual (genetic testing, screening navigation) and community (community-based breast health education) levels and targeted to the biological (genetic risk), behavioral (mammography screening), sociocultural (underserved Black and Hispanic women), and the health care system (patient navigation, automated text messages) related domains. We further integrate the Practical Robust Implementation and Sustainability Model (PRISM) model to describe our program design, the external environment, implementation and sustainability infrastructure, and program recipients.

**Findings:** In collaboration with genetic counselors and community partners, we created educational modules on mammography maintenance and genetic counseling and testing that have been incorporated into the navigator-led community education sessions. We also implemented a universal genetic risk assessment tool and automated text message reminders for repeat mammograms into our mammography navigation workflow. Through the ACE-ITS program implementation, we have collaboratively conducted 22 educational sessions, identified more than 650 women non-adherent to breast cancer screening guidelines, and navigated 585 women to mammography screening over the 2020-2021 calendar years. From January-December 2021 we conducted genetic risk assessment on 292 women. Of these, 70 women screened positive for further assessment and 58 met eligibility for genetic counseling. Of these, seven (12%) have received genetic counseling/testing.

**Implications for D&I Research:** We describe a multi-level community-engaged quality improvement program designed to reduce screening-related disparities in Black and Hispanic women in our catchment area. Future directions include creating and disseminating an implementation strategies toolkit for breast cancer screening provider organizations in other under-resourced minority communities and addressing barriers as a means to increase rates of genetic counseling and testing among eligible women.

**Primary Funding Source**

American Cancer Society/Pfizer

### S147 Cultural adaption and implementation of an evidence-based lifestyle intervention for vulnerable rural Appalachian residents

#### Ming-Yuan Chih^1^, Deanna Sherman^1^, Angela Pfammatter^2^, Michelle Roberts^1^, Sarah Vos^1^, Bonnie Spring^3^, Nancy Schoenberg^4^

##### ^1^University of Kentucky, Lexington, KY, USA; ^2^Northwestern University, Chicago, IL, USA; ^3^Northwestern University School of Law, Chicago, IL, USA; ^4^University of Kentucky College of Medicine, Lexington, KY, USA

###### **Correspondence:** Ming-Yuan Chih (m.chih@uky.edu)

*Implementation Science 2023,* **18(Suppl 3):**S147

**Background:** Rural Appalachian residents experience among the nation’s highest prevalence of chronic diseases, premature mortality and decreasing life expectancy. Addressing these growing inequities while avoiding duplicating existing programming and deploying evidence-based interventions (EBI) necessitates a rigorous culturally adaptation process. Yet, few publications or presentations explicate how to undertake this process. We provide insights and data on the adaptation process and implementation protocol of Make Better Choices 2 (MBC2). MBC2, an mHealth diet and activity intervention initially designed for an urban population, includes an app, health coaching, accelerometer, and behavioral incentives.

**Methods:** Cultural adaptation was based on the NIH’s Cultural Framework of Health and Aarons’ Dynamic Adaptation Process. We employed an iterative process, engaging in six sequential steps to assess the MBC2 intervention’s acceptability, feasibility, and need for cultural and contextual adaptation. These steps include focus groups, key informant interviews (KII), community advisory board (CAB) verification, wireframing, usability testing, and pilot testing.

**Findings:** Focus groups (4 groups, 38 participants), KII (N=16), and later verification with a CAB (N=9) revealed seven areas in need of adaption, including existing approaches to eligibility, recruitment, and MBC2 programmatic components. Recommendations included (1) revising age eligibility; (2) focusing on Facebook for recruitment; (3) deploying group activities in addition to individual enrichment; and (4) training and employing only lay, local coaches who have familiarity with sparse resources, and others. Wireframing (N=8) uncovered confusion over the app’s depiction of targeted behaviors (diets, physical activity, and sedentary behavior) and how to enter data, fostering multiple rounds of modification. A REDCap usability survey conducted with the CAB (N=9) refined design issues, e.g., data visualization. Finally, we conducted a brief 6-week pilot study (N=10) of the adapted EBI, which demonstrated the feasibility and appropriateness. We provide a description of the process, adaptions, and protocol in this presentation.

**Implications for D&I Research:** To propel implementation science forward, more explicit descriptions of the process and outcomes of culturally adapted EBI are needed. This approach has reached a balance between local fit and fidelity with the existing EBI. To achieve health equity among vulnerable rural residents and all groups experiencing inequities requires such a systematic approach prior to deploying the EBI.

**Primary Funding Source**

National Institutes of Health

### S148 Barriers and facilitators to the implementation of a community-clinic linkage model for hypertension management among blacks in a new york city network of churches: a faith-based perspective

#### Claire Cooper^1^, Joyce Gyamfi^2^, Deborah Onakomaiya^3^, Aigna Barber^4^, Linda Thompson^5^, Roger Abrams^5^, Jennifer Zanowiak^6^, Moses Mansu^4^, Laura Diaz^7^, Miriam Gofine^8^, Wen-Yu Lee^4^, Antoinette Schoenthaler^9^, Gbenga Ogedegbe^10^, Nadia Islam^6^

##### ^1^New York University Grossman School of Medicine, New York, NY, USA; ^2^New York University School of Global Public Health, New York, NY, USA; ^3^Vilcek Institute of Graduate Biomedical Sciences, New York University Grossman School of Medicine, New York, NY, USA; ^4^Institute for Excellence in Health Equity New York University Grossman School of Medicine, NEW YORK, USA; ^5^Institute for Excellence in Health Equity New York University Grossman School of Medicine, NEW YORK, NY, USA; ^6^Department of Population Health, New York University Grossman School of Medicine, New York, USA; ^7^Institute for Excellence in Health Equity New York University Grossman School of Medicine, New York, NY, USA; ^8^Vilcek Institute of Graduate Biomedical Sciences, NYU Grossman School of Medicine, New York, NY, USA; ^9^NYU Langone Health, New York, USA; ^10^Department of Population Health, New York University Grossman School of Medicine, New York, NY, USA

###### **Correspondence:** Claire Cooper (claire.cooper@nyulangone.org)

*Implementation Science 2023,* **18(Suppl 3):**S148

**Background:** US Black communities face the nation’s highest hypertension burden. Although achieving hypertension control is requisite for improving this disparity, barriers exist at the individual-, community- and healthcare-levels. Community-Clinic Linkage Models (CCLMs) represent a strategy for improving the adoption/effectiveness of evidence-based interventions by linking a broad range of stakeholders within community/clinical settings, to improve hypertension control at multiple levels of influence. This study aimed to assess community-level factors that may influence effective implementation of a CCLM in a primary care practice network within a New York City (NYC)-based health system, by seeking key stakeholder perspectives from NYC faith-based organizations (FBOs).

**Methods:** Fifteen participants representing 11 NYC FBOs (2 religions/5 denominations) were engaged in 1:1 interviews, using a semi-structured moderator’s guide, to assess community-level factors influencing implementation of a CCLM among Black-hypertensive patients residing in FBO catchment areas. Constructs from the Health Belief Model (HBM) (*perceived susceptibility/severity/benefits/barriers*; *self-efficacy*) were pragmatically applied to guide preliminary content-analyses of the interviews.

**Findings:** Participants were mostly female (83%), Black (93%), and age 65+ (53%). Five key themes emerged from the interviews. Stakeholders characterized hypertension as a community-wide problem, indicating *Black Americans are at higher risk* (HBM: perceived susceptibility), and describing hypertension as *widespread* and often *uncontrolled* among their congregants/communities (HBM: perceived severity). Participants believed that using a CCLM to implement a hypertension program would benefit both congregations (e.g., through *increasing capacity/knowledge-building, improved reach, capitalizing on* r*eputation/legitimacy)* and community members (e.g., through *increased awareness/access to resources/services, improved health/quality-of-life, increased self-efficacy)* (HBM: perceived benefits)*.* Perceived barriers (HBM) to implementation were identified at the congregational-level (*financial constraints, consistency/sustainment of program, capacity, reach at the organizational-level)* and at the community-level (*limited knowledge, financial/technological/healthcare barriers; and lifestyle factors influencing risk).* Finally, self-efficacy (HBM) was characterized as an important facilitator at the congregational-level *(leadership buy-in, supportive congregational structures)* but also identified as a potential implementation barrier *(limited experience with hypertension program, limited manpower).*

**Implications for D&I Research:** Stakeholders provided rich insights on community-level factors influencing the implementation of a CCLM to improve hypertension management in NYC Black communities. Findings provide insight on integrating stakeholder feedback to better tailor the CCLM, to ensure adoption/sustainability.

This-study-received-ethical-approval-from-NYU-School-of Medicine-Institutional-Review-Board.

This-study-is-ongoing;-full-data-collection/analysis-will-be-complete-and-ready-for-presentation-by-10/1/22.

**Primary Funding Source**

National Institutes of Health

### S149 Exploring the inner and outer context factors associated with disaster preparedness and response in federally qualified health centers in the usvi and puerto rico

#### Saria Hassan^1^, Myrna del Mar Gonzalez-Montalvo^2^, Karla Escobar^2^, Tess Richards^3^, Jean M. Ortiz^4^, Hector L. Villanueva^4^, Adithya Cattamanchi^5^, Marcella Nunez-Smith^6^

##### ^1^Emory University School of Medicine, Atlanta, GA, USA; ^2^Emory Rollins School of Public Health, Atlanta, GA, USA; ^3^St Thomas East End Medical Center, St Thomas, Virgin Islands (U.S.); ^4^HealthproMed, San Juan, PR, USA; ^5^University of California San Francisco, San Francisco, CA, USA; ^6^Yale School of Medicine, New Haven, CT, USA

###### **Correspondence:** Saria Hassan (saria.hassan@emory.edu)

*Implementation Science 2023,* **18(Suppl 3):**S149

**Background:** At least one third of the deaths after Hurricanes Irma and Maria were due to poorly managed non-communicable diseases (NCDs). With the advent of climate change, more severe weather events threaten to worsen existing health disparities. Federally Qualified Health Centers (FQHCs) provide care to the most vulnerable including individuals living with NCDs. Our objective is to understand the inner and outer context factors associated with the disaster preparedness and response of FQHCs.

**Methods:** We use the Exploration, Preparation, Implementation, and Sustainment (EPIS) Framework to guide our work. In the Exploration phase we conducted a qualitative study to understand how factors in the outer context (sociopolitical, funding, and network) and inner context (organizational characteristics, culture, leadership, and individual-level factors) were associated with disaster preparedness and response in two FQHCs in Puerto Rico and the US Virgin Islands. Staff, administrators, healthcare providers, nurses, and patients with NCDs who experienced the 2017 hurricanes were invited to participate in in-depth qualitative interviews. Interviews were recorded and transcribed. A thematic analysis approach was used, and emergent themes mapped onto factors within the Exploration phase of EPIS.

**Findings:** Twenty staff/providers/administrators and 10 patients were interviewed. Leadership that equally valued the wellbeing of its employees and its patients, baseline knowledge/skills due to prior experiences, as well as a culture of constant improvement were deemed important for disaster preparedness and response. At the individual level, employees who had support at home were more engaged; they valued the importance of their job for the well—being of patients but acknowledged the challenge of concurrently ensuring the safety of family members. In the outer context, interorganizational networks with relief agencies, emergency response teams, and community-based organizations were critical to ensuring adequate preparedness. Policies allowing access to medication/services outside of insurance were deemed critical for patients living with NCDs in disasters.

**Implications for D&I Research:** The Exploration phase of EPIS can be successfully applied to understand organizational needs and potential solutions to improve disaster preparedness for vulnerable groups. The disproportionate effect of climate-induced disasters on minority populations will worsen health disparities unless we develop scale-able, evidence-based strategies to improve preparedness and response for the most vulnerable.

**Primary Funding Source**

National Institutes of Health

### S150 Impact of an implementation support intervention on community clinics’ adoption of social risk screening: stepped-wedge trial results

#### Rachel Gold^1^, Erika Cottrell^2^, Miguel Marino^3^, Jorge Kaufmann^3^, Arwen Bunce^2^, Suzanne Morrissey^4^, Laura Gottlieb^5^, Christina Sheppler^6^, Megan Hoopes^2^, Molly Krancari^7^, Megan Bowen^2^, Ned Mossman^2^

##### ^1^Science Program, Kaiser Permanente Center for Health Research, Portland, OR, USA; ^2^OCHIN, Inc., Portland, OR, USA; ^3^Oregon Health & Science University, Portland, OR, USA; ^4^OCHIN, Inc, Portland, OR, USA; ^5^University of California, San Francisco, San Francisco, CA, USA; ^6^Kaiser Permanente Center for Health Research, Portland, OR, USA; ^7^OCHIN, Inc., Irvine, CA, USA

###### **Correspondence:** Rachel Gold (rachel.gold@kpchr.org)

*Implementation Science 2023,* **18(Suppl 3):**S150

**Background:** Screening for social risks (adverse social determinants of health) in clinical settings is widely recommended, but difficult to implement. We tested whether a 6-month implementation support intervention (technical assistance, practice coaching, adaptive support, five-step process) increased adoption of social risk screening. This is the first trial of multi-component implementation support targeting this outcome.

**Methods:** Community health center (CHC) clinics with a shared electronic health record (EHR) (n=31) were block-randomized in this pragmatic stepped-wedge trial. The first of 6 intervention wedges began in September 2018. Quantitative data were extracted from the shared EHR. The primary outcome was the monthly rate of patients with clinic encounters for whom any social risk data were documented in the EHR, assessed from 6 months prior to the first wedge’s intervention through 6 months after the last wedge’s intervention concluded (study period 3/2018-12/2021). Negative binomial mixed-effects modeling assessed intervention effects in clinics that had versus had not yet participated in the intervention; these models accounted for a general time trend and assessed sustainment of intervention effect post-intervention. Qualitative data were collected for a mixed methods realist evaluation of context-specific pathways through which the intervention impacted adoption of targeted activities.

**Findings:** In the 6 months of active intervention, intervention CHCs more than doubled their social risk screening rates compared to control CHCs (p<0.01), but this impact was not sustained in the period following intervention. The realist evaluation identified relationships between logistical, contextual, and systemic factors and intrinsic motivations that exceeded the supports of the implementation intervention and posed challenges to social risk screening. Notably, the COVID pandemic began during study wedge 4, which should influence interpretation of these results.

**Implications for D&I Research:** Systematic social risk screening could help mitigate the health impacts of social risks, but clinics encounter multi-level barriers to implementing screening practices. Intensive support can help clinics launch social risk screening programs; in this trial, intensive support was highly impactful while it was provided, but six months of support were inadequate to sustain adoption. Stakeholders aiming for universal social risk screening should consider adequate and ongoing resources to support implementation to overcome substantial barriers.

**Primary Funding Source**

National Institutes of Health

### S151 Strategies to support implementation of social risk screening in community health care settings: a realist-informed evaluation

#### Arwen Bunce^1^, Suzanne Morrissey^2^, Molly Krancari^3^, Erika Cottrell^1^, Rachel Gold^4^

##### ^1^OCHIN, Inc., Portland, OR, USA; ^2^OCHIN, Inc, Portland, OR, USA; ^3^OCHIN, Inc., Irvine, CA, USA; ^4^Science Program, Kaiser Permanente Center for Health Research, Portland, OR, USA

###### **Correspondence:** Arwen Bunce (buncea@ochin.org)

*Implementation Science 2023,* **18(Suppl 3):**S151

**Background:** Despite increasing interest in social risk (adverse social determinants of health) screening, little is known about how to support clinics in implementing social risk data collection and documentation. We tested the impact of a customized five-step approach to implementing social risk activities in 31 community health centers using tailored electronic health record training, change management support, and intervention materials.

**Methods:** A realist-informed evaluation identified context-specific pathways through which the tailored implementation support impacted the systematic collection and integration of social risk data. To limit clinic burden, data were derived from clinic interactions with the implementation support team (IST), including recordings / transcripts from phone check-ins and group discussions as well as email exchanges. We also conducted regular IST debrief sessions that were recorded and transcribed. Data collection and analysis involved iterative cycles of theory generation, testing, and refinement. Initial program theories were tested against and refined through engagement with the data. The objective was to move toward explanatory theories with interpretive validity: explanations of why and how the intervention worked the way it did, for whom, and in what circumstances.

**Findings:** We identified three theories that explain why and how this intervention impacted the collection of social risk data. 1) Standardized social risk screening creates tension between staff visions of good care (relationship-based, patient-centered) and pragmatic structural constraints. Clinics that were able to bridge this tension by using the screening process to facilitate patient-provider rapport were more likely to engage deeply with the process. 2) Relationships between IST members, the IST and clinic champions, and clinic champions and staff were key to uptake. Strong, respectful relationships empowered clinic staff to experiment with workflows that could work in their settings. 3) The materiality of intervention materials (i.e., workbooks, summaries) anchored collaborative clinic discussions and acted as legitimizing “proof of work” for clinic champions.

**Implications for D&I Research:** A key priority of implementation science is identifying the mechanisms by which implementation strategies exert their effects. While community health care clinicians have long considered social risk when providing care, this realist-informed evaluation advances our understanding of how to best support that work.

**Primary Funding Source**

National Institutes of Health

### S152 Stakeholder engagement in the design of EHR tools for social care

#### Maura Pisciotta^1^, Rose Gunn^1^, Rachel Gold^2^, Erika Cottrell^3^, Mary Middendorf^3^, Danielle Hessler^4^, Arwen Bunce^3^, Laura Gottlieb^5^

##### ^1^OCHIN, Inc, Portland, OR, USA; ^2^Science Program, Kaiser Permanente Center for Health Research, Portland, OR, USA; ^3^OCHIN, Inc., Portland, OR, USA; ^4^Department of Family Community Medicine, UCSF School of Medicine, San Francisco, CA, USA; ^5^University of California, San Francisco, San Francisco, CA, USA

###### **Correspondence:** Maura Pisciotta (pisciottam@ochin.org)

*Implementation Science 2023,* **18(Suppl 3):**S152

**Background:** Social context like the availability of food, housing, and transportation can affect patients’ ability to act on care plans. A 2019 National Academy of Sciences, Engineering, and Medicine report recommends that health care teams adjust care plans based on these kinds of contextual risks. In care settings such as community health centers (CHCs), which primarily serve under-resourced patient populations, automated clinical decision support (CDS) tools that suggest care plan adjustments may serve as effective implementation strategies for increasing the adoption of such care plan adjustments that seek to improve health outcomes. We engaged a diverse group of clinical stakeholders from CHCs with the goal of developing a suite of EHR-based CDS tools that might facilitate the adoption of social risk-informed care plan adjustments.

**Methods:** We conducted a systematic scoping review of hypertension and diabetes clinical guidelines to identify guideline-recommended care plan adjustments related to patients’ social context. Results of the clinical guideline reviews informed focus group discussions and individual interviews about CDS tool development. Stakeholders provided feedback on how the potential CDS tools' content, form, and function could be optimized to support CHC teams’ adoption of social risk-informed care.

**Findings:** Stakeholders universally prioritized tool content specific to social risk screening and documentation. Other prioritized content related to supports for i) adjusting medication costs; ii) changing follow-up visit plans based on social risks; and iii) encouraging dialogue between providers and patients about social risks and care plan adjustments. Recommended tool functions included alerts and shortcuts to support and document care plan adjustments.

**Implications for D&I Research:** Prior D&I research assessing the potential of using automated EHR-based tools to enhance the adoption of social risk-informed care is limited. The CDS tools developed in this stakeholder-driven development process are an innovative implementation strategy for supporting clinical guideline-based care plan adjustments in response to patients’ social risks. These CDS tools may support CHC providers in systematically implementing care adjustments to enhance patients’ ability to adhere to care plans.

**Primary Funding Source**

National Institutes of Health

### S153 Sdh data as “clues” to patient-centered care: stakeholder input on ehr-based clinical decision support tools as an implementation strategy

#### Suzanne Morrissey^1^, Arwen Bunce^2^, Erika Cottrell^2^, Maura Pisciotta^1^, Laura Gottlieb^3^, Rachel Gold^4^

##### ^1^OCHIN, Inc, Portland, OR, USA; ^2^OCHIN, Inc., Portland, OR, USA; ^3^University of California, San Francisco, San Francisco, CA, USA; ^4^Science Program, Kaiser Permanente Center for Health Research, Portland, OR, USA

###### **Correspondence:** Suzanne Morrissey (morrisseys@ochin.org)

*Implementation Science 2023,* **18(Suppl 3):**S153

**Background:** Social risks (e.g., transportation, food, and housing insecurity) influence patient engagement in and adherence to care plans. Yet little research explores ways to incorporate information about these contextual risks into care planning, including by using EHR-based clinical decision support (CDS) tools as an implementation strategy. This paper presents stakeholder recommendations for, perceptions of, and experiences with a suite of CDS tools designed to enhance adoption of social risk-informed care in community health centers (CHCs) collected during the design and pilot phases of a clinical trial of these tools (R01MD014886).

**Methods:** We conducted a realist evaluation with clinic staff and leadership to assess whether and how CDS tools could/did support contextualized, social risk-informed care for patients with social risks. The tools were developed to support the provision of care plan adjustments targeting social risks’ impact on health through a stakeholder design process. Then in Fall 2021-Spring 2022, three CHCs pilot-tested these tools. Data sources consisted of semi-structured interviews, group discussions and facilitated check-ins with the pilot CHCs. As insights emerged from the pilot evaluation, we revised our initial program theories and returned to the stakeholder data to test their validity. We then applied new knowledge to refine the tools for the trial phase of the study and future implementation efforts.

**Findings:** Our original hypothesis was that these CDS tools would help CHC staff make care plan adjustments for patients with health-related social risks. Stakeholder input, however, suggested that the tools support staff through more indirect pathways, including by helping them communicate with patients and colleagues about needed adjustments. These findings underscore how CDS tools targeting such care plan adjustments can support both patient-clinician relationships and the use of social risk data as “clues” to initiate conversations with patients, establish trust and confidence, and enable productive transfer of knowledge for care planning.

**Implications for D&I Research:** CDS tools can facilitate the implementation of social risk-informed care but need to be designed specifically to meet stakeholder needs. Clinical stakeholders indicated that these needs may be more relationship and communication-focused than social risk content-specific.

**Primary Funding Source**

National Institutes of Health

### S154 Adapting and reporting on implementation strategies to enhance intervention adoption in community health centers

#### Molly Krancari^1^, Rachel Gold^2^, Arwen Bunce^3^, Laura Mae Baldwin^4^, Meg Bowen^5^, Charisma Jenkins^6^, Amber Haley^7^

##### ^1^OCHIN, Inc., Irvine, CA, USA; ^2^Science Program, Kaiser Permanente Center for Health Research, Portland, OR, USA; ^3^OCHIN, Inc., Portland, OR, USA; ^4^University of Washington, Seattle, WA, USA; ^5^OCHIN, Inc., Portland, USA; ^6^Kaiser Permanente - Center for Health Research, Portland, USA; ^7^Gillings School of Global Public Health, University of North Carolina at Chapel Hill, Chapel Hill, NC, USA

###### **Correspondence:** Molly Krancari (krancarim@ochin.org)

*Implementation Science 2023,* **18(Suppl 3):**S154

**Background**

There is growing awareness in implementation science of the need to report on adaptations / modifications made to implementation strategies in practice. Such changes may be necessary to enhance strategies’ ability to support the adoption of evidence-based innovations. However, they are rarely reported in the literature with enough granularity to inform future users of when / what adaptations may be necessary to enhance implementation strategies’ impact. We tracked how a set of strategies were adapted over a six-month implementation support intervention (technical assistance, practice coaching, five-step process) designed to support community health centers’ (CHCs) adoption of systematized screening for social risks (adverse social determinants of health).

**Methods**

Between 9/2018 and 7/2021, our pragmatic stepped-wedge trial offered a six-month intervention to 31 clinics, randomized into six sequential wedges. We systematically tracked changes made to the intervention’s implementation strategies following the Haley method (Haley et al 2021). This method modifies standard D&I methods (FRAME, CFIR, Proctor, ERIC) to report on adaptations / modifications made to implementation strategies, including the reason for and nature of each adaptation made, and its affected actors, target, and outcome. The Haley method covers five overarching components: 1) describe planned strategy; 2) track its use; 3) monitor barriers; 4) describe modifications; and 5) identify / describe new strategies.

**Findings**

Preliminary analyses indicate that most strategy adaptations centered around how CHC staff were oriented to and trained in the use of electronic health record-based tools involving social risk screening. We will present completed analyses of the implementation strategy adaptations / modifications made throughout the intervention. This will include: 1) themes and patterns in the adaptations made both within a given clinic support period and between wedges, 2) which strategies most often required adaptations, and 3) the types of needed adaptations. All reporting will follow the Haley method.

**Implications for D&I Research**

Documenting implementation strategy adaptations / modifications is needed to replicate implementation studies’ results. This work could inform future implementers on how implementation strategies may need to be adapted in practice to successfully support innovation adoption. This is one of the first formal evaluations of implementation strategy adaptation.

**Primary Funding Source**

National Institutes of Health

### S155 Are we being pragmatic enough? Lessons learned from sites lost in a pragmatic clinical trial

#### Elizabeth Austin^1^, Elsa Briggs^1^, Jessica Chen^2^, Lori Ferro^3^, Geoffrey Curran^4^, Andrew Saxon^5^, John Fortney^6^, Dr. Anna Ratzliff^2^, Emily Williams^7^

##### ^1^Department of Health Systems and Population Health, University of Washington, Seattle, WA, USA; ^2^University of Washington, Seattle, WA, USA; ^3^University of Washington, Seattle, USA; ^4^University of Arkansas for Medical Sciences, Little Rock, AR, USA; ^5^VA Center of Excellence in Substance Addiction Treatment and Education, Seattle, WA, USA; ^6^VA Puget Sound Health Care System, Seattle, WA, USA; ^7^VA Puget Sound Health Care System, Health Services Research & Development, Center of Innovation for Veteran-Centered & Value-Driven Care, Seattle, WA, USA

###### **Correspondence:** Elizabeth Austin (austie@uw.edu)

*Implementation Science 2023,* **18(Suppl 3):**S155

**Background**: Research that draws on the real-world conditions of care delivery can generate data that supports rapid translation of evidence to practice. Too often though, research occurs in academic settings, leaving out pivotal community-based perspectives. In 2019, we launched a national hybrid type 1 effectiveness-implementation trial of collaborative care for patients with co-occurring opioid use and mental health disorders in primary care settings. Early in the trial’s launch, 3 of the 12 participating sites dropped out, including two of our three safety net primary care clinics serving more diverse patient communities. We conducted exit interviews with staff at these community-based sites to better understand barriers and facilitators to study participation.

**Methods**: Key informant interviews (n=7) were conducted between October-December of 2021 with clinical and administrative staff from exited sites. Fieldnotes - collected via ongoing formative evaluation – included participant observation of all implementation-related meetings with study sites. Exit interviews asked participants to reflect on experiences with study participation, implementation of study-related practice change, and their decision-making around leaving the study. All interviews were professionally transcribed, and qualitative data from interviews and fieldnotes were analyzed using a Rapid Assessment Process. Two trained qualitative researchers coded data using structured templates guided by the Consolidated Framework for Implementation Research (CFIR) and iteratively reviewed with multiple team members until themes were confirmed.

**Findings**: We identified 3 themes that characterize the unique experiences of community sites unable to continue their trial participation: 1) the work of research threatens community sites’ most precious (and at risk) resource – staff; 2) community site staff lack comfort with and skills for research-related tasks, and 3) research participation in its current form does not offer a clear return on investment for community sites. Impacts from the COVID-19 pandemic pervaded each of these themes in unique ways.

**Implications for D&I Research:** While these findings only reflect the experiences of three community sites, they signal that future trials may wish to consider more robust implementation supports that address barriers to community site engagement. This includes rethinking traditional approaches to research design and evaluation to augment equitable inclusion of diverse practice settings in pragmatic research.

**Primary Funding Source**

National Institutes of Health

### S156 Engaging stakeholders to inform national implementation of critical time intervention (cti) in a program serving homeless-experienced veterans

#### Sonya Gabrielian^1^, Kristina Cordasco^1^, Lauren Hoffmann^1^, Taylor Harris^1^, Ronald Calderon^1^, Jenny Barnard^1^, David Ganz^1,4^, Erin Finley^2,3^, Tanya Olmos-Ochoa^4^

##### ^1^VA Greater Los Angeles, Los Angeles, CA, USA; ^2^South Texas Veteran Health Care System, San Antonio, TX, USA; ^3^University of Texas Health San Antonio, San Antonio, USA; ^4^David Geffen School of Medicine, University of California at Los Angeles, Los Angeles, CA, USA

###### **Correspondence:** Sonya Gabrielian (Sonya.Gabrielian@va.gov)

*Implementation Science 2023,* **18(Suppl 3):**S156

**Background**

The VA Grant and Per Diem Case Management “Aftercare” program provides six months of case management for homeless-experienced Veterans (HEVs) transitioning to permanent housing, with the aim of decreasing returns to homelessness. Implementing Critical Time Intervention (CTI)—an evidence-based case management practice—would standardize care across the 128 community-based agencies that provide Aftercare services. To prepare for national CTI implementation in Aftercare, we conducted a four-agency implementation pilot in which we adapted a CTI implementation package (training and external facilitation); assessed stakeholder perspectives regarding CTI’s acceptability and appropriateness; and characterized contextual factors that affected CTI implementation.

**Methods**

We conducted 67 semi-structured interviews at pre-implementation, mid-implementation, and six months post-implementation, with HEVs (n=37), case managers (n=16), supervisors (n=10), and VA leaders (n=4). Using rapid qualitative analyses, we assessed satisfaction with CTI and our implementation package, and contextual factors influencing CTI implementation.

**Findings**

HEVs expressed goals that aligned with CTI principles (e.g., engaging in behavioral healthcare). VA leaders thought CTI implementation would standardize and improve Aftercare practices. Case managers and supervisors had limited experience implementing evidence-based practices and desired training with realistic case examples. Most had no prior knowledge of CTI, were highly satisfied with the training offered, and comfortable using CTI practices with HEVs. Staff at all agencies reported uncertainty about CTI’s alignment with Aftercare’s performance metrics. There was agency-level variation in other contextual factors impacting implementation, including pre-implementation case management supervision practices; variable levels of leadership buy-in; competing case manager responsibilities; and clinical documentation and reporting requirements.

**Implications for D&I Research**

CTI was successfully implemented in four Aftercare agencies that serve HEVs. Aftercare stakeholders found CTI acceptable and appropriate; there was consensus that CTI was a compatible and useful practice. To support national CTI scale-up in Aftercare, tangible CTI training that highlights the congruence of CTI with relevant performance metrics and documentation requirements, leadership engagement, staffing redundancy; and longitudinal implementation supports are likely to be crucial. Moreover, this pilot suggested that implementing CTI in diverse contexts requires balancing practice fidelity with adaptations that accommodate contextual differences across Aftercare settings. Variations in agency-level contextual factors necessitate tailored CTI implementation supports.

**Primary Funding Source**

Department of Veterans Affairs

### S157 School nurses as implementation champions: facilitators and barriers to supporting innovations to advance lgbtq+ health equity

#### Mary Ramos^1^, Daniel Shattuck^2^, Sonnie Davies^2^, Evelyn Byrd^2^, Bonnie Richard^3^, Janie Lee Hall^2^, Cathleen Willging^2^

##### ^1^University of New Mexico School of Medicine, Albuquerque, NM, USA; ^2^Pacific Institute for Research and Evaluation, Albuquerque, NM, USA; ^3^Pacific Institute for Research and Evaluation, Louisville, KY, USA

###### **Correspondence:** Daniel Shattuck (dshattuck@pire.org)

*Implementation Science 2023,* **18(Suppl 3):**S157

**Background**

In 2021, the National Association of School Nurses re-acknowledged the school nurse role in supporting the health and wellbeing of lesbian, gay, bisexual, transgender, and queer/questioning (LGBTQ+) students by helping to create LGBTQ+ friendly spaces, enabling access to resources, advocating for school-wide protections, and affirming young peoples’ identities. In a five-year randomized cluster-controlled trial that promoted the implementation of six LGBTQ+ supportive practices in New Mexico high schools, we conceptualized school nurses as pivotal champions for change. This presentation examines the facilitators and barriers school nurses faced in advancing LGBTQ+ health equity.

**Methods**

Qualitative data were drawn from interviews and focus groups conducted annually at schools randomized into an implementation condition. Throughout the study, a total of 24 nurses from 13 schools participated in 54 individual interviews. We used a thematic analytic approach to identify facilitators and barriers stemming from inner- and outer-contexts affecting school nurse involvement in implementation process. These data were analyzed in relation to the contents of implementation coach logs and participant demographic forms.

**Findings**

Several factors impacted school nurses’ effective championing for change. Facilitators included school nurses’ organizational and leadership skills, personal interest in supporting LGBTQ+ youth, relationships within schools, and district-level support. Additionally, nurses in larger schools could draw on a bigger pool of staff to support implementation, while those in smaller schools exerted wider personal influence. Barriers included time constraints, requirements to cover multiple schools, staffing shortages, and workloads that included responsibilities beyond the traditional scope of school nursing. With the exception of time constraints—which shaped the work of all participants—these challenges disproportionately affected school nurses in smaller and rural schools.

**Implications for D&I Research**

This study underscores the need to consider the reality of theorized implementation champions. The overlapping influences of outer- and inner- contexts and personal characteristics/attitudes affect school nurses’ capacity to lead and support implementation. Findings confirm that school nurses hold strategically important positions in schools for systemic innovations to address health equity; yet job duties, school and community contexts pose challenges that can undermine nurses’ involvement in change efforts.

**Primary Funding Source**

National Institutes of Health

### S158 Understanding dynamic sustainability to promote health equity during the COVID-19 pandemic: Community-led adaptations to cancer screening programs to address evolving contexts and community priorities

#### Savannah Alexander^1^, Ravali Mukthineni^1^, Detric Johnson^2^, Deborah Erwin^2^, Rachel Shelton^1^

##### ^1^Columbia University Mailman School of Public Health, New York, NY, USA; ^2^Roswell Park Comprehensive Cancer Center, Buffalo, NY, USA

###### **Correspondence:** Savannah Alexander (sa3921@cumc.columbia.edu)

*Implementation Science 2023,* **18(Suppl 3):**S158

**Background**

There is a need to advance understanding of dynamic sustainability, as implementers and researchers grapple with how to adapt evidence-based interventions/strategies over time to address evolving contexts and community needs while staying true to programs’ core functions. Studying such planned adaptations is crucial within the context of the COVID-19 pandemic, to advance understanding of what types of adaptations might reduce or exacerbate social/health inequities. Examining the dynamic sustainability of National Witness Project (NWP) before, during, and after the pandemic’s peak addresses this important gap. NWP is an NCI evidence-based, peer-led, community-based cancer screening program to address inequities among African-American women that has been nationally implemented for 30 years.

**Methods**

Data was collected using a prospective, mixed-methods comparative case study design at 3 timepoints over 4 years across 16 NWP sites of low, moderate, high sustainment levels (i.e., varying levels of program delivery over time). This analysis focused on understanding adaptations needed to promote program sustainability during the pandemic. Data includes surveys among 200 Lay Health Advisors, 16 Project Directors at 3 timepoints; in-depth interviews among a 72-participant sub-sample at 2 timepoints. Data was analyzed using SPSS, NVivo.

**Findings**

Participants identified the pandemic as the dominant challenge to site sustainability and implementer activity/retention, which exacerbated sustainability challenges in low-resource settings (e.g., limited funding/resources/organizational infrastructure). Participants described programmatic adaptations (e.g., transition to virtual program delivery/outreach; adaptations to address social needs/community priorities around COVID vaccination, food/housing insecurity) and organizational adaptations (e.g., building new infrastructure/partnerships) as critical to address inequities exacerbated by COVID and sustain the program. Sites with strong, trusted academic, public health/community, and health system partnerships were able to leverage resources and organizational infrastructure to facilitate these equity-focused adaptations. Participants specified strategies/capacity-building needed to dynamically support community-led adaptations to promote sustainability and health equity.

**Implications for D&I Research**

This study is one of the first to provide empirical insight into the intersection of health equity and dynamic sustainability during the pandemic. Our findings suggest that adaptations made to address social/health inequities are critical for long-term sustainability of program impact and delivery. This research advances growing empirical evidence on how to more explicitly address health equity in implementation science.

**Primary Funding Source**

American Cancer Society

### S159 Using evidence-informed interventions to improve health outcomes among people living with hiv: findings from the e2i initiative

#### Alex Keuroghlian^1^, Demetrios Psihopaidas^2^, Janet Myers^3^, Nicole Chavis^4^

##### ^1^Fenway Health, Boston, MA, USA; ^2^HRSA, Rockville, MD, USA; ^3^University of California San Francisco, San Francisco, CA, USA; ^4^Health Resources and Services Administration, Rockville, MD, USA

###### **Correspondence:** Alex Keuroghlian (akeuroghlian@fenwayhealth.org)

*Implementation Science 2023,* **18(Suppl 3):**S159

**Background**

The Health Resources and Services Administration’s HIV/AIDS Bureau (HAB) administers the Ryan White HIV/AIDS Program (RWHAP), which supports services for more than half a million people with HIV. Since 2010, HAB has increasingly leveraged program data, paired with insights from the field of implementation science (IS), to maximize the reach and impact of the RWHAP.

**Methods**

HAB funded an implementation science initiative between 2018-2022, *Using Evidence-Informed Interventions to Improve Outcomes among People Living with HIV* (E2i), to identify and implement evidence-informed interventions in four focus areas with known disparities in HIV care continuum outcomes: transgender women, Black men who have sex with men (MSM), behavioral health integration, and addressing trauma. Twenty-six RWHAP recipient organizations implemented 11 evidence-informed interventions targeted to one of the four focus areas. We employed the HAB IS framework, informed by the Proctor Model for Implementation Research, to evaluate implementation and change in client engagement in HIV care. In parallel, the initiative developed dissemination products to support future replication and scale up of E2i interventions at HIV direct service organizations.

**Findings**

Almost all (25 of 26) sites were able to implement the selected interventions and the majority of clients received core intervention components (88%-100% of clients received any exposure to the interventions and 54%-100% received core components of the selected interventions). Almost all (10-11) interventions were associated with increases in suppression of HIV viral load, although only 3 interventions demonstrated statistically significant changes due to small sample sizes. Interventions that had well-defined, discrete, time-limited core activities (i.e. linkage to care; or defined curriculum) were more effective and cost-effective than system-level interventions and those that identified fewer clients or clients who were already fully engaged in HIV care. Implementation toolkits for each intervention were released in July 2022.

**Implications for D&I Research**

HAB’s E2i initiative is now a proven model for evaluating the implementation of innovative interventions to improve outcomes for people with HIV along the HIV care continuum and supporting their replication and scale up. Widespread dissemination of the products of E2i and other IS initiatives can help achieve optimal health outcomes and end the HIV epidemic in the US.

**Primary Funding Source**

Health Resources and Services Administration

### S160 Using implementation science to longitudinally evaluate a diabetes prevention and management intervention in an underserved south asian population in new york city

#### Shahmir H. Ali^1^, Deborah Onakomaiya^2^, Tanzeela Islam^3^, Jennifer Zanowiak^3^, Shinu Mammen^3^, Sadia Mohaimin^3^, Laura C. Wyatt^3^, Sahnah Lim^3^, Nadia S. Islam^3^

##### ^1^Department of Social and Behavioral Sciences, New York University School of Global Public Health, New York, NY, USA; ^2^Vilcek Institute of Graduate Biomedical Sciences, New York University Grossman School of Medicine, New York, NY, USA; ^3^Department of Population Health, New York University Grossman School of Medicine, New York, USA

###### **Correspondence:** Shahmir H. Ali (sha371@nyu.edu)

*Implementation Science 2023,* **18(Suppl 3):**S160

**Background:** Community-clinical linkage models demonstrate potential for improving health outcomes in underserved communities, but factors that impact their successful implementation are understudied. This study aims to longitudinally evaluate the implementation of community-clinical linkage model designed to improve diabetic management and prevention among South Asians.

**Methods:** Between 2019 and 2021, an annual interview was conducted among participating intervention stakeholders on perceptions regarding intervention implementation and intra-team dynamics. Stakeholders included 5 research staff (n=10 interviews), 7 community health workers (CHWs) (n=21 interviews), 7 representatives from community-based organizations (CBOs) (n=14 interviews), and 16 primary care practice providers/staff (n=16 interviews) involved in the intervention. Research staff, CHWs, and CBO partners were interviewed annually (with some staff changes between years), while providers were interviewed once. Interviews were coded using the Consolidated Framework for Implementation Research (CFIR).

**Findings:** Within the *Inner Setting*, the triangular relationship between healthcare providers/staff, research staff, and CHWs evolved significantly throughout the intervention in response to changes in stakeholder responsibilities and constraints in the capacity of healthcare providers/staff to participate in the study. *Outer Setting* dynamics revealed community collaborations to be crucial in informing intervention success (e.g., through involvement in or dissemination of intervention activities). Over time, these partnerships expanded beyond the intervention (e.g., intra-CBO collaborations, support from academic partners to secure additional resources for CBO activities). *Intervention Characteristics* highlighted iterative changes in the intervention complexity, delivery, and culturally relevant content, including the central role of CHWs in adapting the intervention to an evolving resource and social landscape. Occupation, family structure, and technological capacity were particularly important *Characteristics of Individuals*. Within *Process*, multi-component fidelity assessments were crucial in ensuring consistency as the intervention was adapted to the COVID context, and the protocolization of lessons learned were particularly informative in planning activities as the pandemic evolved. A fourth round of interviews are currently being conducted and will be presented with this data.

**Implications for D&I Research:** Findings revealed unique facilitators and challenges in the implementation of a complex diabetes intervention for South Asians in NYC and provide insights for implementation scientists seeking to develop sustainable, dynamic, and community-sensitive health interventions aimed at underserved racial and ethnic minority populations.

**Primary Funding Source**

National Institutes of Health

### S161 Evaluation of an integrated case management pilot aligning medical and social services for high-risk, high-need patients using the health equity implementation framework

#### Nadia Safaeinili^1^, Emmeline Chuang^1^, Mark Fleming^1^, Shoba Ramanadhan^2^, Amanda Brewster^1^

##### ^1^University of California, Berkeley, Berkeley, CA, USA; ^2^Dana-Farber Cancer Institute, Boston, MA, USA

###### **Correspondence:** Nadia Safaeinili (nadiasaf@berkeley.edu)

*Implementation Science 2023,* **18(Suppl 3):**S161

**Background:** Health systems increasingly use case management programs to integrate social and medical services to support health equity for high-risk, high-need patients. Limited evidence exists about key components of integrated case management program implementation, especially from a health equity perspective. This longitudinal qualitative study applied a health equity implementation framework to examine patient, frontline case manager, and administrator perspectives on implementation of a multidisciplinary, team-based case management pilot serving high-risk, high-need MediCal patients.

**Methods:** We conducted 86 semi-structured phone interviews with patients (n=31), case managers (n=41), and county administrators (n=14) across two time points using purposive sampling to identify a representative sample. Interviews were transcribed and coded using an inductive-deductive approach informed by the Health Equity Implementation Framework (HEIF) to identify facilitators and barriers to equitable implementation.

**Findings:** Supportive characteristics of the innovation included prevention-focused nursing leadership in designing the pilot and development of a predictive algorithm incorporating socioeconomic factors to determine pilot eligibility. Encounters between patients and case managers centered around a hierarchically flat, multidisciplinary team of case managers leveraging varied expertise. Provider factors surfaced the importance of case management and supervisory staff with diverse backgrounds and lived experience. Challenges included the invisible emotional labor of case management work. Patient factors highlighted the interdependent nature of patient needs, from emotional support to access to stable housing. In the inner and outer context, initial challenges included shifting health system values to prioritize preventative care and social service integration. Strong inter- and intra-organizational relationships were essential to implementation success and equitable access to resources. National recognition of the social determinants of health’s impact on health and corresponding funding from the Centers from Medicare and Medicaid Services provided needed resources to achieve the innovation, restructuring, and partnerships needed to implement the pilot.

**Implications for D&I Research:** Factors central to equitable implementation included equity-focused leadership, multidisciplinary teams with lived experience, eligibility criteria attentive to social factors, strong partnerships within and across organizations, and sufficient resources. Important challenges included an initial mismatch in value alignment within the organization, and case manager administrative and emotional burden, among others.

**Primary Funding Source**

The Robert Wood Johnson Foundation

### S162 Rapid cycle designs to adapt interventions for covid-19 in safety-net healthcare systems

#### Chelsey Schlechter

##### University of Utah, Salt Lake City, USA

###### **Correspondence:** Chelsey Schlechter (chelsey.schlechter@hci.utah.edu)

*Implementation Science 2023,* **18(Suppl 3):**S162

**Background:** The dynamic nature of the COVID-19 pandemic has resulted in interventions that need to be rapidly ‘developed, adapted, employed, and abandoned’ to keep pace with scientific and policy advances, as well as the status of the pandemic. While still allowing rigorous evaluation, rapid-cycle designs enable iterative adaptation of interventions by incorporating data from multiple sources, including policy (e.g., vaccination approvals, eligibility), setting capacity (e.g., clinic testing capacity, vaccine availability), and stakeholder priorities (e.g., infection ‘hot-spots’ for testing, priority groups for vaccination). The objective of this presentation is to describe the application of a rapid-cycle design and adaptation process and exemplar adaptations in SCALE-UP Utah, which addressed COVID-19 among patients served by seven Community Health Center systems (CHCs).

**Methods:** SCALE-UP Utah is a two-arm, patient-level randomized clinical trial conducted with CHCs and their patients across Utah. The rapid-cycle design and adaptation process included 1) assessing context and determining relevant models/frameworks; 2) determining core and modifiable components of interventions; and 3) conducting iterative adaptations using Plan-Do-Study-Act (PDSA) cycles. PDSA cycles included: *Plan*. Gather information from potential adopters/implementers (e.g., CHC staff/patients) and design initial interventions; *Do*. Implement interventions in a single CHC or patient cohort; *Study*. Examine process, outcome, and context data (e.g., infection rates); and, *Act*. If necessary, refine interventions based on process and outcome data, then disseminate interventions to other CHCs and patient cohorts.

**Findings:** Rapid-cycle, PDSA-based adaptations were made to adapt to evolving COVID-19 related needs. Near real-time data used for adaptation included data on infection hot-spots, CHC capacity, stakeholder priorities, local/national policies, and testing/vaccine availability. Adaptations were made to study design, intervention content, and intervention cohorts. Decision-making included multiple stakeholders (e.g., State Department of Health, Primary Care Association, CHCs, patients, researchers). Over the two year project, adaptations resulted in 37 different workflows and 227 different patient cohorts.

**Implications for D&I Research:** Using a community-engaged, rapid-cycle design process to adapt interventions may improve the relevance and timeliness of interventions for CHCs and other settings that provide care to populations experiencing health inequities. This process was acceptable to CHC systems for addressing COVID-19, and could be used to address other healthcare challenges.

**Primary Funding Source**

National Institutes of Health

### S163 Population health management interventions to address covid-19 among safety-net healthcare systems: preliminary results from the scale-up utah randomized clinical trial

#### Guilherme Del Fiol

##### University of Utah, Salt Lake City, UT, USA

###### **Correspondence:** Guilherme Del Fiol (guilherme.delfiol@utah.edu)

*Implementation Science 2023,* **18(Suppl 3):**S163

**Background:** Populations that have been historically marginalized (racial/ethnic minority, rural, low socioeconomic status) are disproportionately affected by COVID-19. Increasing the reach of interventions to address COVID testing/vaccination at scale among these populations is crucial to improving health inequities.

**Methods:** Eligible patients (i.e., ≥ 18, seen at a participating Community Health Center (CHC) system in the last 3 years ) were randomized to text messaging (TM) or text messaging+patient navigation (TM+PN). Patients in TM received bidirectional messages including a brief message regarding risk, along with an offer for COVID-19 testing/vaccination. Patients who replied YES received information for scheduling testing/vaccination or were mailed at-home test kits. Messages were sent in English or Spanish based on patients’ primary language in the EHR. Patients randomized to TM+PN received identical messages to the TM group and were potentially eligible to receive PN via a phone call. Navigation was delivered using a resource conserving approach such that patients only received PN calls if they replied to a TM indicating interest in COVID-19 testing/vaccination or requested a PN call.

**Findings:** Patients (n=99,839) from seven CHC systems were randomized; 54% were female, 40% Hispanic/Latino, 30% Spanish Primary Language, 21% rural (RUCA≥4), with a mean age of 42 years (SD=16.2). Ninety-six percent of patients (n=96,020) had a valid cell phone number that could receive TM and were sent at least 1 TM. Of the patients in TM who received at least 1 message (n=47,919), 35% responded to at least 1 message (29% [n=13,707] responded to at least one message and never opted-out; 6% [n=2,946] responded to at least one message, then subsequently opted-out); 14% (n=6,650) opted-out as their only response; and 56% (n=27,562) did not respond to any messages. Of the patients in TM+PN who received at least 1 message (n=48,101), 28% (n=13,266) responded to at least one message and never opted-out; 6% (n=3,110) responded to at least one message, then subsequently opted-out; 13% (n=6,057) opted-out as their only message response; and 60% (n=28,778) did not respond to any messages. Results of PN are forthcoming.

**Implications for D&I Research:** TM and PN are feasible strategies for population-level scale-up for reaching diverse groups of CHC patients.

**Primary Funding Source**

National Institutes of Health

### S164 Ensuring health equity: predictors of patient engagement with population health management (phm) interventions to address covid-19 in safety-net healthcare systems

#### David Wetter

##### University of Utah, Salt Lake City, UT, USA

###### **Correspondence:** David Wetter (david.wetter@hci.utah.edu)

*Implementation Science 2023,* **18(Suppl 3):**S164

**Background:** Population health management (PHM) interventions may inadvertently exacerbate health inequities by differentially benefitting certain subgroups. Consequently, understanding variability in engagement by subgroups may inform intervention adaptation to improve equitable implementation of interventions.

**Methods:** Patient demographics were obtained from the Electronic Health Record (EHR) of participating CHCs. Engagement outcomes were obtained from the PHM platform. Logistic and multinomial models were used to examine the association between randomization condition, patient demographics (i.e., rurality; ethnicity; gender; age; message language [i.e., preferred language]) and outcomes. Outcomes included valid cell phone status, TM-Conversation (i.e., responded to at least one message and did not opt-out); TM-Opt-Out (i.e., opted-out as only response); TM-No Response (i.e., did not respond to any messages).

**Findings:** Patients (n=99,839) from seven CHC systems were randomized to TM or TM+PN and were included in at least one workflow. Ninety-six percent (n=96,020) of patients had a valid cellphone. Patients with a valid cell phone were significantly less likely (*p*≤.05) to be rural (β=-0.181, SE=0.056) or older (β=-0.181, SE=0.056); and significantly more likely (*p*≤.05) to be Hispanic/Latino (β=0.539, SE=0.054), female (β=0.373, SE=0.034), or had Spanish message language (β=0.789, SE=0.062). Patients who had TM–Conversation were significantly less likely (*p*≤.05) to be in TM+MAPS (β=-0.053, SE=0.014), rural (β=-0.177, SE=0.023), Hispanic/Latino (β=-0.102, SE=0.022), or receive Spanish messages (β=-0.102, SE=0.022); and were significantly more likely (*p*≤.05) to be female (β=0.230, SE=0.015), older (β=0.019, SE=0.005). Patients who had TM-Opt-Out were significantly less likely (*p*≤.05) to be in TM+MAPS (β=-0.110, SE=0.019), Hispanic/Latino (β=-0.376, SE=0.030), older (β=-0.007, SE=0.001), or receive Spanish messages (β=-0.427, SE=0.034); and were significantly more likely (*p*≤.05) to be female (β=0.088, SE=0.020). Patients who did not respond were significantly less likely (*p*≤.05) to be female (β=-0.235, SE=0.014), and older (β=-0.013, SE=0.000); and were significantly more likely (*p*≤.05) to be in TM+PN (β=0.096, SE=0.013), rural (β=0.053, SE=0.021), Hispanic/Latino (β=0.253, SE=0.020), or receive Spanish messages (β=0.189, SE=0.021).

**Implications for D&I Research:** Engagement with PHM interventions varied by demographic characteristics, consequently interventions may need to be adapted for specific subgroups.

**Primary Funding Source**

National Institutes of Health

### S165 Organizational commitments to racial equity as determinants of implementing an evidence-based intervention for young black sexual minority men living with hiv: a mixed-methods study in chicago and alabama

#### Kristen Ethier^1^, Maggie Denning^2^, Emma Sophia Kay^3^, Justin Knox^2^, Brie Scrivener^4^, D. Scott Batey^5^, Alida Bouris^1^

##### ^1^University of Chicago, Chicago, IL, USA; ^2^Columbia University, New York, NY, USA; ^3^Birmingham AIDS Outreach, Birmingham, AL, USA; ^4^University of Alabama at Birmingham, Birmingham, AL, USA; ^5^University of Alabama, Birmingham, AL, AL, USA

###### **Correspondence:** Kristen Ethier (kethier@uchicago.edu)

*Implementation Science 2023,* **18(Suppl 3):**S165

**Background**

Scholars have called for greater attention to how racism and processes of racialization shape implementation of evidence-based programs (EBPs). While implementation science (IS) highlights the importance of internal and external contexts in implementation, the roles of racism and racialization remain under-theorized, with limited empirical data on how they shape organizational dynamics.

**Methods**

We conducted a sequential explanatory mixed-methods study with staff from five organizations in Chicago (n=2) and Alabama (n=3) that will implement Project nGage, a social network support intervention promoting HIV care engagement and viral suppression among young Black sexual minority men. In phase one, N=64 staff completed a closed-ended survey with items on organizational culture, implementation climate, and support for EBPs derived from the Consolidated Framework for Implementation Research. Items on organizational commitments to racial justice were selected based on literature and a modified Delphi process with a 19-person implementation team. Site-level means were calculated, which informed a purposive sampling frame and focus group protocol. Focus groups were conducted with n=39 frontline, leadership, and implementation staff. Rapid Qualitative Analysis examining site- and staff-level themes was conducted by seven researchers.

**Findings**

Survey data showed variation in site-level means for all domains including racial justice, with the Alabama sites having a stronger implementation climate. Qualitative themes contextualized survey data and yielded support for Ray’s^1^ Theory of Racialized Organizations. While site leadership viewed racial justice as integral to their mission, frontline staff were often unaware of how the organization challenged existing racial hierarchies. Frontline staff noted that people of color were prioritized for frontline but not for leadership positions. In some sites, staff described mistrust in the organization’s ability to support employees and follow through with action and support for racial justice.

**Implications for D&I Research**

Findings highlight the importance of utilizing organizational theories of racialization in IS research. Work is needed to build a science for understanding and operationalizing a racial justice lens in implementation, especially with respect to understanding how these dynamics shape organizational culture, staff attitudes and retention, and leadership’s ability to respond to ongoing acts of racial injustice.

**Primary Funding Source**

National Institutes of Health

### S166 Examining anti-racist approaches in an ongoing trial implementing universal family psychosocial risk screening in pediatric cancer

#### Janet Deatrick^1^, Lamia Barakat^2^, Michele Scialla^3^, Anne Kazak^3^

##### ^1^University of Pennsylvania, Philadelphia, PA, USA; ^2^The Children's Hospital of Phiadelphia, Philadelphia, PA, USA; ^3^Nemours Children's Health System, Wilmington, DE, USA

###### **Correspondence:** Anne Kazak (anne.kazak@nemours.org)

*Implementation Science 2023,* **18(Suppl 3):**S166

**Background**

Using the Anti-Racism Framework (ARF) of Shelton and colleagues, we examined our current trial (American Cancer Society RSG-19-122) that is focused on promoting health equity by implementing universal family psychosocial risk screening in 18 pediatric oncology sites in the U.S. The ARF outlines best practices for constructing implementation research that acknowledges and considers the effects of racism. It includes constructs such as stakeholder engagement; conceptual frameworks and models; development, selection, and adaptation of evidence-based interventions; evaluation approaches, and implementation strategies. The purpose of this report is to examine ways in which our current implementation trial is consist with anti-racist approaches.

**Methods**

Sources of data from our ongoing implementation trial were semi-structured qualitative interviews with 19 stakeholders (parent advocates, clinicians, leaders in professional organizations and healthcare policy) to refine implementation strategies, the study protocol, and implementation strategy materials. Anti-racism approaches were identified from these data and categorized by the study team using crosswalks to link to the ARF.

**Findings**

Identified approaches from the trial consistent with the ARF were stakeholder engagement (e.g., interviews with key stakeholders); conceptual frameworks (e.g., Interactive Systems Framework for Dissemination and Implementation; Psychosocial Preventive Health Model); evidence-based interventions/ implementation strategies (e.g., multilevel and structural intervention strategies); and evaluation approaches (e.g., mixed methods process and outcome measures). We also identified the importance of implementation approaches that are consistent with health care policy.

**Implications for D&I Research**

Many ongoing implementation trials were designed prior to the more recent focus on anti-racism in implementation science. Nonetheless, these trials can be examined by reviewing the consistency of approaches with the ARF. This process can help inform the design of future trials.

**Primary Funding Source**

American Cancer Society

### S167 Implementation science partnerships to advance health equity in healthcare settings that serve socially at-risk communities

#### Kelly Aschbrenner^1^, Gina Kruse^2,3^, Jen Cruz^4^, Rebecca Lee^5^, Huy Nguyen^6^, Maria Celli^7^, Cristina Huebner Torres^8^, Karen Emmons^9^

##### ^1^Geisel School of Medicine at Dartmouth College, Nashua, NH, USA; ^2^Massachusetts General Hospital, Boston, MA, USA; ^3^Harvard Medical School, Boston, MA, USA; ^4^Harvard T.H. Chan School of Public Health, Boston, MA, USA; ^5^Harvard T.H. School of Public Health, Boston, USA; ^6^DotHouse Health, Boston, USA; ^7^Brockton Neighborhood Health Center, Brockton, MA, USA; ^8^Caring Health Center, Springfield, MA, USA; ^9^Harvard University, Boston, MA, USA

###### **Correspondence:** Kelly Aschbrenner (kelly.aschbrenner@dartmouth.edu)

*Implementation Science 2023,* **18(Suppl 3):**S167

**Background:** Implementation science partnerships between academics and healthcare professionals can accelerate progress toward health equity goals. While health equity is central to the mission of Community Health Centers (CHCs), there remain gaps in access and benefit from this critical safety net. Implementation science partnerships offer value to healthcare partners seeking to equitably implement evidence-based interventions. This presentation provides an in-depth case example of an academic-community partnered approach to identifying priority areas where implementation science can be applied to advance health equity in healthcare settings, in particular those that serve socially at-risk communities.

**Methods:** Our team at the Implementation Science Center for Cancer Control Equity (ISCCCE) partnered with leadership and staff at CHCs and the Massachusetts League of Community Health Centers (MLCHC) to qualitatively explore their perspectives on health equity. We conducted interviews with 31 leaders and staff across 10 CHCs investigating their perspectives on identifying, measuring, and addressing healthcare inequities. We analyzed the data using deductive coding to facilitate rapid analysis. We presented findings in an Implementation Learning Community (ILC) where CHC leaders facilitated discussions with other leaders and staff to interpret results and identify action areas for research and practice.

**Findings:** Over 50 CHC leaders and staff attended the ILC. Qualitative codes from the interviews related to identifying and measuring healthcare inequities were used to develop cross-site suggestions for improving data systems, structures and ways of entering and analyzing data. CHC participants confirmed qualitative findings related to challenges to prioritizing health equity including the funding model, underuse of available data, and lack of language resources. Facilitated discussions among CHC leaders and staff identified at least two key considerations for equity-focused implementation research and practice: 1) navigating the tension between wanting to address root cause social determinants of inequities and the scope and resources available to address healthcare inequities; and 2) decolonizing academic and scientific research through meaningful collaboration with communities for data collection, interpretation and use of data.

**Implications for D&I Research:** This study highlights how equity-centered implementation science partnerships between academics and healthcare professionals can identify areas for action to advance health equity in healthcare settings that serve socially at-risk communities.

**Primary Funding Source**

National Institutes of Health

### S168 How measurement science influenced the design of a pay-for-equity program

#### Mark Friedberg, Gabriella Silva

##### Blue Cross Blue Shield of Massachusetts, Boston, MA, USA

###### **Correspondence:** Mark Friedberg (mark.friedberg@bcbsma.com)

*Implementation Science 2023,* **18(Suppl 3):**S168

**Background:** In 2020, Blue Cross Blue Shield of Massachusetts (BCBSMA) renewed its focus on the equity of health care and health outcomes. Key components of BCBSMA’s equity strategy have included publishing a racial and ethnic equity report to foster transparency and accountability for improvement, giving providers confidential equity reports on care they deliver, convening an equity improvement collaborative, and introducing pay-for-equity (P4E) financial incentives into the Alternative Quality Contract (AQC), an accountable care model originally introduced in 2009. When implemented on January 1, 2023, BCBSMA’s P4E program will be among the United States’ first equity focused ACO incentives.

**Methods:** In 2021, BCBSMA convened AQC providers and community members to develop and refine 12 design principles for P4E. These principles include, for example, “Emphasize collaboration over competition between provider groups,” “Do not pay for equity improvements resulting from performance declines,” “Do not penalize providers who serve more diverse patient populations,” and “Apply BCBSMA’s longstanding standards for measurement validity and reliability to pay-for-equity.” Using these principles as guardrails, BCBSMA leaders and analysts then developed P4E program specifications. Ensuring acceptable measurement reliability for equity measures (differences between racial and ethnic categories on quality measures) required application of Monte Carlo simulation. Results of this simulation helped determine which AQC quality measures were eligible for P4E.

**Findings:** To satisfy the P4E design principles, BCBSMA constructed P4E as a within-provider equity improvement incentive: providers will be paid more if equity improves over time, with additional bonuses if providers cumulatively achieve market-wide equity improvements. But if quality decreases significantly for any racial or ethnic group, a provider group will receive zero equity payout on that measure. Due to the reliability criterion, AQC provider groups with adequate sample size and racial/ethnic membership diversity were eligible for P4E for approximately 25-50% of their AQC quality measures: those with the greatest baseline inequities affecting the largest numbers of minoritized members.

**Implications for D&I Research:** Via a participatory process to identify design principles and by application of measurement science, it is possible to develop and implement explicit equity incentives within an ACO-style contract. BCBSMA’s P4E model could serve as a basis for other payers’ equity-focused incentive programs.

**Primary Funding Source**

Blue Cross Blue Shield of Massachusetts

